# Supplementary material for: C-Terminal 1-Aminoethyltetrazole-Containing Oligopeptides as Novel Alanine Racemase Inhibitors
Source: Molecules. 2020 Mar 13;25(6):1315. doi: 10.3390/molecules25061315 (PMC7145296; doi:10.3390/molecules25061315)

Supporting information

C-terminal 1-aminoethyltetrazole containing oligopeptides as novel alanine racemase inhibitors

Laszlo A. Kondacs [1], Sylvain Orenga [2], The late Rosaleen J. Anderson [1], Emma C. L. Marrs [3], John D. Perry [3], Mark Gray [1*]

[1] Sunderland Pharmacy School, University of Sunderland, Sunderland SR1 3SD, U.K.
[2] Research & Development Microbiology, bioMérieux, 38390 La Balme-les-Grottes, France
[3] Department of Microbiology, Freeman Hospital, Newcastle upon Tyne NE7 7DN, U.K.

Methyl-(2*S* or *R*)-2-{[(benzyloxy)carbonyl]amino}propanoate **6a**:

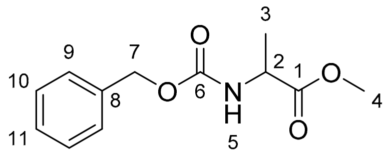

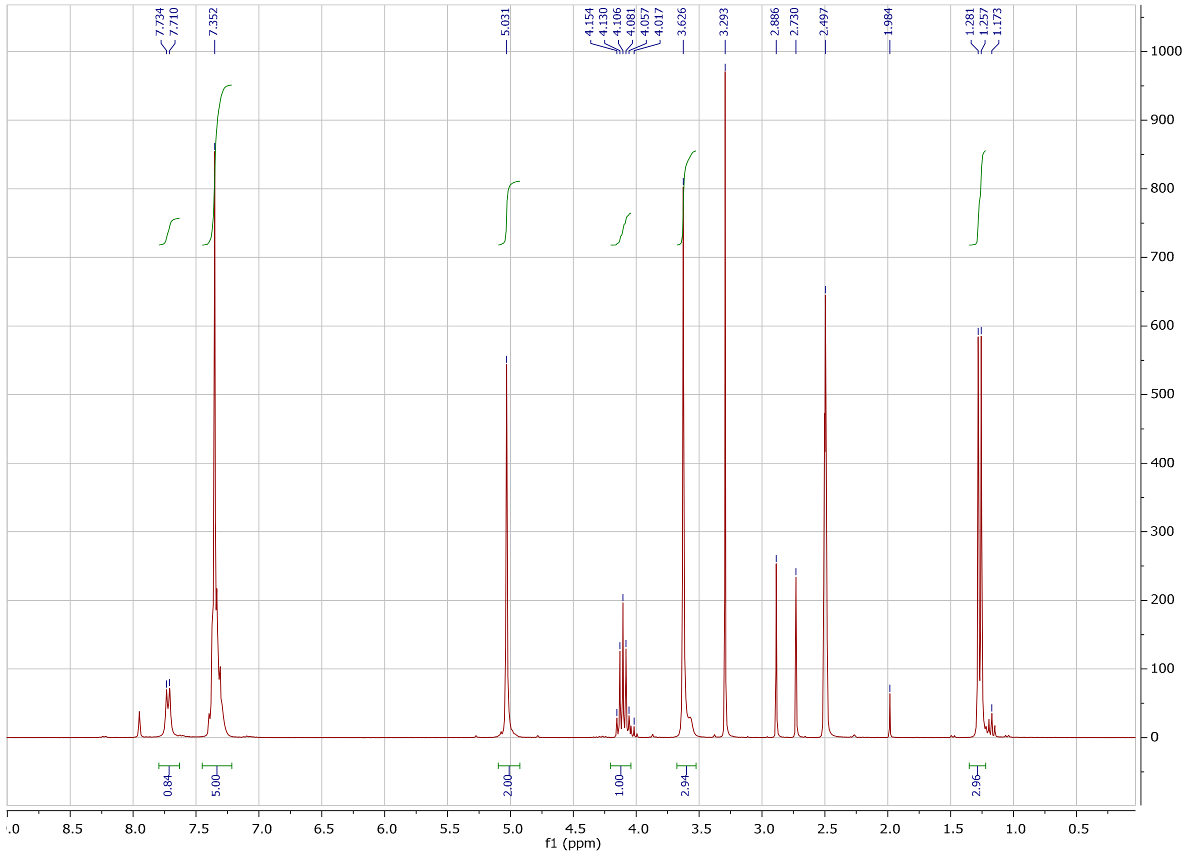

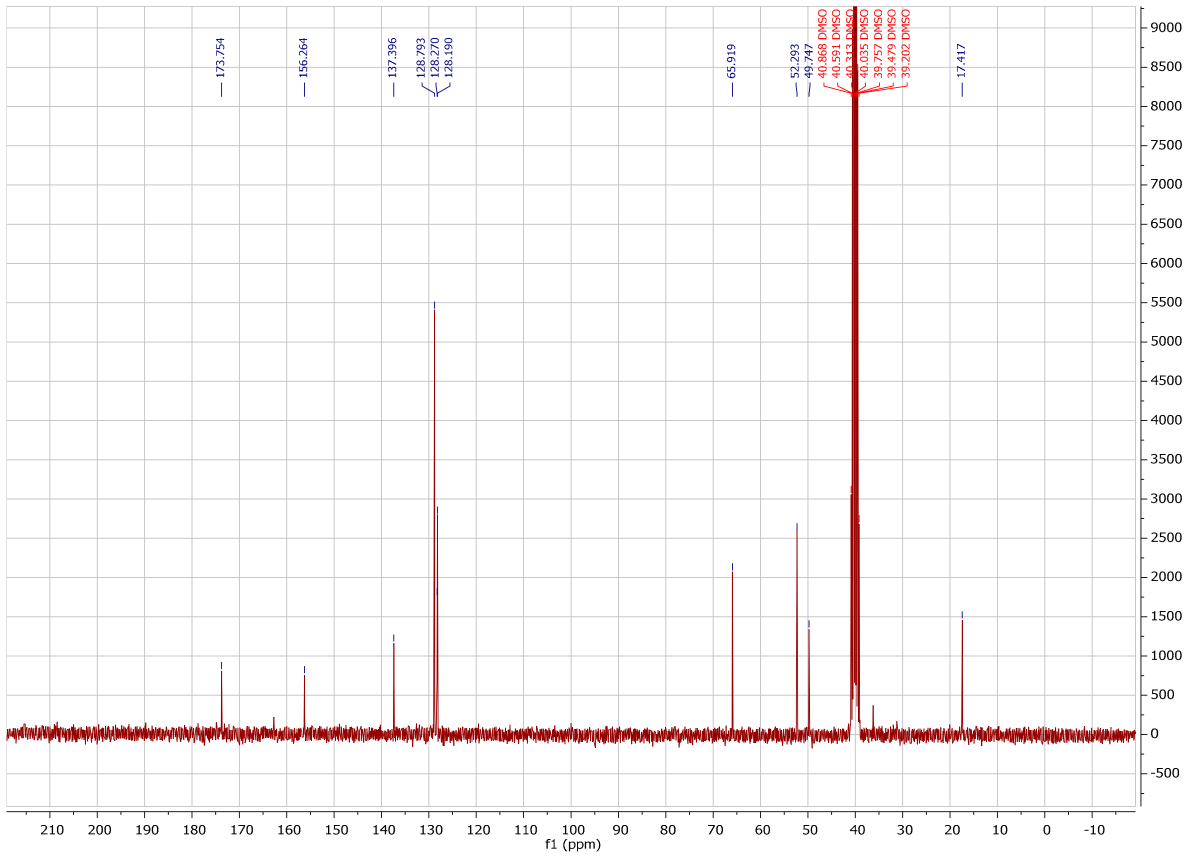

Benzyl-*N*-[(1*S* or *R*)-1-carbamoylethyl]carbamate **7a**:

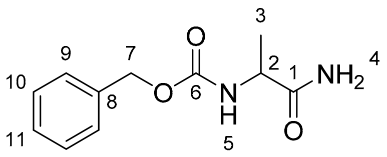

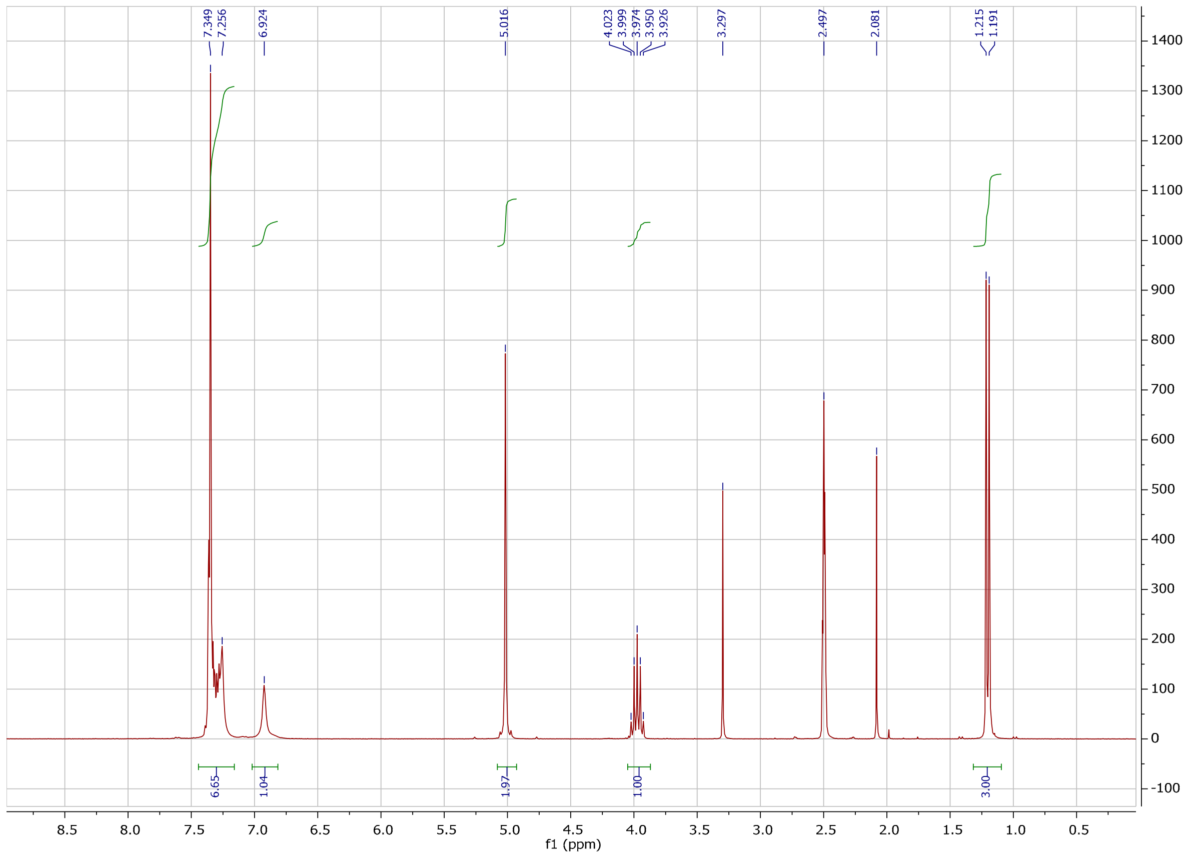

7.349
7.256
6.924
5.016
4.023
3.999
3.974
3.950
3.926
3.297
2.497
2.081
1.215
1.191
6.65
1.04
1.97
1.00
3.00
f1 (ppm)

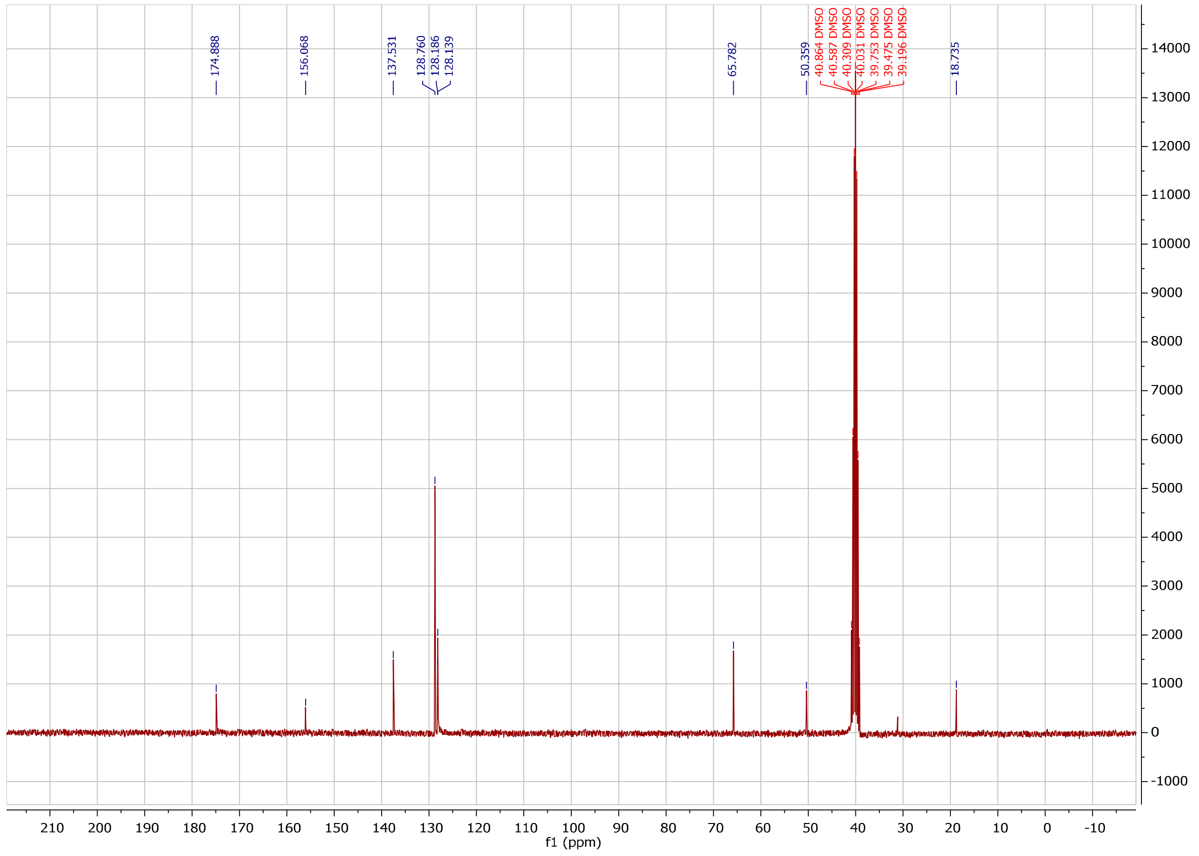

174.888
156.068
137.531
128.760
128.186
128.139
65.782
50.359
40.864 DMSO
40.587 DMSO
40.309 DMSO
40.031 DMSO
39.753 DMSO
39.475 DMSO
39.196 DMSO
18.735
f1 (ppm)

Benzyl-N-[(1*S* or *R*)-1-cyanoethyl]carbamate **8a**:

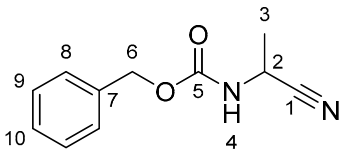

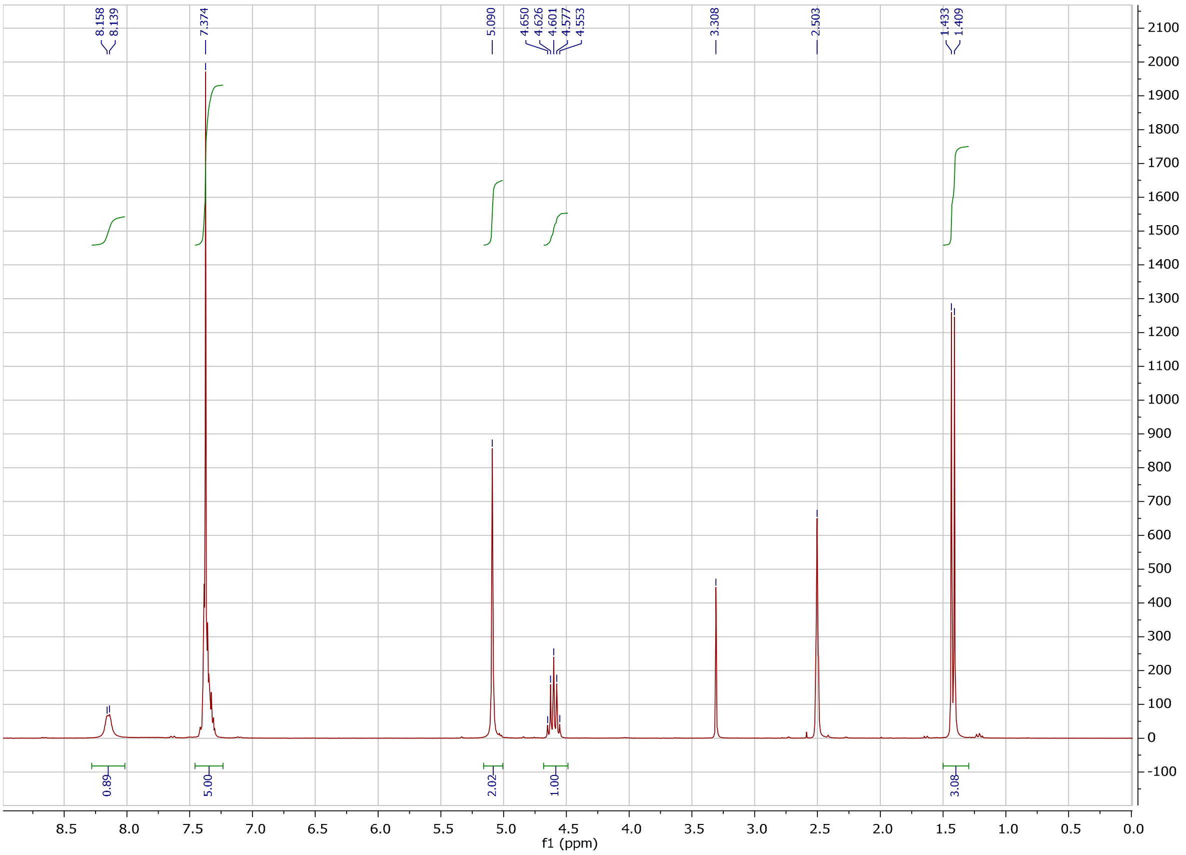

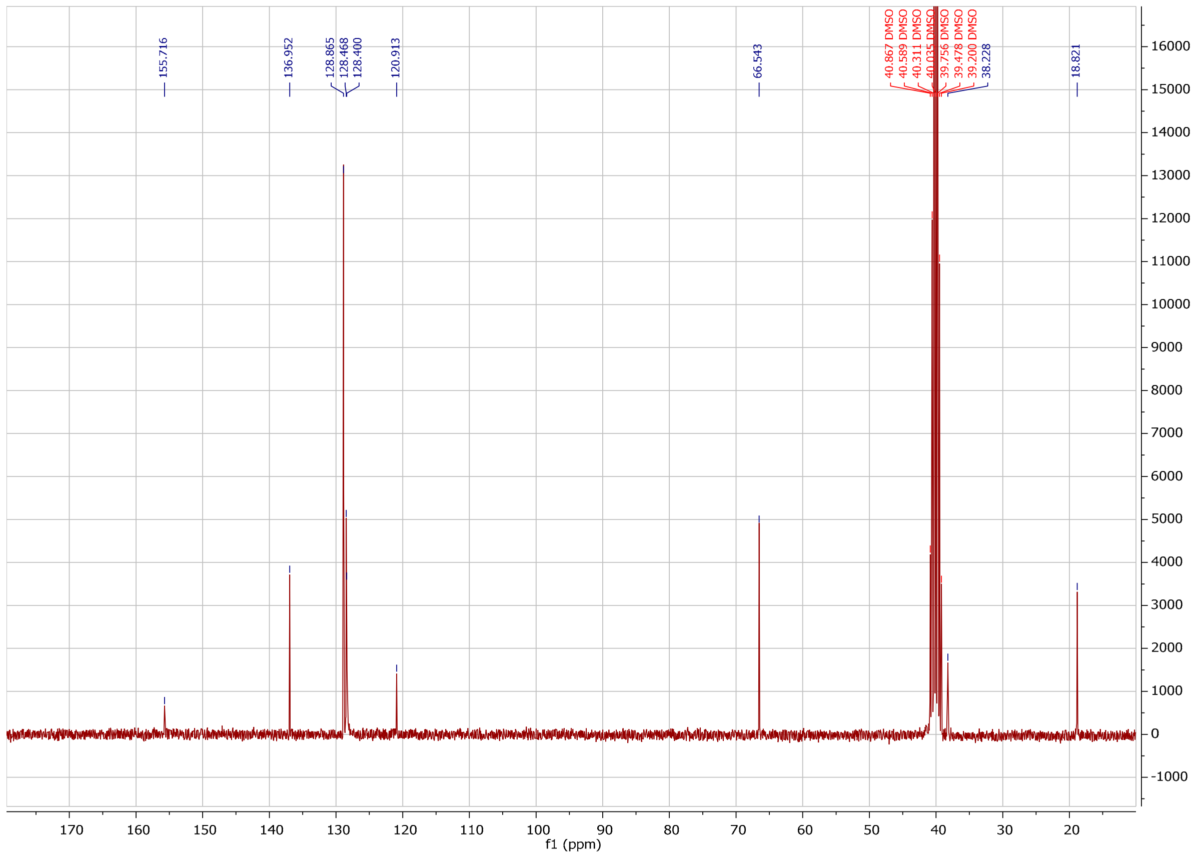

Benzyl-*N*-[(1*S* or *R*)-1-(1*H*-1,2,3,4-tetrazol-5-yl)ethyl]carbamate **9a**:

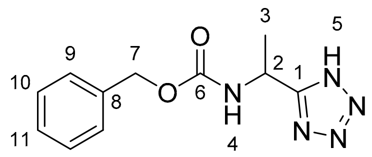

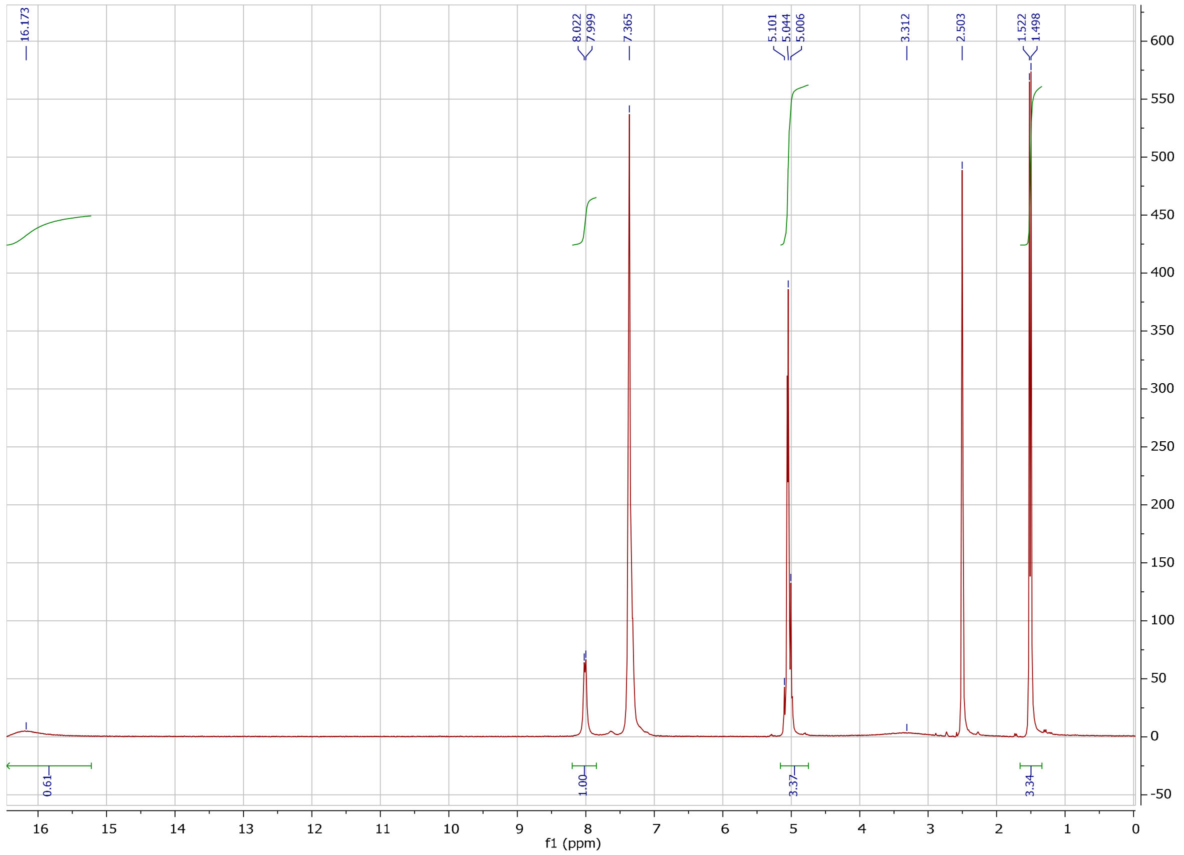

16.173
8.022
7.999
7.365
5.101
5.044
5.006
3.312
2.503
1.522
1.498
0.61
1.00
3.37
3.34
f1 (ppm)

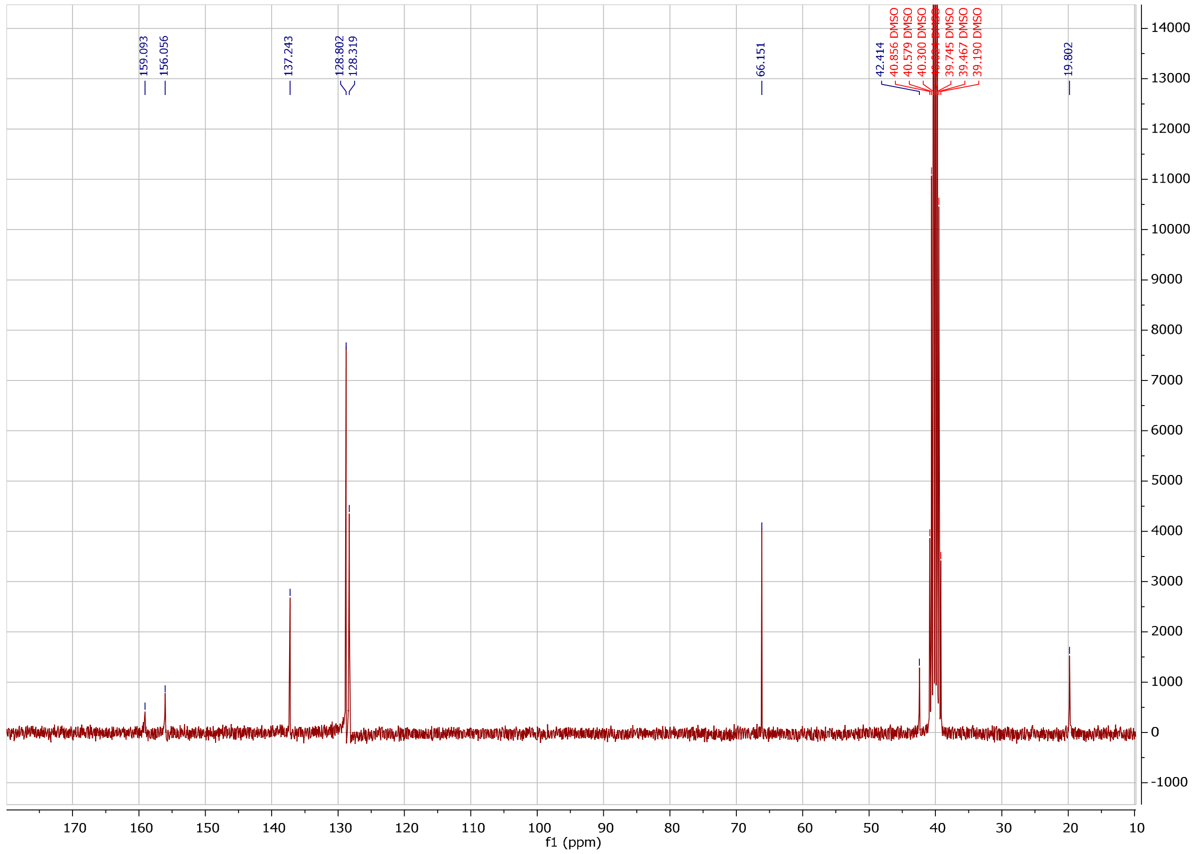

159.093
156.056
137.243
128.802
128.319
66.151
42.414
40.856 DMSO
40.579 DMSO
40.300 DMSO
39.745 DMSO
39.467 DMSO
39.190 DMSO
19.802
f1 (ppm)

(1*S* or *R*)-1-(1*H*-1,2,3,4-tetrazol-5-yl)ethan-1-amine **4a**:

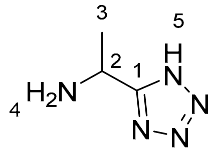

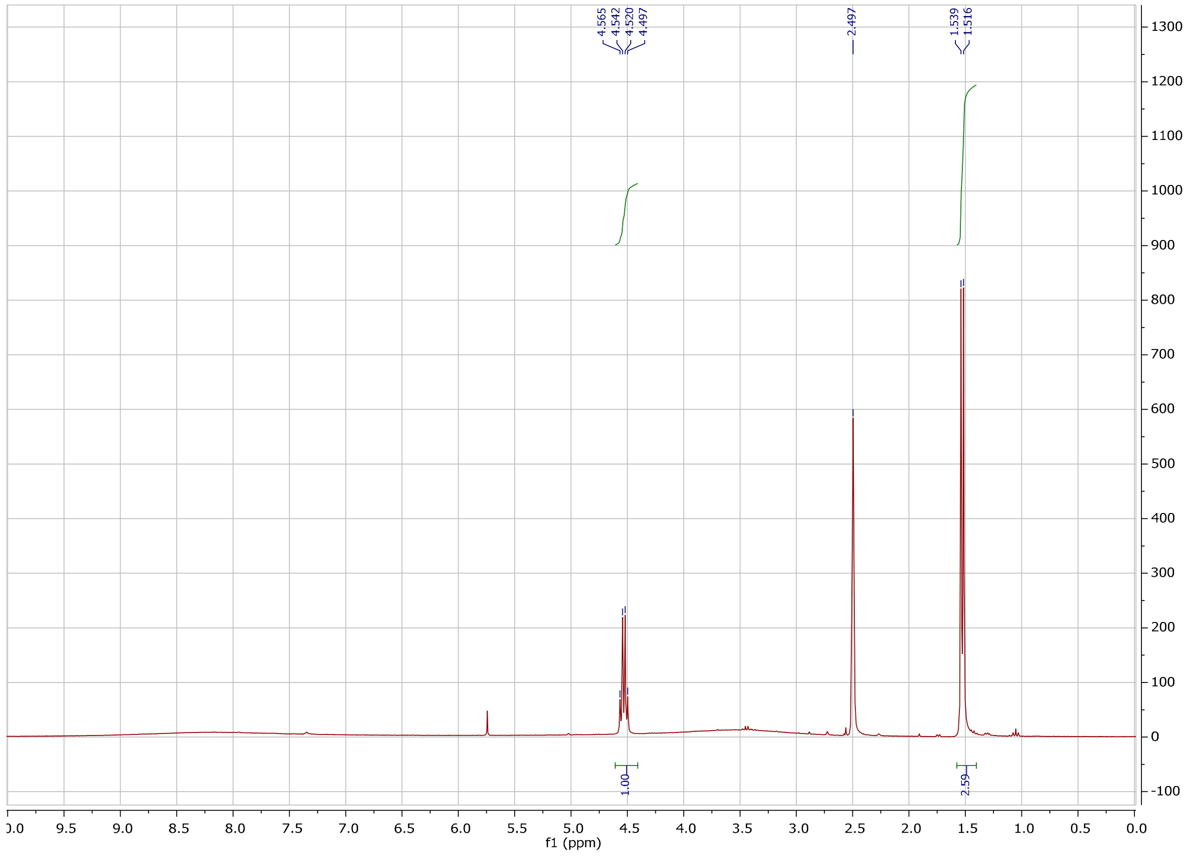

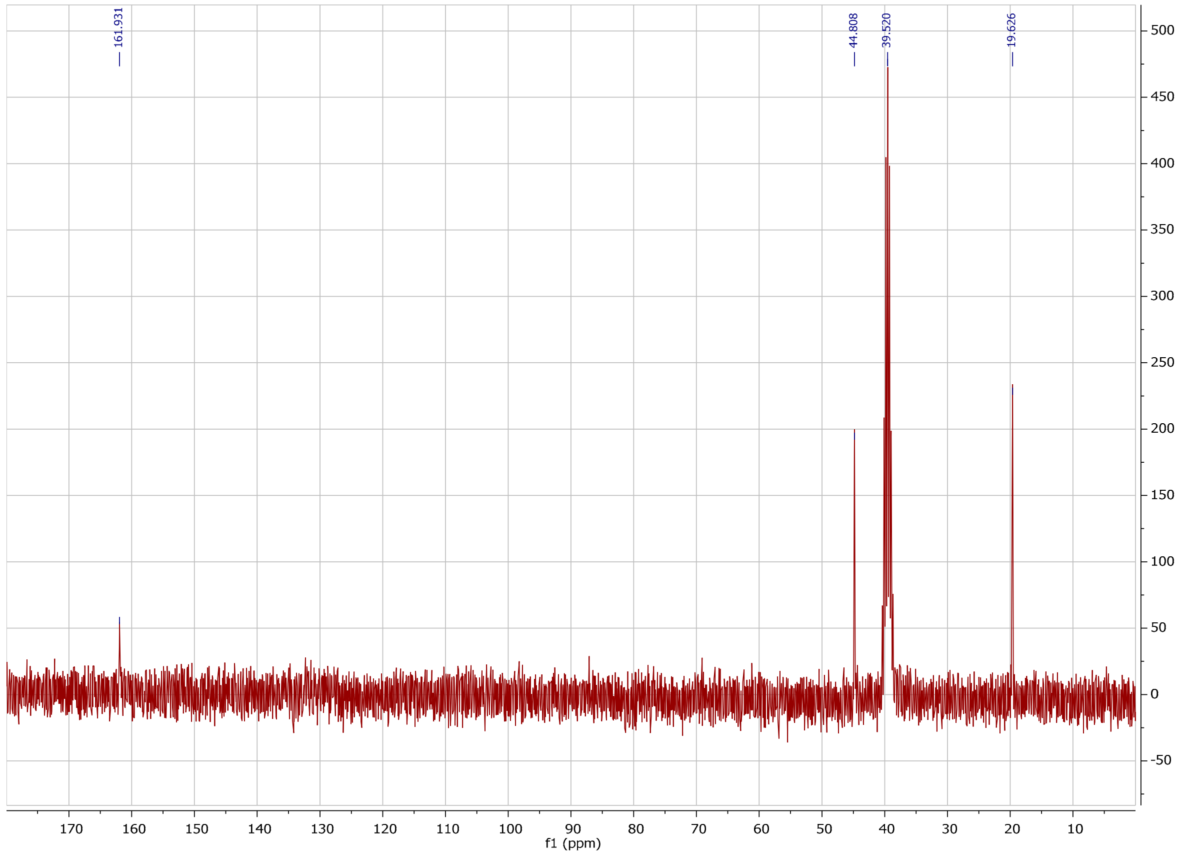

Methyl-(2*S*)-2-{[(benzyloxy)carbonyl]amino}butanoate **6b-L**:

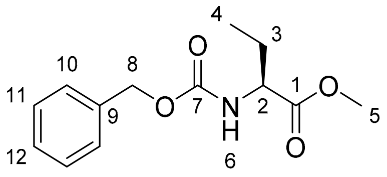

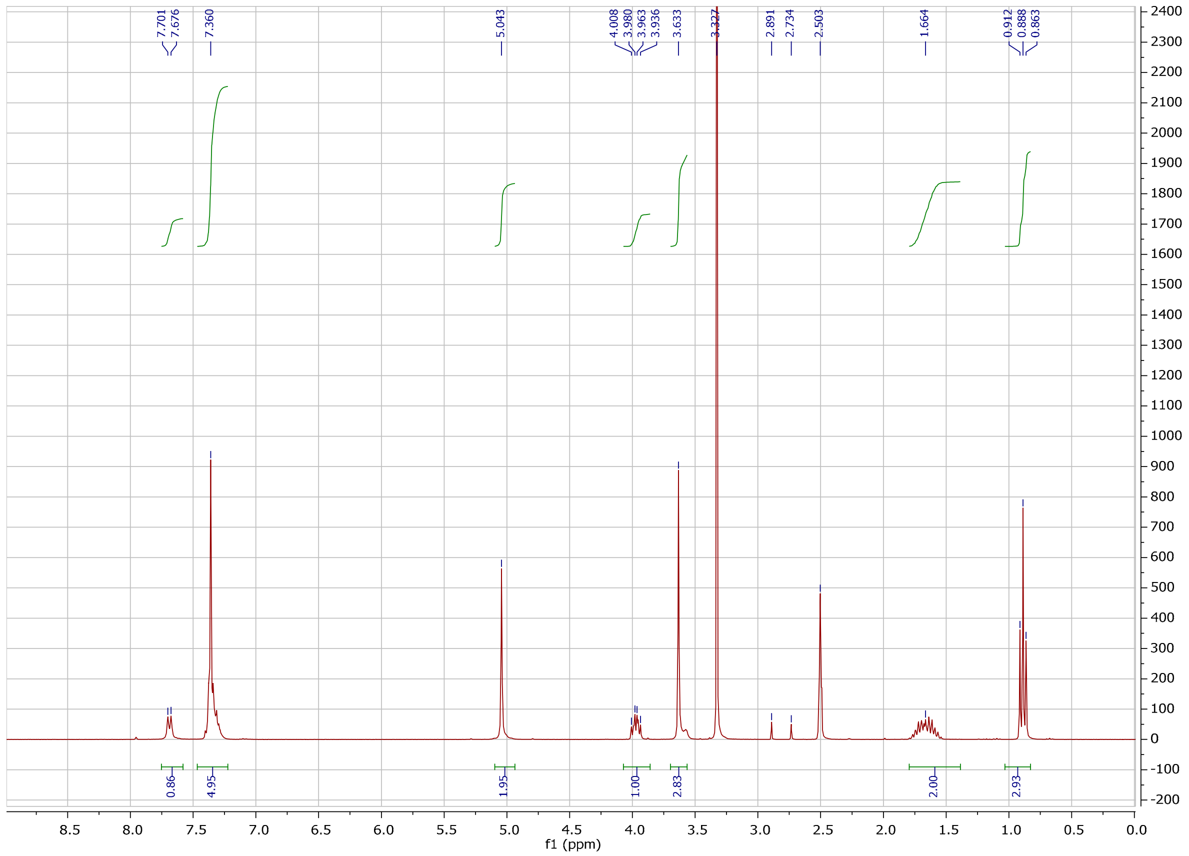
7.701
7.676
7.360
5.043
4.008
3.980
3.963
3.936
3.633
3.327
2.891
2.734
2.503
1.664
0.912
0.888
0.863
0.86
4.95
1.95
1.00
2.83
2.00
2.93
f1 (ppm)

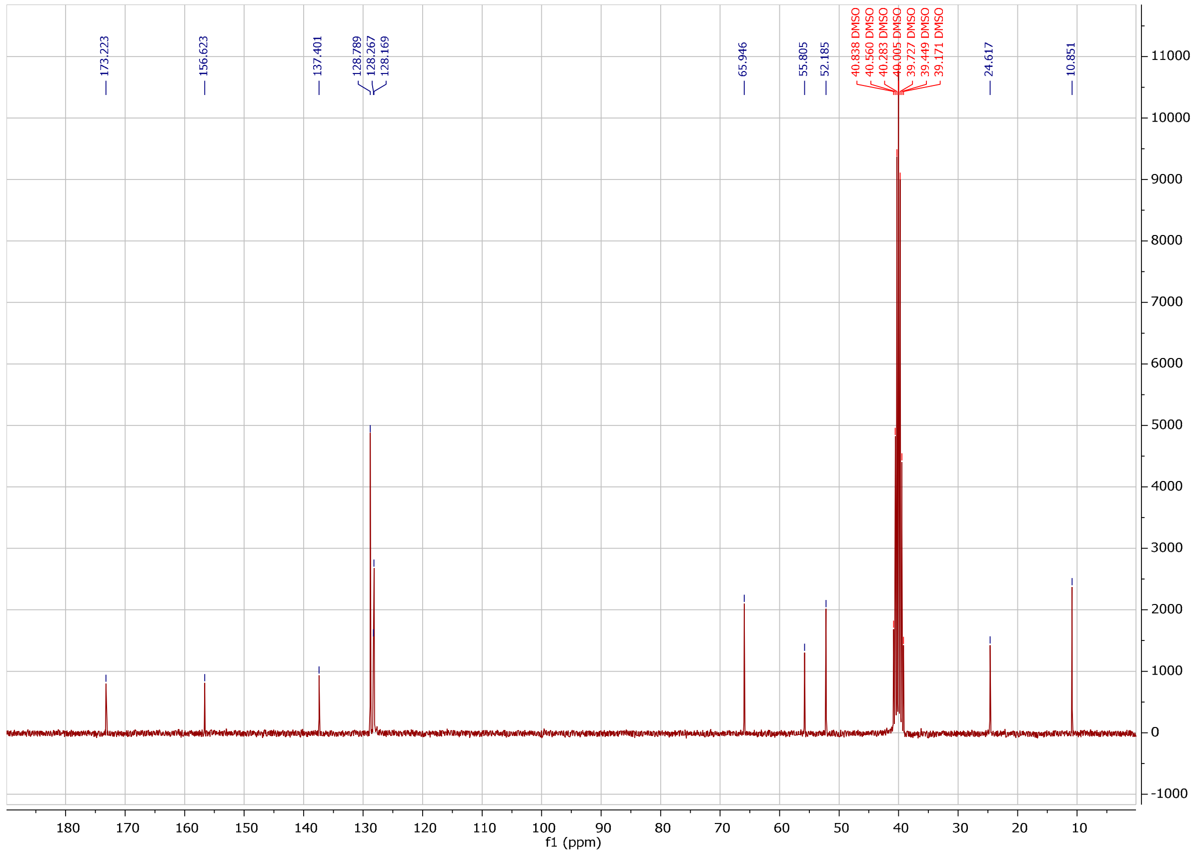
173.223
156.623
137.401
128.789
128.267
128.169
65.946
55.805
52.185
40.838 DMSO
40.560 DMSO
40.283 DMSO
40.005 DMSO
39.727 DMSO
39.449 DMSO
39.171 DMSO
24.617
10.851
f1 (ppm)

Benzyl-*N*-[(1*S*)-1-carbamoylpropyl]carbamate **7b-L**:

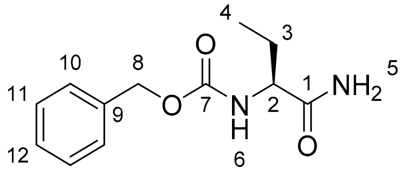

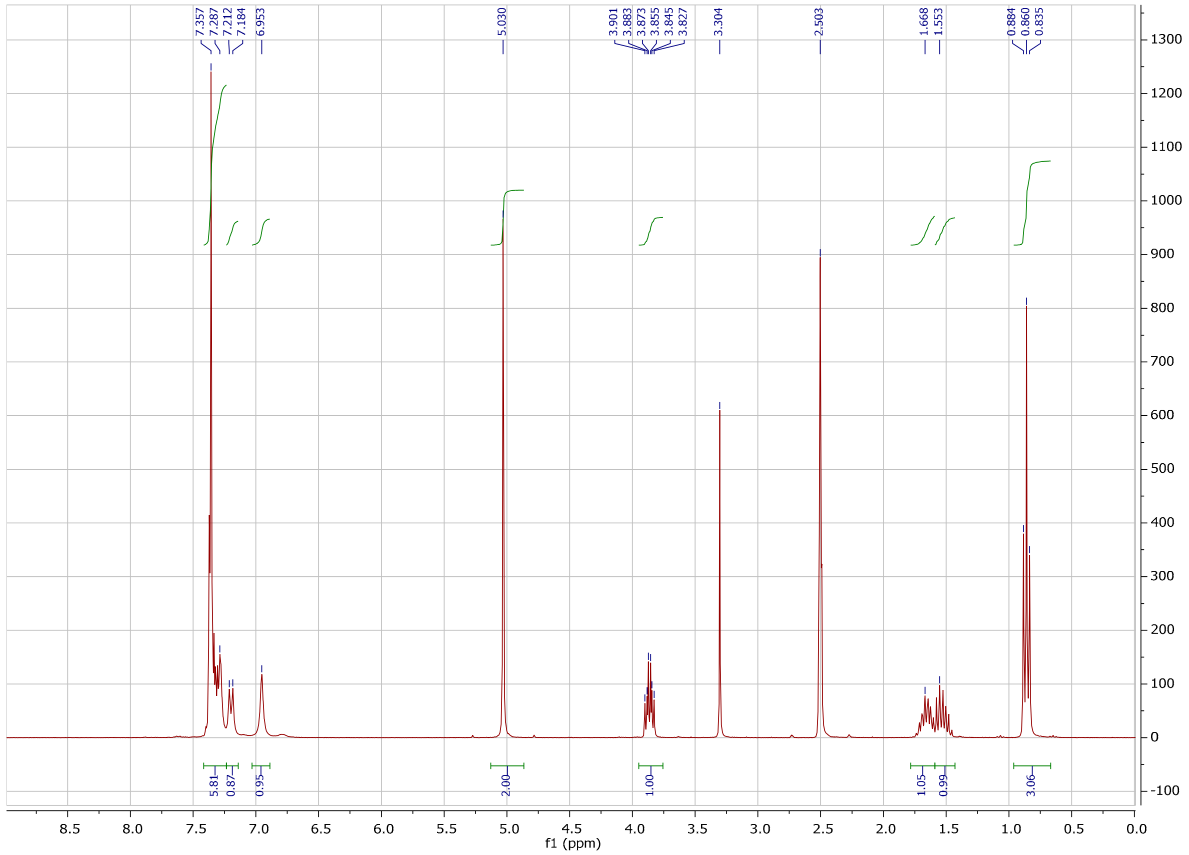

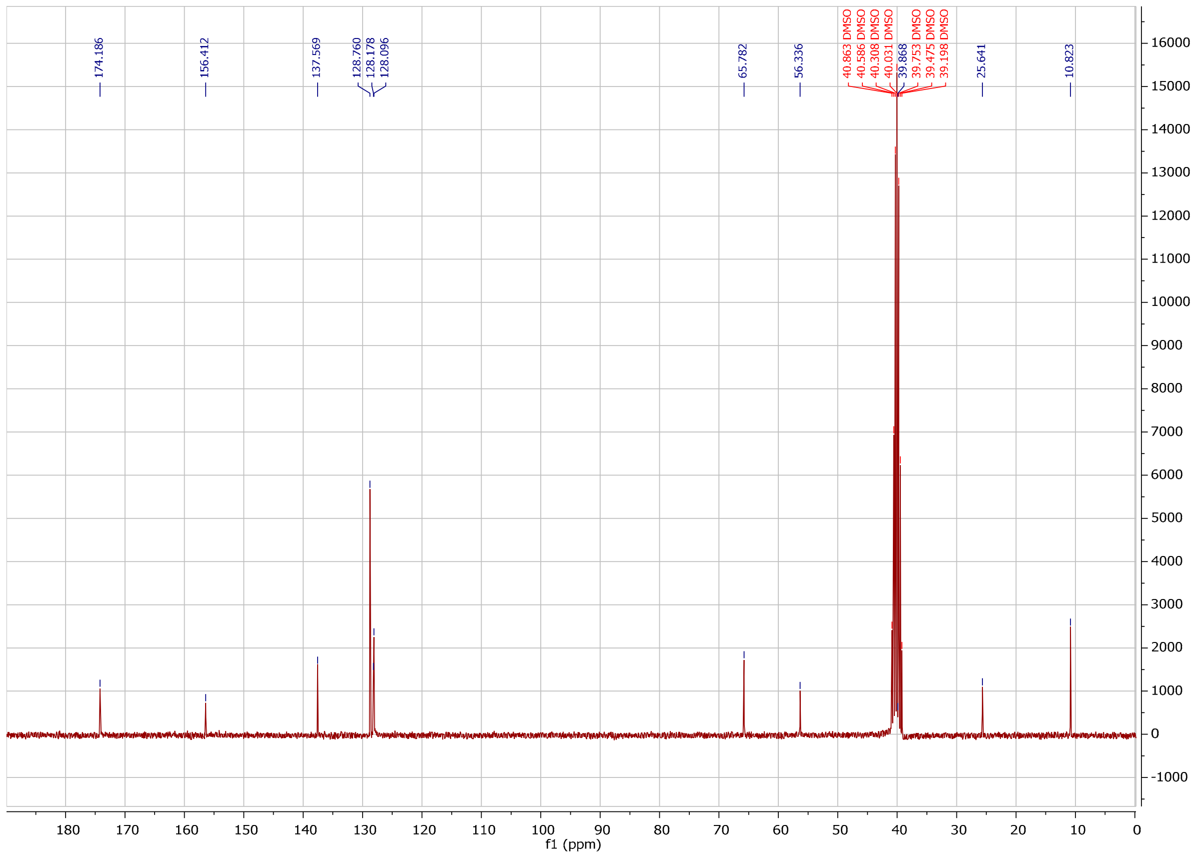

Benzyl-N-[(1*S*)-1-cyanopropyl]carbamate **8b-L**:

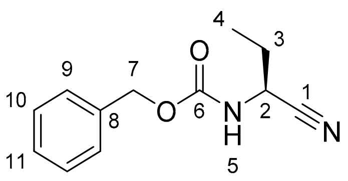

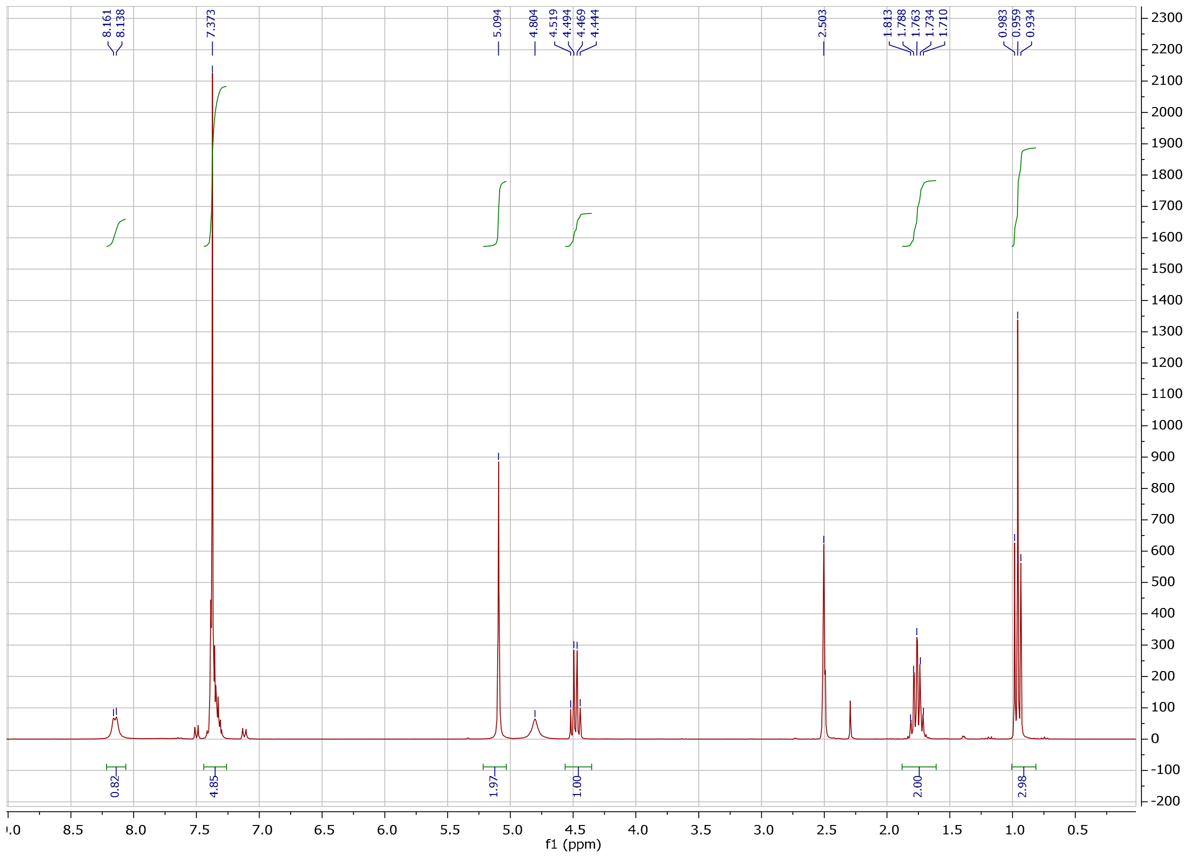
8.161
8.138
7.373
5.094
4.804
4.519
4.494
4.469
4.444
2.503
1.813
1.788
1.763
1.734
1.710
0.983
0.959
0.934
0.82
4.85
1.97
1.00
2.00
2.98
f1 (ppm)

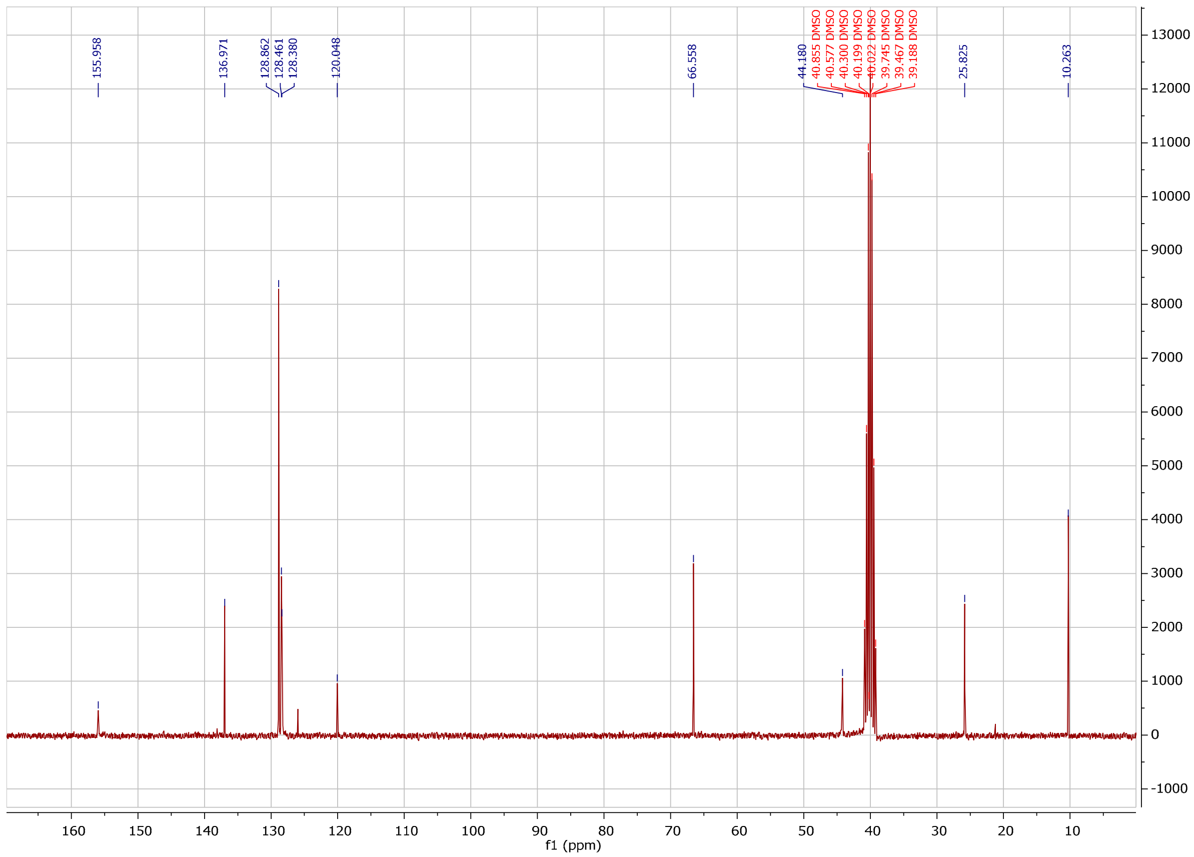
155.958
136.971
128.862
128.461
128.380
120.048
66.558
44.180
40.855 DMSO
40.577 DMSO
40.300 DMSO
40.199 DMSO
40.022 DMSO
39.745 DMSO
39.467 DMSO
39.188 DMSO
25.825
10.263
f1 (ppm)

Benzyl-*N*-[(1*S*)-1-(1*H*-1,2,3,4-tetrazol-5-yl)propyl]carbamate **9b-L**:

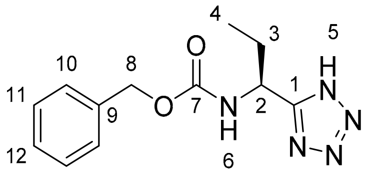

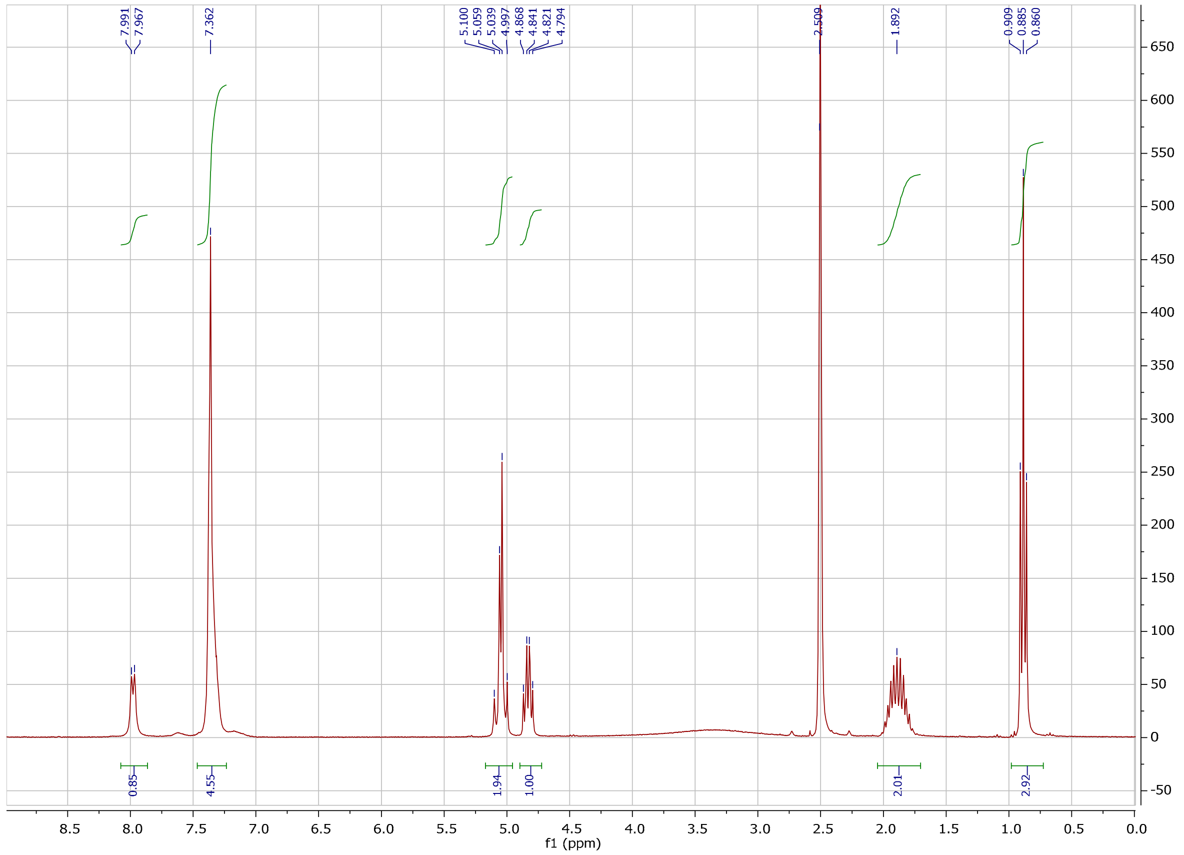

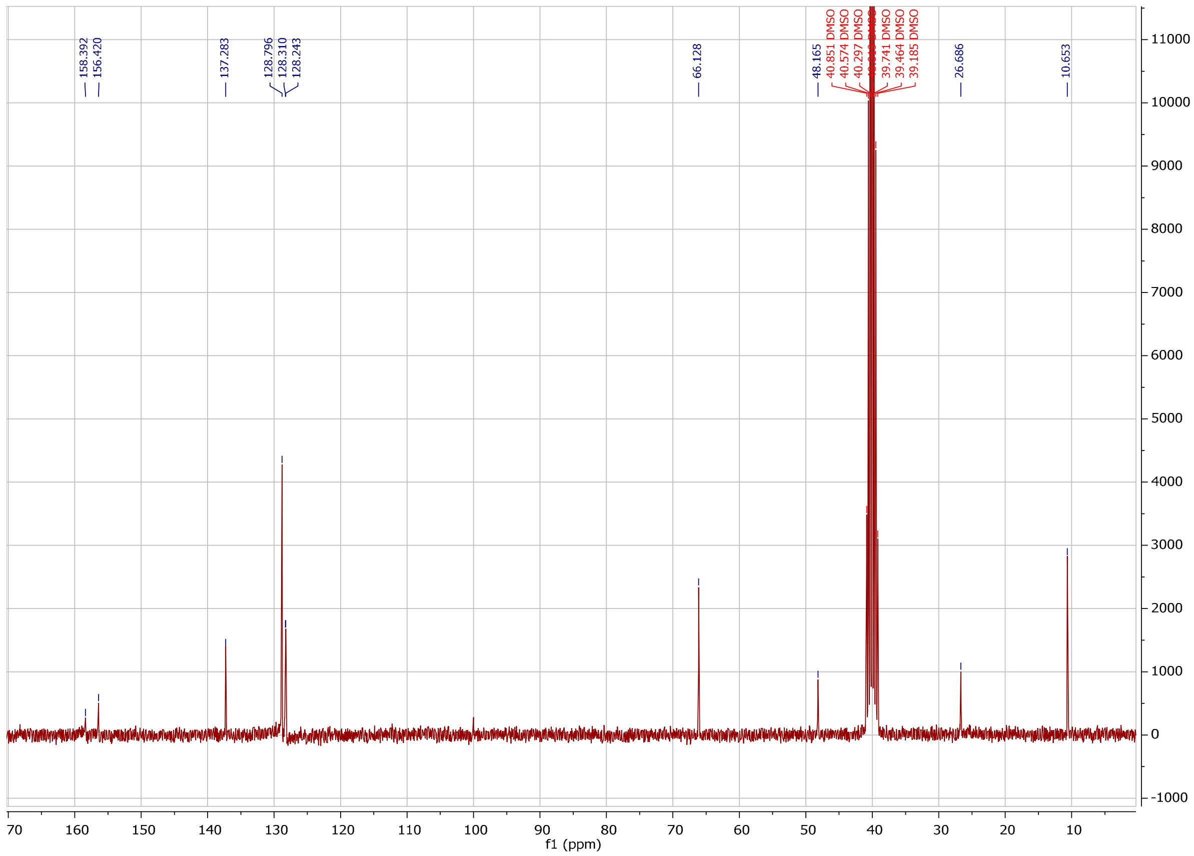

(1*S*)-1-(1*H*-1,2,3,4-tetrazol-5-yl)propane-1-amine **4b-L**:

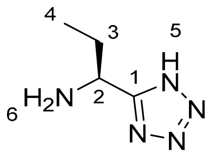

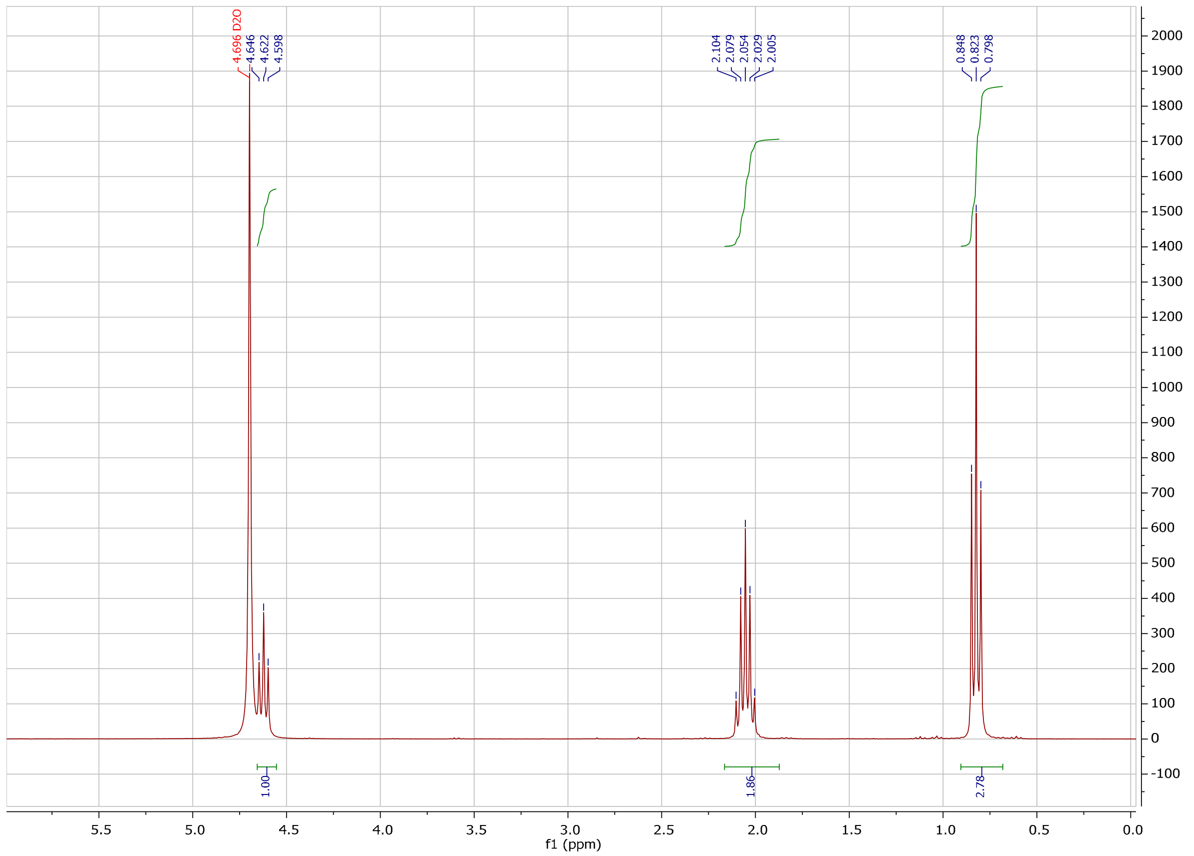
4.696 D2O
4.646
4.622
4.598
2.104
2.079
2.054
2.029
2.005
0.848
0.823
0.798
1.00
1.86
2.78
f1 (ppm)

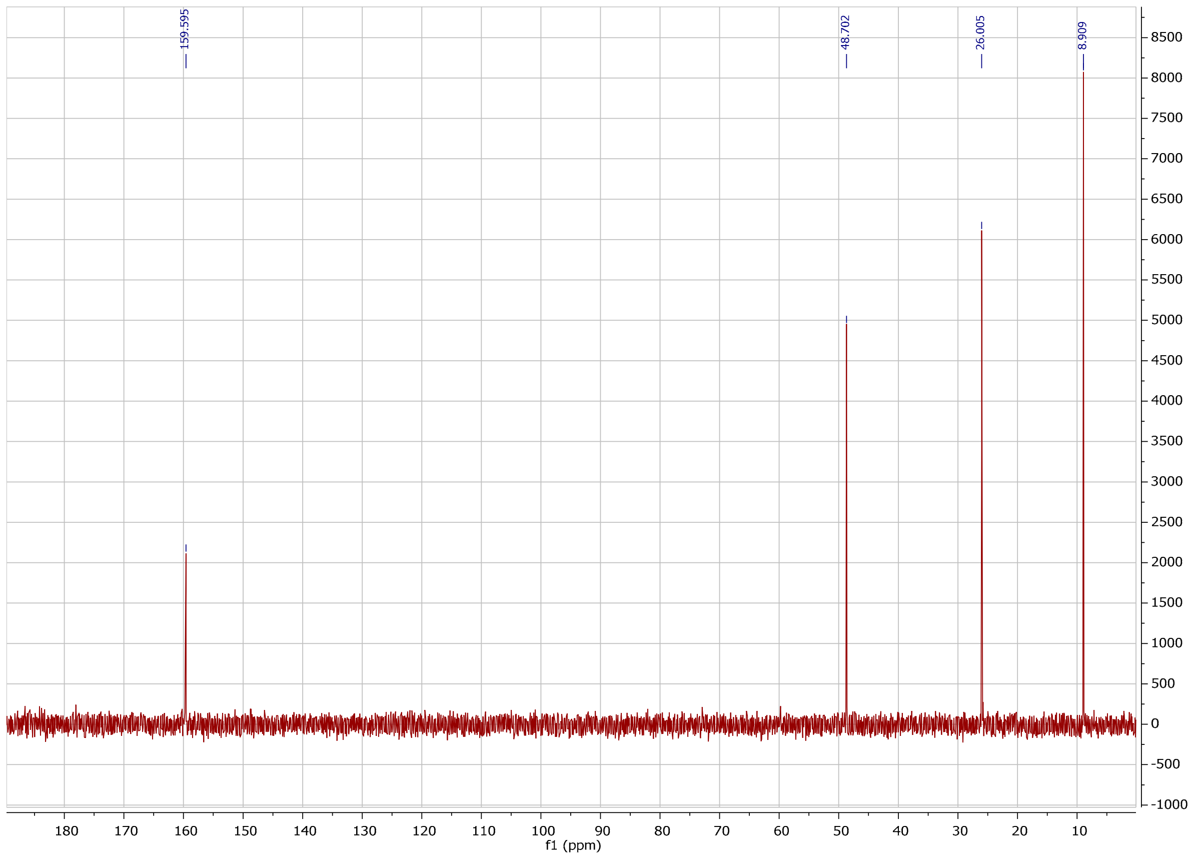
159.595
48.702
26.005
8.909
f1 (ppm)

9*H*-Fluoren-9-ylmethyl-*N*-[(1*S* or *R*)-1-(1*H*-1,2,3,4-tetrazol-5-yl)ethyl]carbamate **10a**:

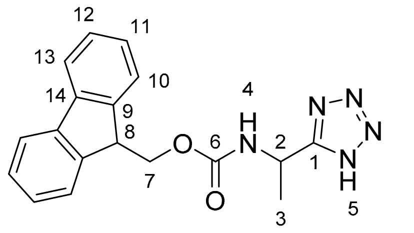

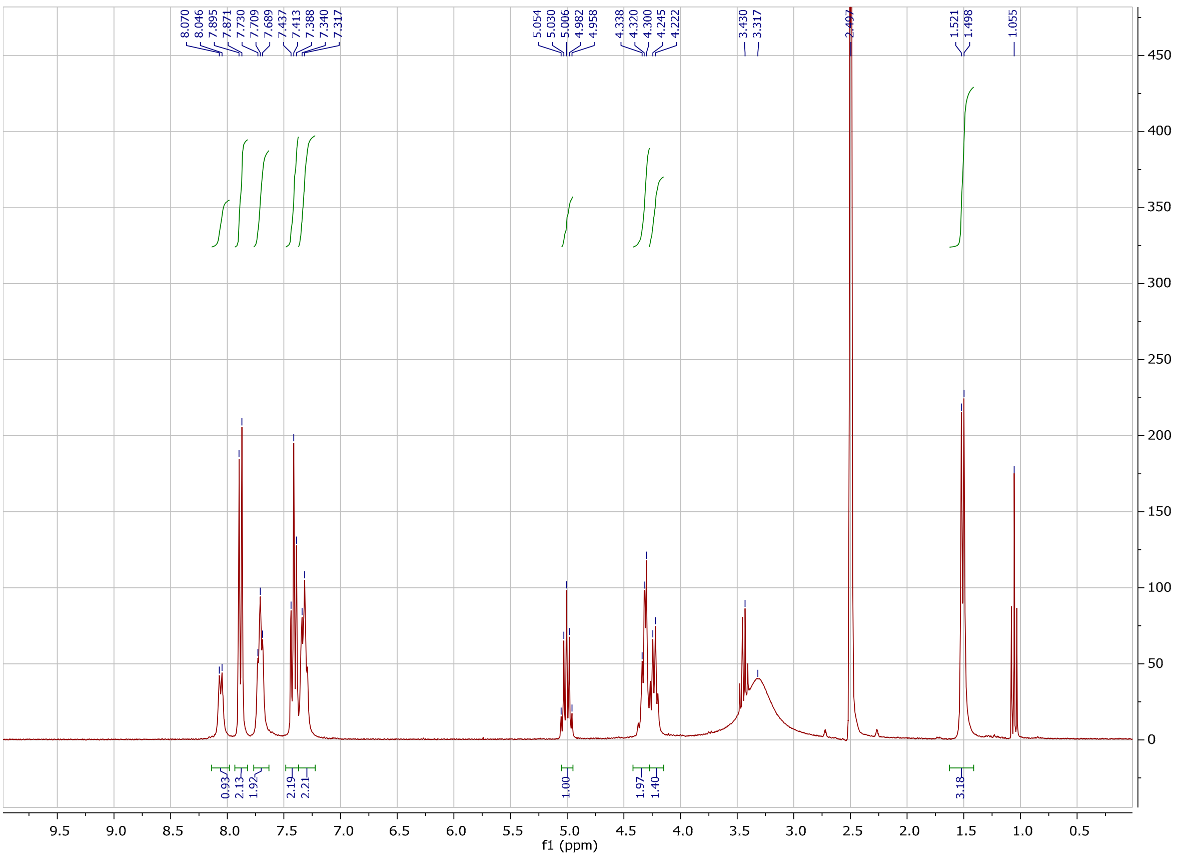

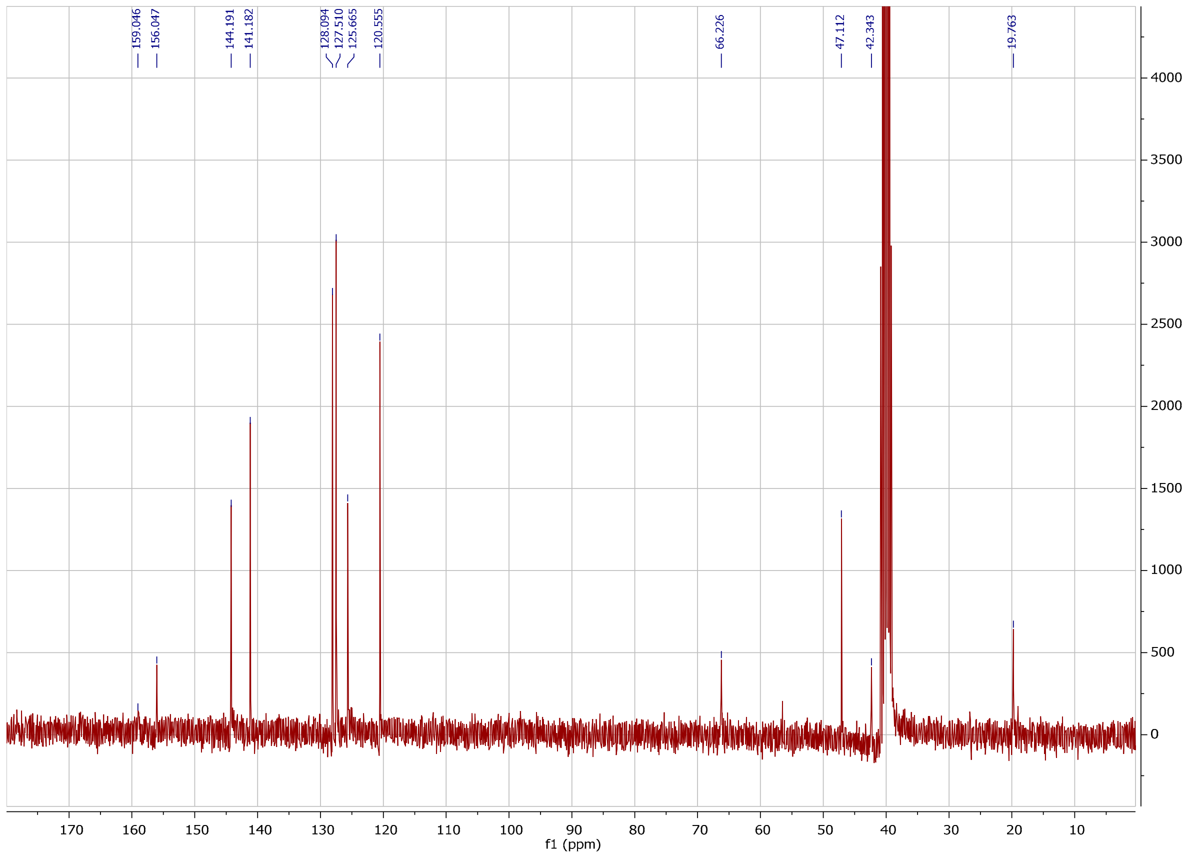

9*H*-Fluoren-9-ylmethyl-*N*-[(1*S*)-1-(1*H*-1,2,3,4-tetrazol-5-yl)propyl]carbamate **10b-L**

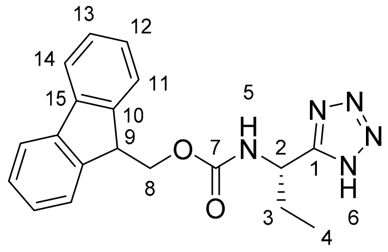

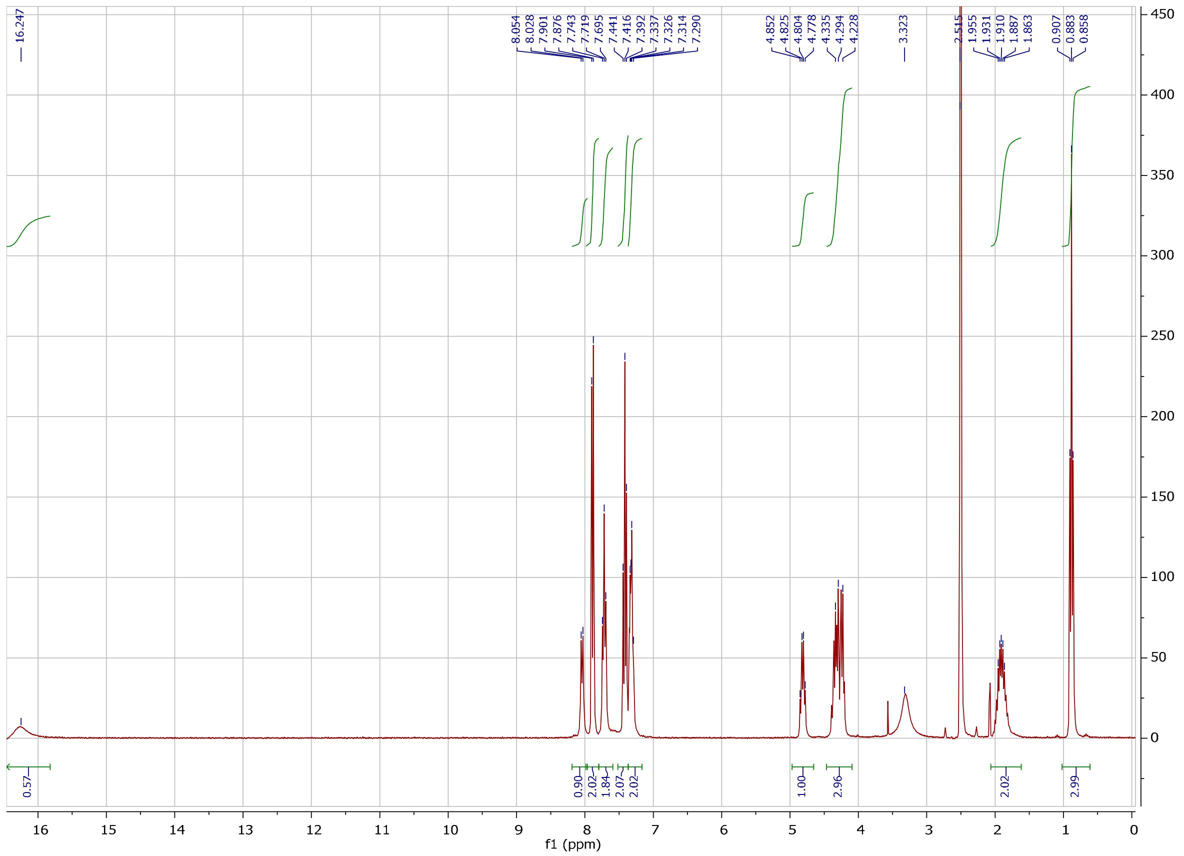

16.247
8.054
8.028
7.901
7.876
7.743
7.719
7.695
7.441
7.416
7.392
7.337
7.326
7.314
7.290
4.852
4.825
4.804
4.778
4.335
4.294
4.228
3.323
2.515
1.955
1.931
1.910
1.887
1.863
0.907
0.883
0.858
0.57
0.90
2.02
1.84
2.07
2.02
1.00
2.96
2.02
2.99
f1 (ppm)

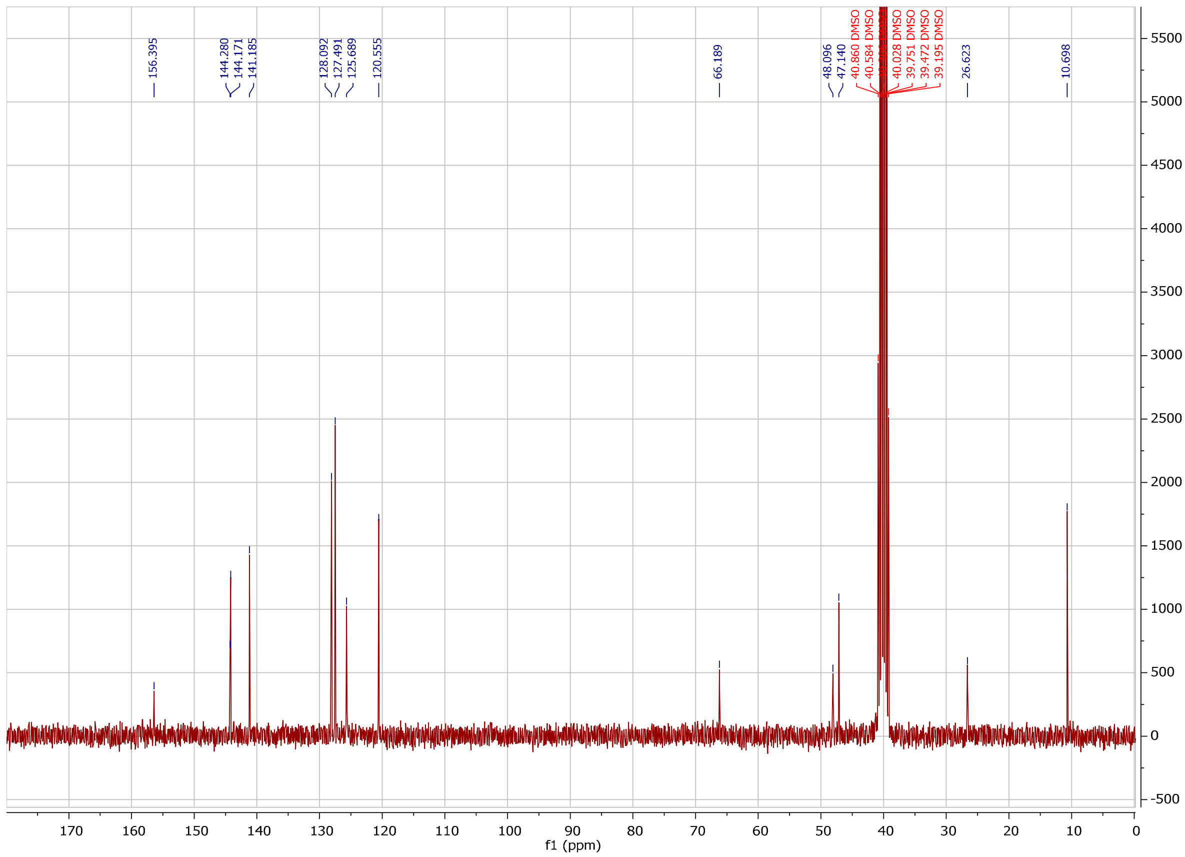

156.395
144.280
144.171
141.185
128.092
127.491
125.689
120.555
66.189
48.096
47.140
40.860 DMSO
40.584 DMSO
40.028 DMSO
39.751 DMSO
39.472 DMSO
39.195 DMSO
26.623
10.698
f1 (ppm)

Benzyl-*N*-({[(1*S*)-1-(1*H*-1,2,3,4-tetrazol-5-yl)ethyl]carbamoyl}methyl)carbamate **13g-L**:

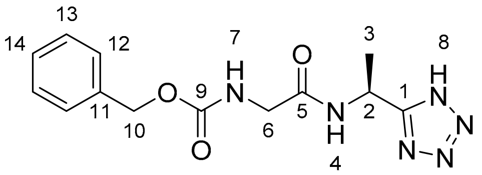

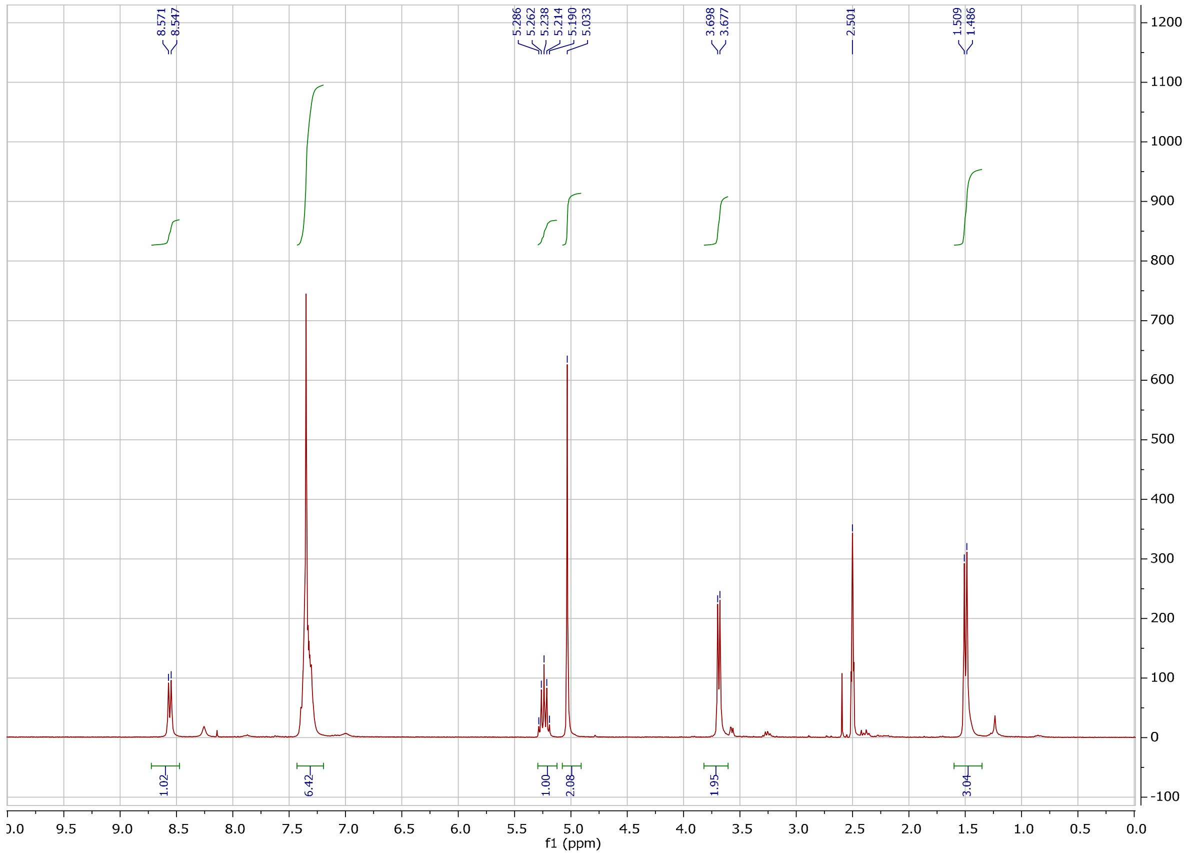

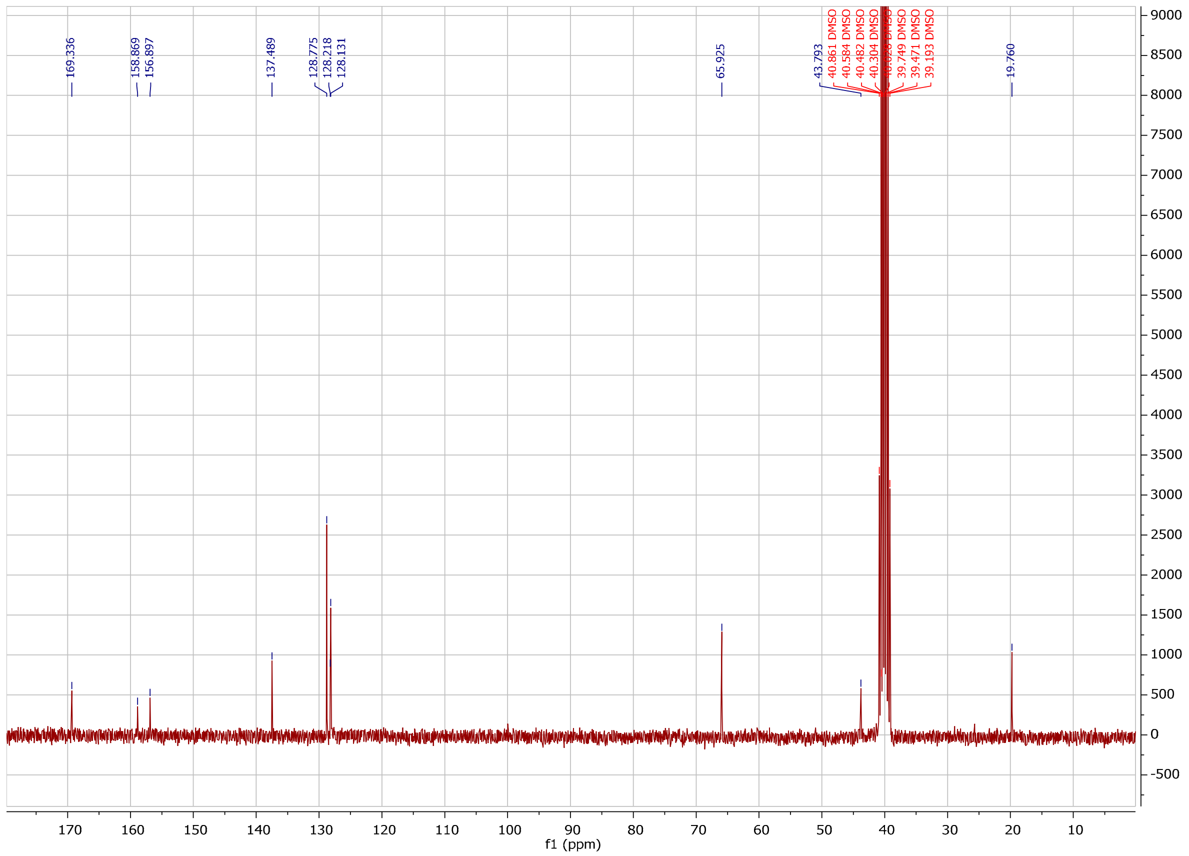

169.336
158.869
156.897
137.489
128.775
128.218
128.131
65.925
43.793
40.861 DMSO
40.584 DMSO
40.482 DMSO
40.304 DMSO
39.749 DMSO
39.471 DMSO
39.193 DMSO
19.760
f1 (ppm)

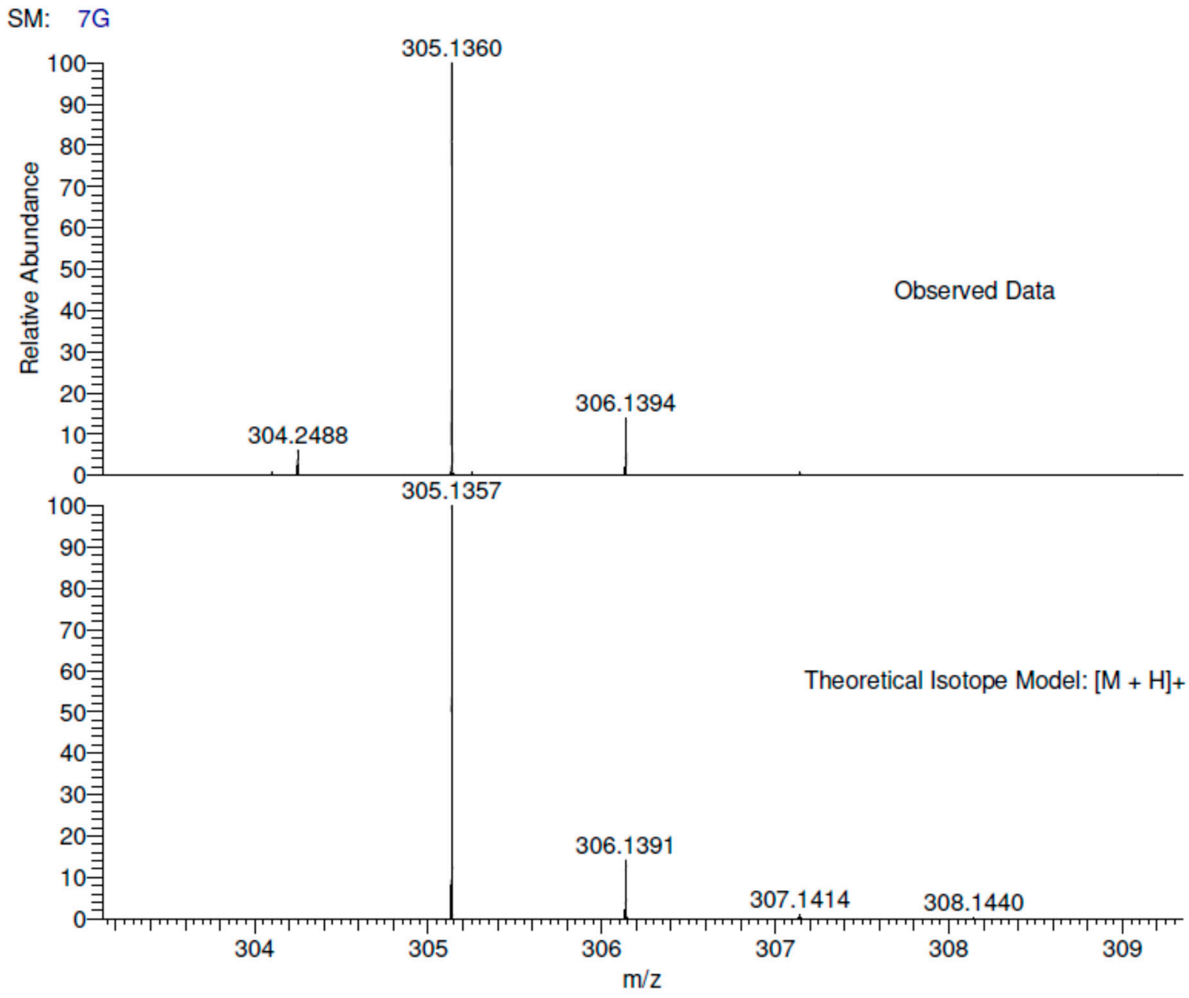

SM: 7G
305.1360
Relative Abundance
Observed Data
304.2488
306.1394
305.1357
Theoretical Isotope Model: [M + H]+
306.1391
307.1414
308.1440
304
305
306
307
308
309
m/z

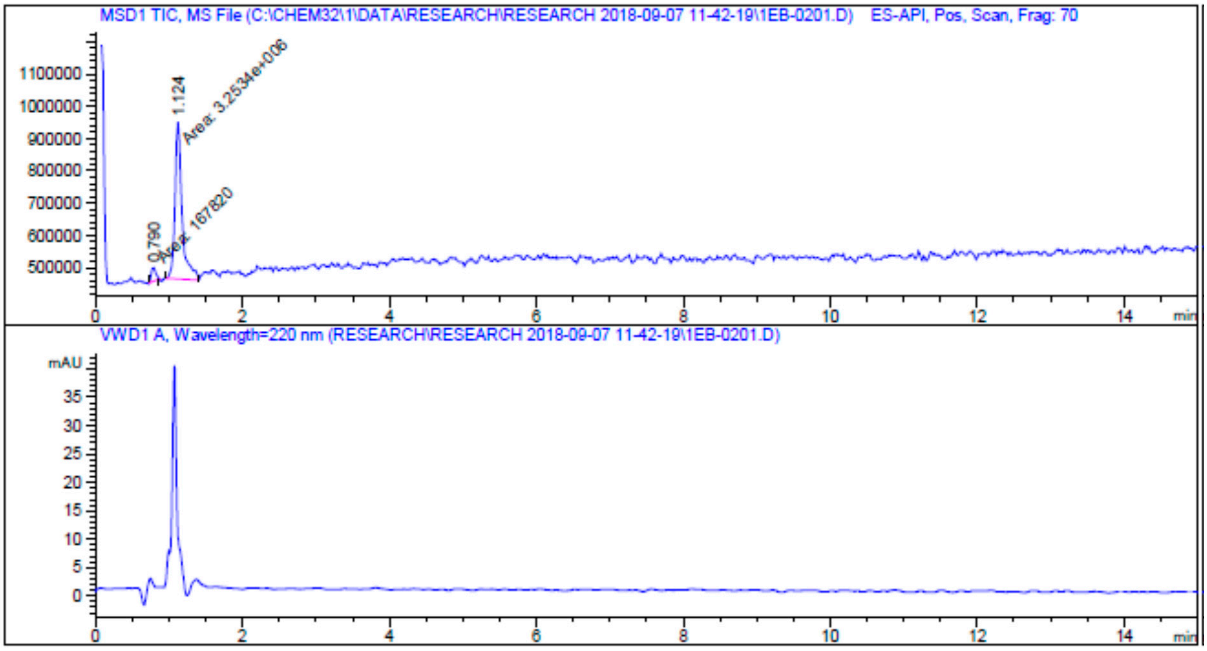

Area Percent Report

Sorted By : Signal
Multiplier: : 1.0000
Dilution: : 1.0000
Use Multiplier & Dilution Factor with ISTDs

Signal 1: MSD1 TIC, MS File

Peak #	RetTime [min]	Type	Width [min]	Area	Height	Area %
1	0.790	MM	0.0623	1.67820e5	4.49148e4	4.9053
2	1.124	MM	0.1104	3.25340e6	4.91366e5	95.0947

Totals : 3.42122e6 5.36281e5

2-Amino-*N*-[(1*S*)-1-(1*H*-1,2,3,4-tetrazol-5-yl)ethyl]acetamide **14g-L**:

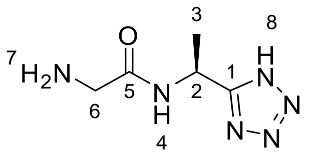

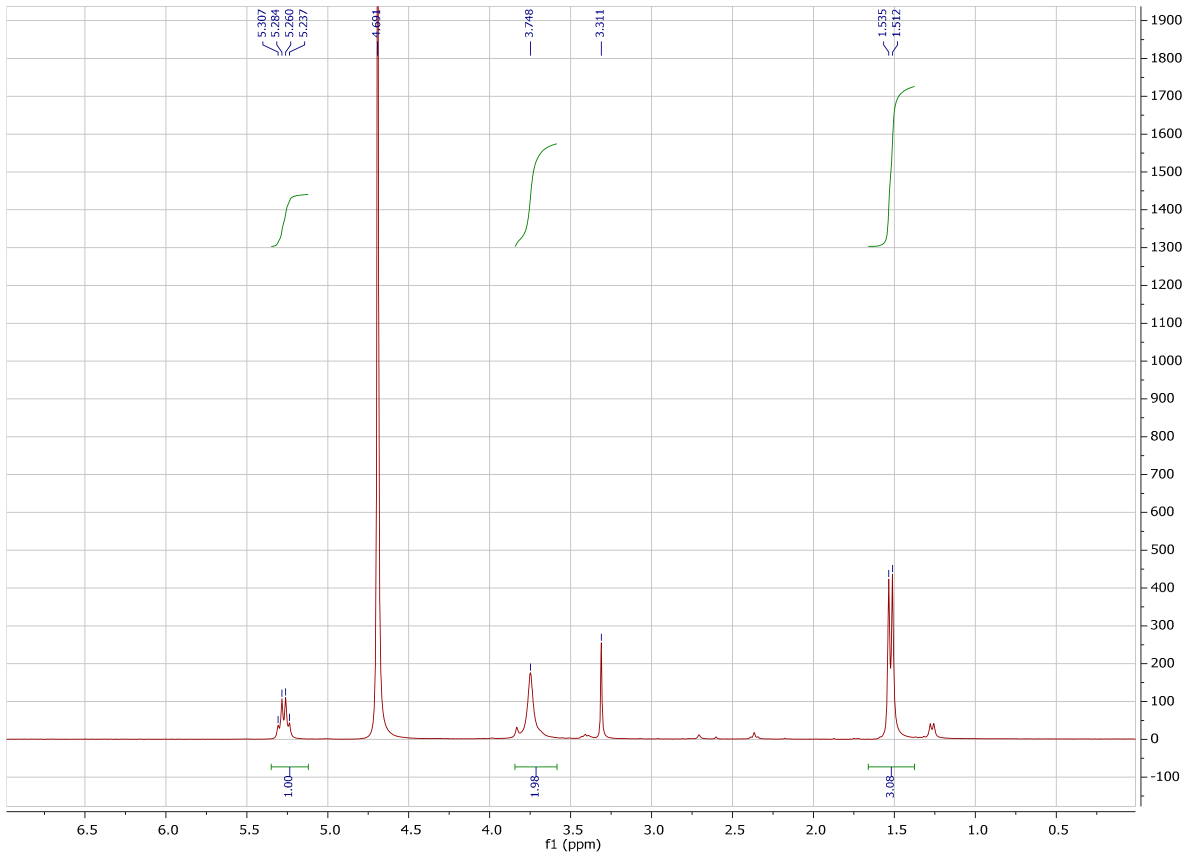
5.307
5.284
5.260
5.237
4.691
3.748
3.311
1.535
1.512
1.00
1.98
3.08
f1 (ppm)

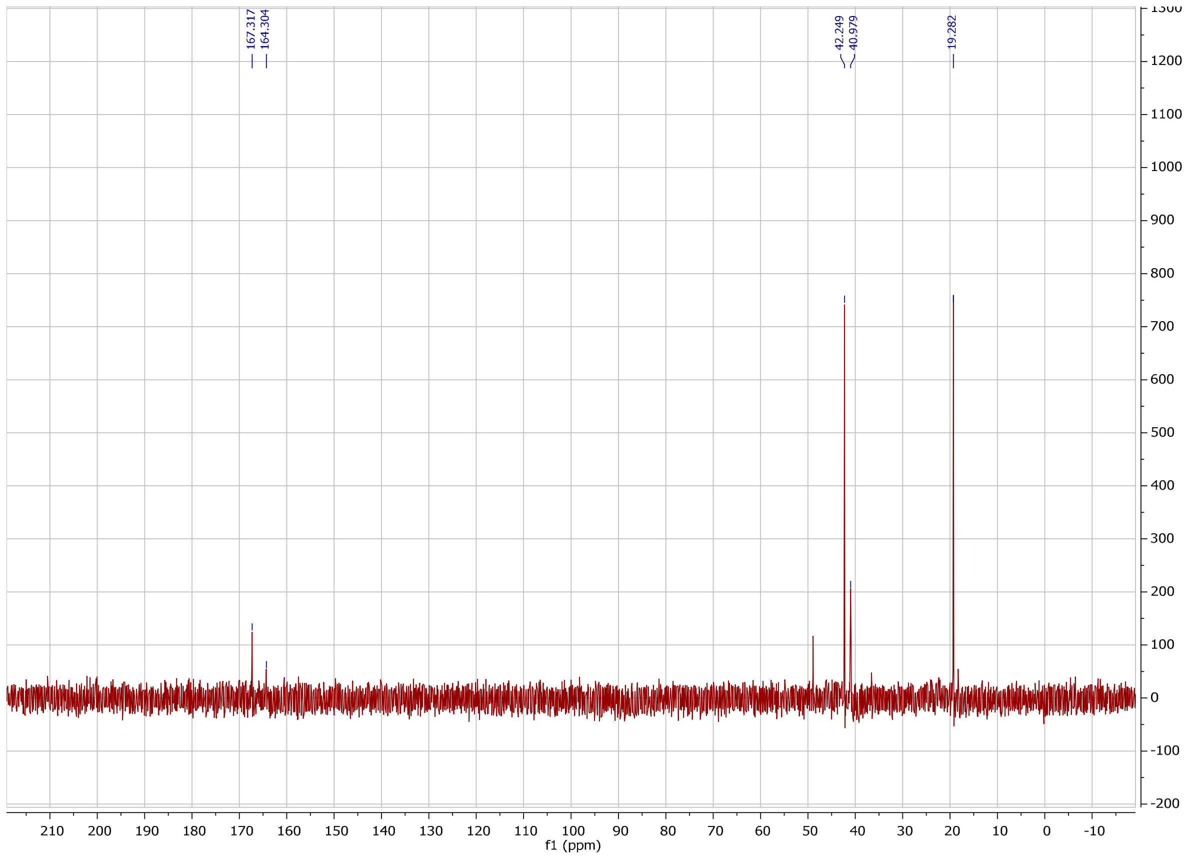
167.317
164.304
42.249
40.979
19.282
f1 (ppm)

SM: 7G

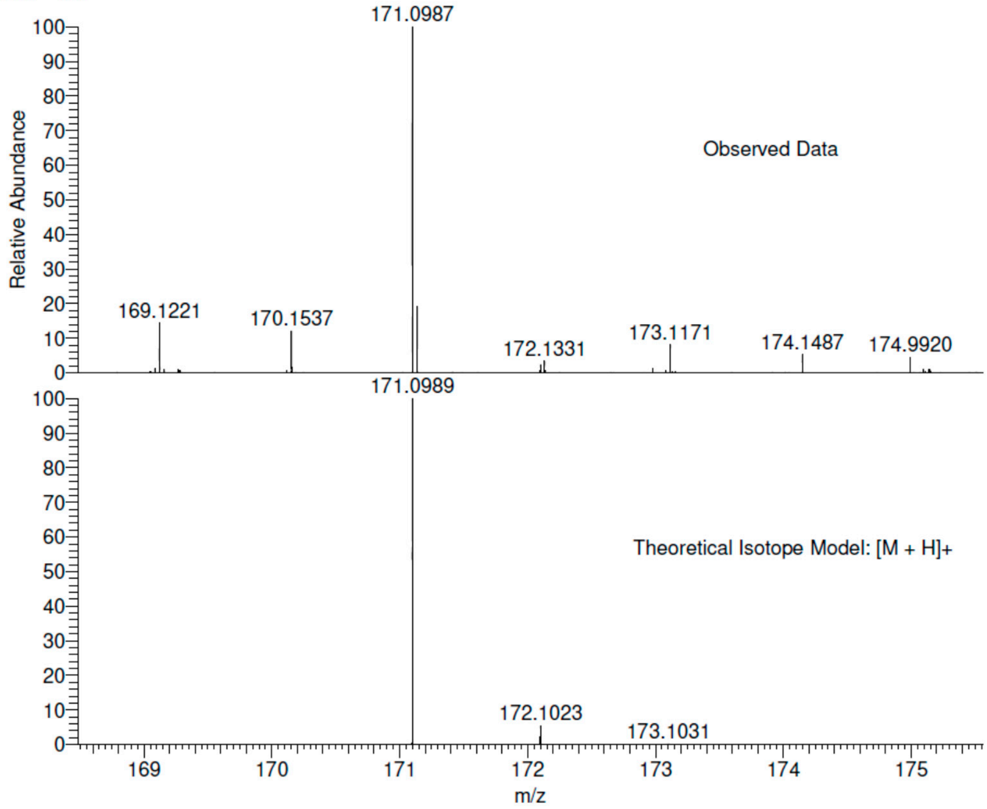

171.0987
Observed Data
Relative Abundance
169.1221
170.1537
172.1331
173.1171
174.1487
174.9920
171.0989
Theoretical Isotope Model: [M + H]+
172.1023
173.1031
m/z

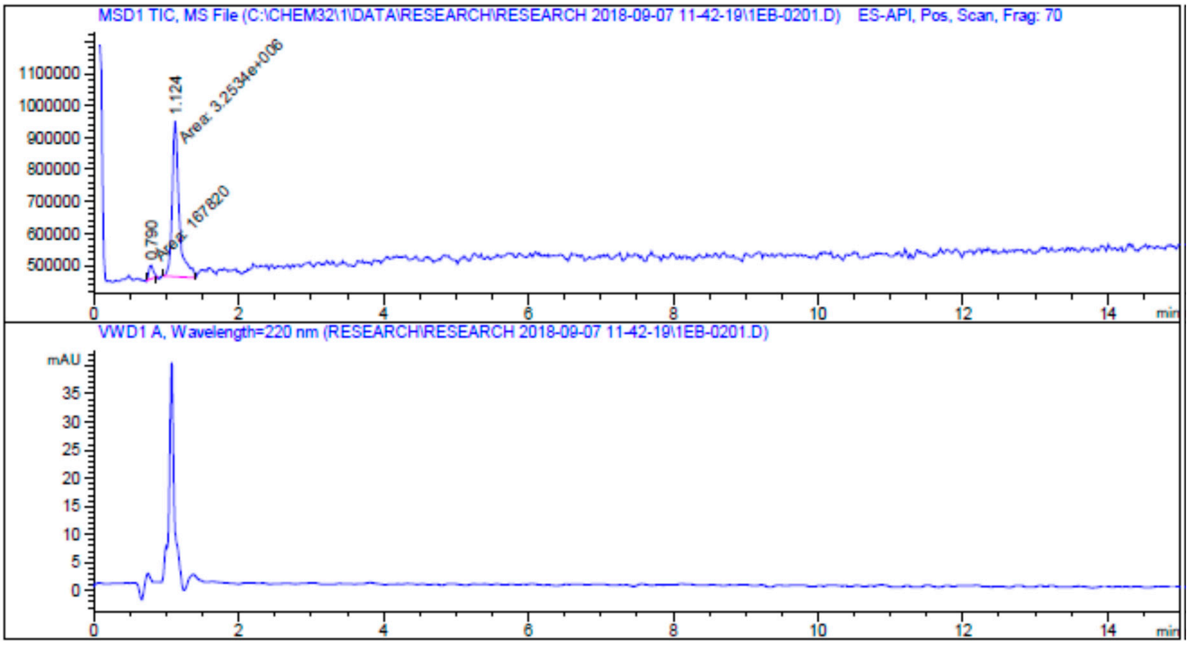

===

Area Percent Report

===

Sorted By : Signal
Multiplier: : 1.0000
Dilution: : 1.0000
Use Multiplier & Dilution Factor with ISTDs

Signal 1: MSD1 TIC, MS File

Peak #	RetTime [min]	Type	Width [min]	Area	Height	Area %
1	0.790	MM	0.0623	1.67820e5	4.49148e4	4.9053
2	1.124	MM	0.1104	3.25340e6	4.91366e5	95.0947

Totals : 3.42122e6 5.36281e5

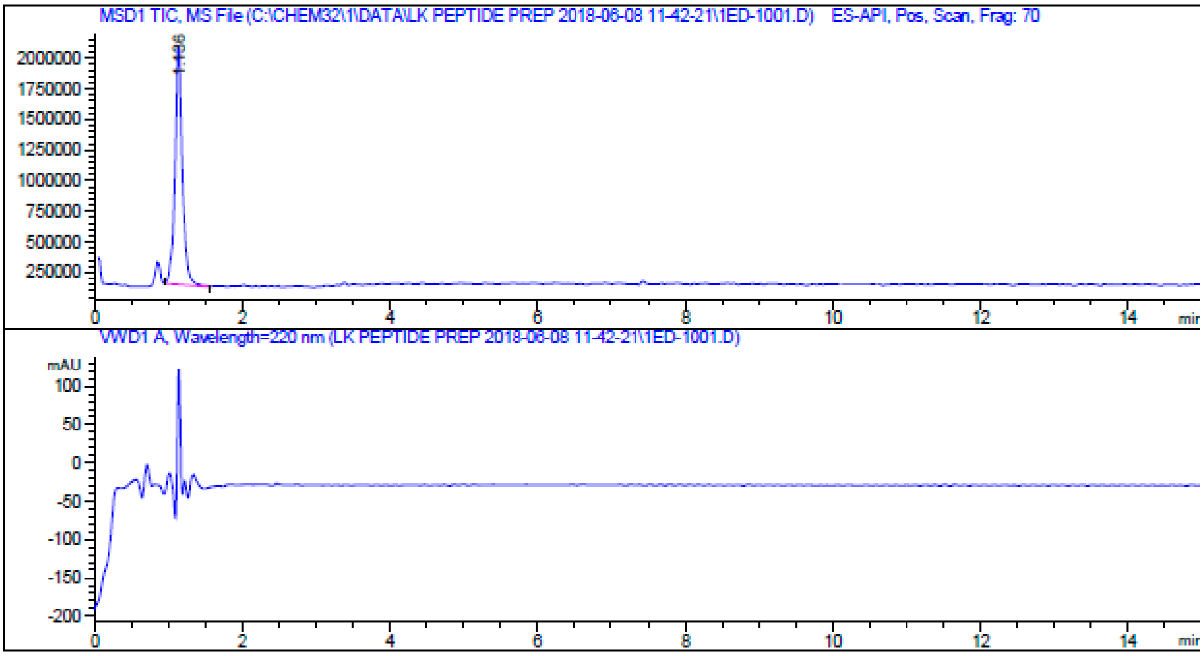


```
=====================================================================
                     Area Percent Report
=====================================================================

Sorted By             :      Signal
Multiplier:                  :      1.0000
Dilution:                    :      1.0000
Use Multiplier & Dilution Factor with ISTDs


Signal 1: MSD1 TIC, MS File

Peak RetTime Type  Width      Area       Height      Area
  #   [min]        [min]                              %
----|-------|----|-------|----------|----------|--------|
   1   1.136 BB    0.1009 1.34913e7  1.99147e6  100.0000

Totals :                  1.34913e7  1.99147e6
```

Benzyl-*N*-[(1*S*)-1-{[(1*S*)-1-(1*H*-1,2,3,4-tetrazol-5-yl)ethyl]carbamoyl}ethyl]carbamate **13a-LL**:

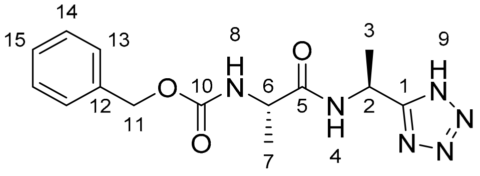

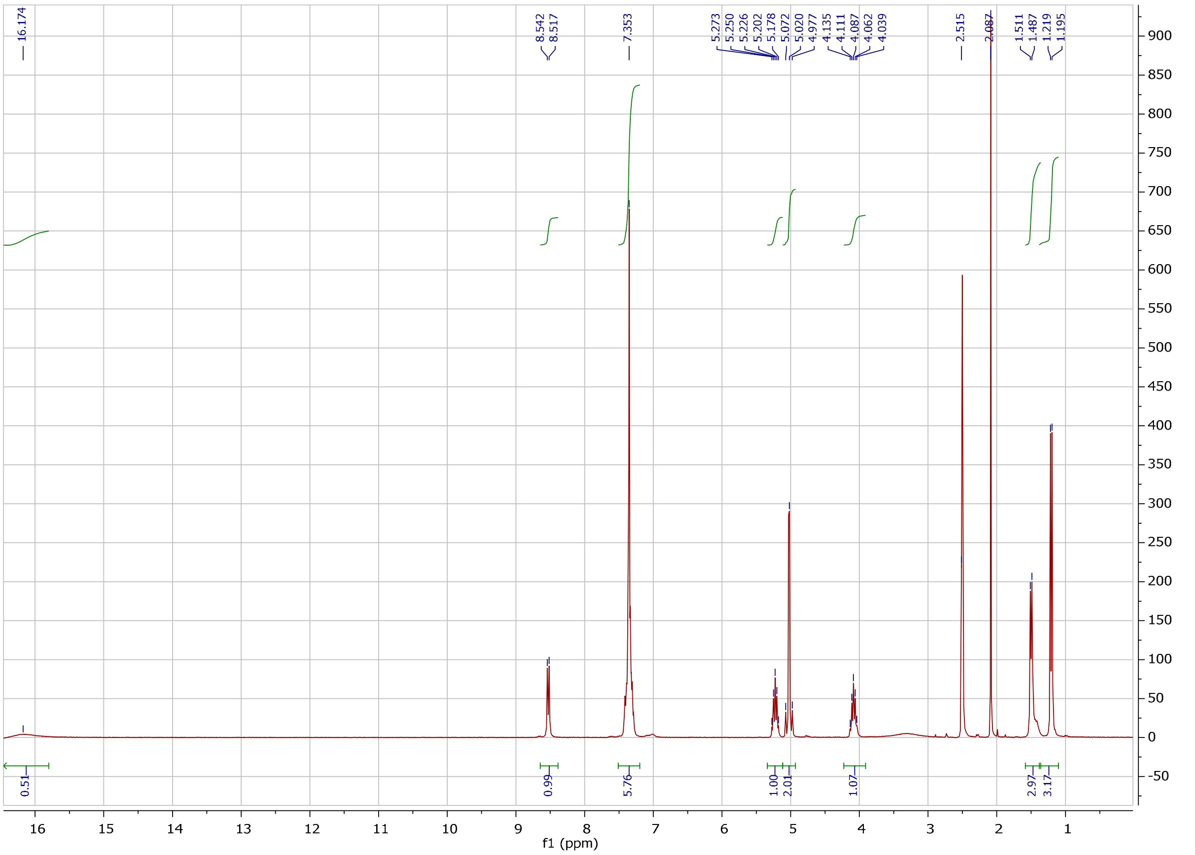
16.174
8.542
8.517
7.353
5.273
5.250
5.226
5.202
5.178
5.072
5.020
4.977
4.135
4.111
4.087
4.062
4.039
2.515
2.087
1.511
1.487
1.219
1.195
0.51
0.99
5.76
1.00
2.01
1.07
2.97
3.17
f1 (ppm)

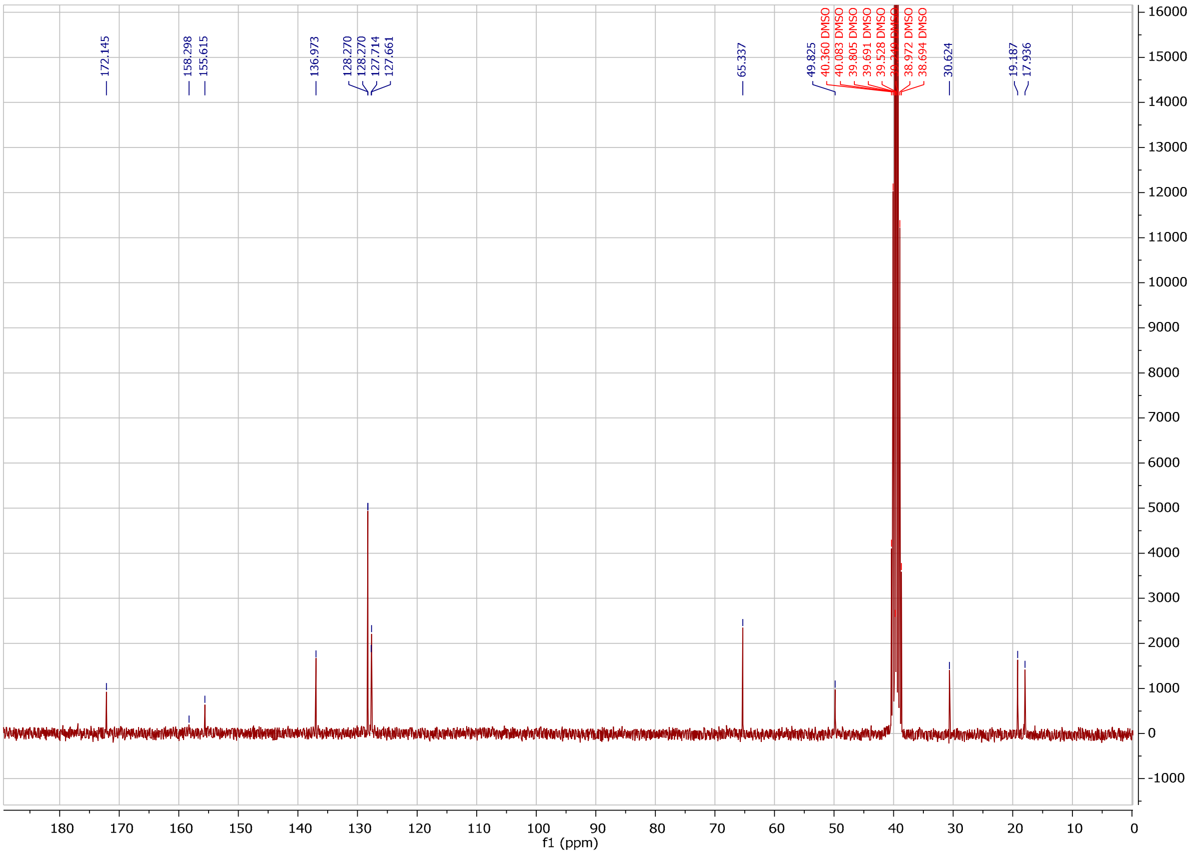
172.145
158.298
155.615
136.973
128.270
128.270
127.714
127.661
65.337
49.825
40.360 DMSO
40.083 DMSO
39.805 DMSO
39.691 DMSO
39.528 DMSO
38.972 DMSO
38.694 DMSO
30.624
19.187
17.936
f1 (ppm)

(2*S*)-2-amino-*N*-[(1*S*)-1-(1*H*-1,2,3,4-tetrazol-5-yl)ethyl]propanamide **14a-LL**:

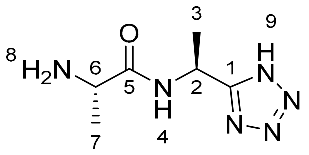

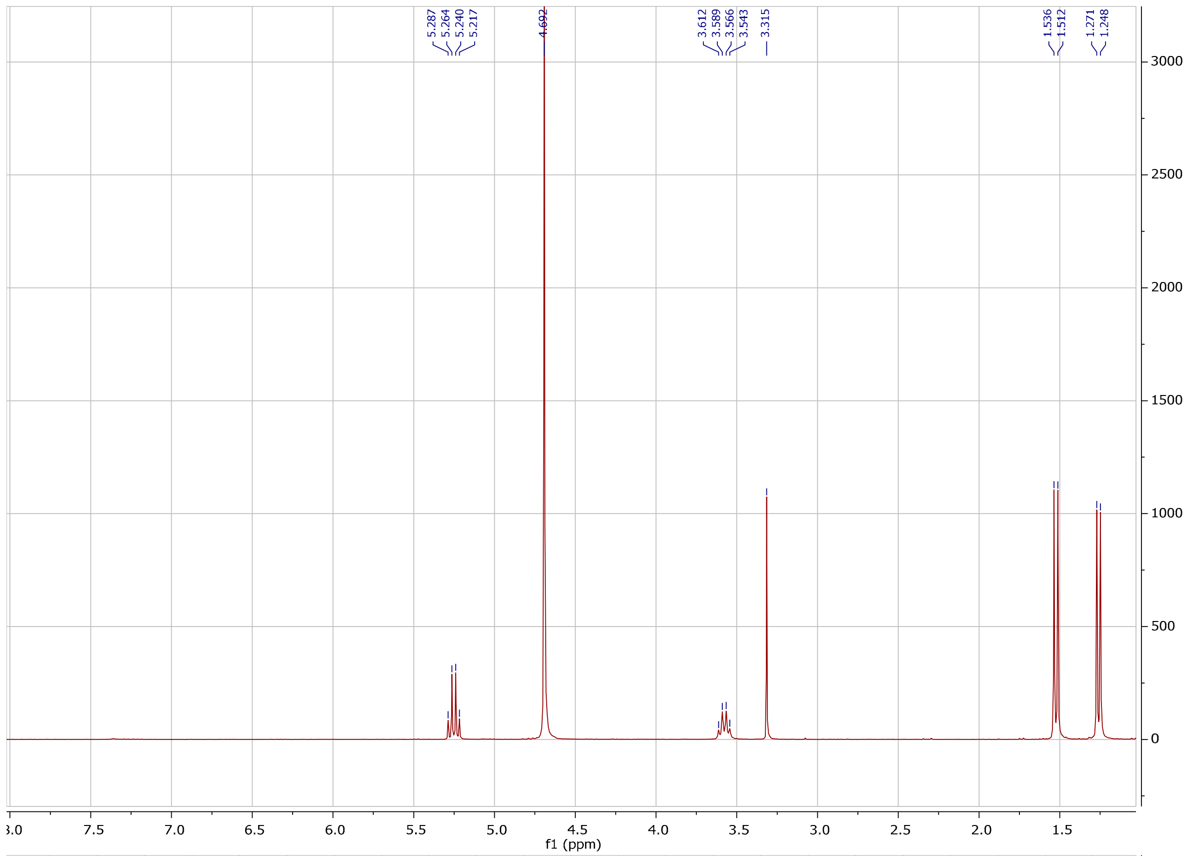
5.287
5.264
5.240
5.217
4.692
3.612
3.589
3.566
3.543
3.315
1.536
1.512
1.271
1.248
3000
2500
2000
1500
1000
500
0
7.5
7.0
6.5
6.0
5.5
5.0
4.5
4.0
3.5
3.0
2.5
2.0
1.5
f1 (ppm)

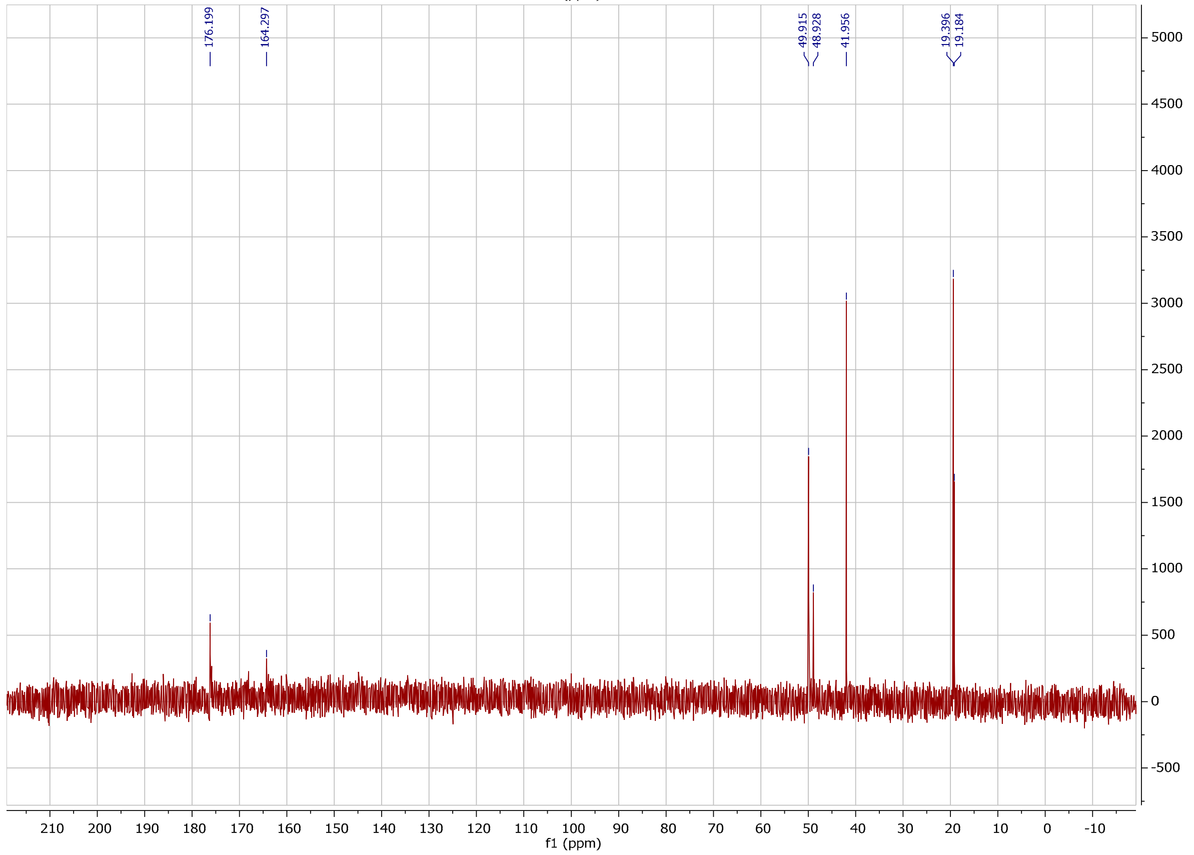
176.199
164.297
49.915
48.928
41.956
19.396
19.184
5000
4500
4000
3500
3000
2500
2000
1500
1000
500
0
-500
210
200
190
180
170
160
150
140
130
120
110
100
90
80
70
60
50
40
30
20
10
0
-10
f1 (ppm)

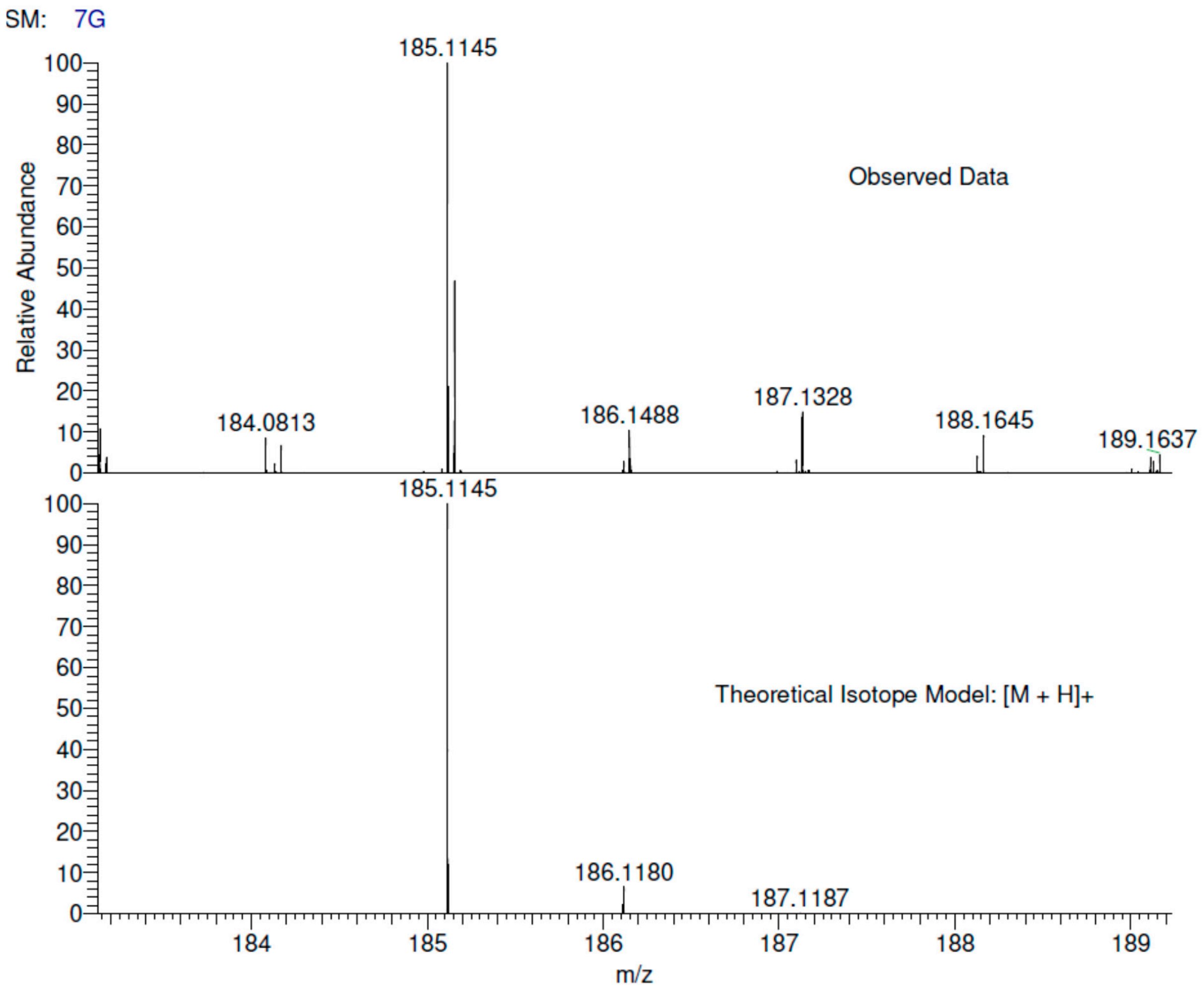
SM: 7G
Observed Data
185.1145
184.0813
186.1488
187.1328
188.1645
189.1637
Theoretical Isotope Model: [M + H]+
185.1145
186.1180
187.1187
Relative Abundance
m/z
184
185
186
187
188
189

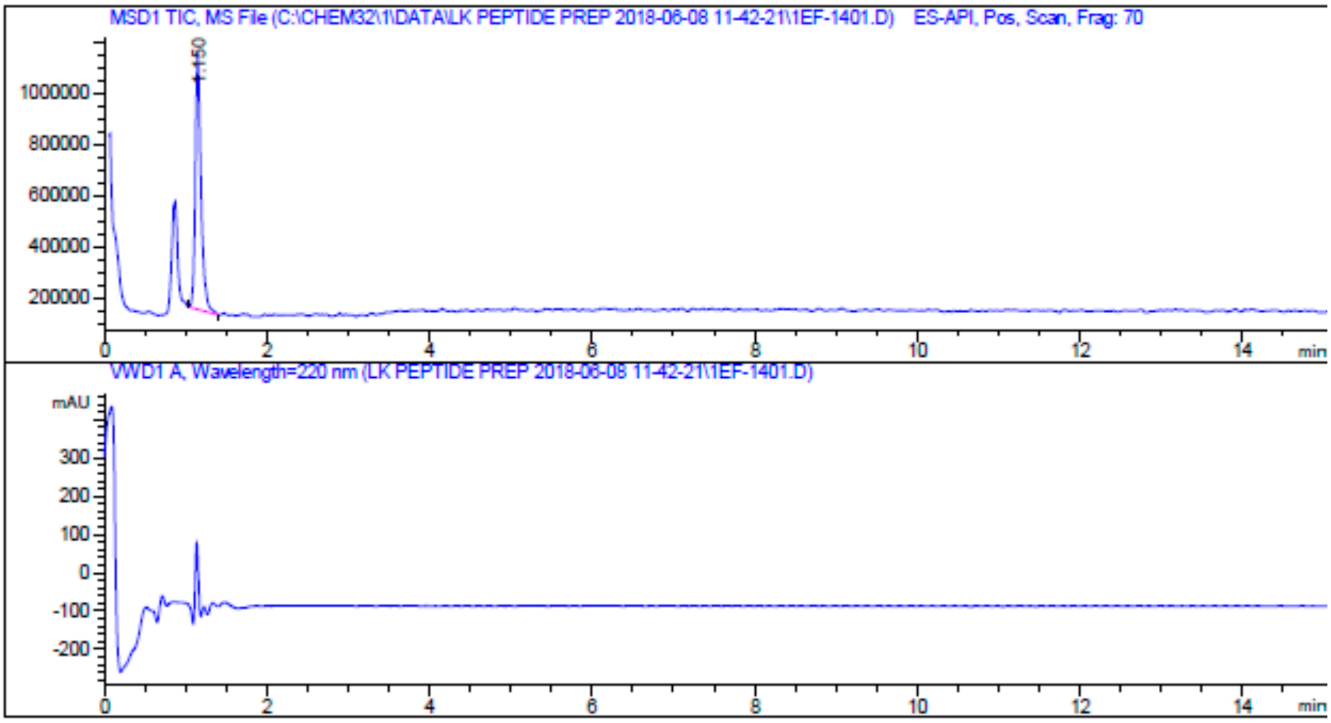


```
=====================================================================
                      Area Percent Report
=====================================================================

Sorted By             :      Signal
Multiplier:                  :      1.0000
Dilution:                    :      1.0000
Use Multiplier & Dilution Factor with ISTDs


Signal 1: MSD1 TIC, MS File

Peak RetTime Type  Width     Area      Height     Area
  #   [min]        [min]                           %
----|-------|----|-------|----------|----------|--------|
   1   1.150 BB     0.0730 4.93549e6  1.01672e6  100.0000

Totals :                   4.93549e6  1.01672e6
```

Benzyl-*N*-[(1*S*)-1-{[(1*R*)-1-(1H-1,2,3,4-tetrazol-5-yl)ethyl]carbamoyl}ethyl]carbamate **13a-LD**:

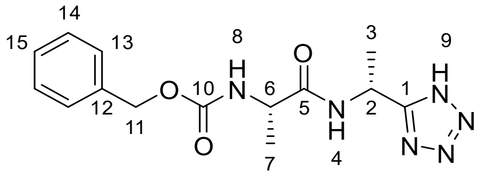

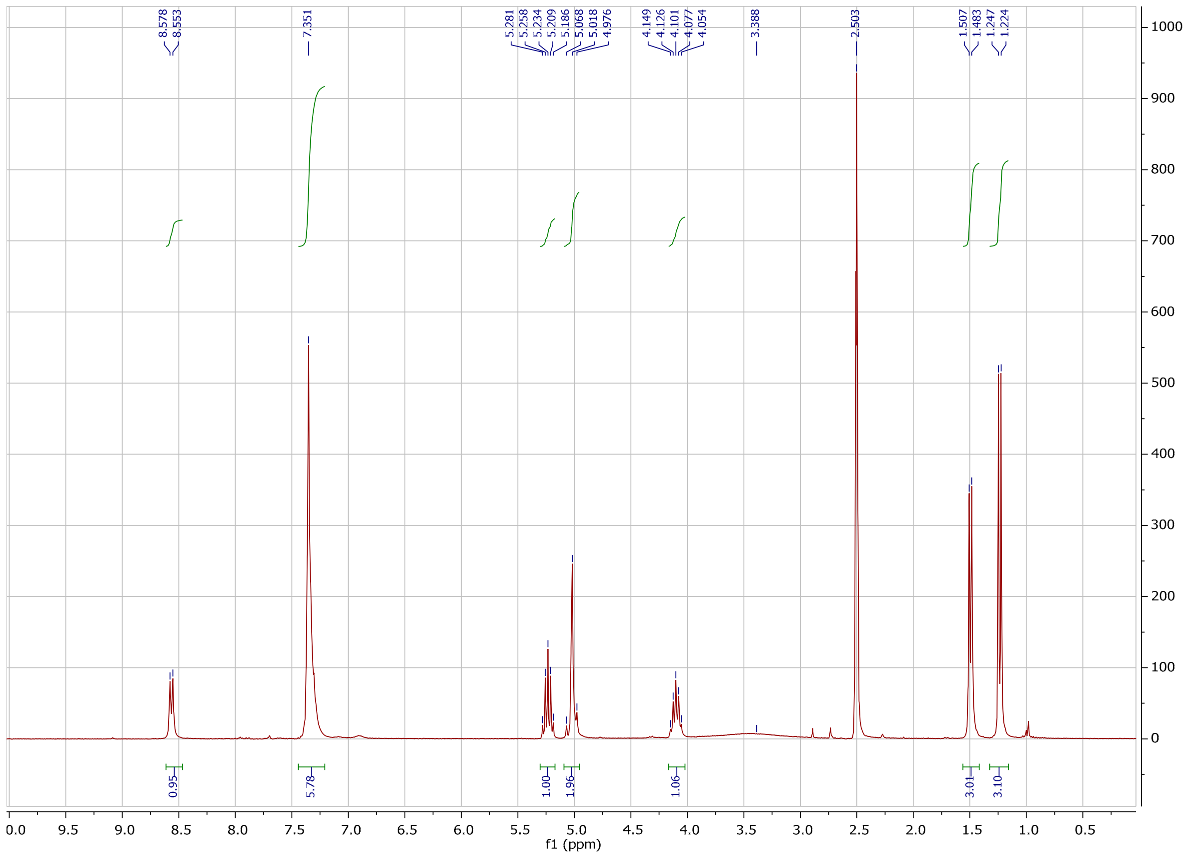

8.578
8.553
7.351
5.281
5.258
5.234
5.209
5.186
5.068
5.018
4.976
4.149
4.126
4.101
4.077
4.054
3.388
2.503
1.507
1.483
1.247
1.224
0.95
5.78
1.00
1.96
1.06
3.01
3.10
f1 (ppm)

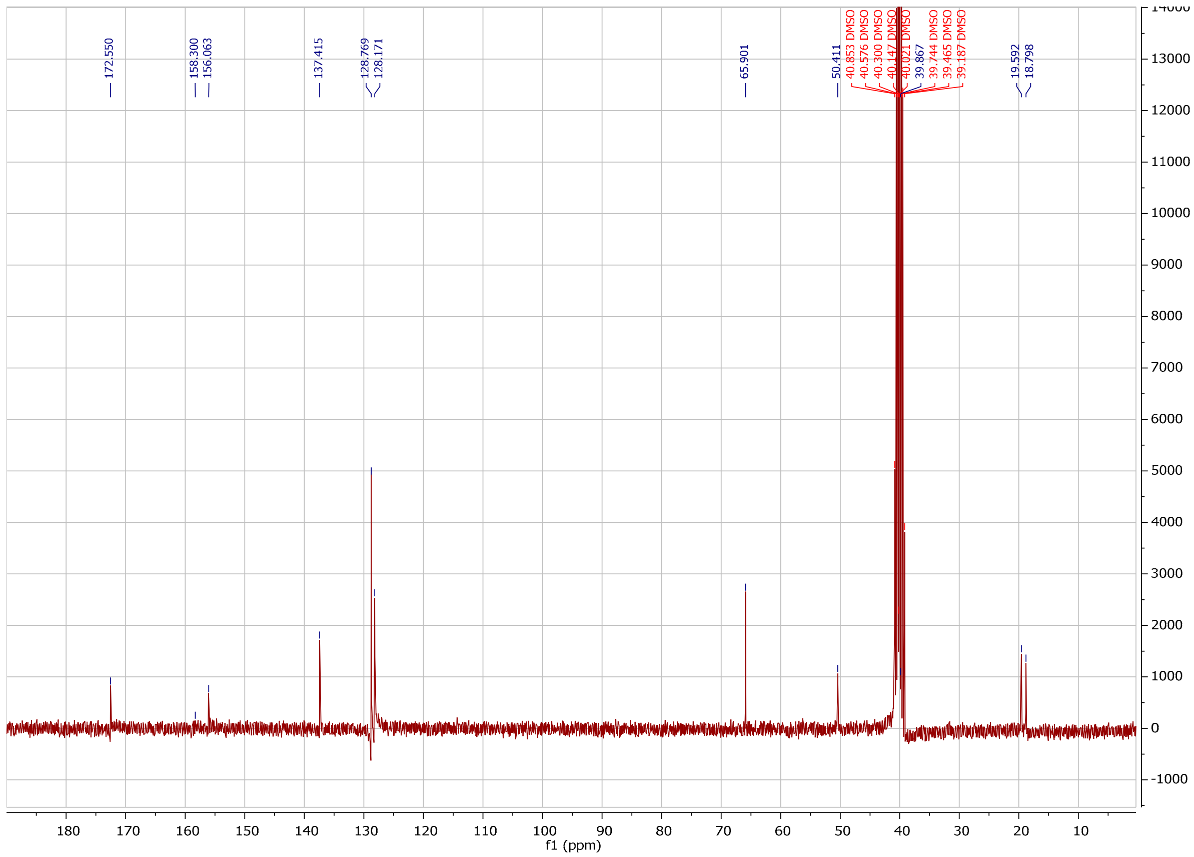

172.550
158.300
156.063
137.415
128.769
128.171
65.901
50.411
40.853 DMSO
40.576 DMSO
40.300 DMSO
40.147 DMSO
40.021 DMSO
39.867
39.744 DMSO
39.465 DMSO
39.187 DMSO
19.592
18.798
f1 (ppm)

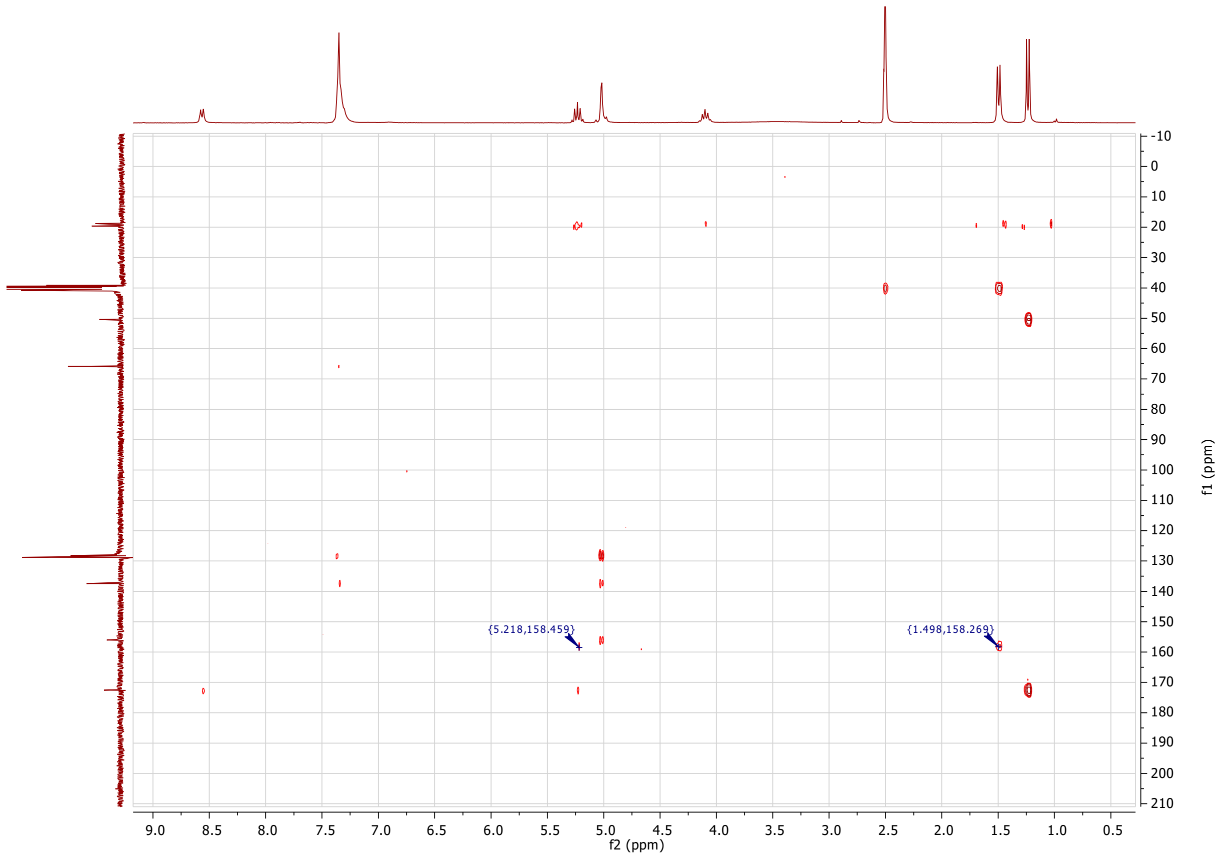

{5.218,158.459}
{1.498,158.269}
f2 (ppm)
f1 (ppm)

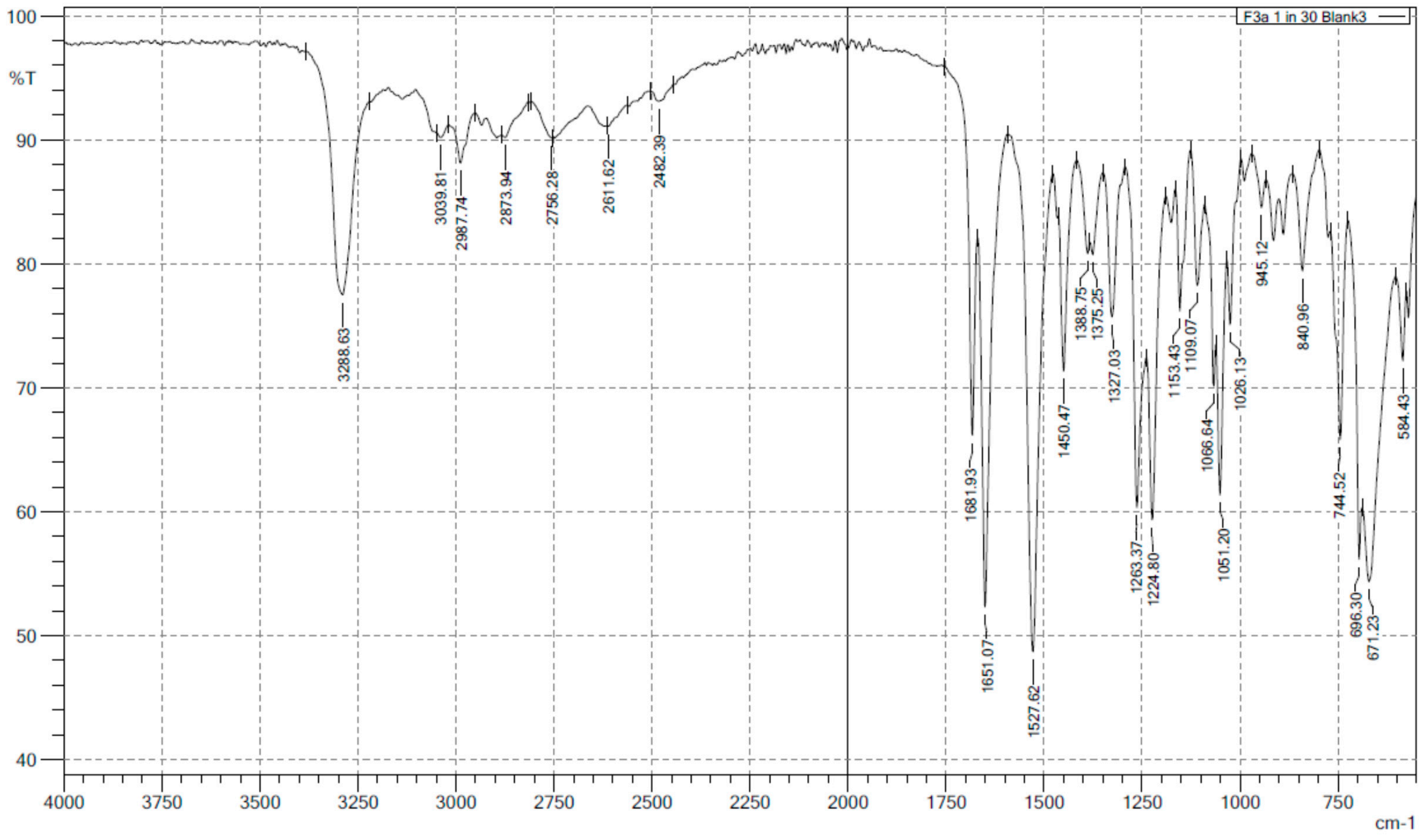

F3a 1 in 30 Blank3
%T
cm-1
3288.63
3039.81
2987.74
2873.94
2756.28
2611.62
2482.39
1681.93
1651.07
1527.62
1450.47
1388.75
1375.25
1327.03
1263.37
1224.80
1153.43
1109.07
1066.64
1051.20
1026.13
945.12
840.96
744.52
696.30
671.23
584.43

(2*S*)-2-amino-*N*-[(1*R*)-1-(1*H*-1,2,3,4-tetrazol-5-yl)ethyl]propanamide **14a-LD**:

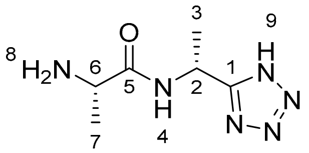

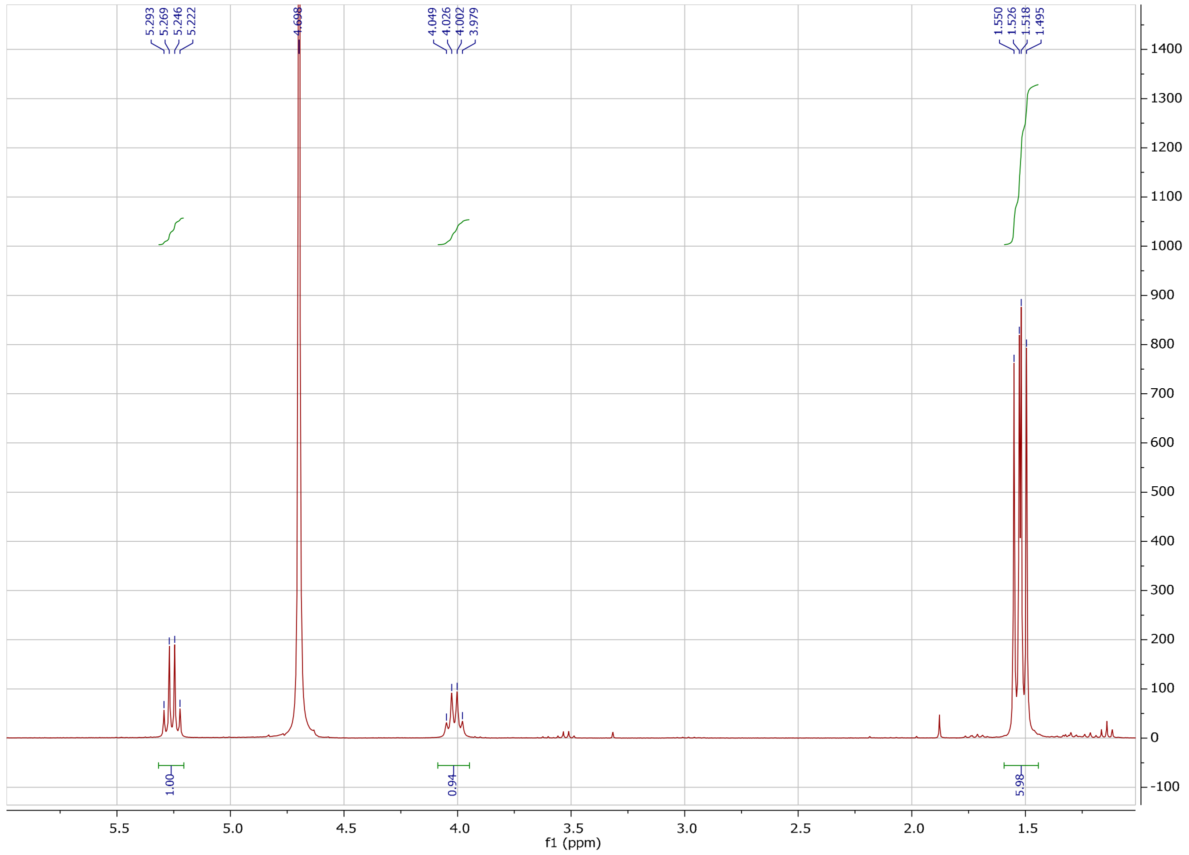

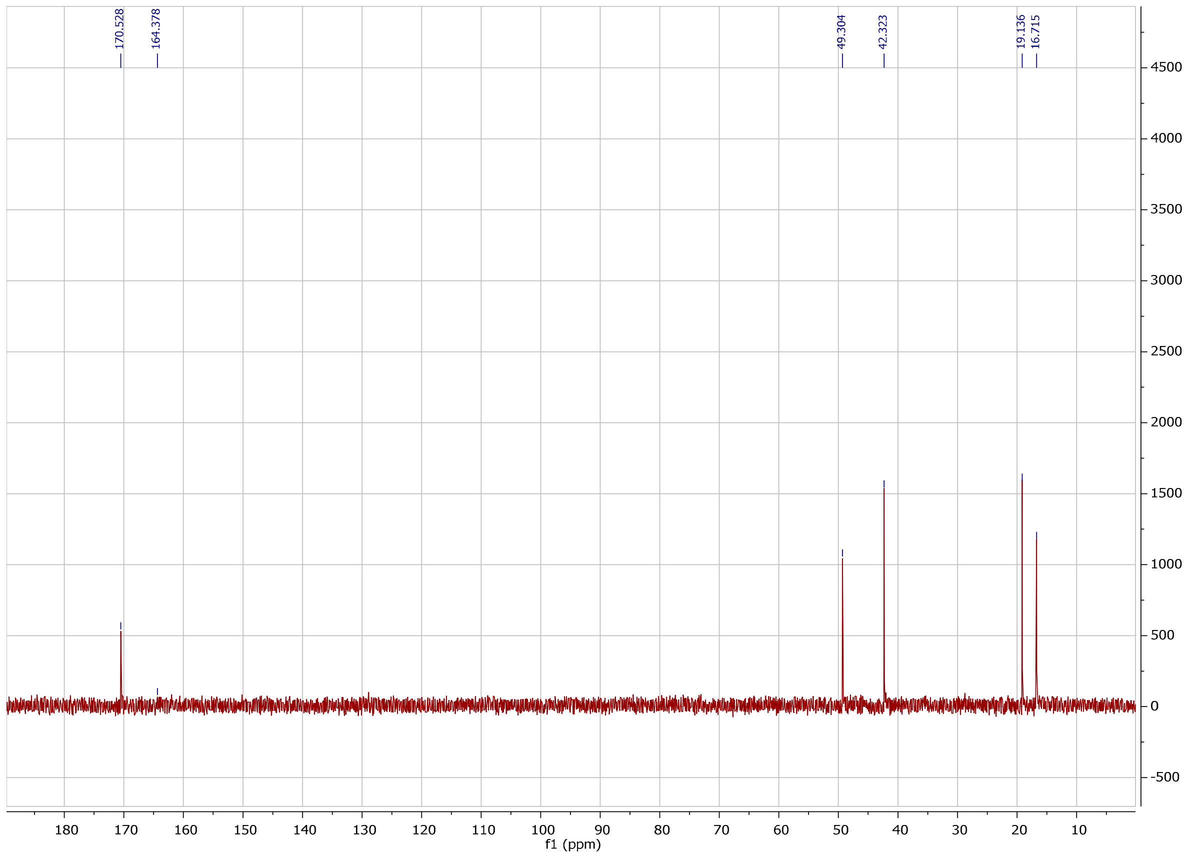
170.528
164.378
49.304
42.323
19.136
16.715
f1 (ppm)

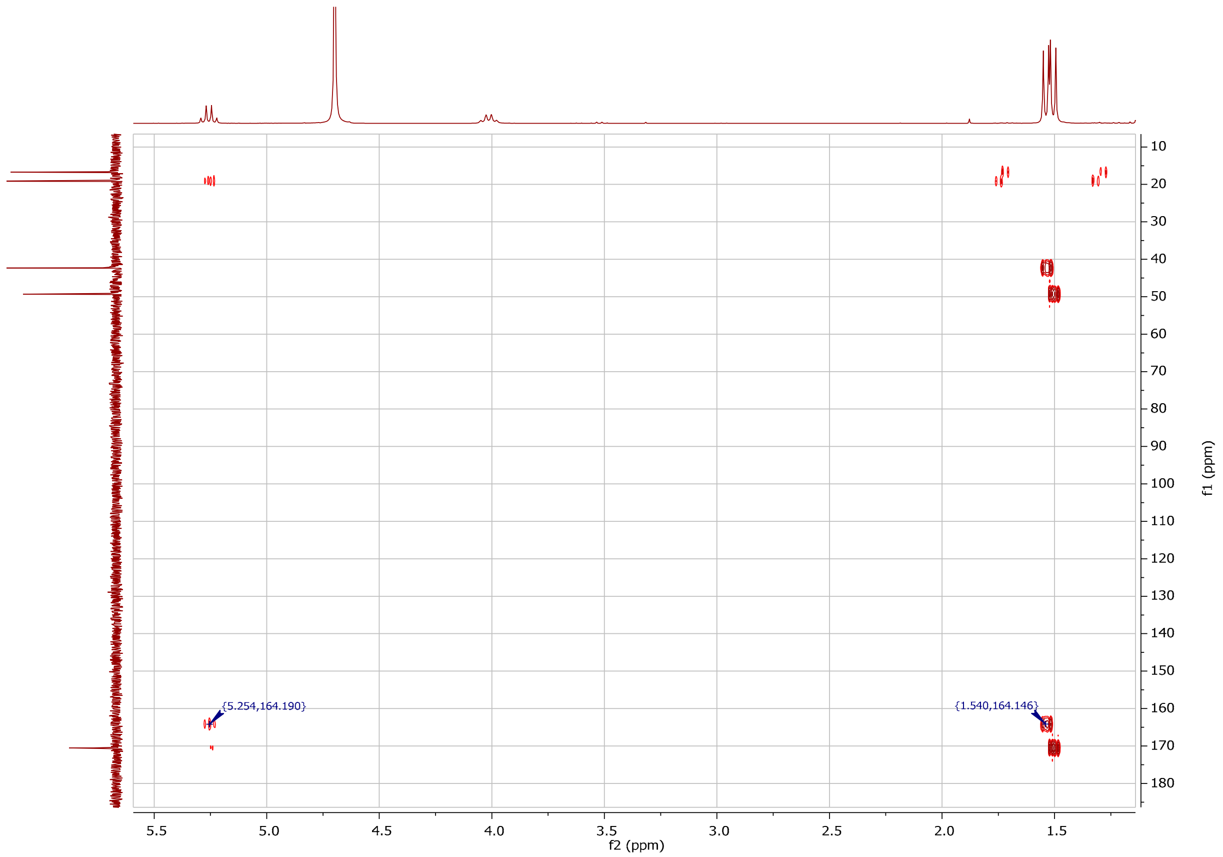
{5.254,164.190}
{1.540,164.146}
f2 (ppm)
f1 (ppm)

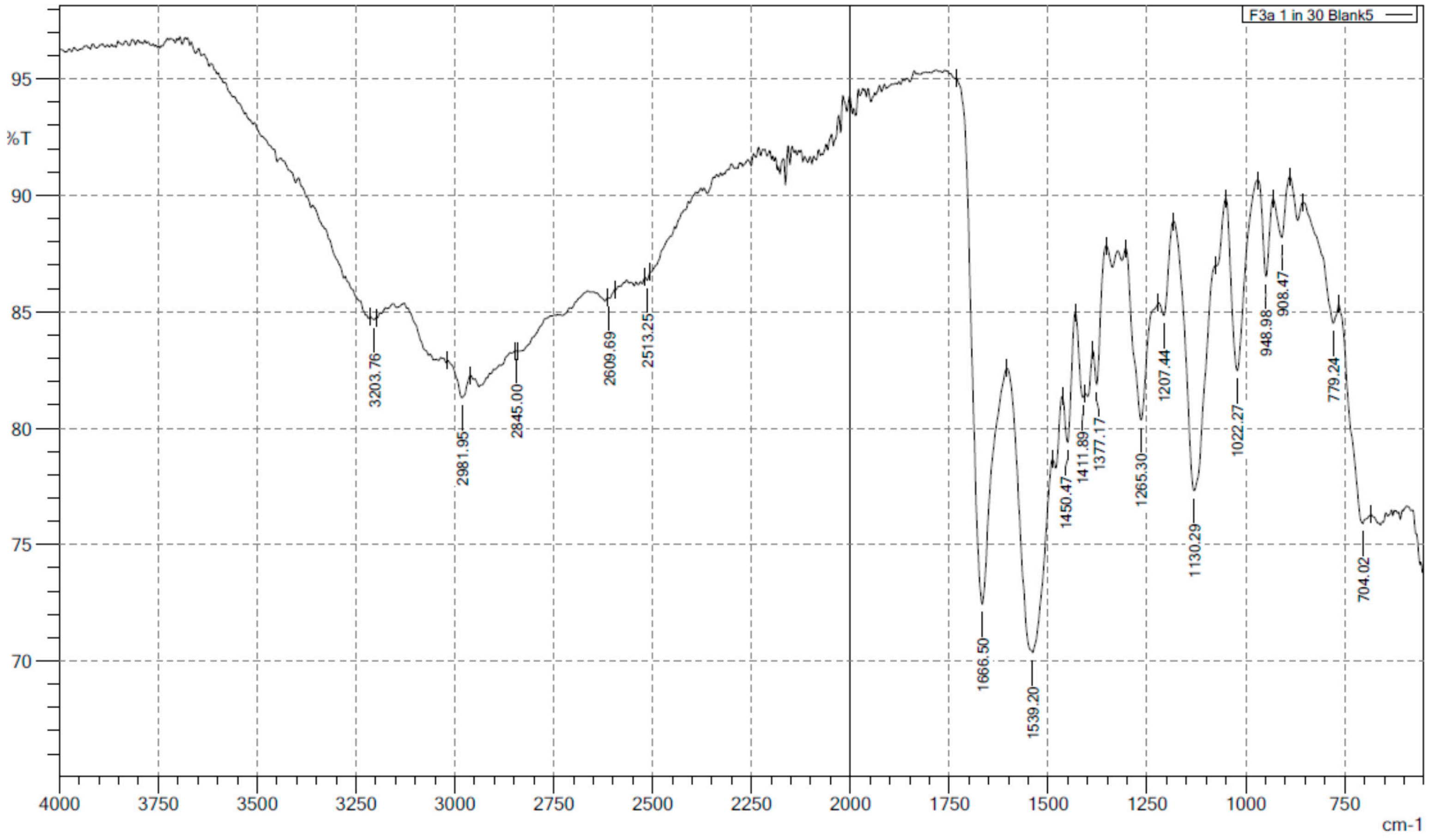

F3a 1 in 30 Blank5
%T
95
90
85
80
75
70
4000
3750
3500
3250
3000
2750
2500
2250
2000
1750
1500
1250
1000
750
cm-1
3203.76
2981.95
2845.00
2609.69
2513.25
1666.50
1539.20
1450.47
1411.89
1377.17
1265.30
1207.44
1130.29
1022.27
948.98
908.47
779.24
704.02

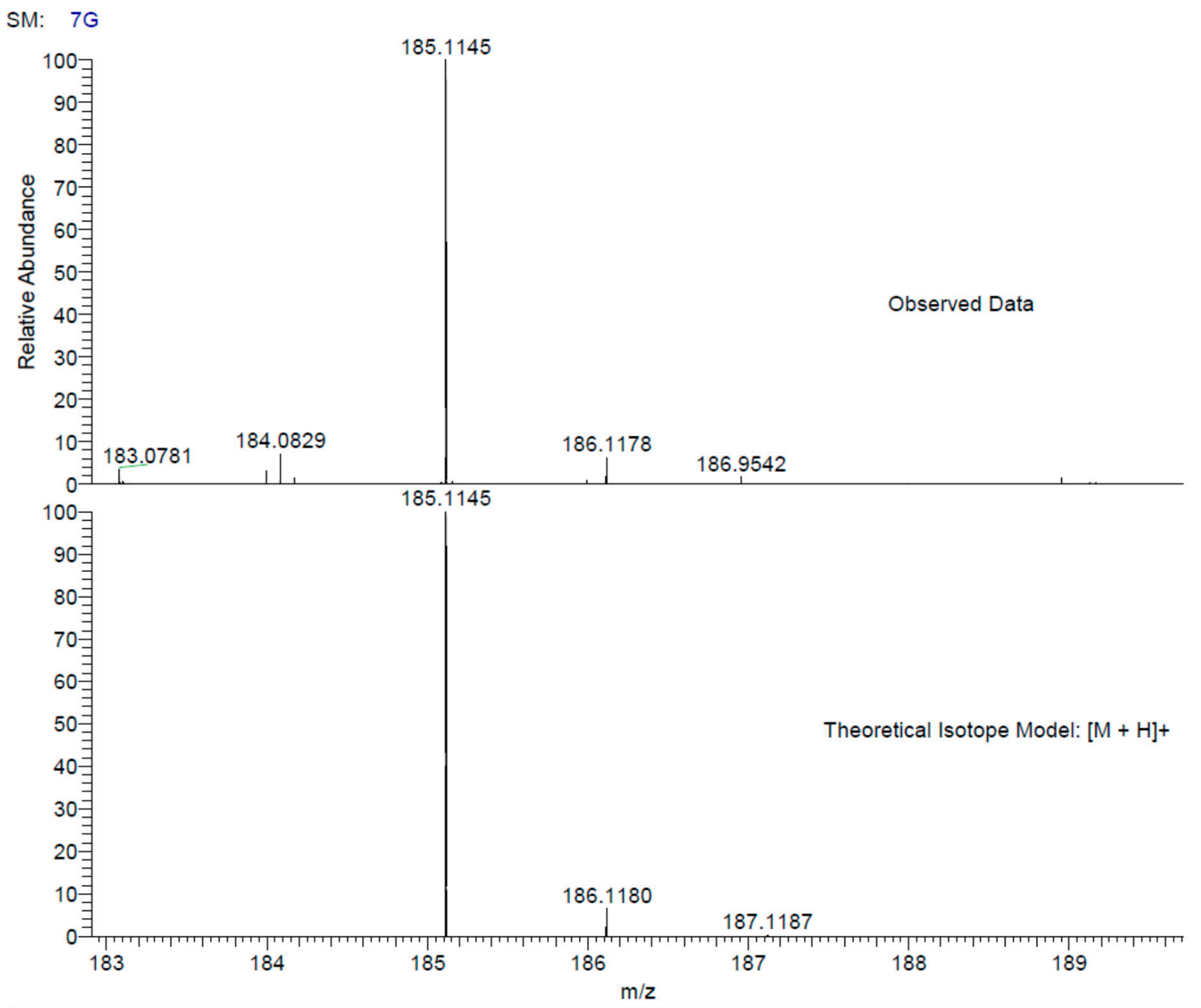

SM: 7G
185.1145
Relative Abundance
Observed Data
183.0781
184.0829
186.1178
186.9542
185.1145
Theoretical Isotope Model: [M + H]+
186.1180
187.1187
m/z

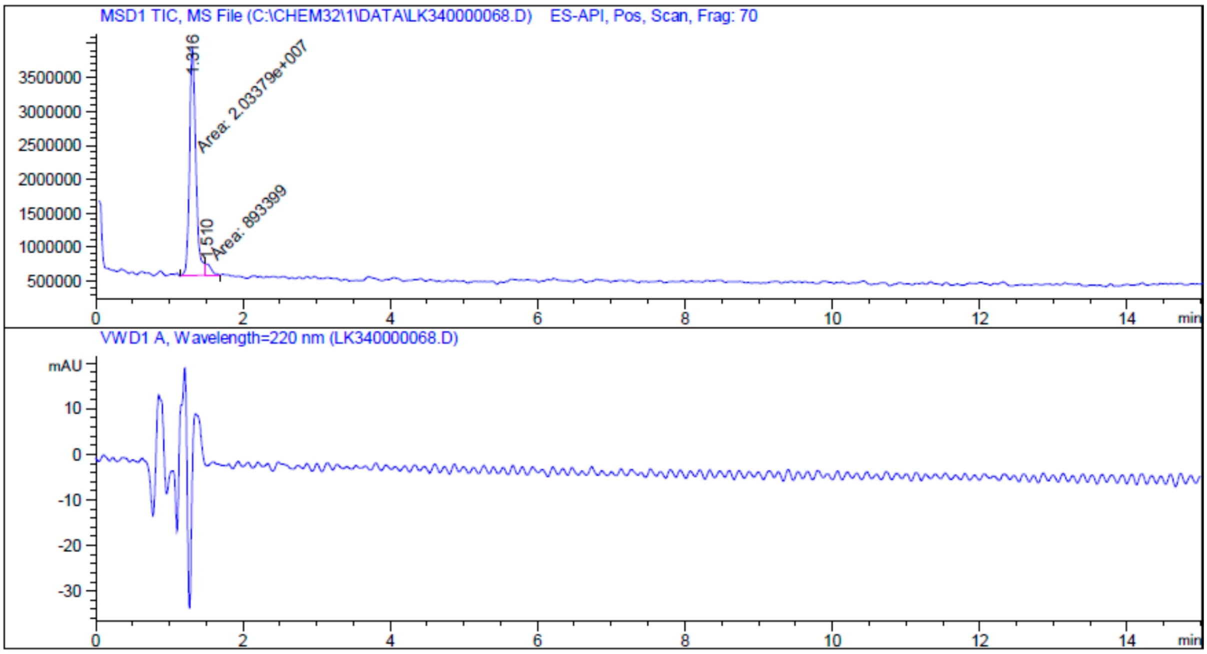


```
=====================================================================
                         Area Percent Report
=====================================================================

Sorted By             :      Signal
Multiplier:                  :       1.0000
Dilution:                    :       1.0000
Use Multiplier & Dilution Factor with ISTDs


Signal 1: MSD1 TIC, MS File
```

Peak #	RetTime [min]	Type	Width [min]	Area	Height	Area %
1	1.316	MF	0.0976	2.03379e7	3.47199e6	95.7921
2	1.510	FM	0.0882	8.93399e5	1.68763e5	4.2079

Totals : 2.12313e7 3.64076e6

(*S*)-Benzyl-(3-((1-(1*H*-tetrazol-5-yl)ethyl)amino)-3-oxopropyl)carbamate **13d-L**:

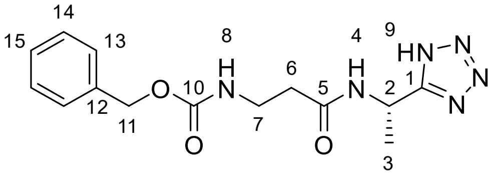

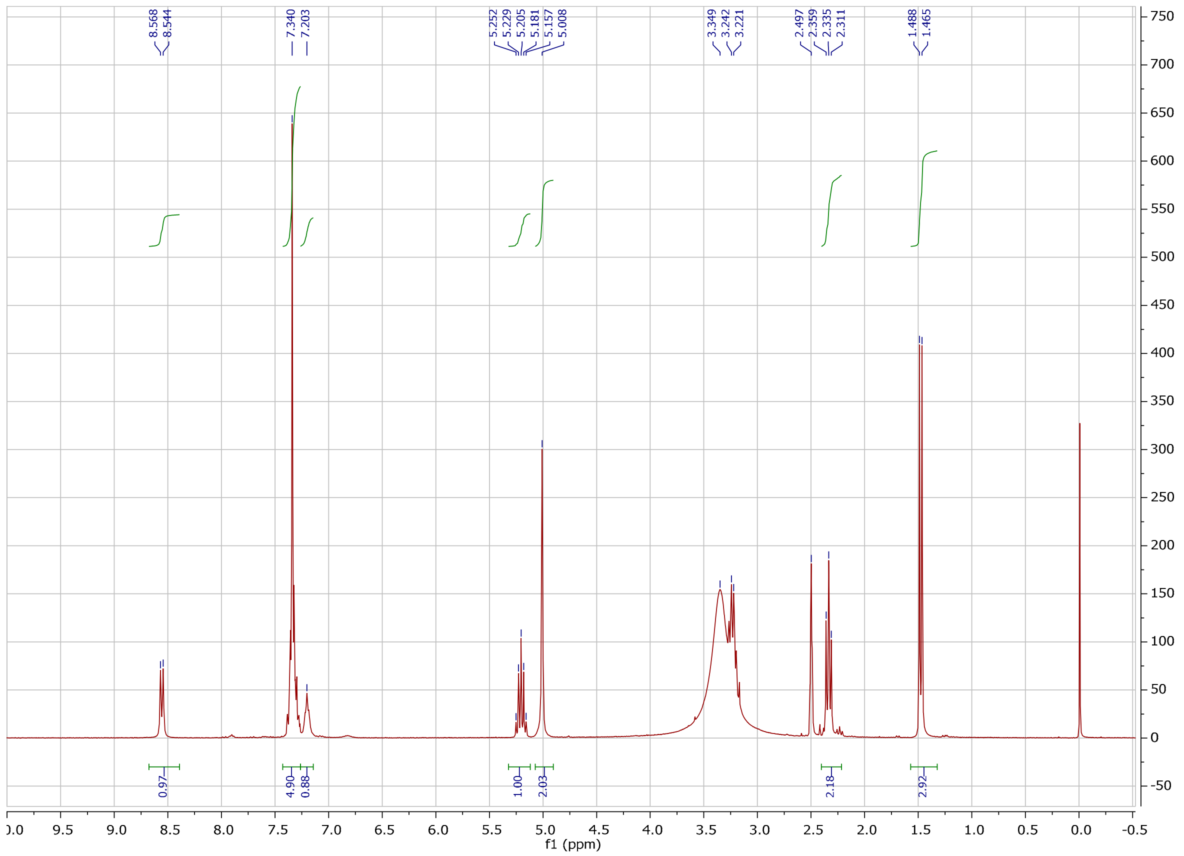

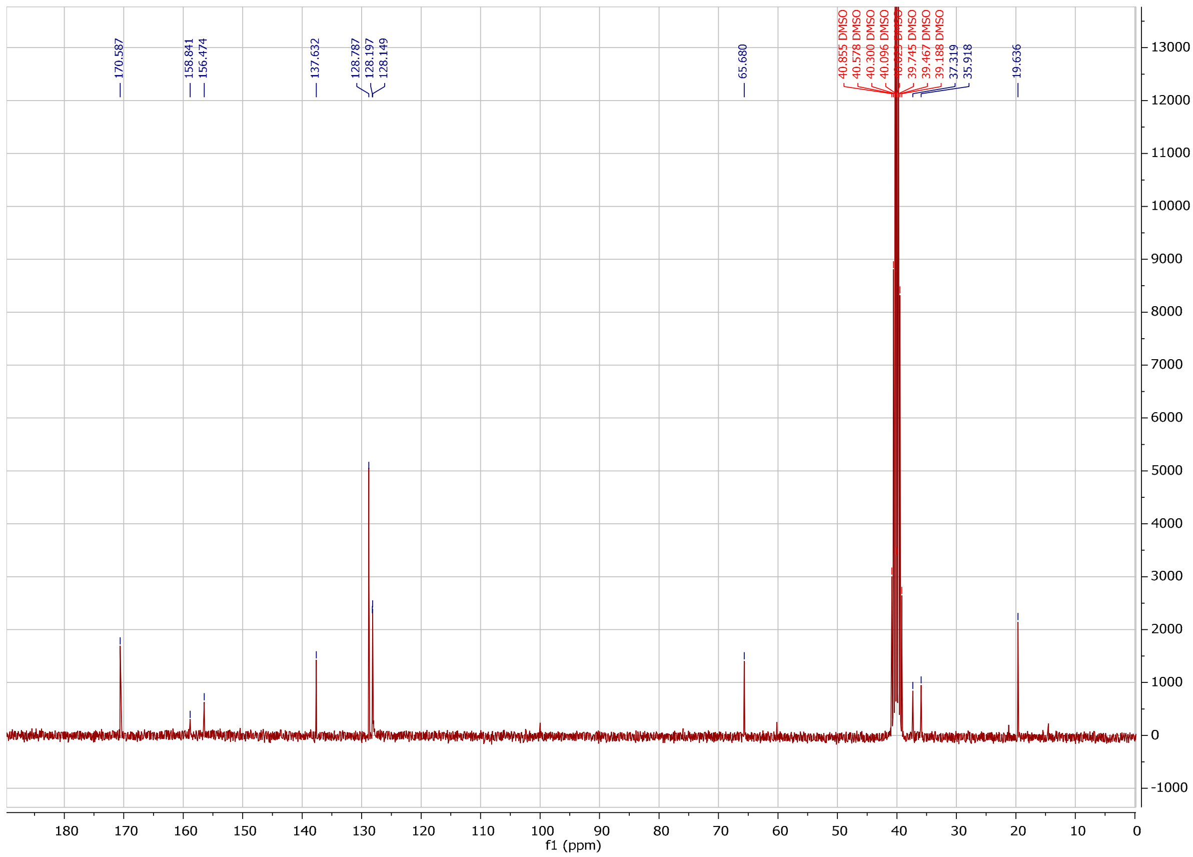

SM: 7G

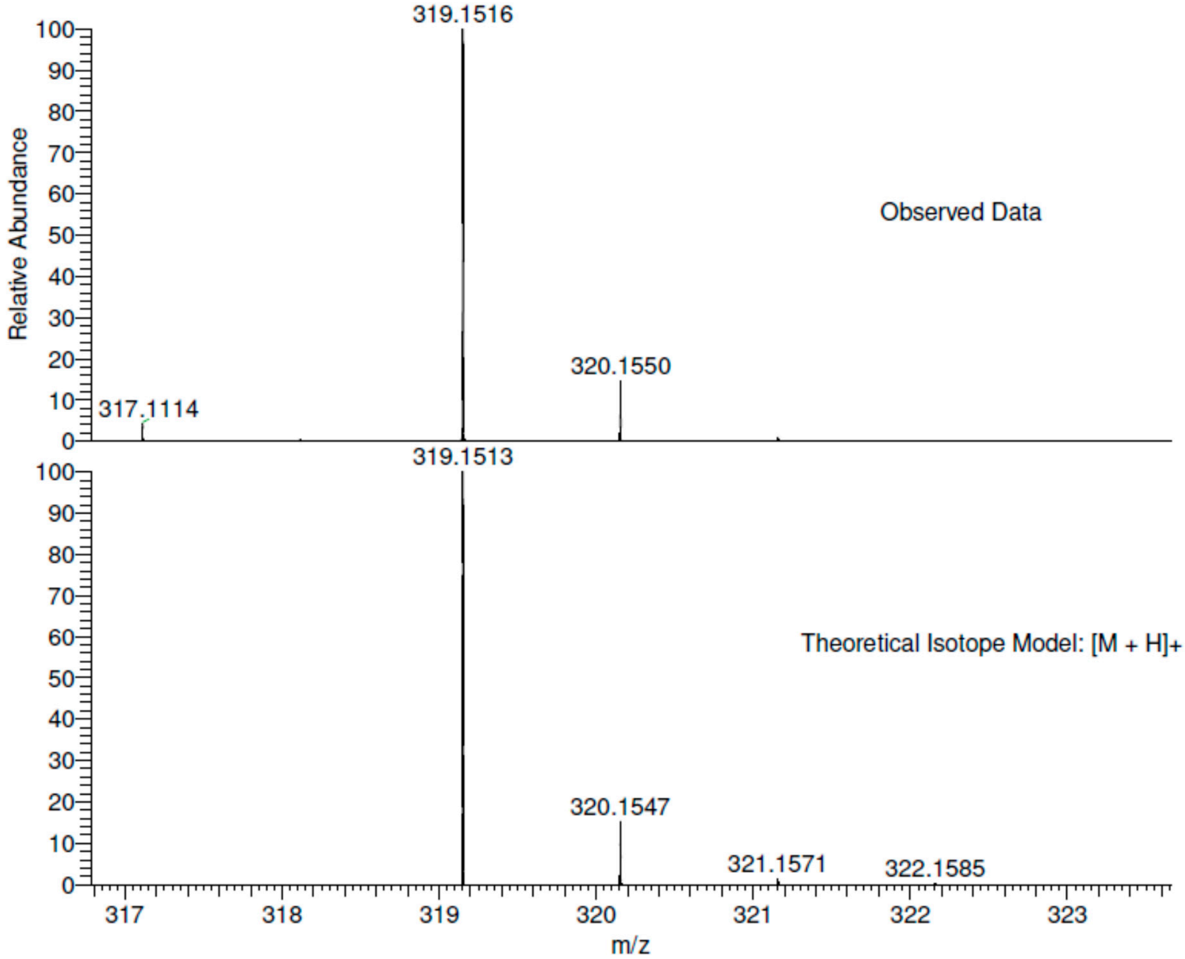

319.1516
Relative Abundance
Observed Data
320.1550
317.1114
319.1513
Theoretical Isotope Model: [M + H]+
320.1547
321.1571
322.1585
m/z

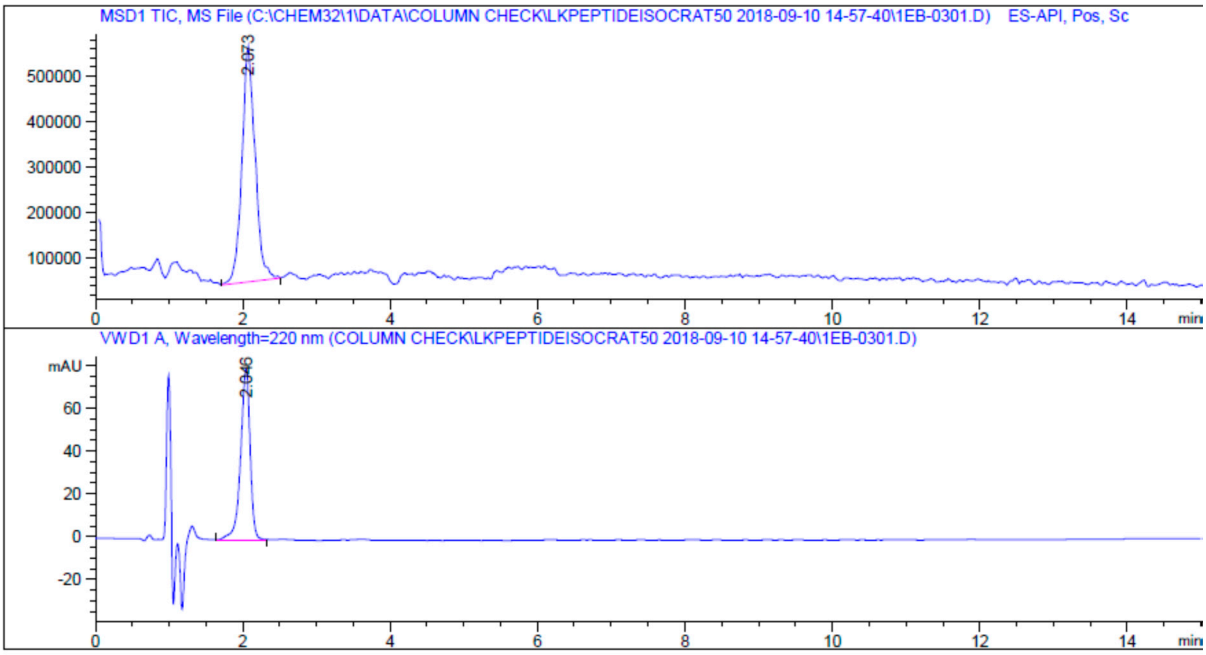


```
=====================================================================
                         Area Percent Report
=====================================================================

Sorted By              :      Signal
Multiplier:                   :      1.0000
Dilution:                     :      1.0000
Use Multiplier & Dilution Factor with ISTDs


Signal 1: MSD1 TIC, MS File

Peak RetTime Type  Width      Area       Height      Area
  #   [min]        [min]                              %
----|-------|----|-------|----------|----------|--------|
   1    2.073 BB    0.1717 6.38522e6  5.16189e5  100.0000

Totals :                   6.38522e6  5.16189e5
```

3-Amino-*N*-[(1*S*)-1-(1H-1,2,3,4-tetrazol-5-yl)ethyl]propanamide **14d-L**:

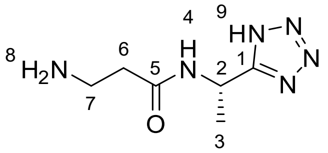

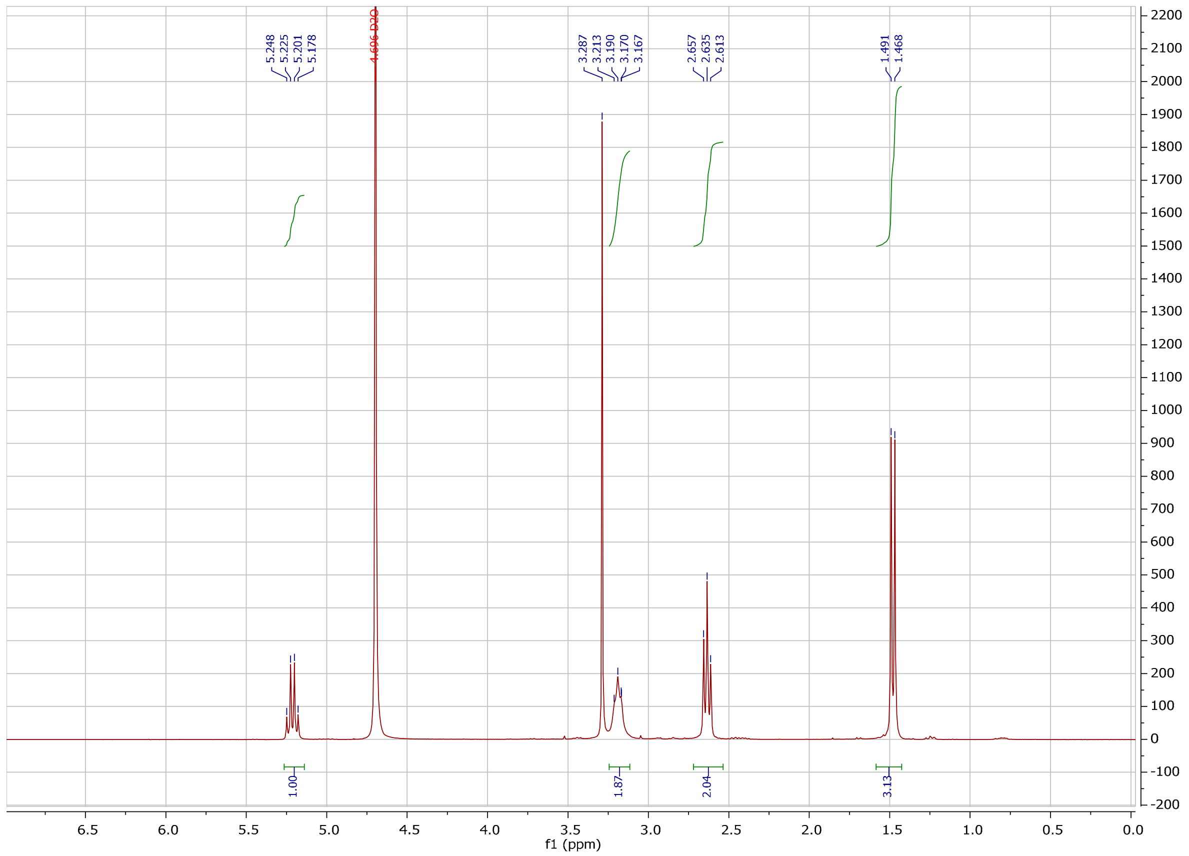
5.248
5.225
5.201
5.178
4.696 D2O
3.287
3.213
3.190
3.170
3.167
2.657
2.635
2.613
1.491
1.468
1.00
1.87
2.04
3.13
f1 (ppm)

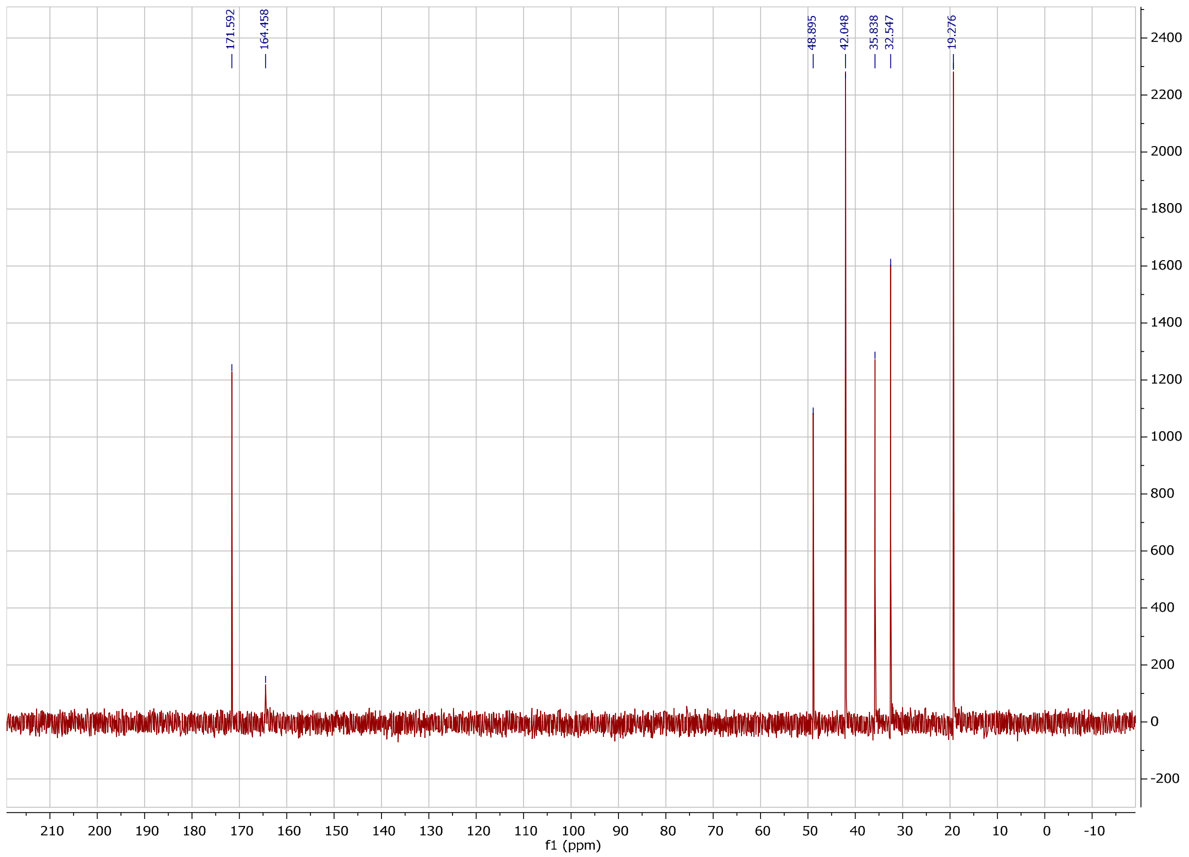
171.592
164.458
48.895
42.048
35.838
32.547
19.276
f1 (ppm)

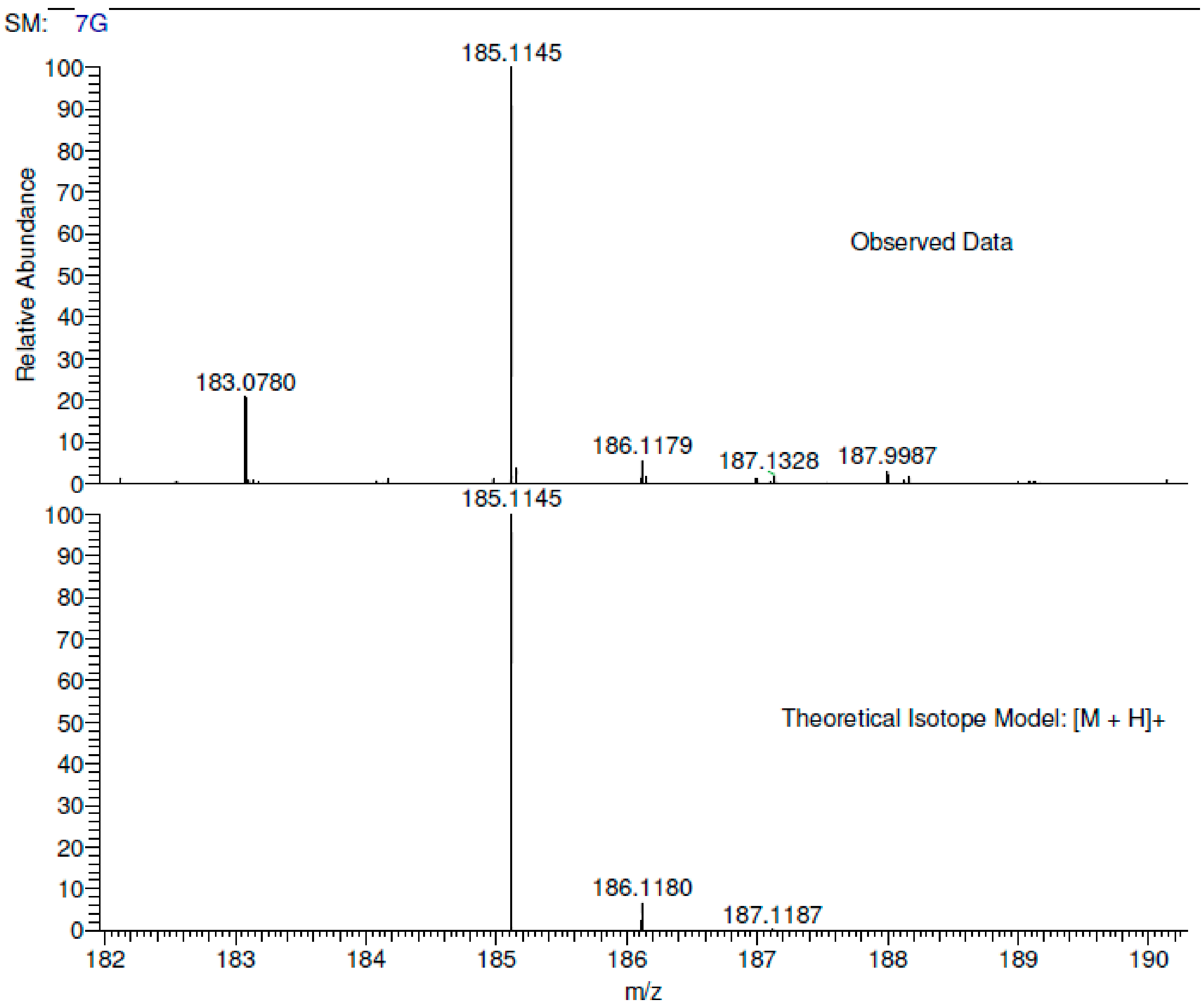

SM: 7G
185.1145
Observed Data
183.0780
186.1179
187.1328
187.9987
185.1145
Theoretical Isotope Model: [M + H]+
186.1180
187.1187
Relative Abundance
m/z

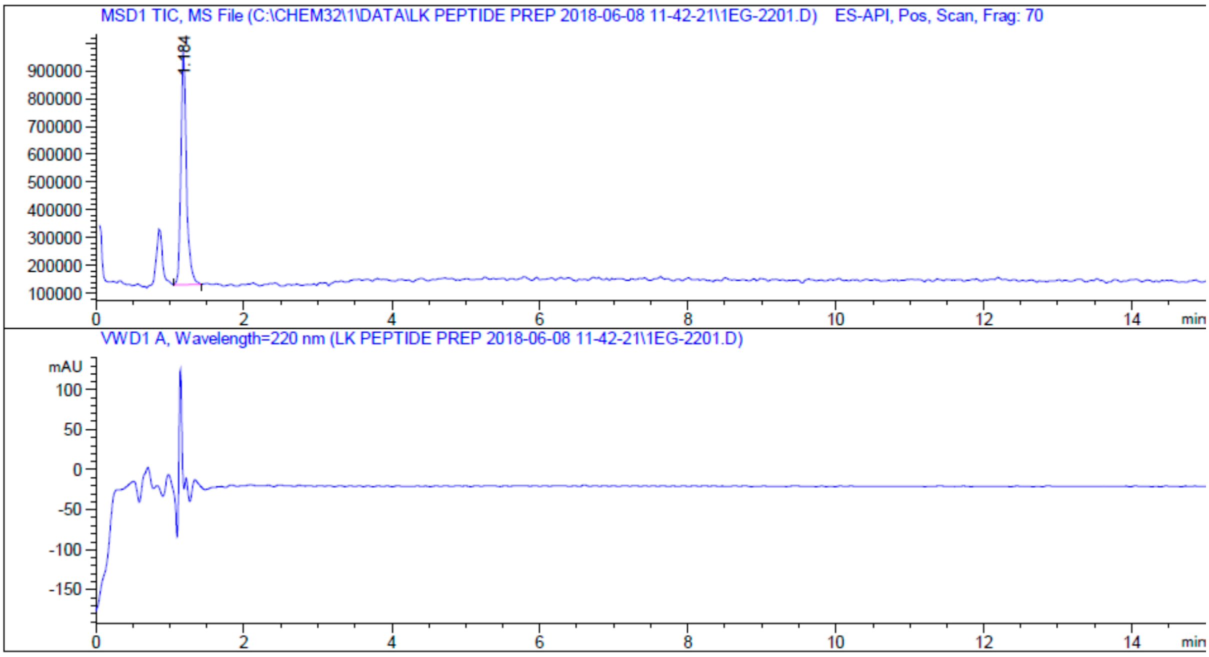


```
=====================================================================
                         Area Percent Report
=====================================================================

Sorted By             :      Signal
Multiplier:                   :      1.0000
Dilution:                     :      1.0000
Use Multiplier & Dilution Factor with ISTDs


Signal 1: MSD1 TIC, MS File

Peak RetTime Type  Width     Area      Height     Area
  #   [min]        [min]                            %
----|-------|----|-------|----------|----------|--------|
   1   1.184 BB    0.0759 4.37722e6  8.57835e5  100.0000

Totals :                  4.37722e6  8.57835e5
```

Benzyl-*N*-[(1*S*)-1-{[(1*S*)-1-(1*H*-1,2,3,4-tetrazol-5-yl)ethyl]carbamoyl}butyl]carbamate **13e-LL**:

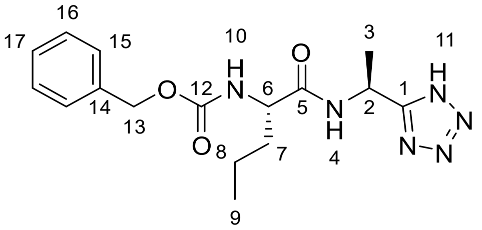

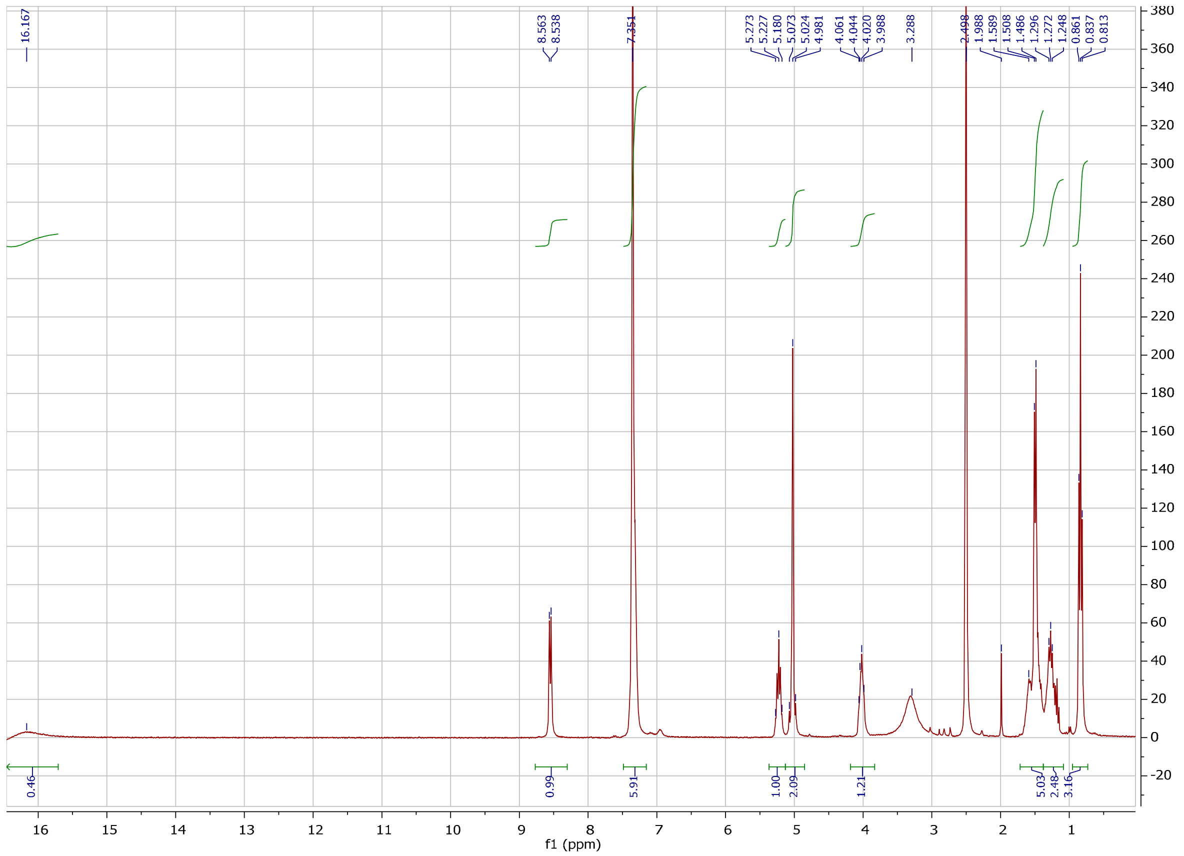
16.167
8.563
8.538
7.351
5.273
5.227
5.180
5.073
5.024
4.981
4.061
4.044
4.020
3.988
3.288
2.498
1.988
1.589
1.508
1.486
1.296
1.272
1.248
0.861
0.837
0.813
0.46
0.99
5.91
1.00
2.09
1.21
5.03
2.48
3.16
f1 (ppm)

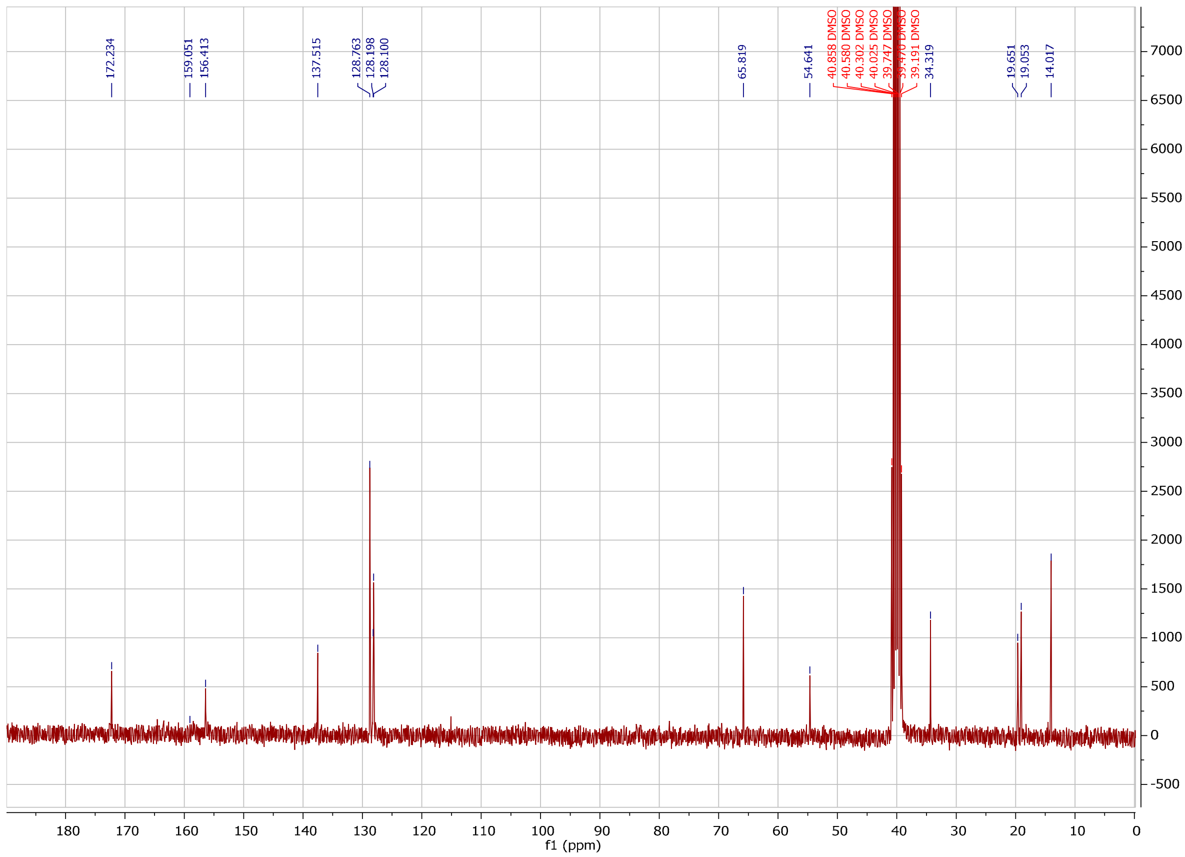
172.234
159.051
156.413
137.515
128.763
128.198
128.100
65.819
54.641
40.858 DMSO
40.580 DMSO
40.302 DMSO
40.025 DMSO
39.747 DMSO
39.470 DMSO
39.191 DMSO
34.319
19.651
19.053
14.017
f1 (ppm)

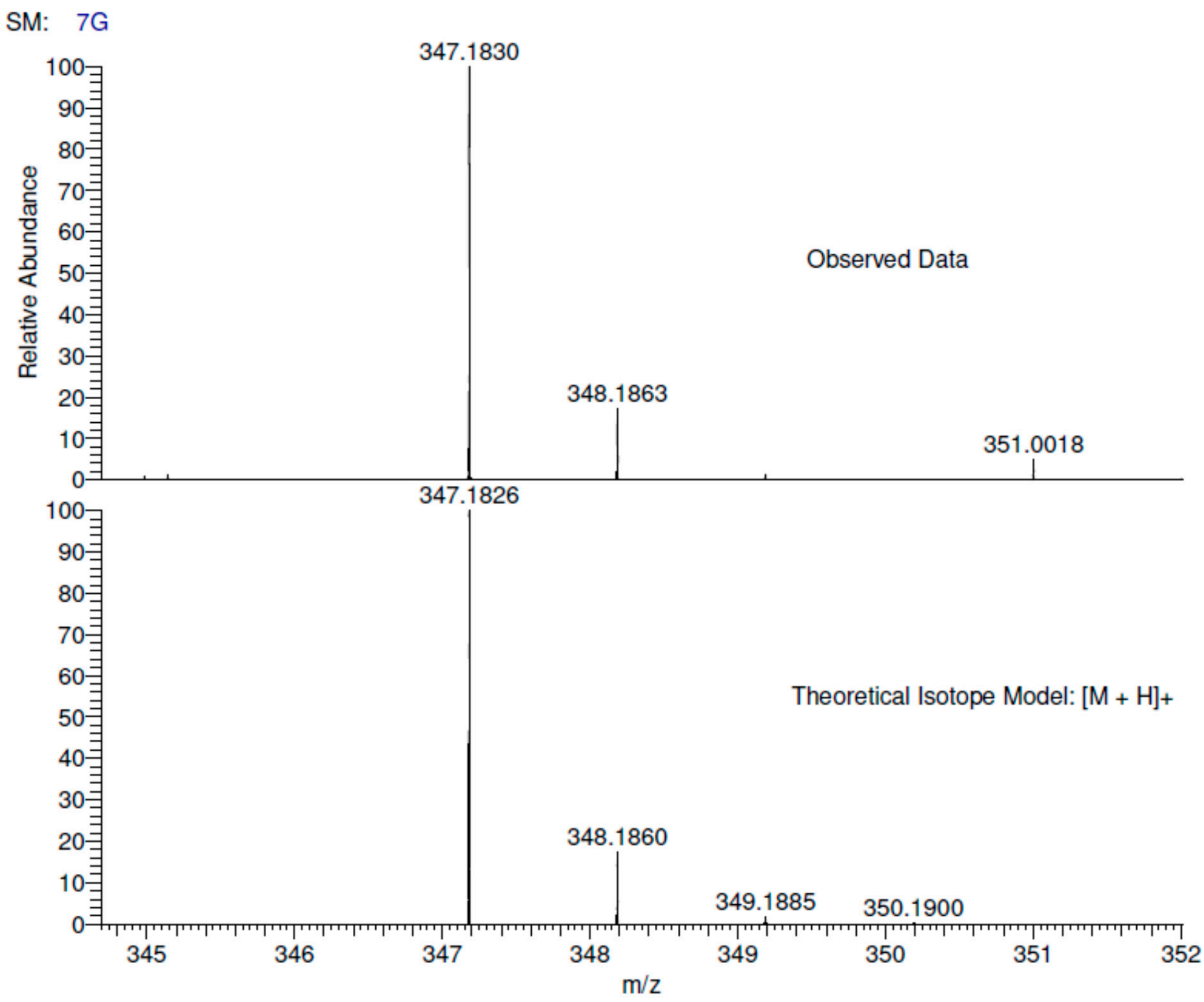

SM: 7G
347.1830
348.1863
351.0018
Observed Data
Relative Abundance
347.1826
348.1860
349.1885
350.1900
Theoretical Isotope Model: [M + H]+
m/z

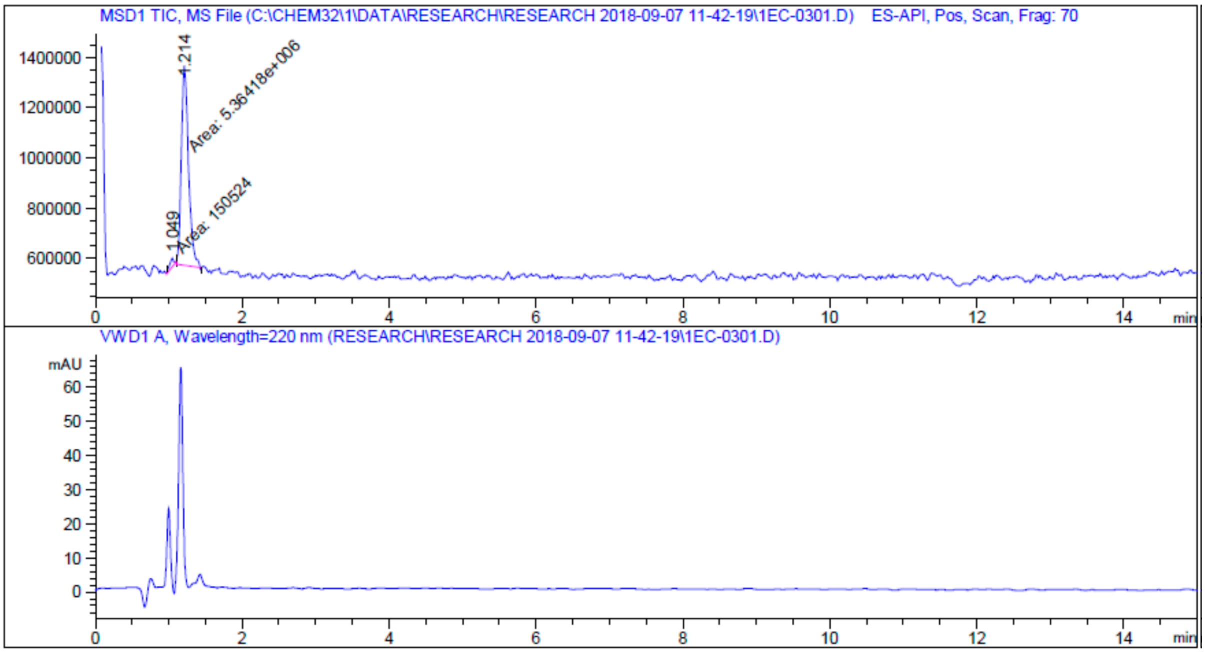


```
=====================================================================
                         Area Percent Report
=====================================================================

Sorted By             :      Signal
Multiplier:                  :      1.0000
Dilution:                    :      1.0000
Use Multiplier & Dilution Factor with ISTDs
```

Signal 1: MSD1 TIC, MS File

Peak #	RetTime [min]	Type	Width [min]	Area	Height	Area %
1	1.049	MM	0.0621	1.50524e5	4.04039e4	2.7295
2	1.214	MM	0.1118	5.36418e6	7.99972e5	97.2705

Totals : 5.51471e6 8.40376e5

(*S*)-*N*-((*S*)-1-(1*H*-tetrazol-5-yl)ethyl)-2-aminopentanamide **14e-LL**:

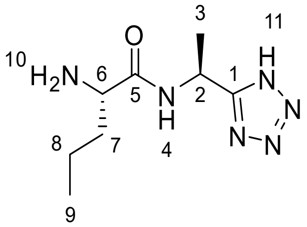

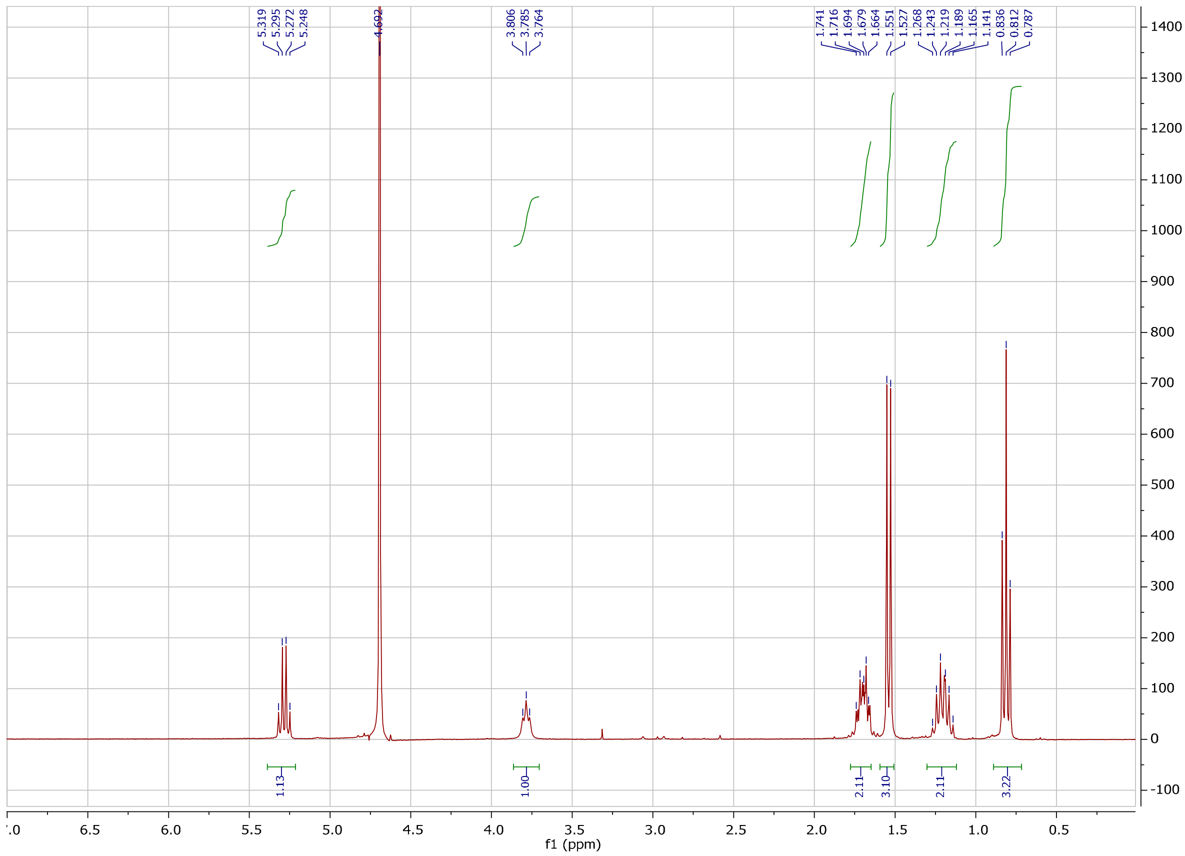
5.319
5.295
5.272
5.248
4.692
3.806
3.785
3.764
1.741
1.716
1.694
1.679
1.664
1.551
1.527
1.268
1.243
1.219
1.189
1.165
1.141
0.836
0.812
0.787
1.13
1.00
2.11
3.10
2.11
3.22
f1 (ppm)

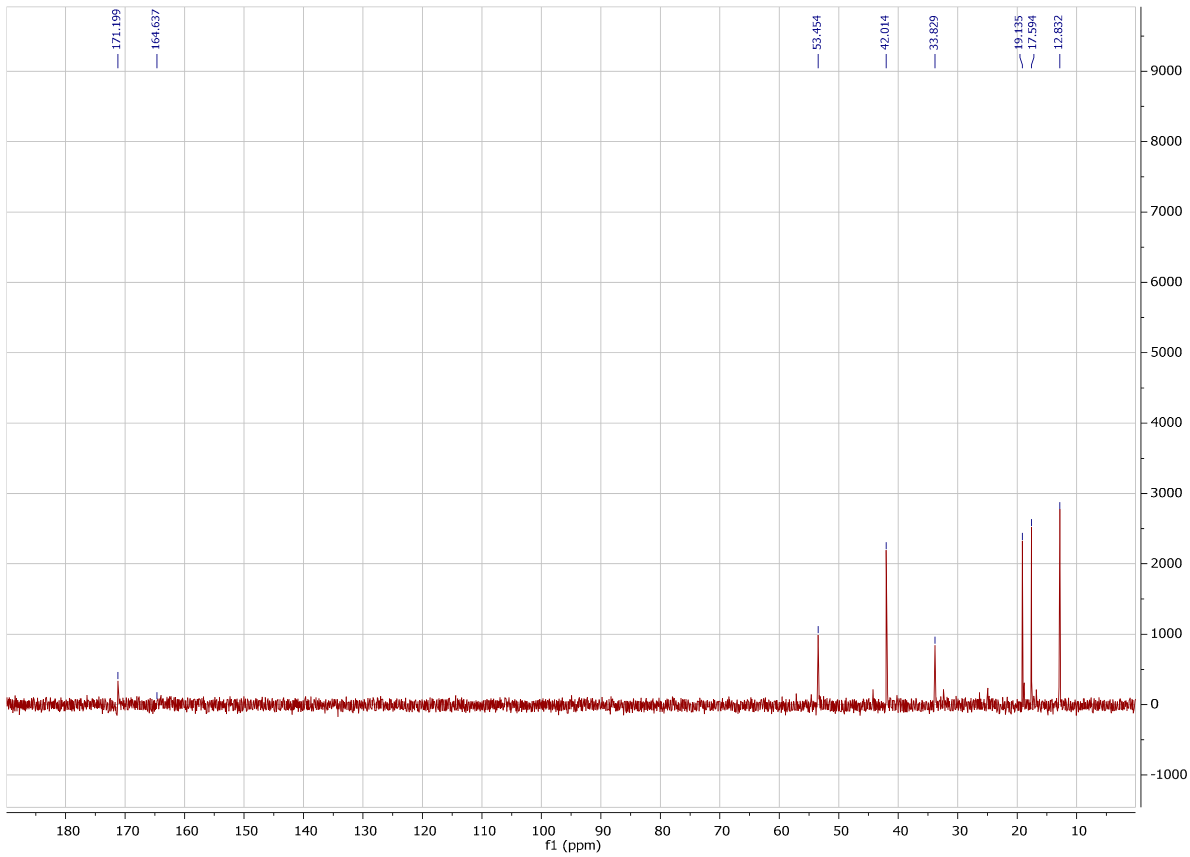
171.199
164.637
53.454
42.014
33.829
19.135
17.594
12.832
f1 (ppm)

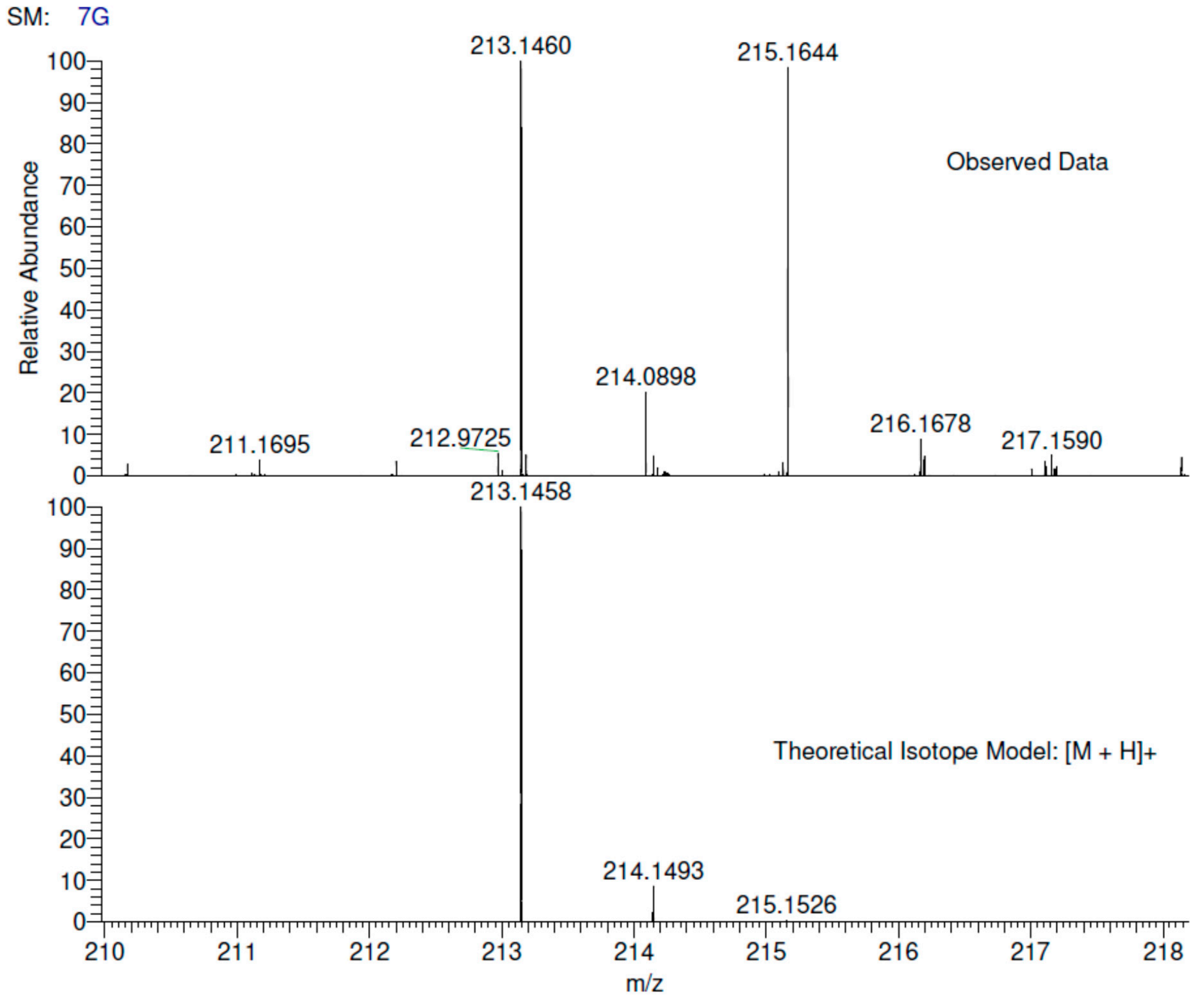

SM: 7G
213.1460
215.1644
Observed Data
Relative Abundance
214.0898
211.1695
212.9725
216.1678
217.1590
213.1458
Theoretical Isotope Model: [M + H]+
214.1493
215.1526
m/z

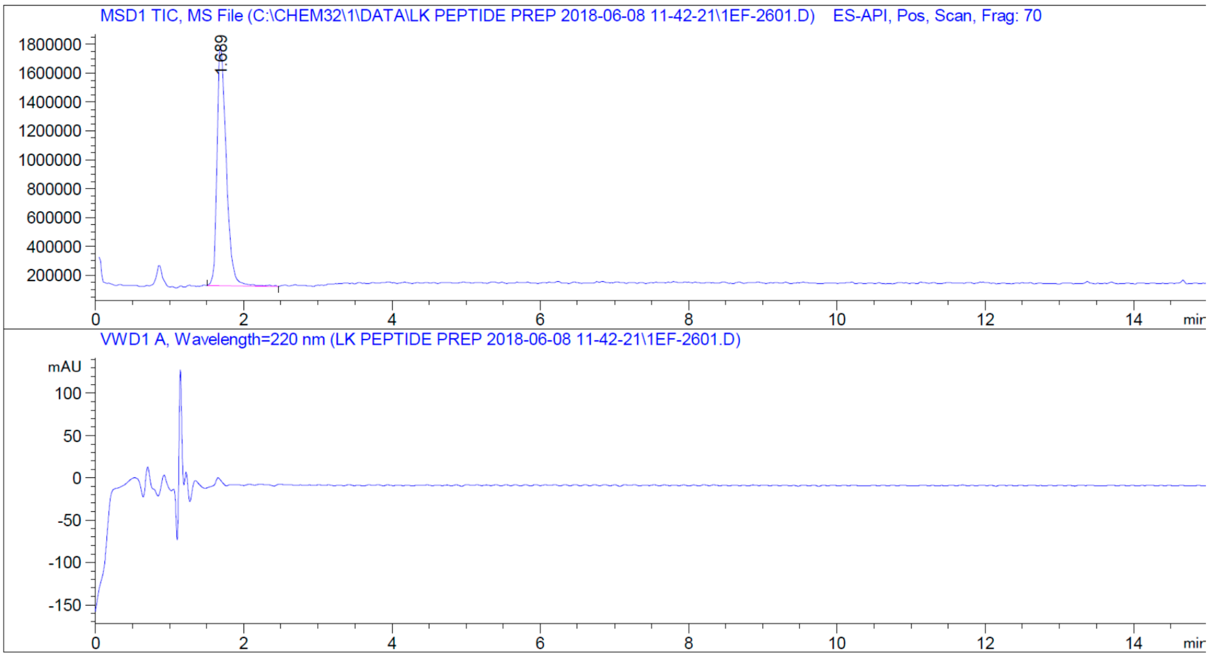

===
Area Percent Report
===

Sorted By : Signal
Multiplier: : 1.0000
Dilution: : 1.0000
Use Multiplier & Dilution Factor with ISTDs

Signal 1: MSD1 TIC, MS File

Peak #	RetTime [min]	Type	Width [min]	Area	Height	Area %
1	1.689	BB	0.1337	1.43625e7	1.66034e6	100.0000

Totals : 1.43625e7 1.66034e6

Benzyl-*N*-[(1*S*)-3-methyl-1-{[(1*S*)-1-(1*H*-1,2,3,4-tetrazol-5-yl)ethyl]carbamoyl}butyl]carbamate **13h-LL**:

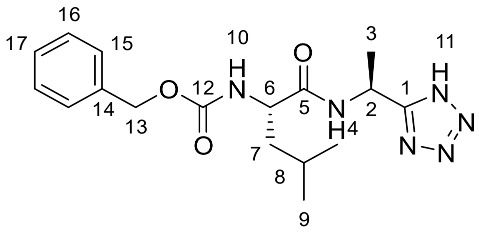

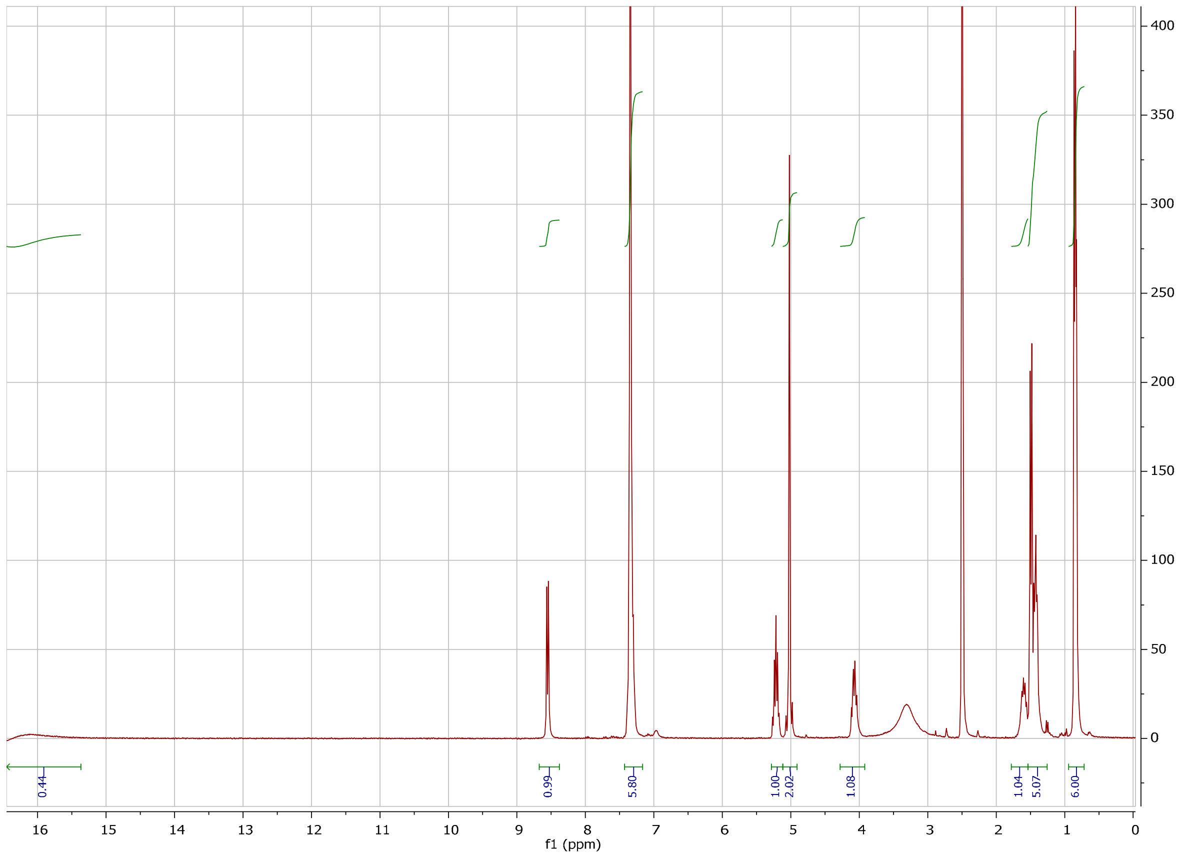
0.44
0.99
5.80
1.00
2.02
1.08
1.04
5.07
6.00
f1 (ppm)

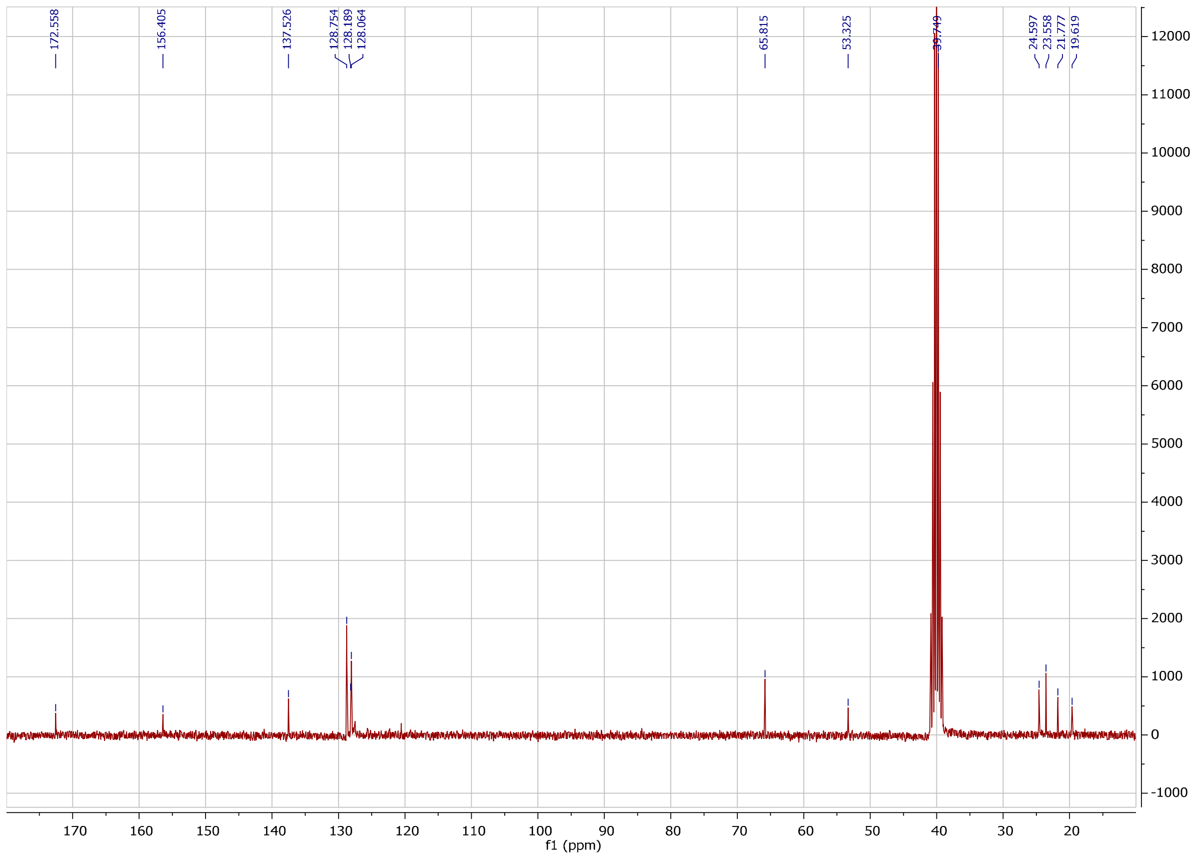
172.558
156.405
137.526
128.754
128.189
128.064
65.815
53.325
39.749
24.597
23.558
21.777
19.619
f1 (ppm)

(2*S*)-2-Amino-4-methyl-*N*-[(1*S*)-1-(1*H*-1,2,3,4-tetrazol-5-yl)ethyl]pentanamide **14h-LL**:

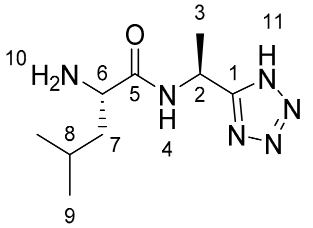

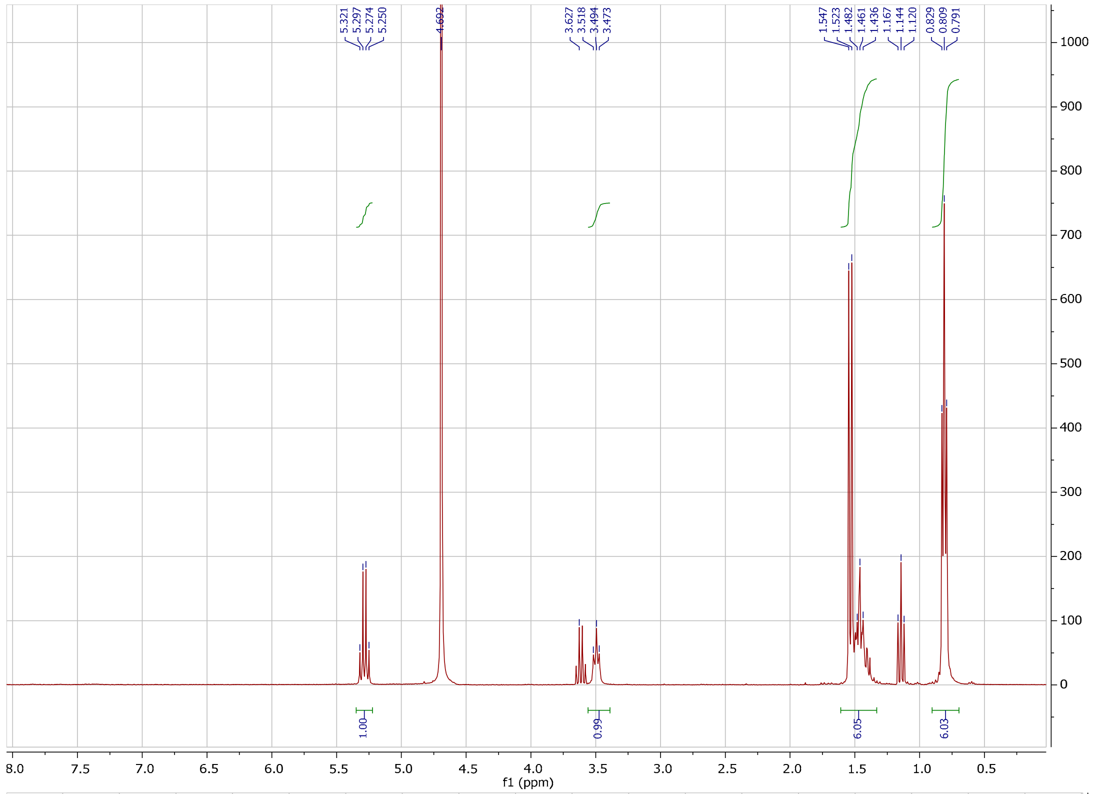

5.321
5.297
5.274
5.250
4.692
3.627
3.518
3.494
3.473
1.547
1.523
1.482
1.461
1.436
1.167
1.144
1.120
0.829
0.809
0.791
1.00
0.99
6.05
6.03
f1 (ppm)

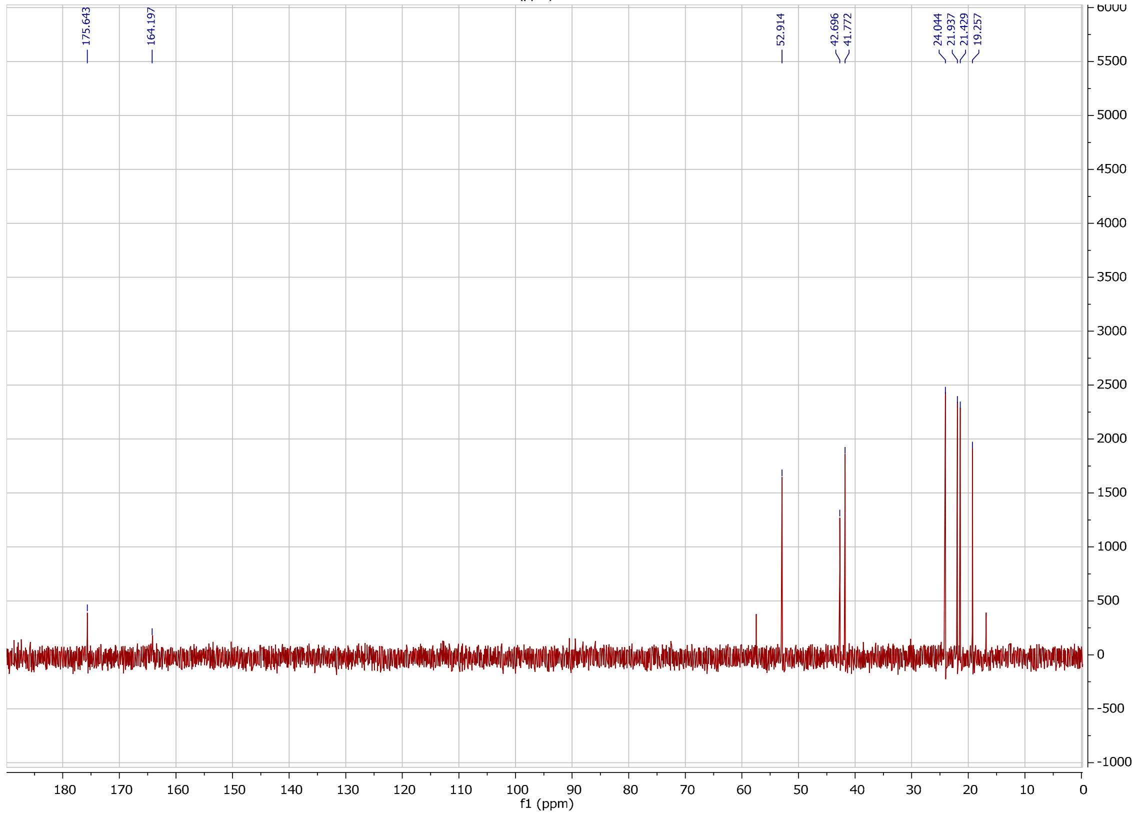

175.643
164.197
52.914
42.696
41.772
24.044
21.937
21.429
19.257
f1 (ppm)

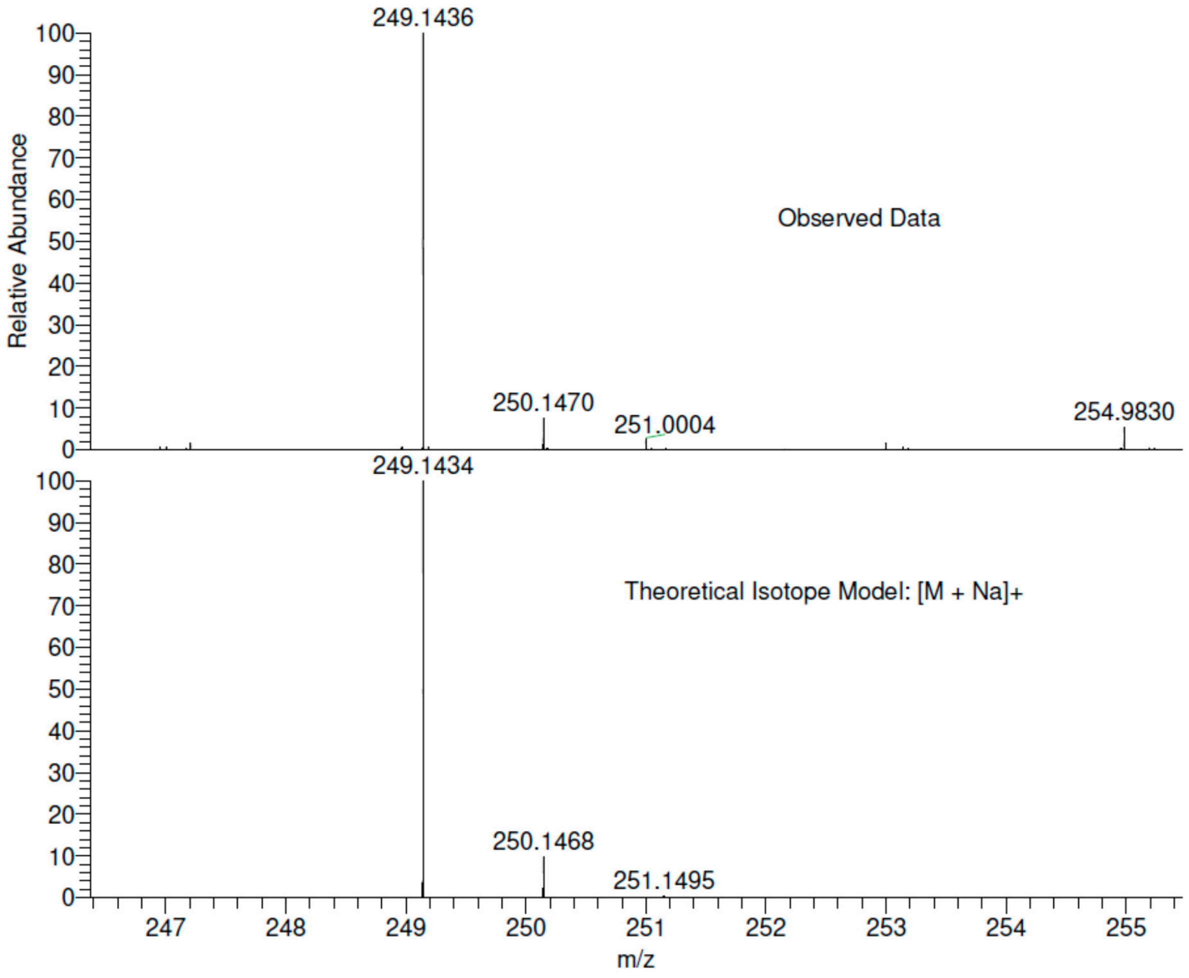
249.1436
Relative Abundance
Observed Data
250.1470
251.0004
254.9830
249.1434
Theoretical Isotope Model: [M + Na]+
250.1468
251.1495
m/z

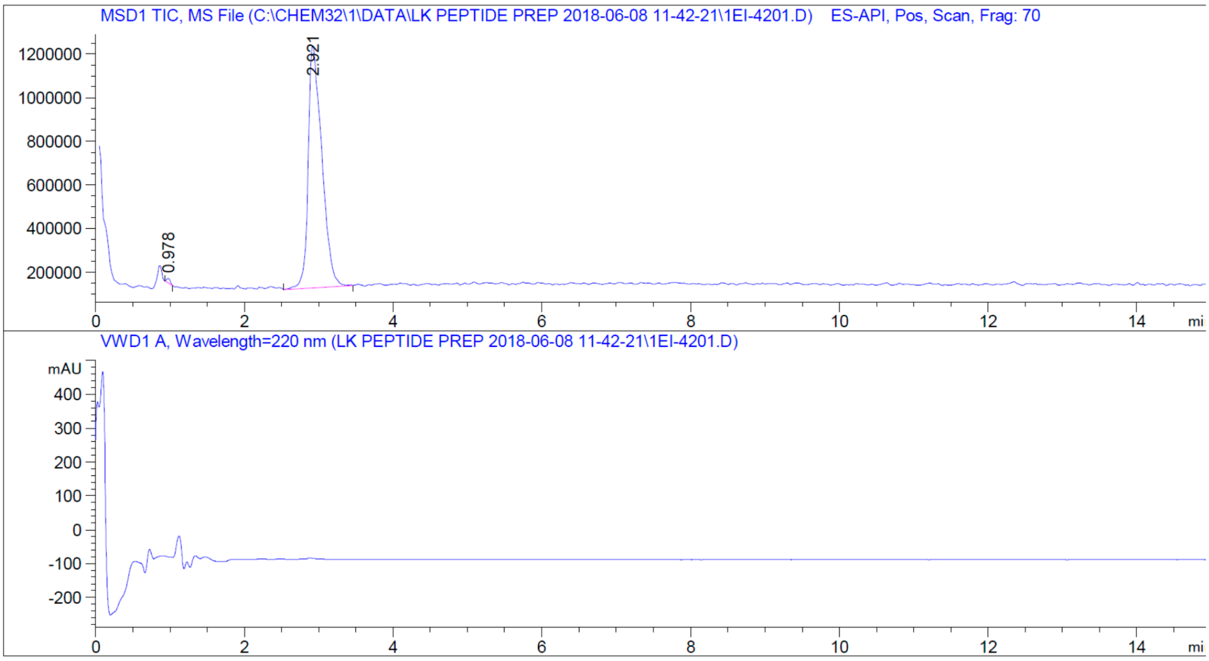

===

Area Percent Report

===

Sorted By : Signal
Multiplier: : 1.0000
Dilution: : 1.0000
Use Multiplier & Dilution Factor with ISTDs

Signal 1: MSD1 TIC, MS File

Peak #	RetTime [min]	Type	Width [min]	Area	Height	Area %
1	0.978	BB	0.0522	6.68819e4	2.18223e4	0.4687
2	2.921	BB	0.1758	1.42027e7	1.11638e6	99.5313

Totals : 1.42696e7 1.13821e6

Benzyl-*N*-[(1*S*)-3-methyl-1-{[(1*R*)-1-(1*H*-1,2,3,4-tetrazol-5-yl)ethyl]carbamoyl}butyl]carbamate **13h-LD**:

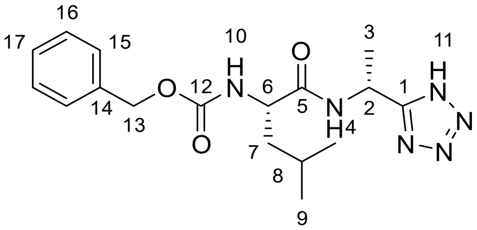

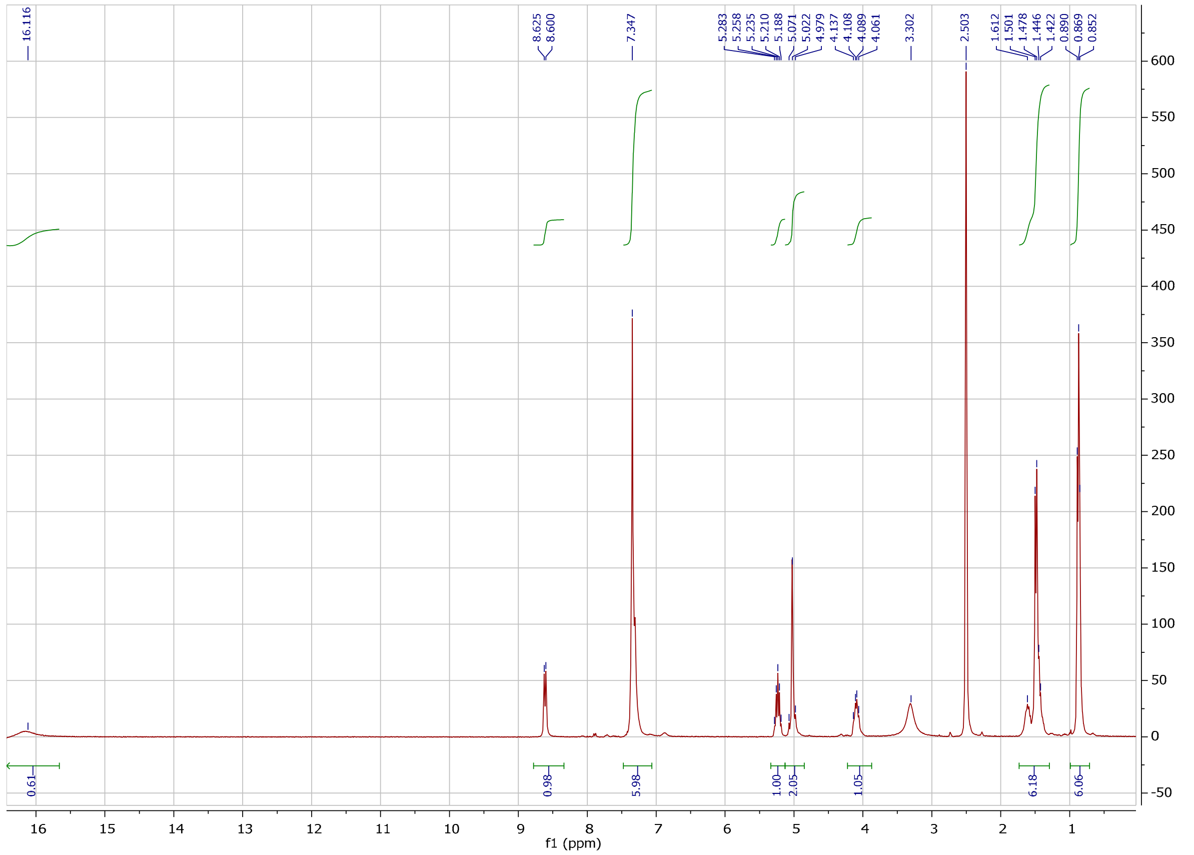
16.116
8.625
8.600
7.347
5.283
5.258
5.235
5.210
5.188
5.071
5.022
4.979
4.137
4.108
4.089
4.061
3.302
2.503
1.612
1.501
1.478
1.446
1.422
0.890
0.869
0.852
0.61
0.98
5.98
1.00
2.05
1.05
6.18
6.06
f1 (ppm)

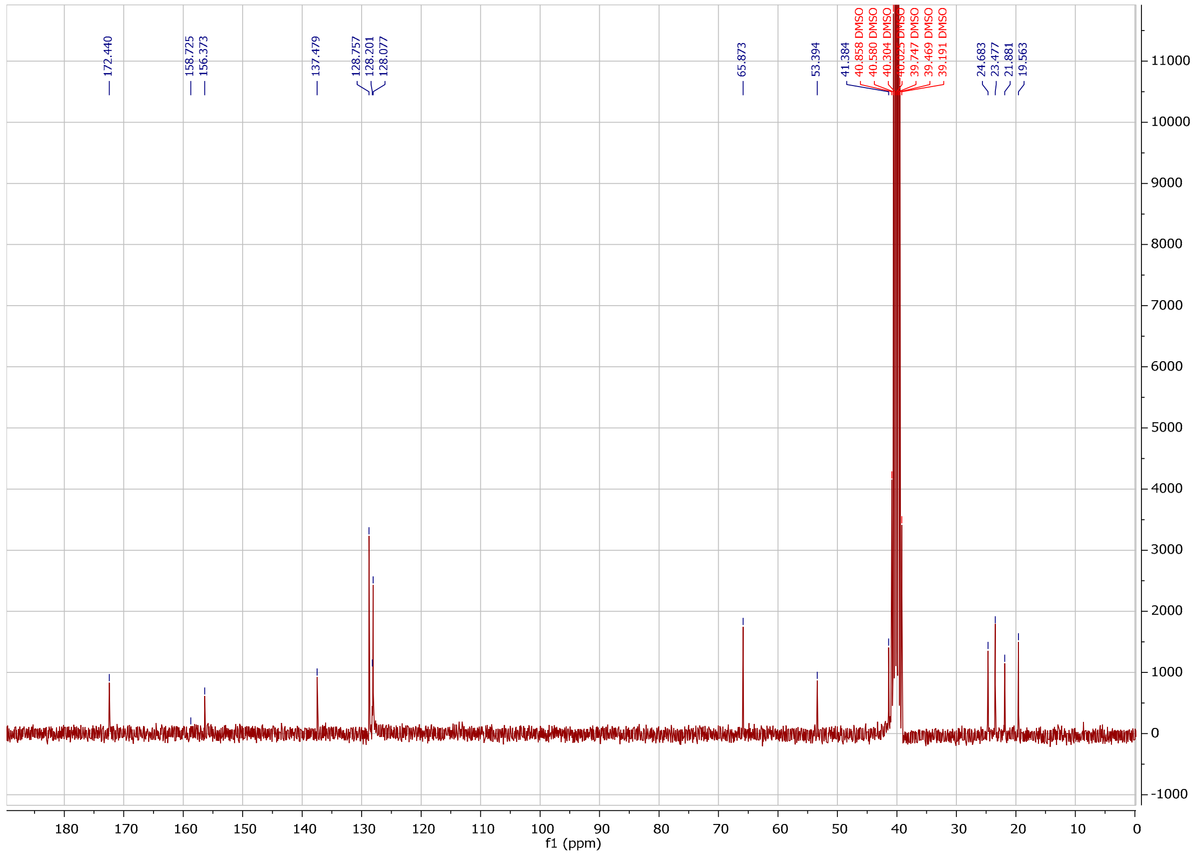
172.440
158.725
156.373
137.479
128.757
128.201
128.077
65.873
53.394
41.384
40.858 DMSO
40.580 DMSO
40.304 DMSO
40.025 DMSO
39.747 DMSO
39.469 DMSO
39.191 DMSO
24.683
23.477
21.881
19.563
f1 (ppm)

SM: 7G

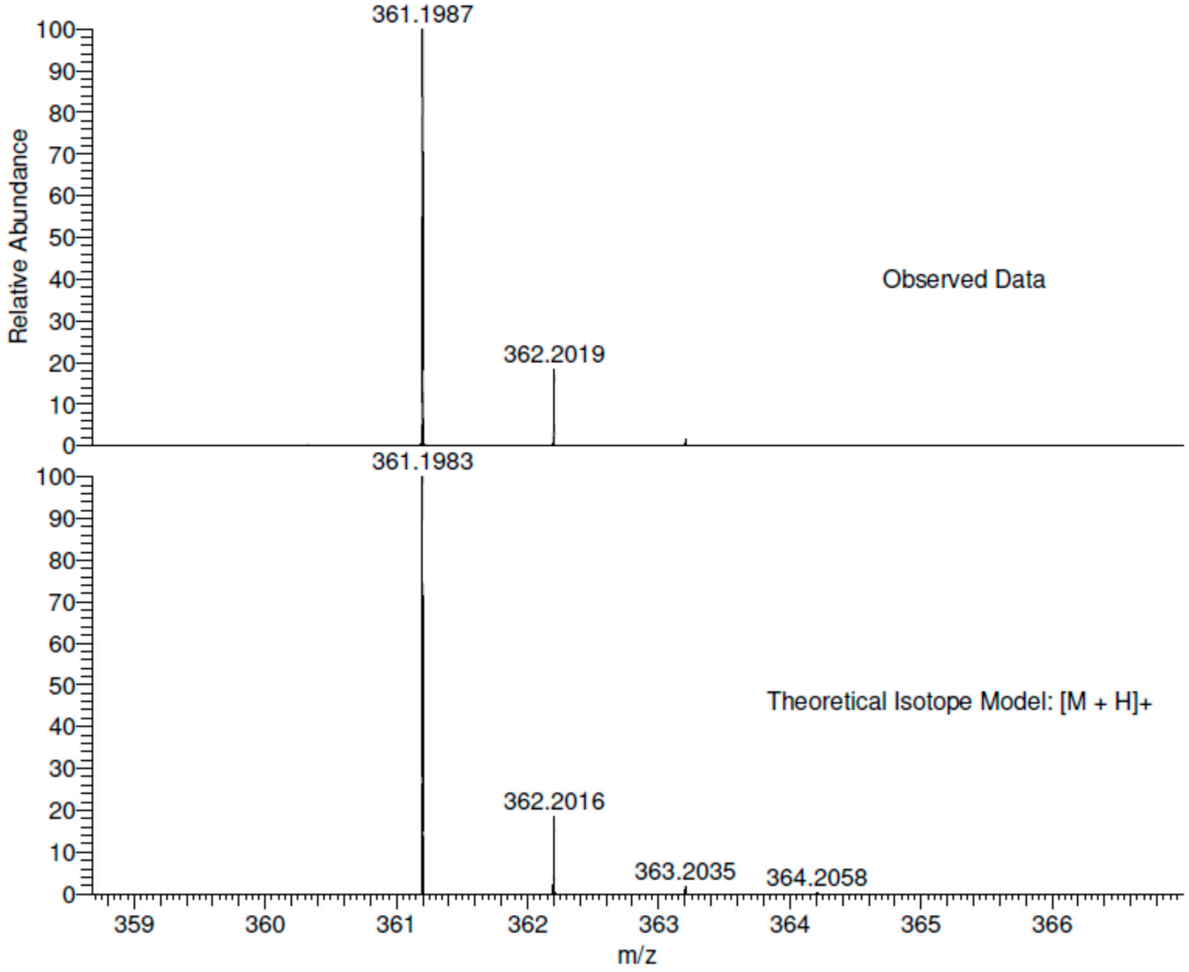

361.1987
Relative Abundance
Observed Data
362.2019
361.1983
Theoretical Isotope Model: [M + H]+
362.2016
363.2035
364.2058
m/z

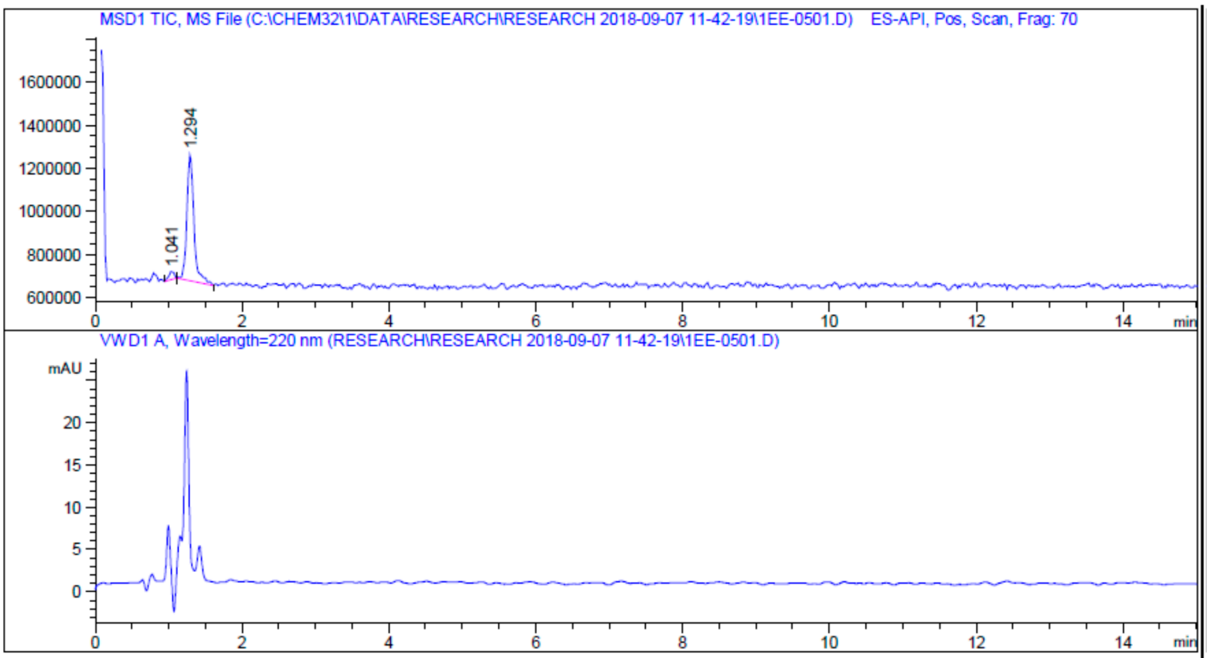


```
=====================================================================
                         Area Percent Report
=====================================================================

Sorted By             :      Signal
Multiplier:                    :      1.0000
Dilution:                      :      1.0000
Use Multiplier & Dilution Factor with ISTDs


Signal 1: MSD1 TIC, MS File

Peak RetTime Type  Width     Area      Height      Area
  #   [min]        [min]                              %
----|-------|----|-------|----------|----------|--------|
   1    1.041 BB     0.0982 1.84721e5 3.75385e4    4.5083
   2    1.294 BB     0.0992 3.91267e6 5.89957e5   95.4917

Totals :                    4.09739e6 6.27495e5
```

(2*S*)-2-Amino-4-methyl-*N*-[(1*R*)-1-(1*H*-1,2,3,4-tetrazol-5-yl)ethyl]pentanamide **14h-LD**:

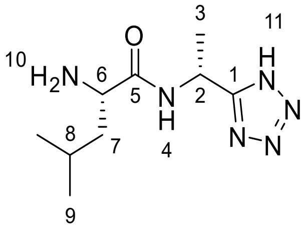

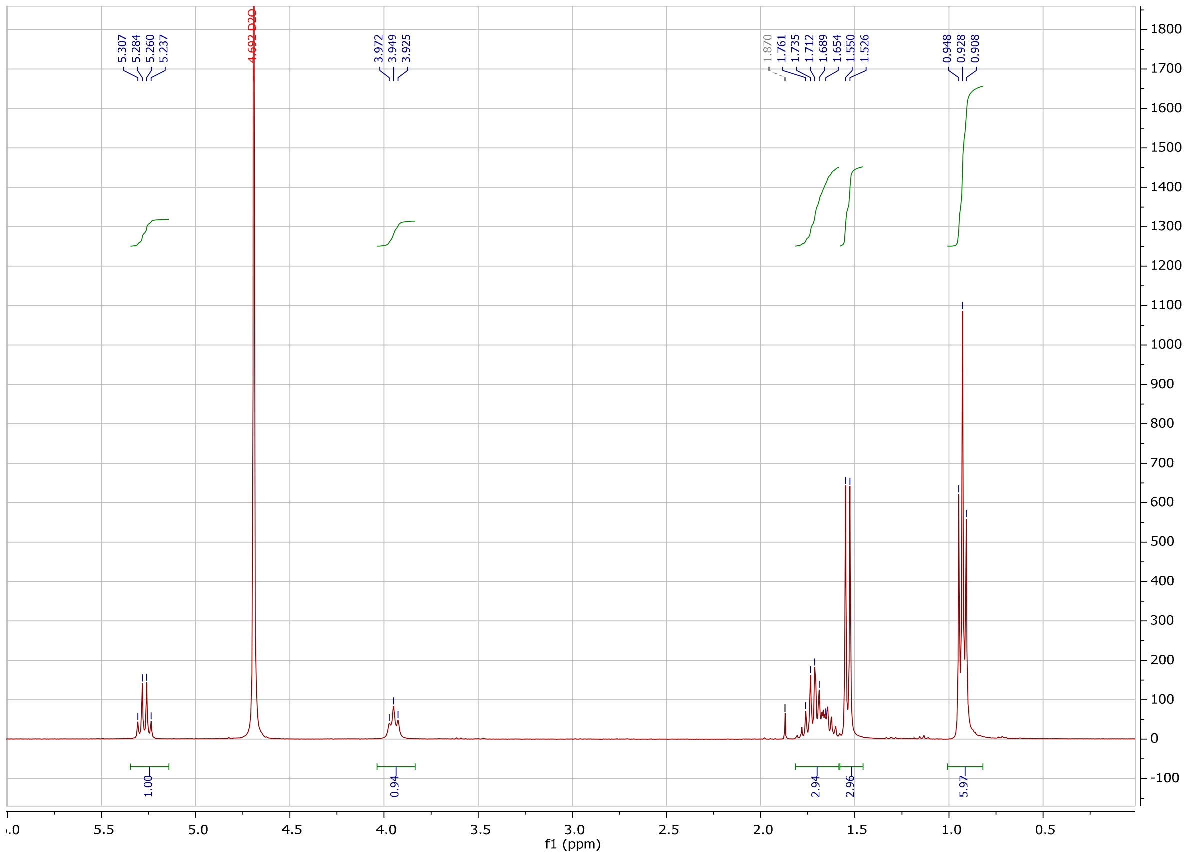

5.307
5.284
5.260
5.237
4.692 D2O
3.972
3.949
3.925
1.870
1.761
1.735
1.712
1.689
1.654
1.550
1.526
0.948
0.928
0.908
1.00
0.94
2.94
2.96
5.97
f1 (ppm)

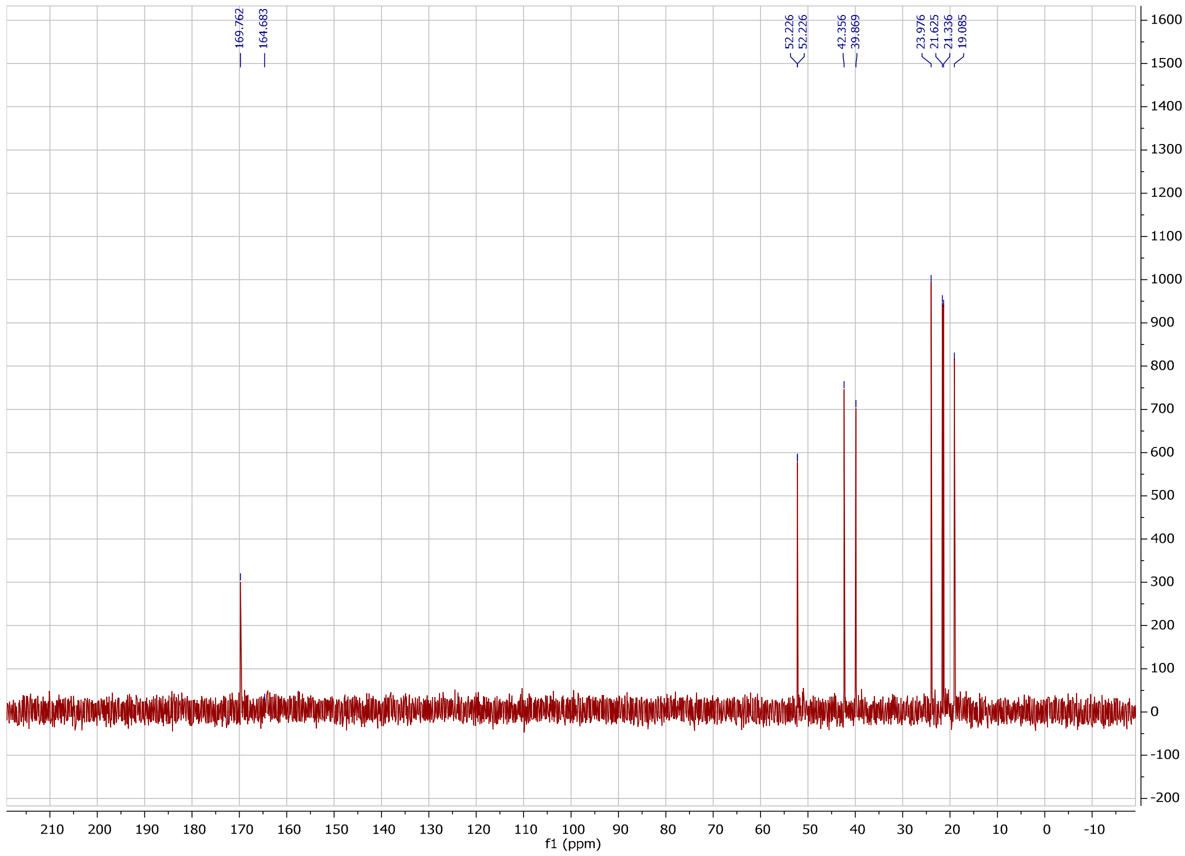

169.762
164.683
52.226
52.226
42.356
39.869
23.976
21.625
21.336
19.085
f1 (ppm)

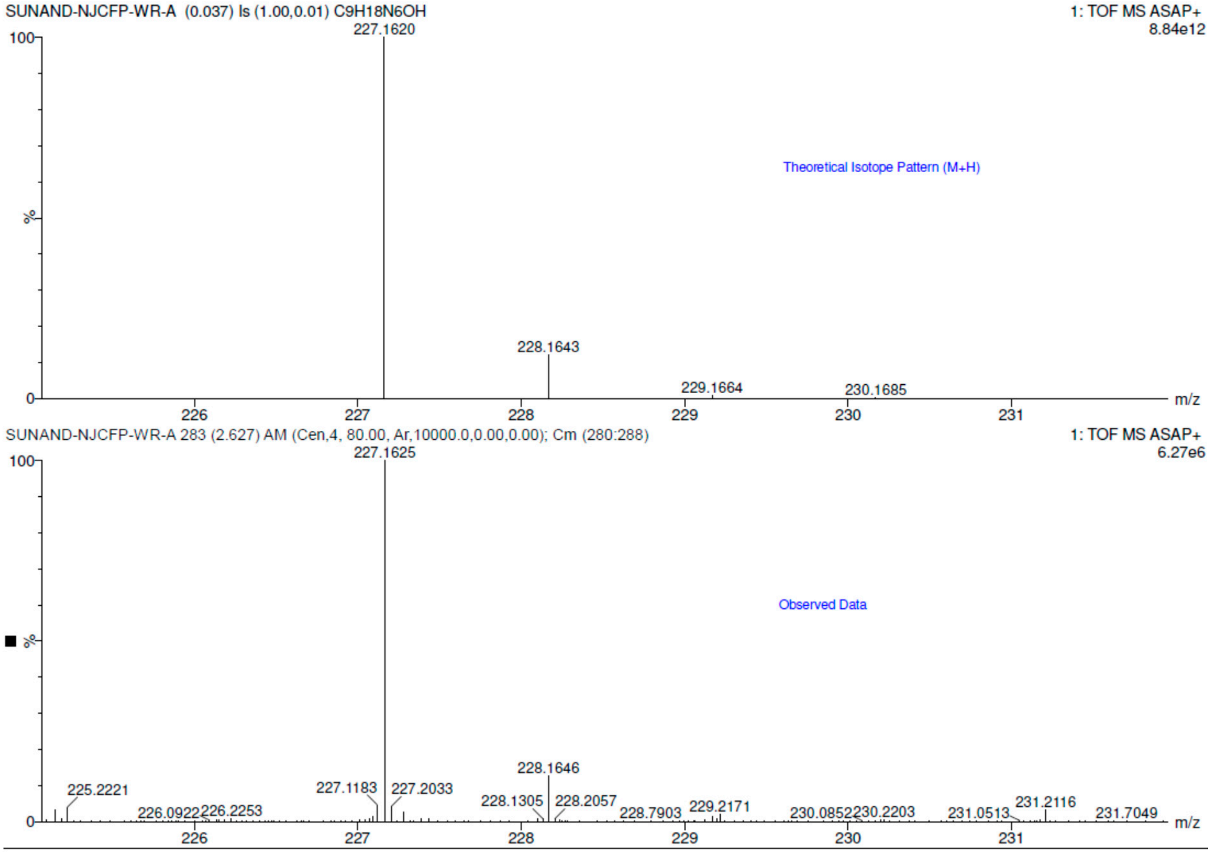

SUNAND-NJCFP-WR-A (0.037) Is (1.00,0.01) C9H18N6OH
1: TOF MS ASAP+
8.84e12
227.1620
Theoretical Isotope Pattern (M+H)
228.1643
229.1664
230.1685
m/z
SUNAND-NJCFP-WR-A 283 (2.627) AM (Cen,4, 80.00, Ar,10000.0,0.00,0.00); Cm (280:288)
1: TOF MS ASAP+
6.27e6
227.1625
Observed Data
228.1646
225.2221
227.1183
227.2033
228.1305
228.2057
226.0922
226.2253
228.7903
229.2171
230.0852
230.2203
231.0513
231.2116
231.7049

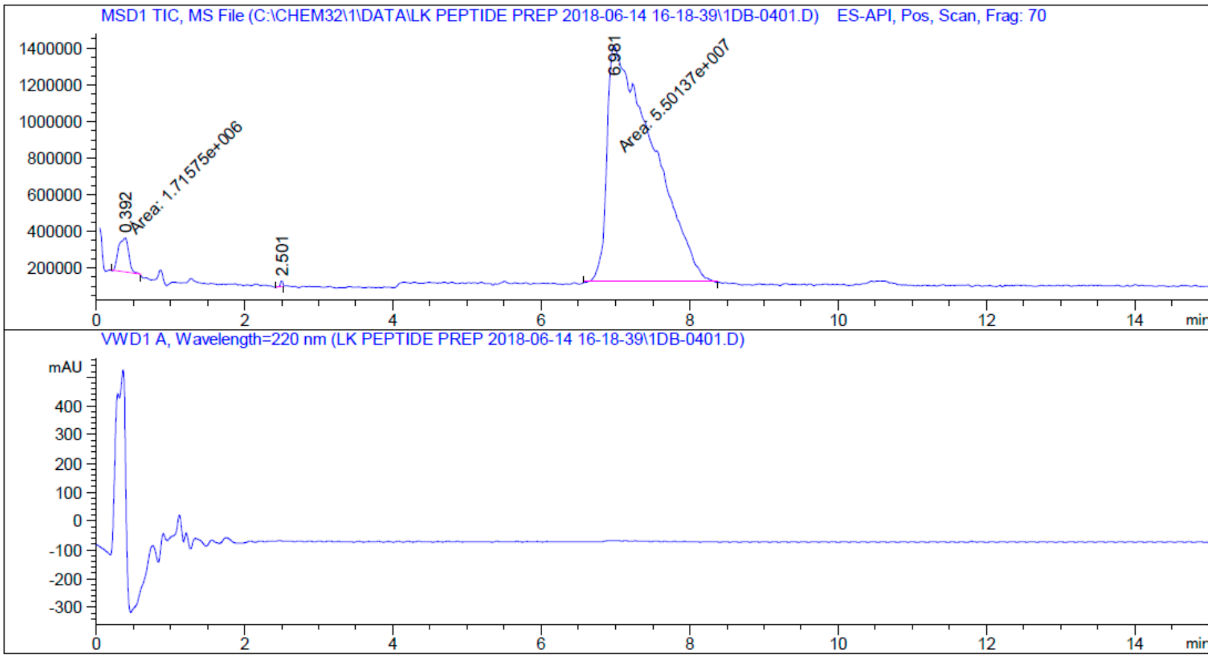

===

Area Percent Report

===

Sorted By : Signal
Multiplier: : 1.0000
Dilution: : 1.0000
Use Multiplier & Dilution Factor with ISTDs

Signal 1: MSD1 TIC, MS File

Peak #	RetTime [min]	Type	Width [min]	Area	Height	Area %
1	0.392	MM	0.1524	1.71575e6	1.87597e5	3.0210
2	2.501	BB	0.0337	6.38562e4	3.15880e4	0.1124
3	6.981	MM	0.7142	5.50137e7	1.28389e6	96.8665

Totals : 5.67933e7 1.50308e6

Benzyl-*N*-[(1*S*,2*S*)-2-methyl-1-{[(1*S*)-1-(1H-1,2,3,4-tetrazol-5-yl)ethyl]carbamoyl}butyl]carbamate **13i-LL**:

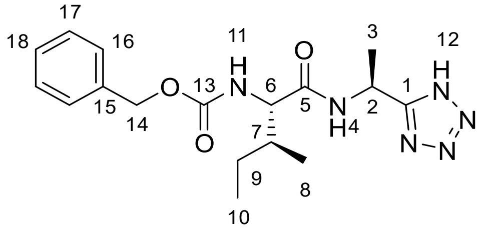

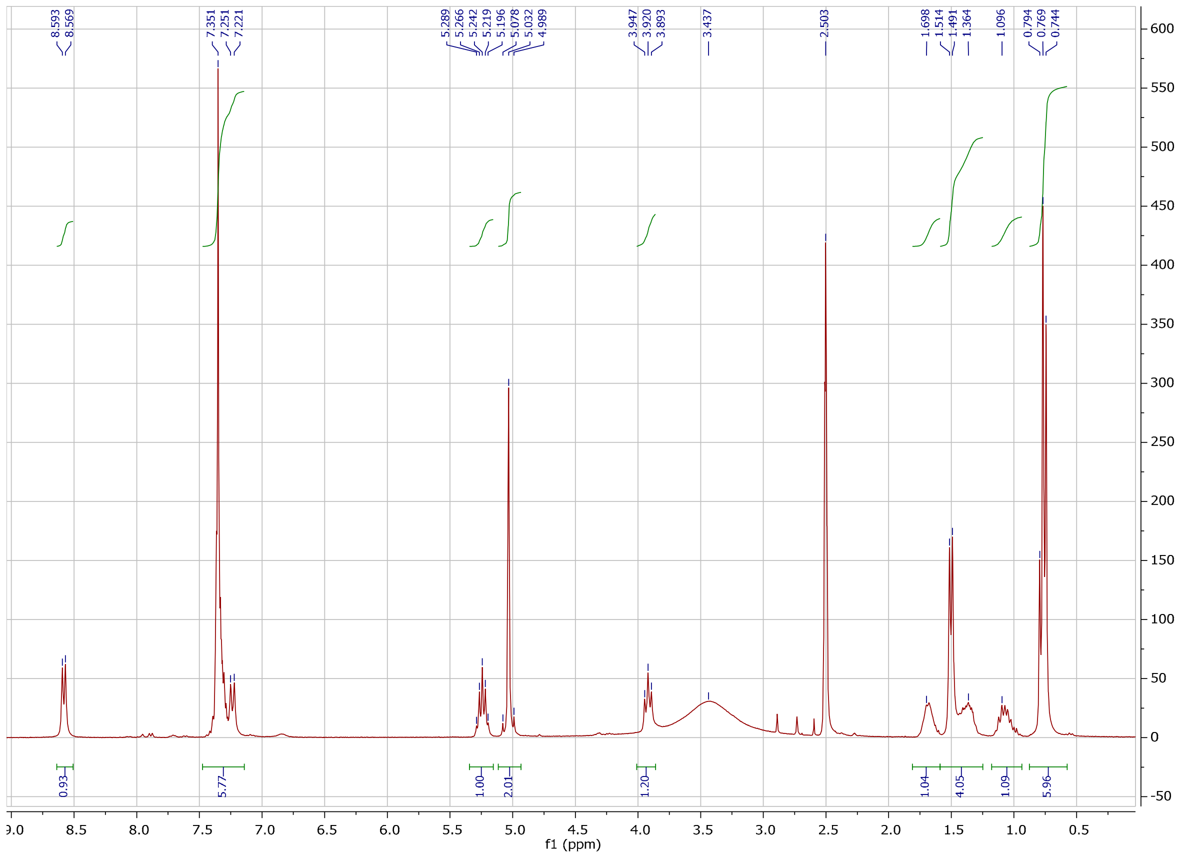

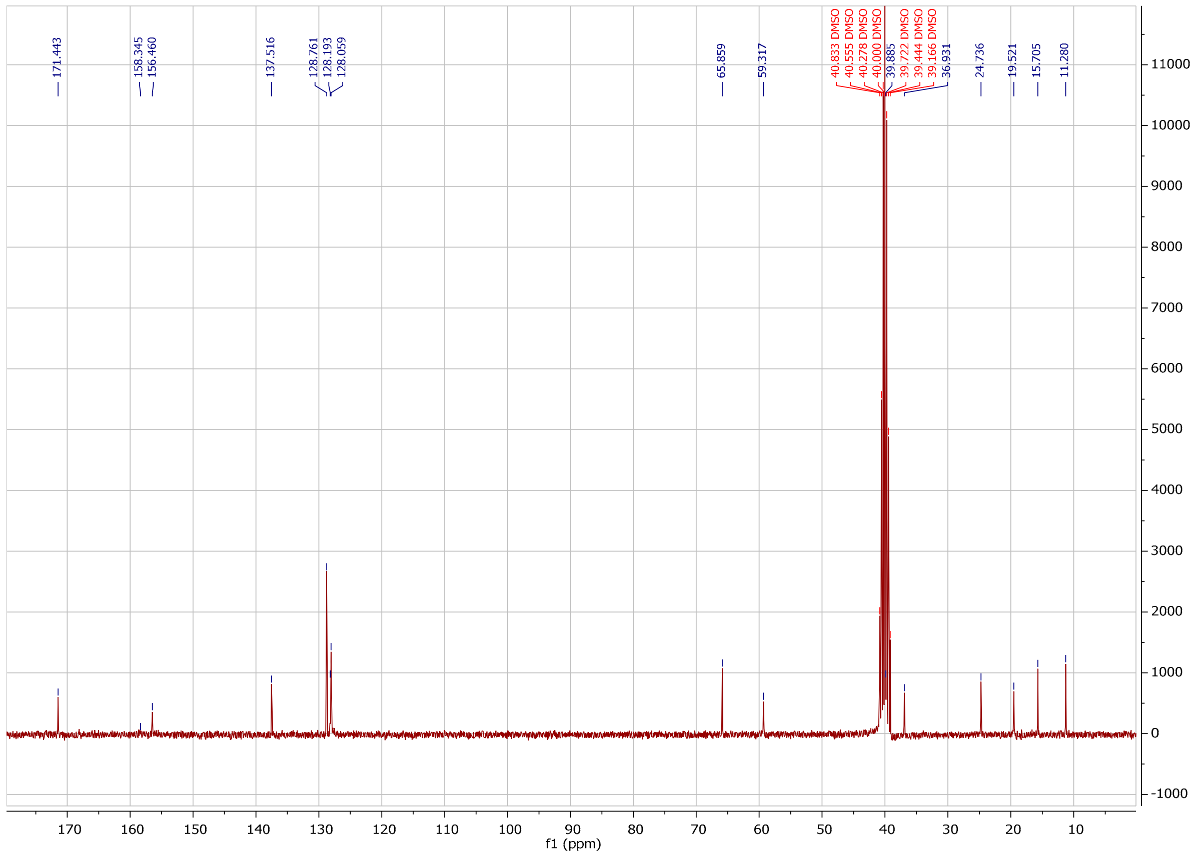

(2*S*,3*S*)-2-amino-3-methyl-*N*-[(1*S*)-1-(1*H*-1,2,3,4-tetrazol-5-yl)ethyl]pentanamide **14i-LL**

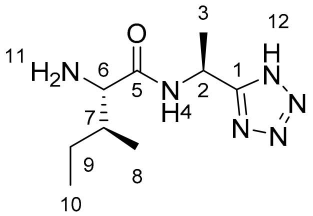

H2N
O
N
H
N
N
N
N
H
1 2 3 4 5 6 7 8 9 10 11 12

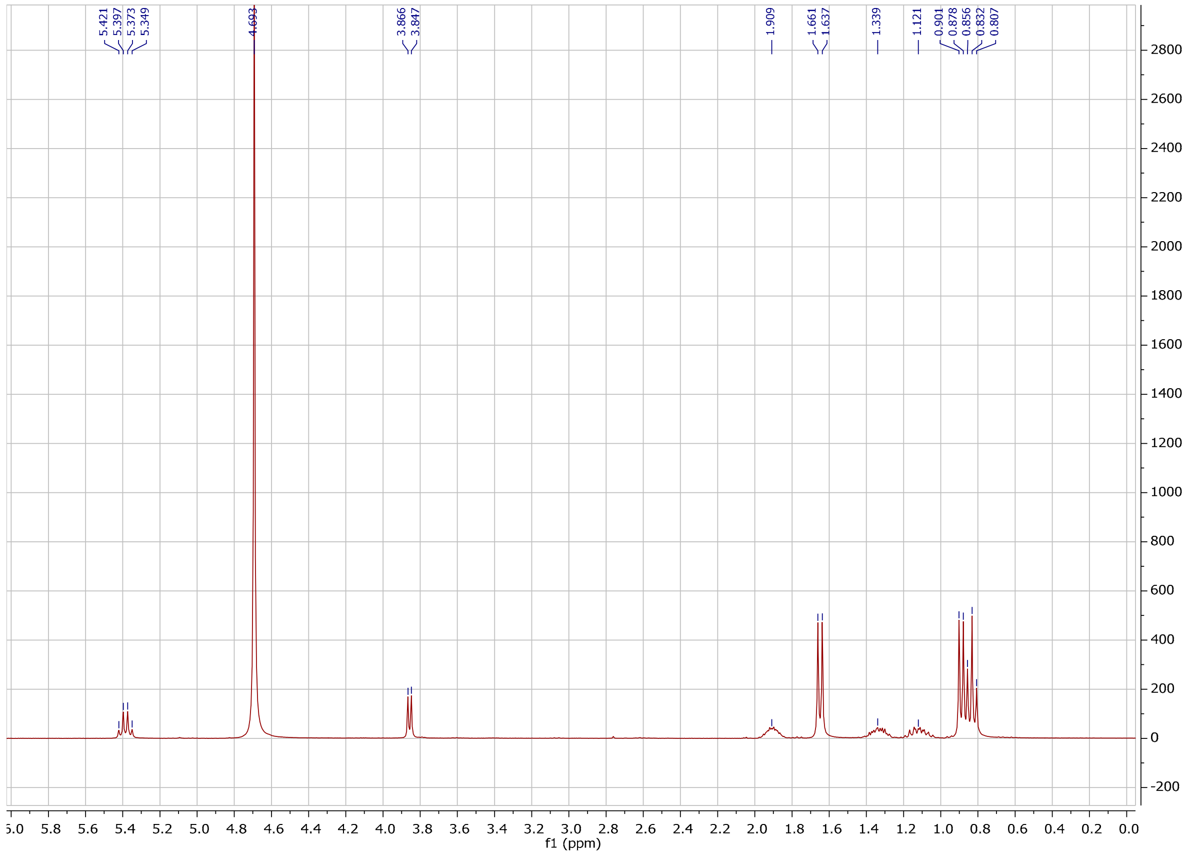

5.421
5.397
5.373
5.349
4.693
3.866
3.847
1.909
1.661
1.637
1.339
1.121
0.901
0.878
0.856
0.832
0.807
f1 (ppm)

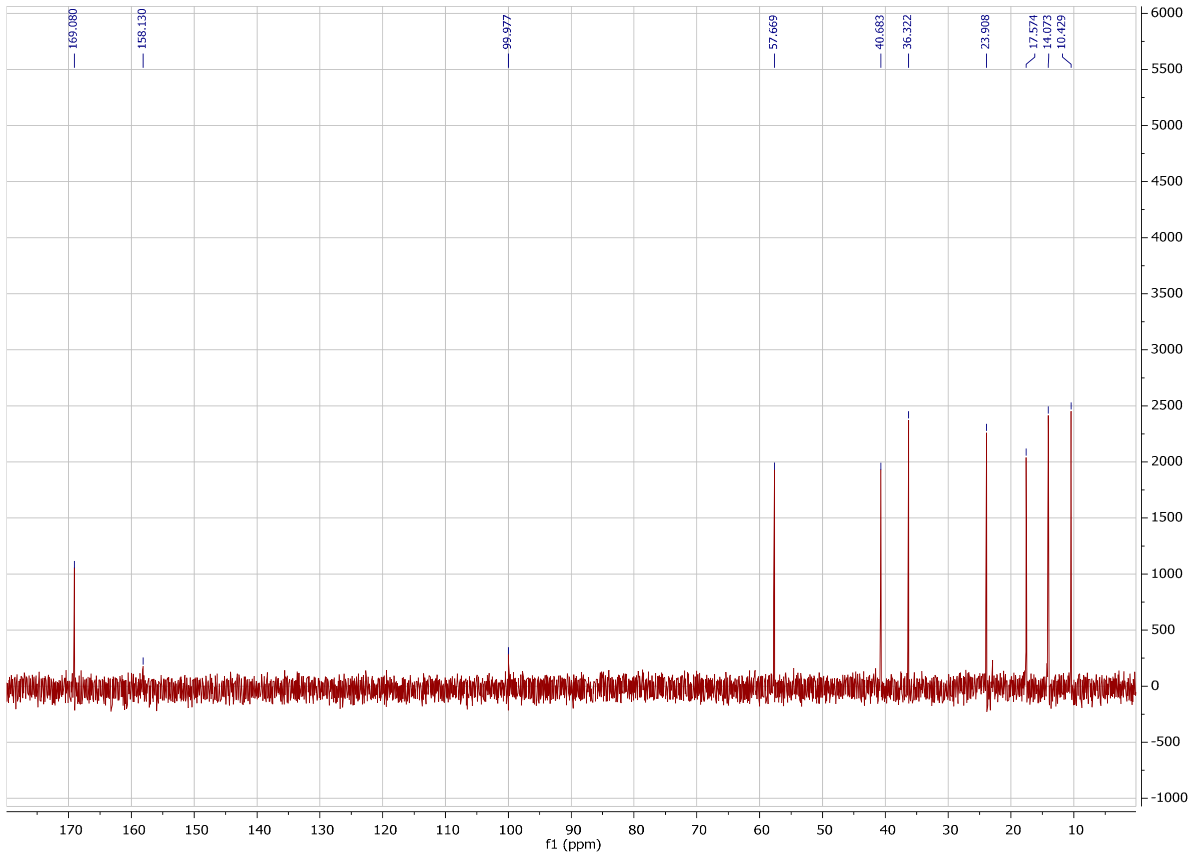
169.080
158.130
99.977
57.669
40.683
36.322
23.908
17.574
14.073
10.429
f1 (ppm)

SM: 7G

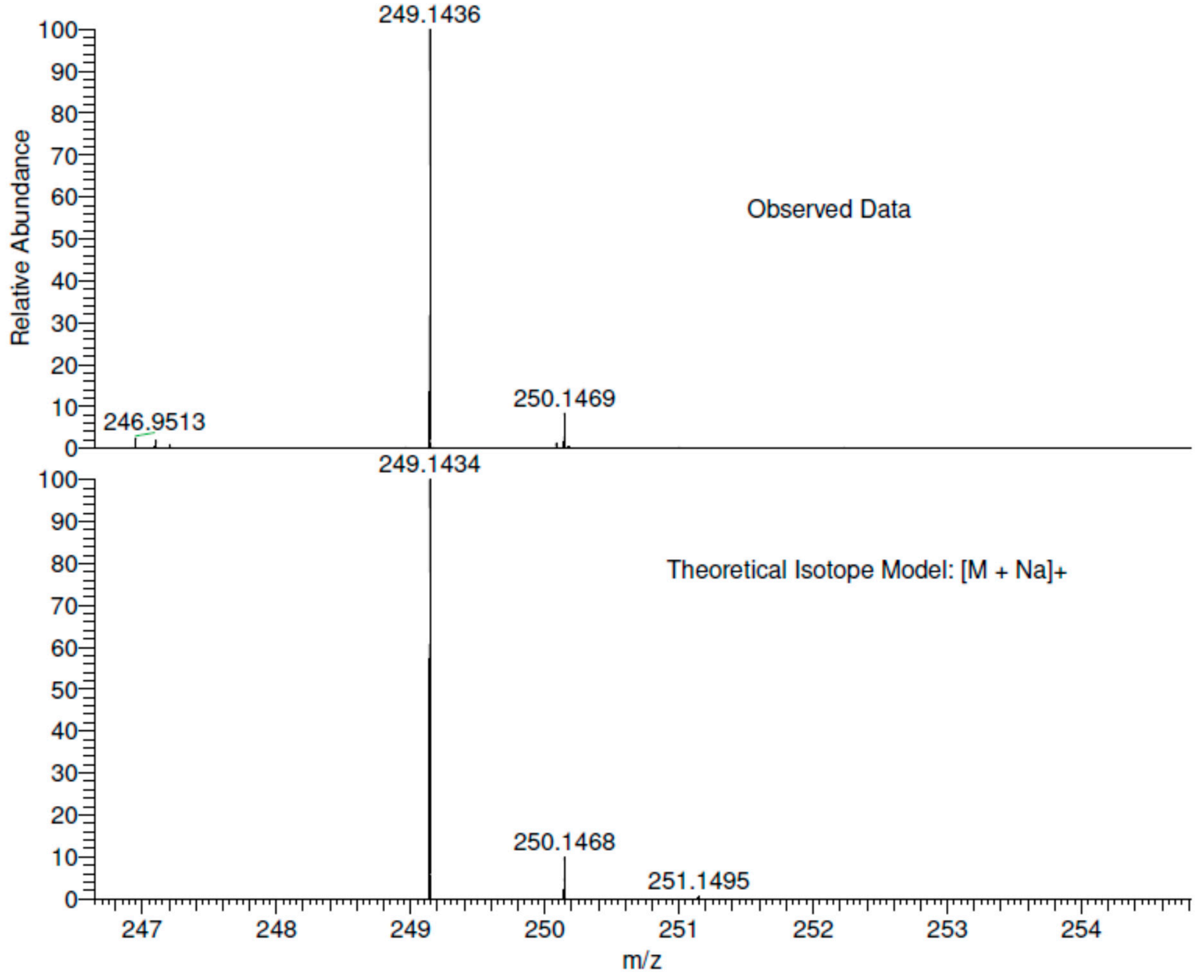

249.1436
Relative Abundance
Observed Data
250.1469
246.9513
249.1434
Theoretical Isotope Model: [M + Na]+
250.1468
251.1495
m/z

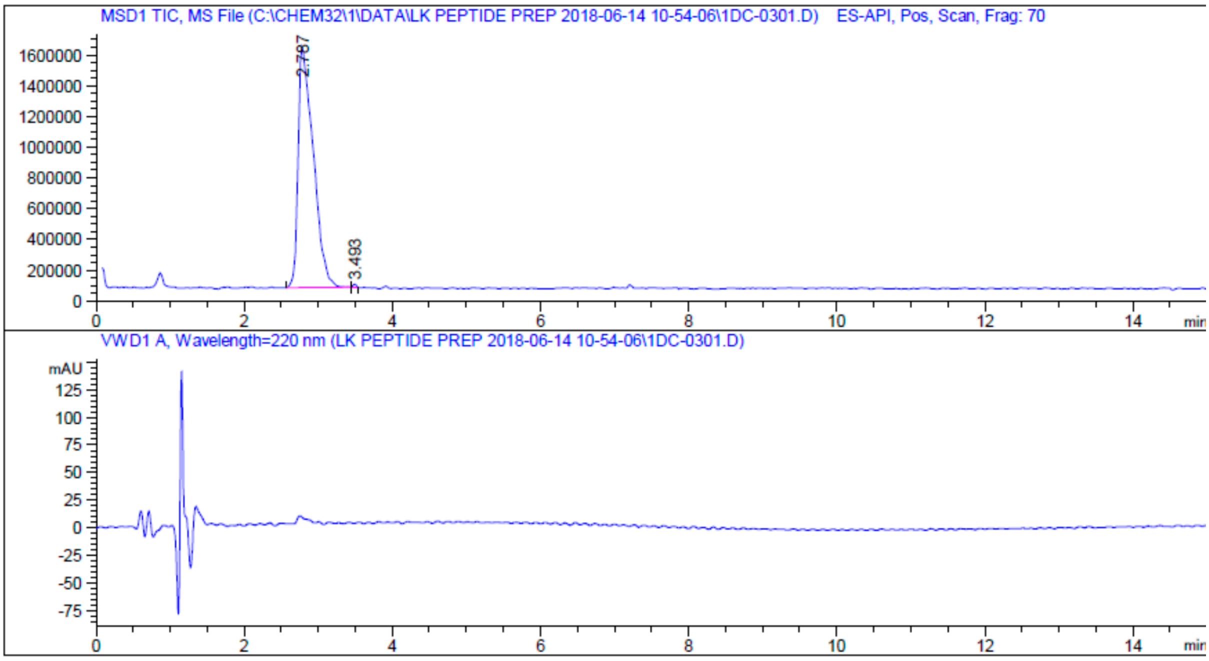


```
=====================================================================
                         Area Percent Report
=====================================================================

Sorted By             :      Signal
Multiplier:                  :       1.0000
Dilution:                    :       1.0000
Use Multiplier & Dilution Factor with ISTDs


Signal 1: MSD1 TIC, MS File

Peak RetTime Type  Width      Area       Height      Area
  #    [min]        [min]                              %
----|-------|----|-------|----------|----------|--------|
   1    2.787 BB    0.1981 2.22201e7  1.56789e6   99.6797
   2    3.493 BB    0.0522 7.13974e4  2.32827e4    0.3203

Totals :                   2.22915e7  1.59117e6
```

Benzyl-(5S)-2-oxo-5-{[(1S)-1-(1H-1,2,3,4-tetrazol-5-yl)ethyl]carbamoyl}pyrrolidine-1-carboxylate **13f-LL**:

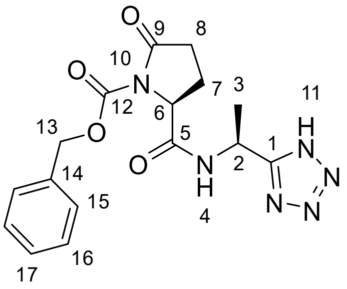

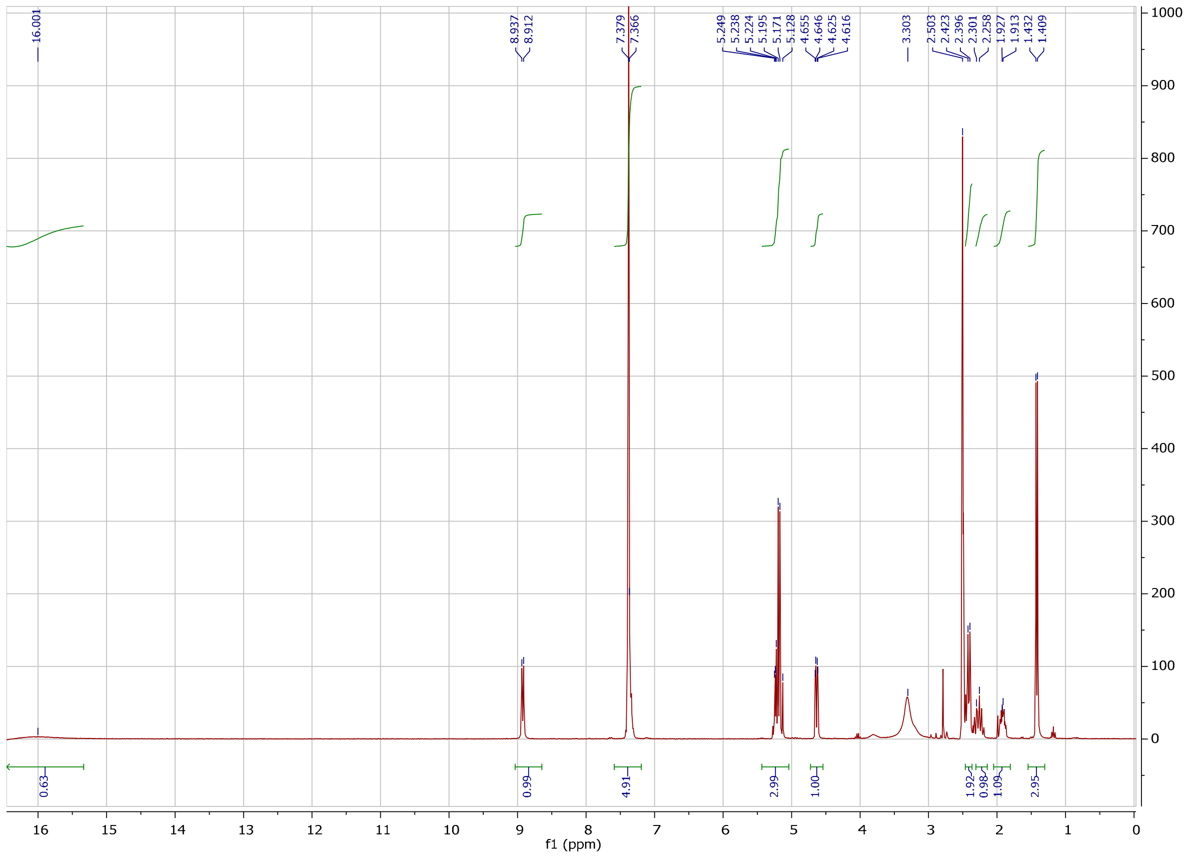
16.001
8.937
8.912
7.379
7.366
5.249
5.238
5.224
5.195
5.171
5.128
4.655
4.646
4.625
4.616
3.303
2.503
2.423
2.396
2.301
2.258
1.927
1.913
1.432
1.409
0.63
0.99
4.91
2.99
1.00
1.92
0.98
1.09
2.95
f1 (ppm)

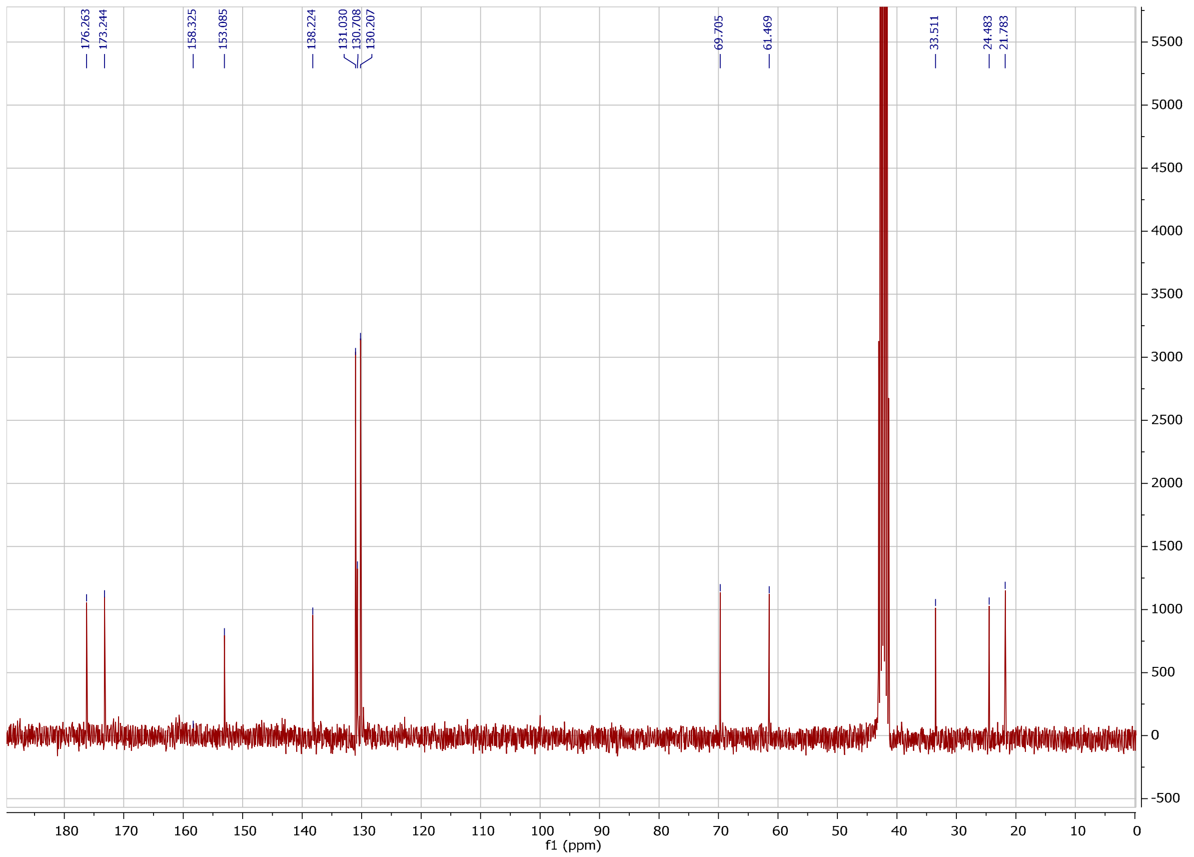
176.263
173.244
158.325
153.085
138.224
131.030
130.708
130.207
69.705
61.469
33.511
24.483
21.783
f1 (ppm)

(*S*)-*N*-((*S*)-1-(1*H*-tetrazol-5-yl)ethyl)-5-oxopyrrolidine-2-carboxamide **14f-LL**:

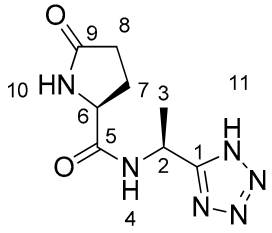

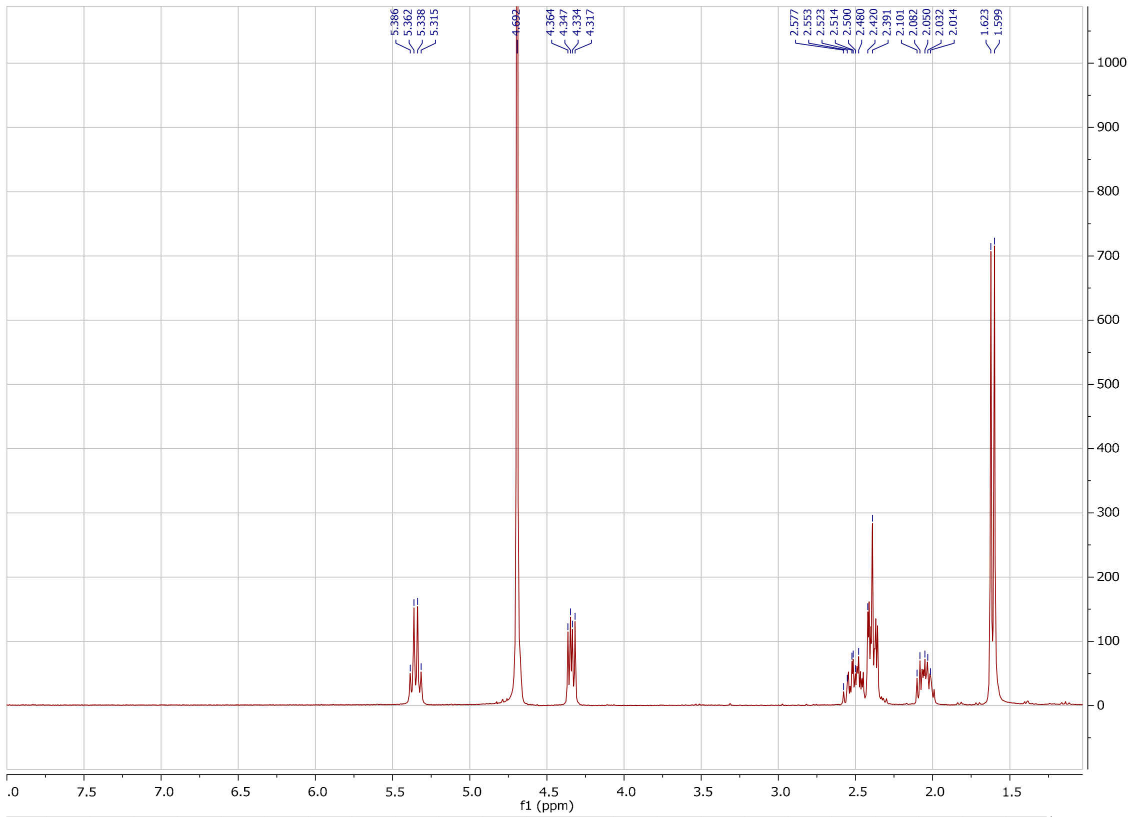
5.386
5.362
5.338
5.315
4.692
4.364
4.347
4.334
4.317
2.577
2.553
2.523
2.514
2.500
2.480
2.420
2.391
2.101
2.082
2.050
2.032
2.014
1.623
1.599
f1 (ppm)

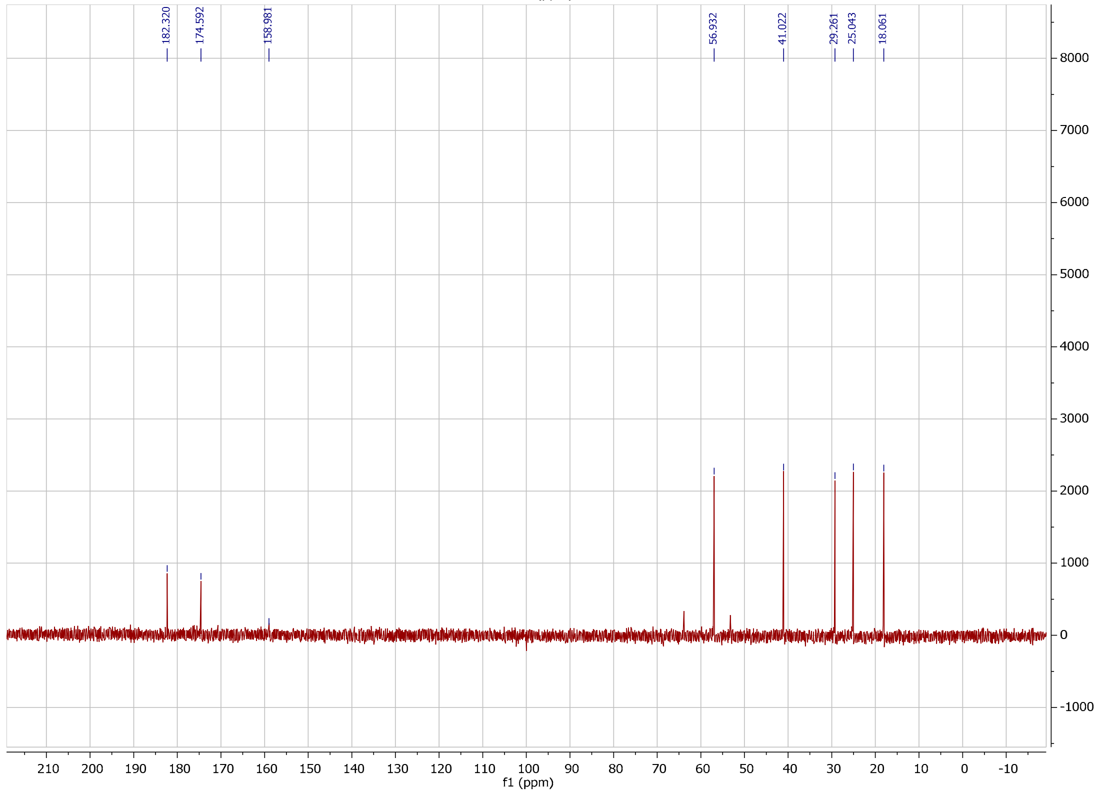
182.320
174.592
158.981
56.932
41.022
29.261
25.043
18.061
f1 (ppm)

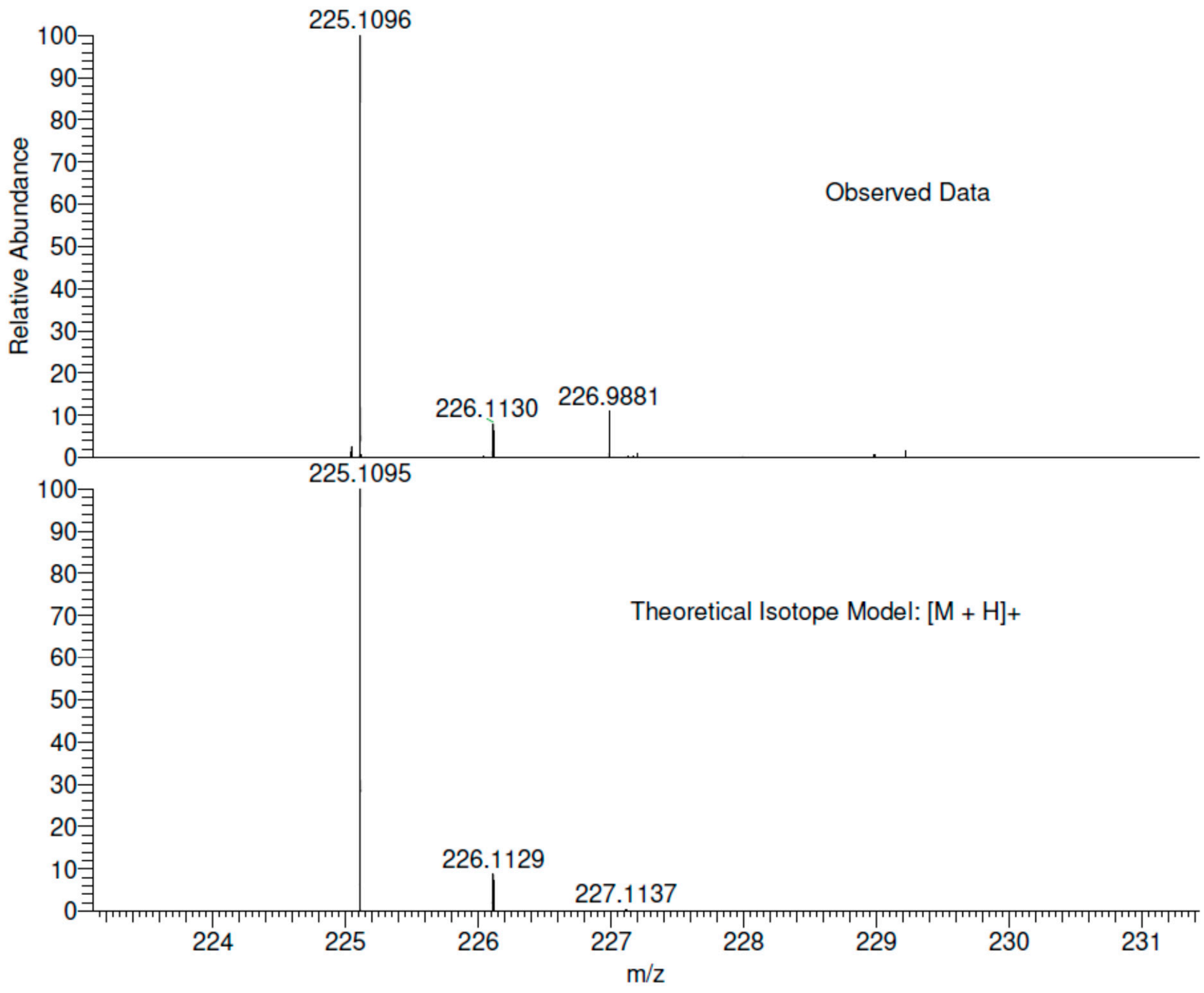
225.1096
Observed Data
226.1130
226.9881
225.1095
Theoretical Isotope Model: [M + H]+
226.1129
227.1137
Relative Abundance
m/z
100
90
80
70
60
50
40
30
20
10
0
224
225
226
227
228
229
230
231

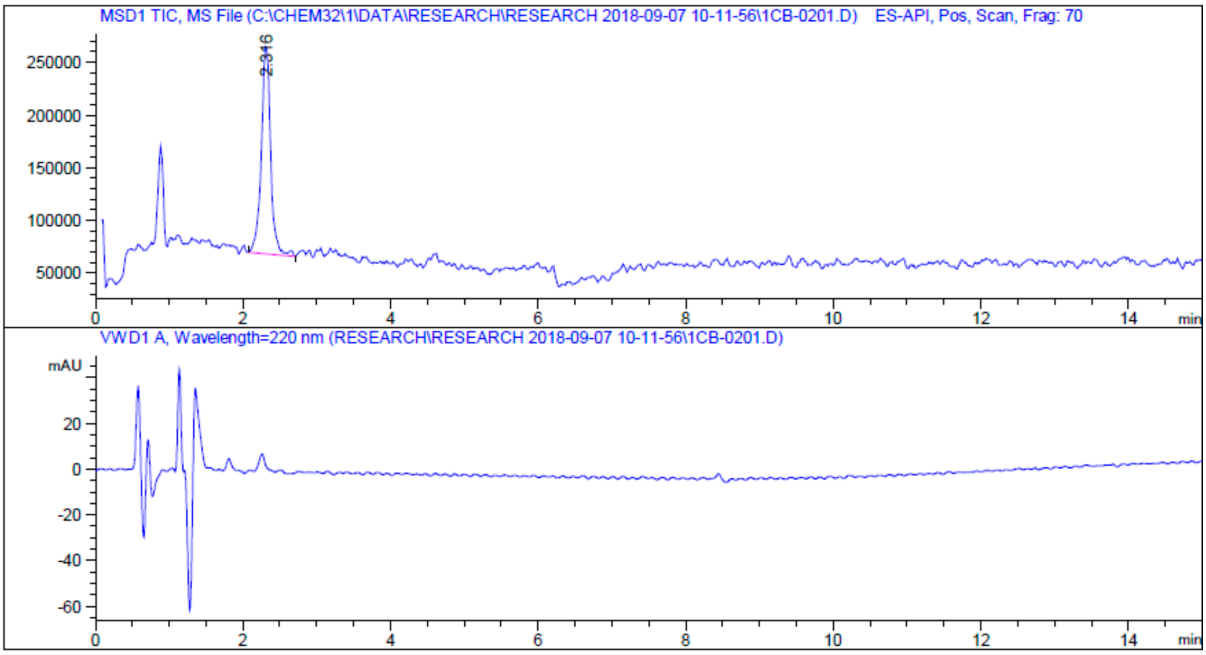


```
=====================================================================
                         Area Percent Report
=====================================================================

Sorted By             :      Signal
Multiplier:                   :      1.0000
Dilution:                     :      1.0000
Use Multiplier & Dilution Factor with ISTDs


Signal 1: MSD1 TIC, MS File

Peak RetTime Type  Width     Area      Height     Area
  #   [min]        [min]                           %
----|-------|----|-------|----------|----------|--------|
   1   2.316 BB    0.1342 1.72300e6  1.98295e5  100.0000

Totals :                  1.72300e6  1.98295e5
```

Blank for **16f-LL**:

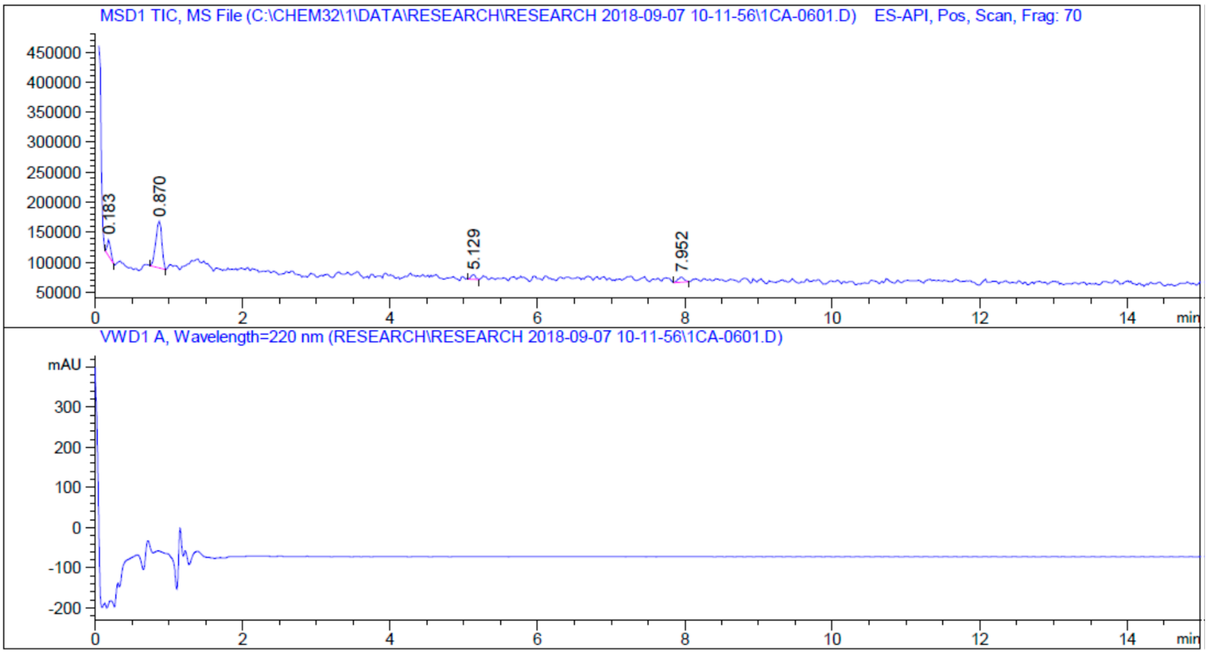

Benzyl-*N*-[(1*S*)-2-phenyl-1-{[(1*S*)-1-(1*H*-1,2,3,4-tetrazol-5-yl)ethyl]carbamoyl}ethyl]carbamate **13j-LL**:

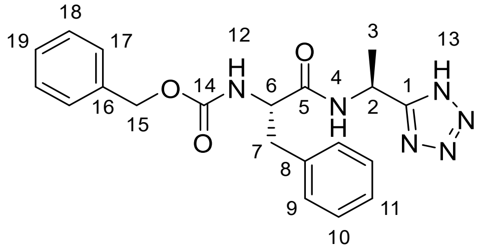

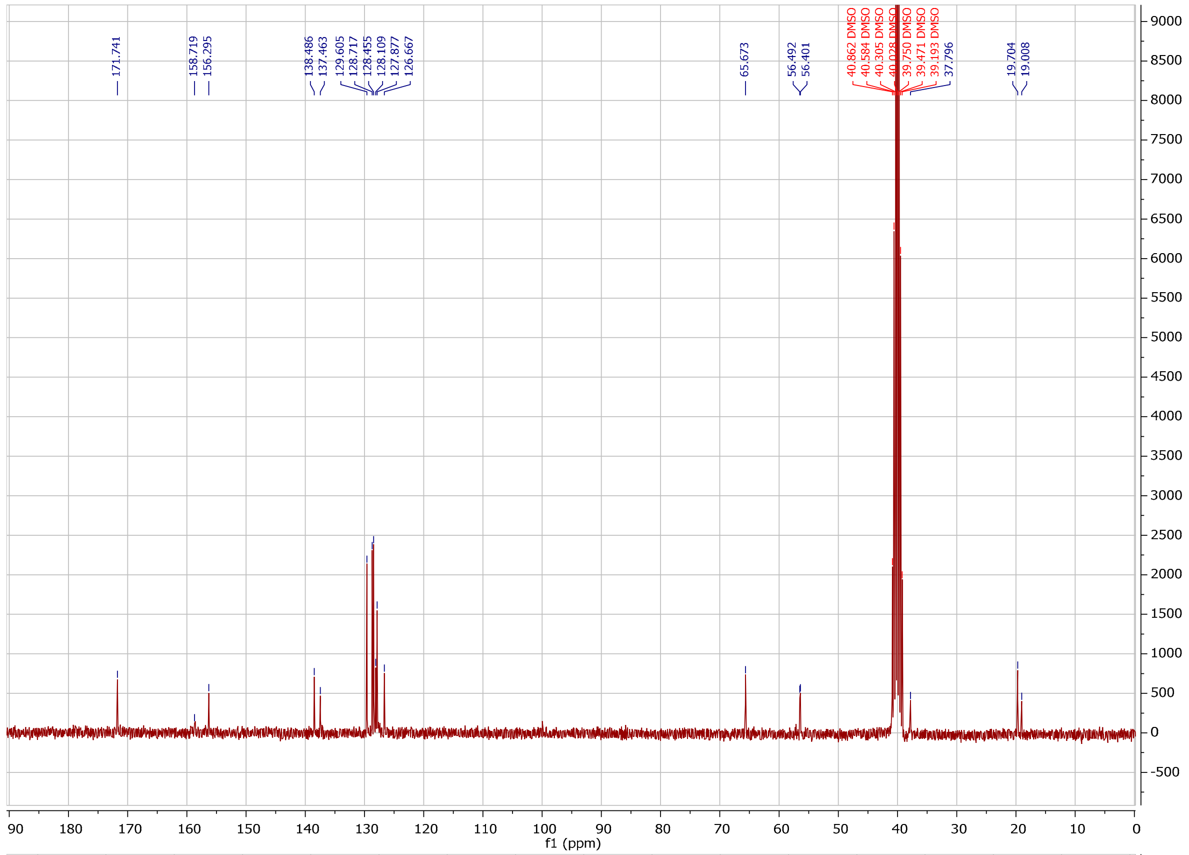

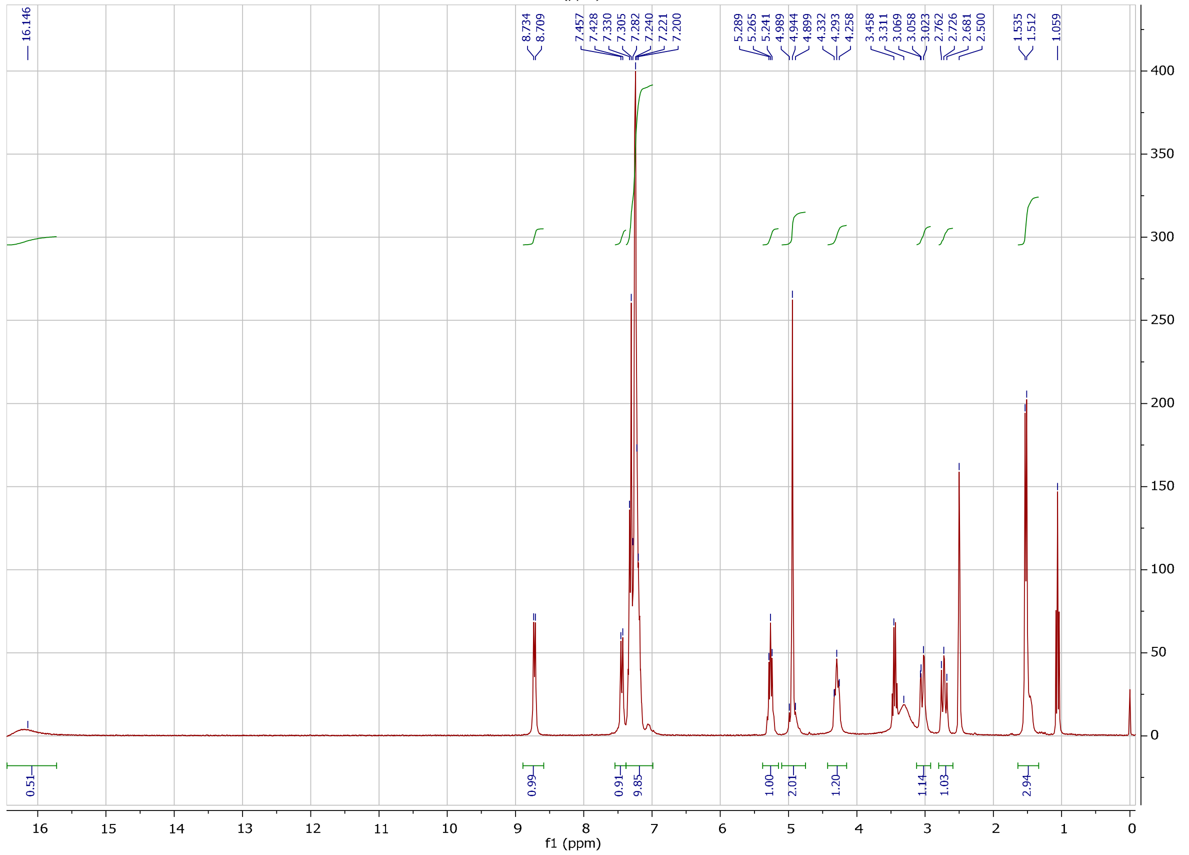

(2*S*)-2-Amino-3-phenyl-*N*-[(1*S*)-1-(1*H*-1,2,3,4-tetrazol-5-yl)ethyl]propanamide **14j-LL**:

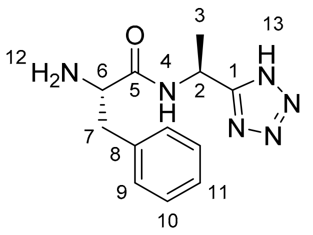

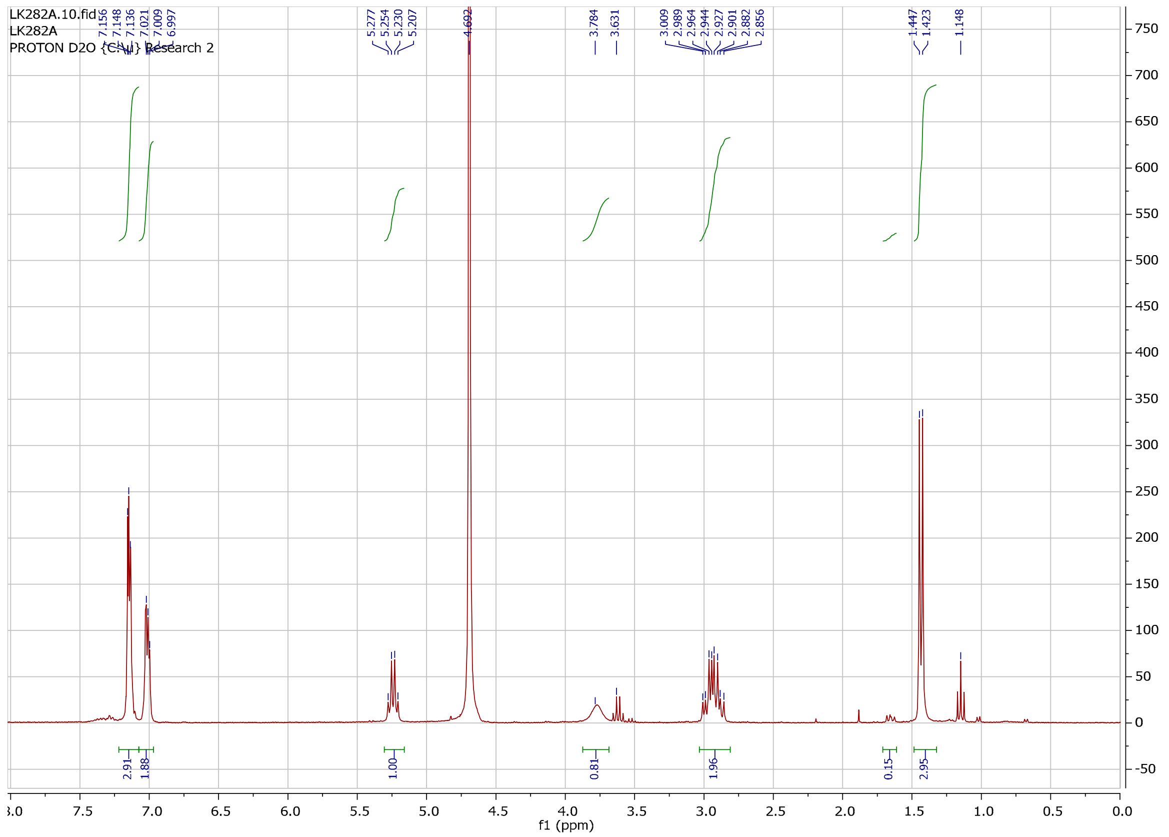
LK282A.10.fid
LK282A
PROTON D2O
f1 (ppm)

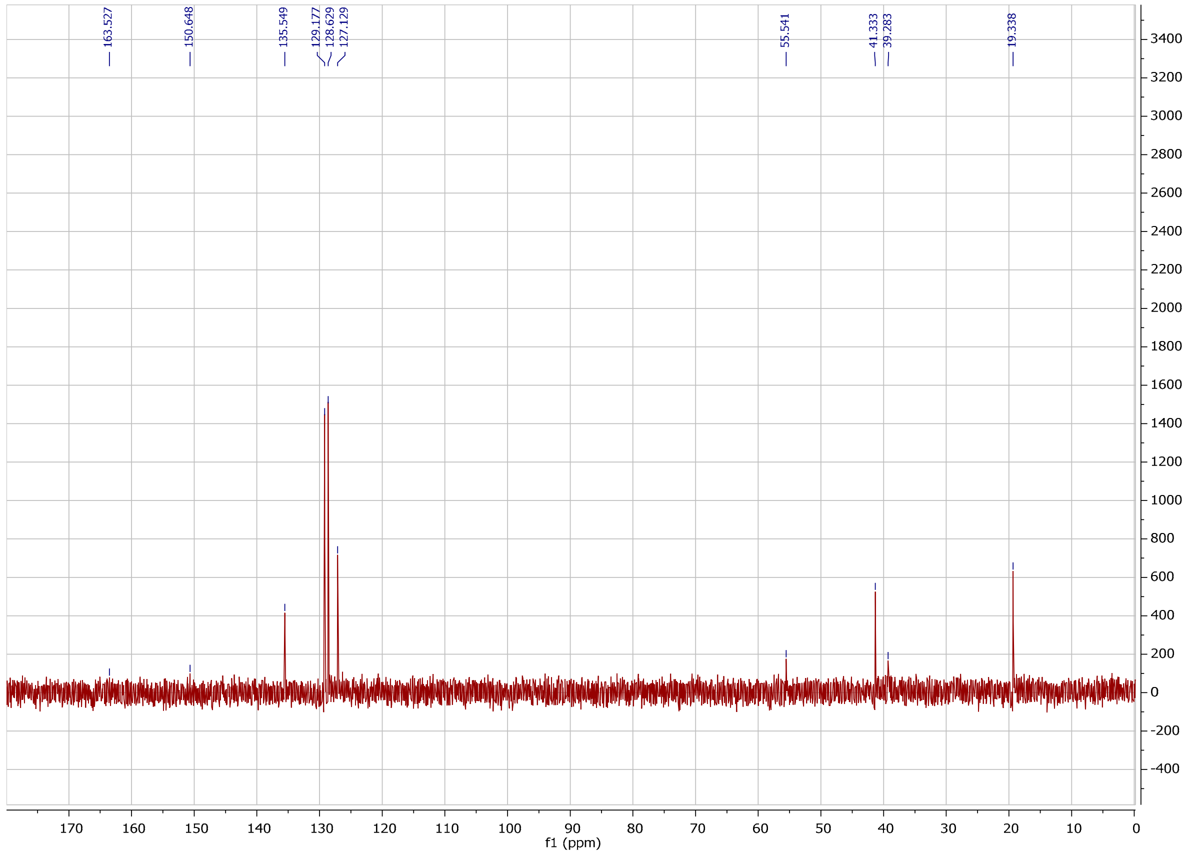
f1 (ppm)

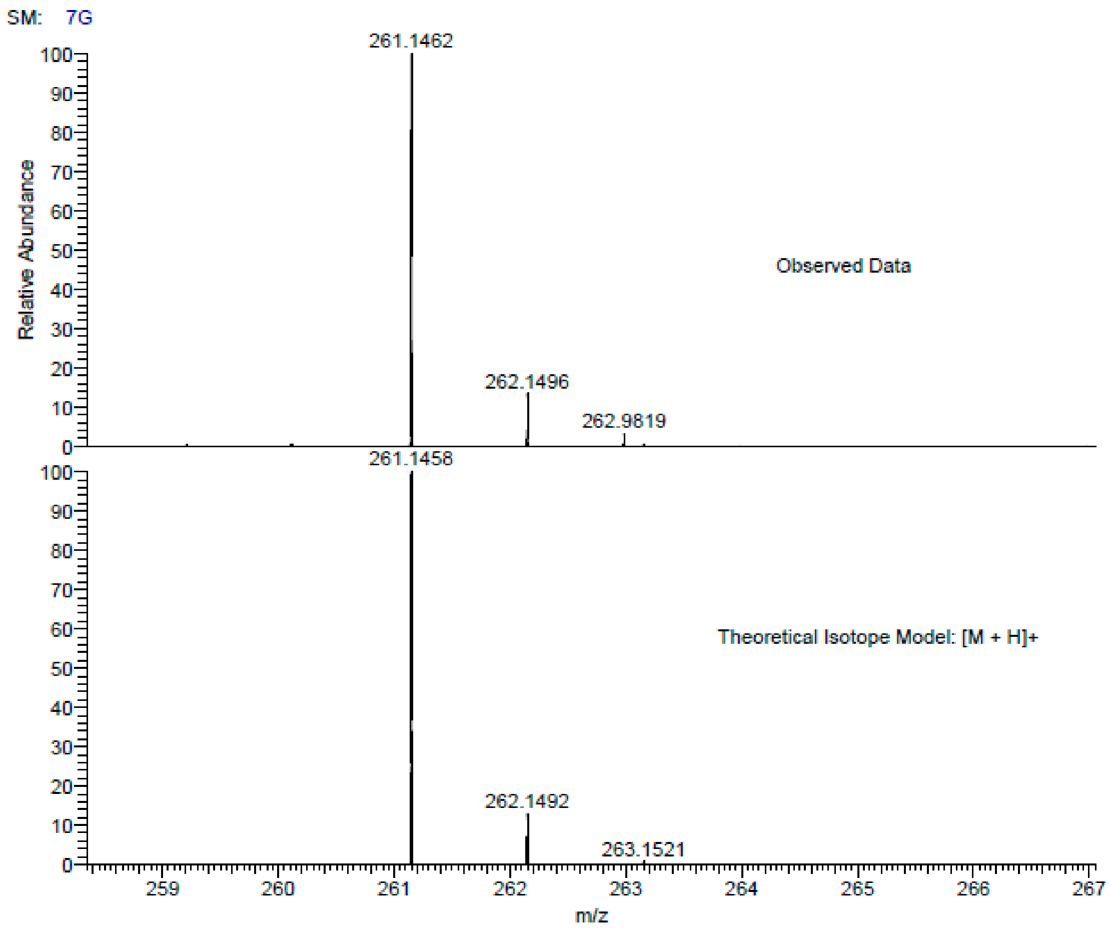

SM: 7G
261.1462
262.1496
262.9819
Observed Data
Relative Abundance
261.1458
262.1492
263.1521
Theoretical Isotope Model: [M + H]+
m/z

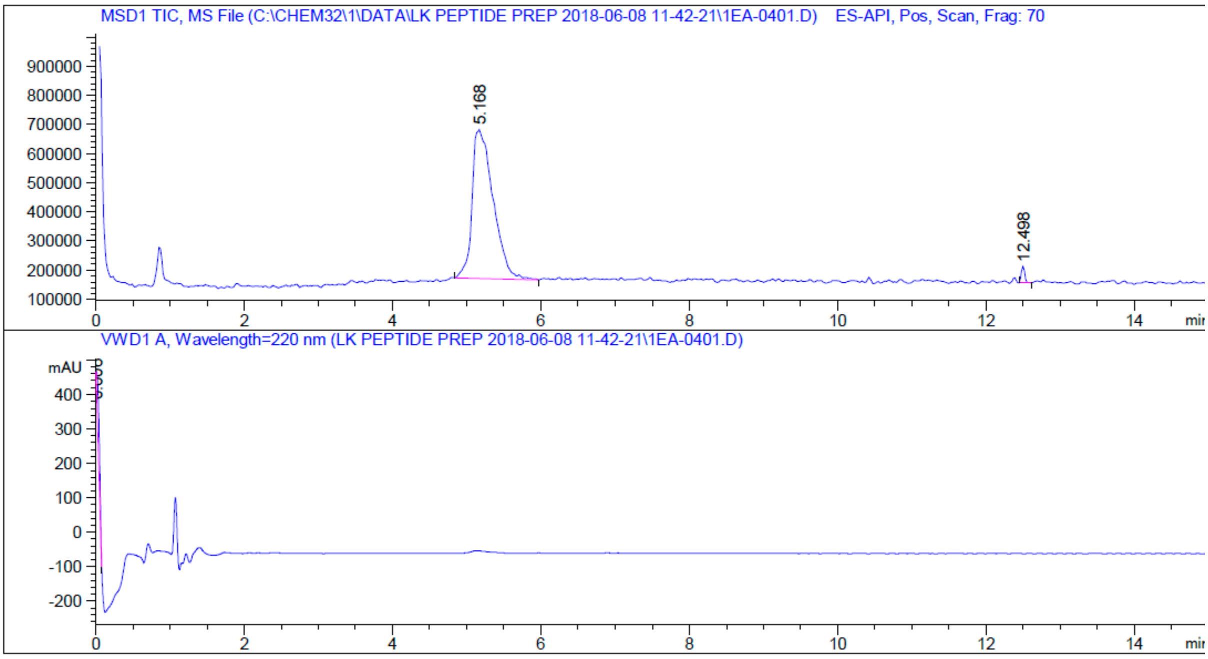


```
=====================================================================
                         Area Percent Report
=====================================================================

Sorted By             :      Signal
Multiplier:                  :      1.0000
Dilution:                    :      1.0000
Use Multiplier & Dilution Factor with ISTDs


Signal 1: MSD1 TIC, MS File

Peak RetTime Type  Width     Area      Height     Area
  #   [min]        [min]                            %
----|-------|----|-------|----------|----------|--------|
   1   5.168 BB    0.2626 1.00788e7  5.11773e5  98.1589
   2  12.498 BB    0.0551 1.89042e5  5.70412e4   1.8411

Totals :                  1.02678e7  5.68814e5
```

Benzyl-*N*-[(1*S*)-1-{[(1*S*)-1-(1*H*-1,2,3,4-tetrazol-5-yl)propyl]carbamoyl}ethyl]carbamate **13b-LL**:

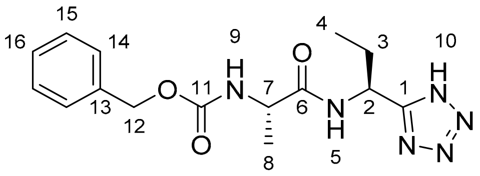

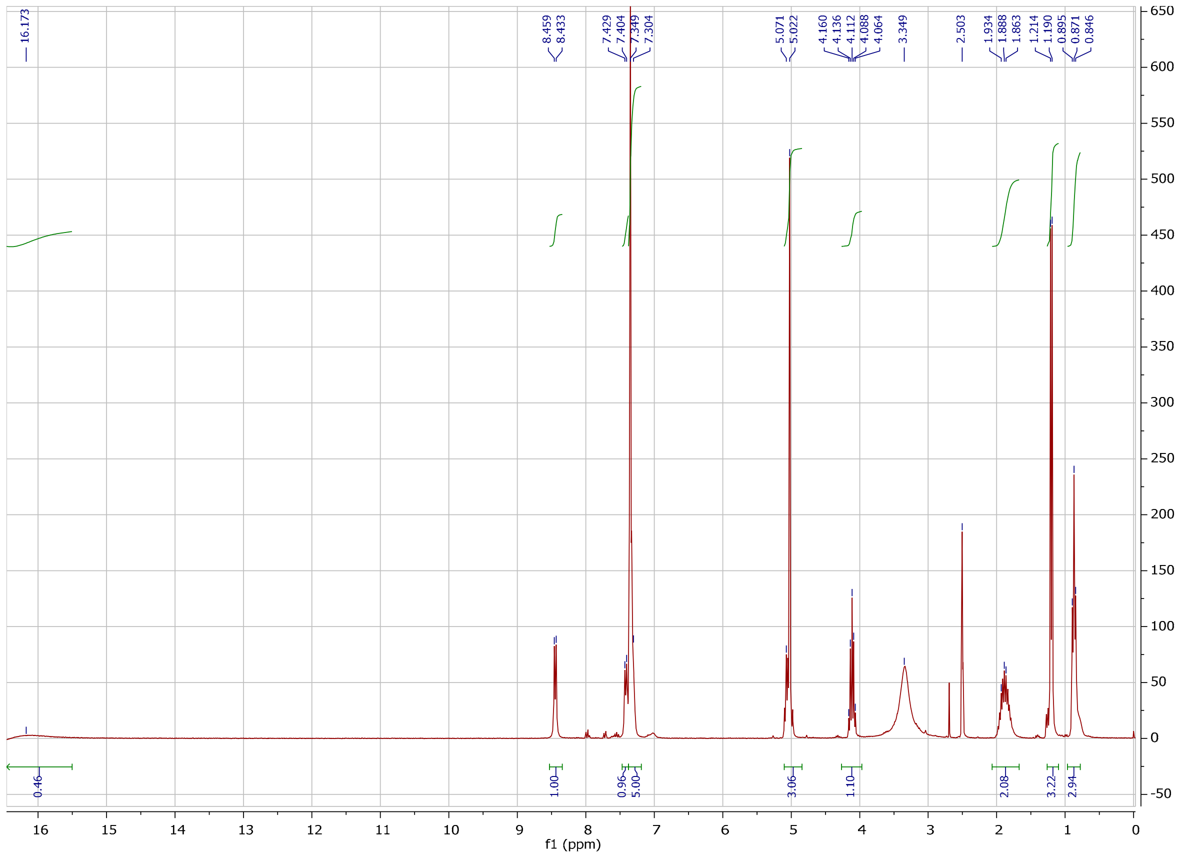
16.173
8.459
8.433
7.429
7.404
7.349
7.304
5.071
5.022
4.160
4.136
4.112
4.088
4.064
3.349
2.503
1.934
1.888
1.863
1.214
1.190
0.895
0.871
0.846
0.46
1.00
0.96
5.00
3.06
1.10
2.08
3.22
2.94
f1 (ppm)

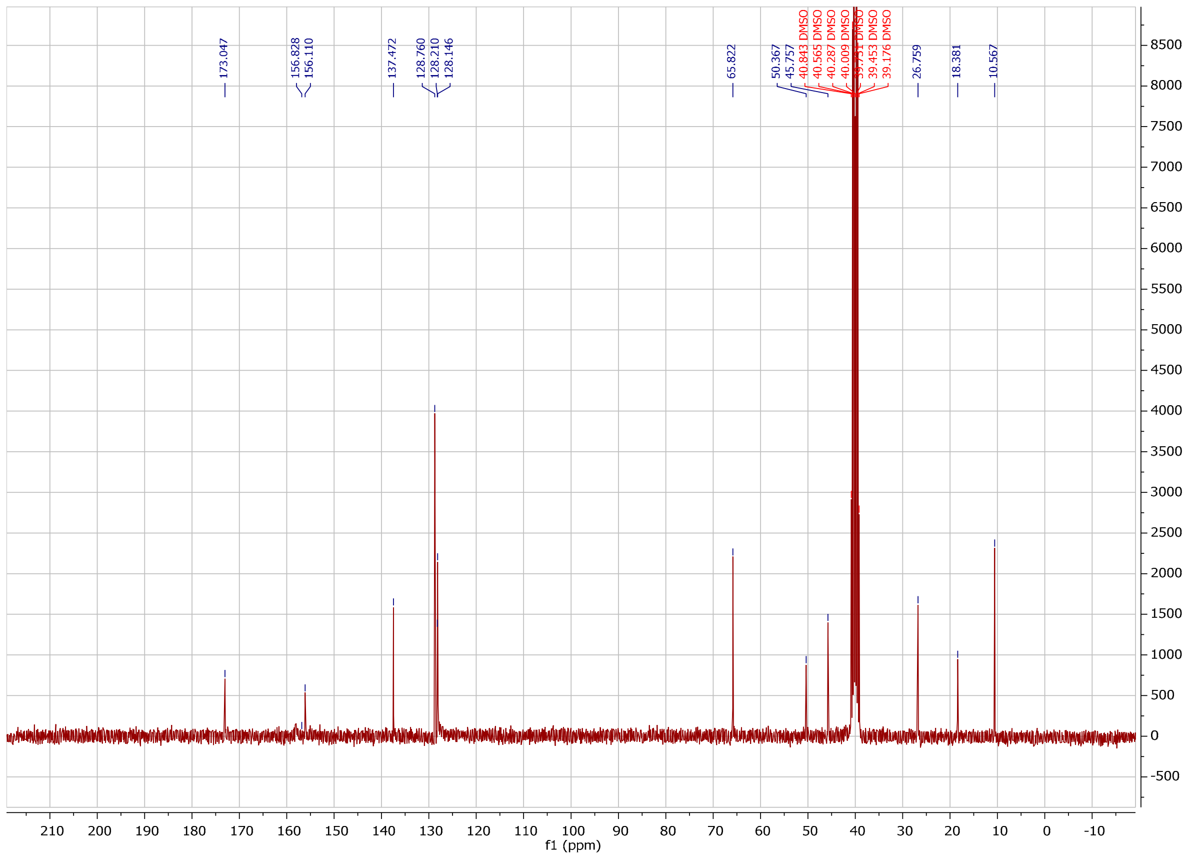
173.047
156.828
156.110
137.472
128.760
128.210
128.146
65.822
50.367
45.757
40.843 DMSO
40.565 DMSO
40.287 DMSO
40.009 DMSO
39.731 DMSO
39.453 DMSO
39.176 DMSO
26.759
18.381
10.567
f1 (ppm)

(2*S*)-2-Amino-*N*-[(1*S*)-1-(1*H*-1,2,3,4-tetrazol-5-yl)propyl]propanamide **14b-LL**:

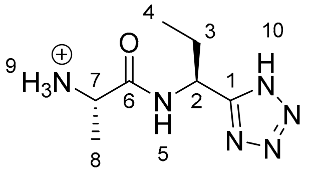

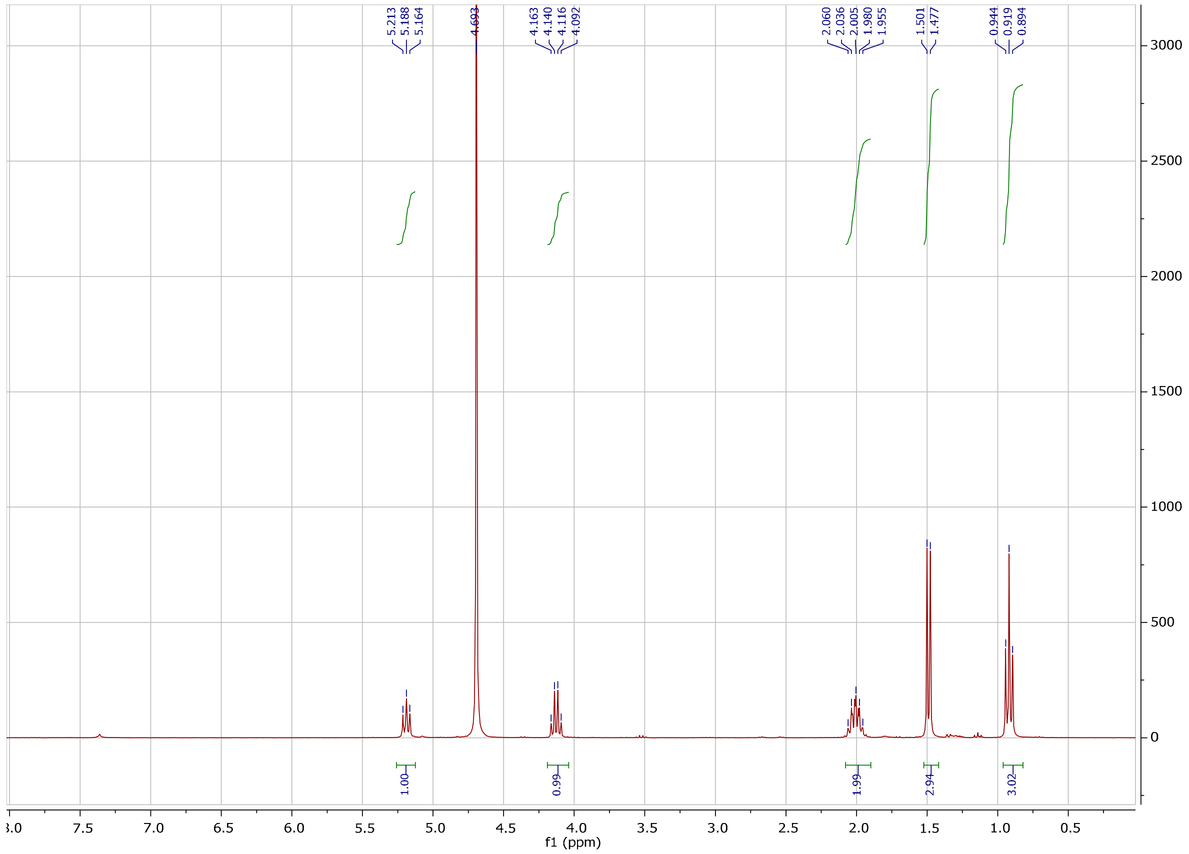

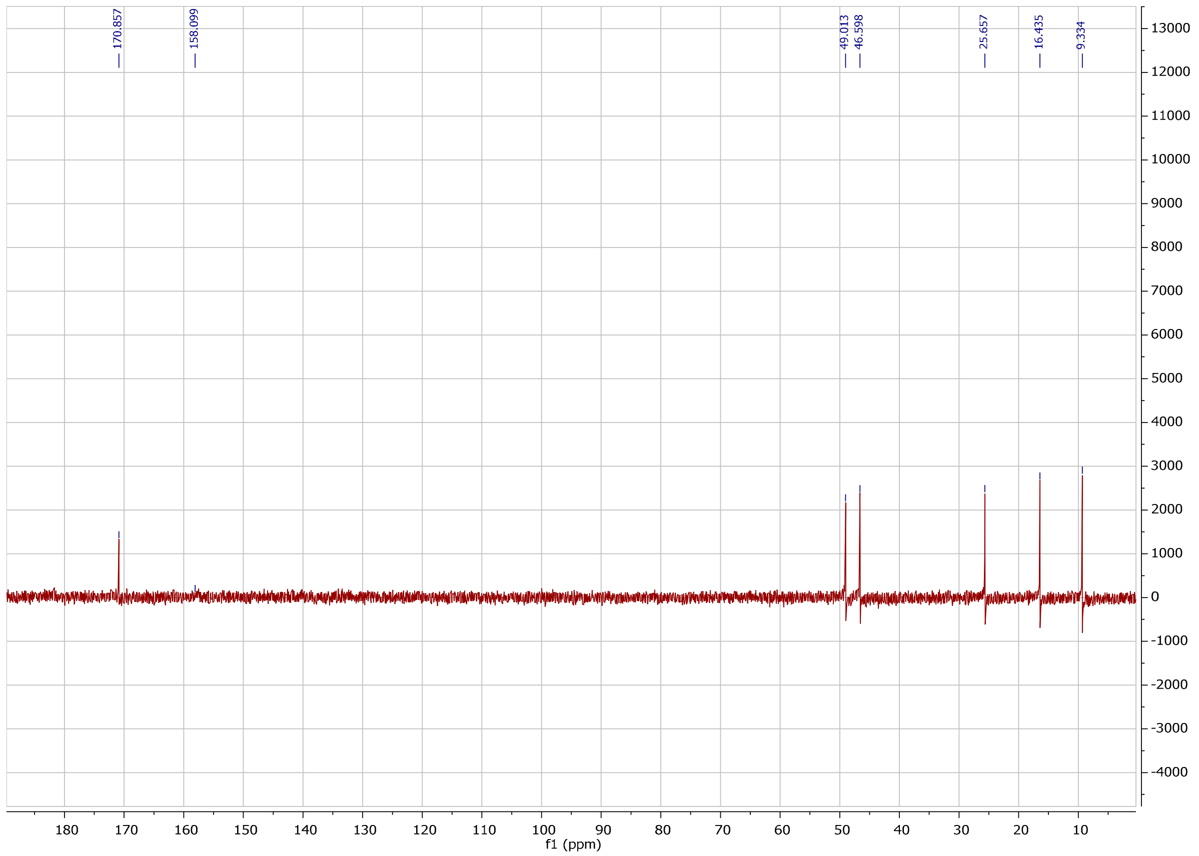
170.857
158.099
49.013
46.598
25.657
16.435
9.334
f1 (ppm)

SM: 7G

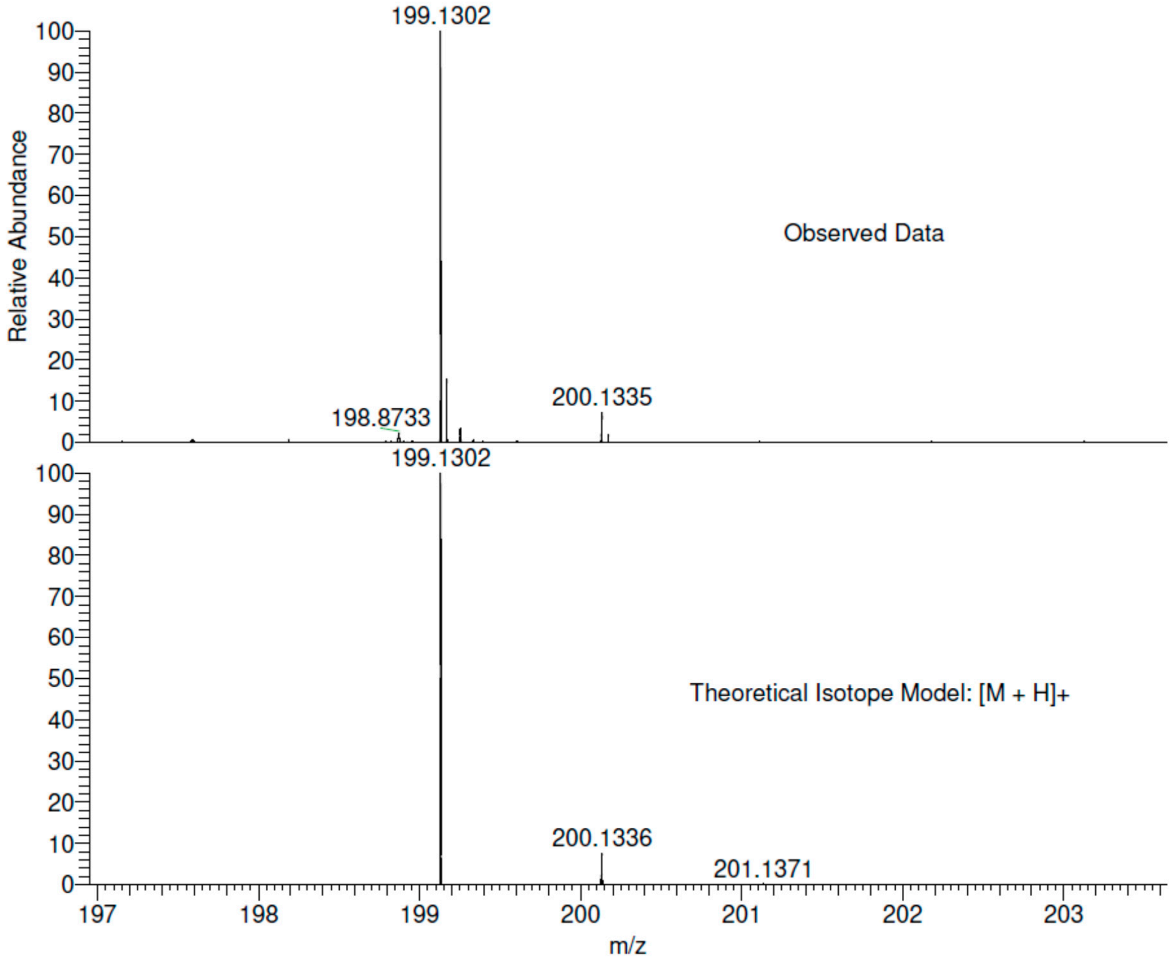

199.1302
Observed Data
198.8733
200.1335
199.1302
Theoretical Isotope Model: [M + H]+
200.1336
201.1371
Relative Abundance
m/z

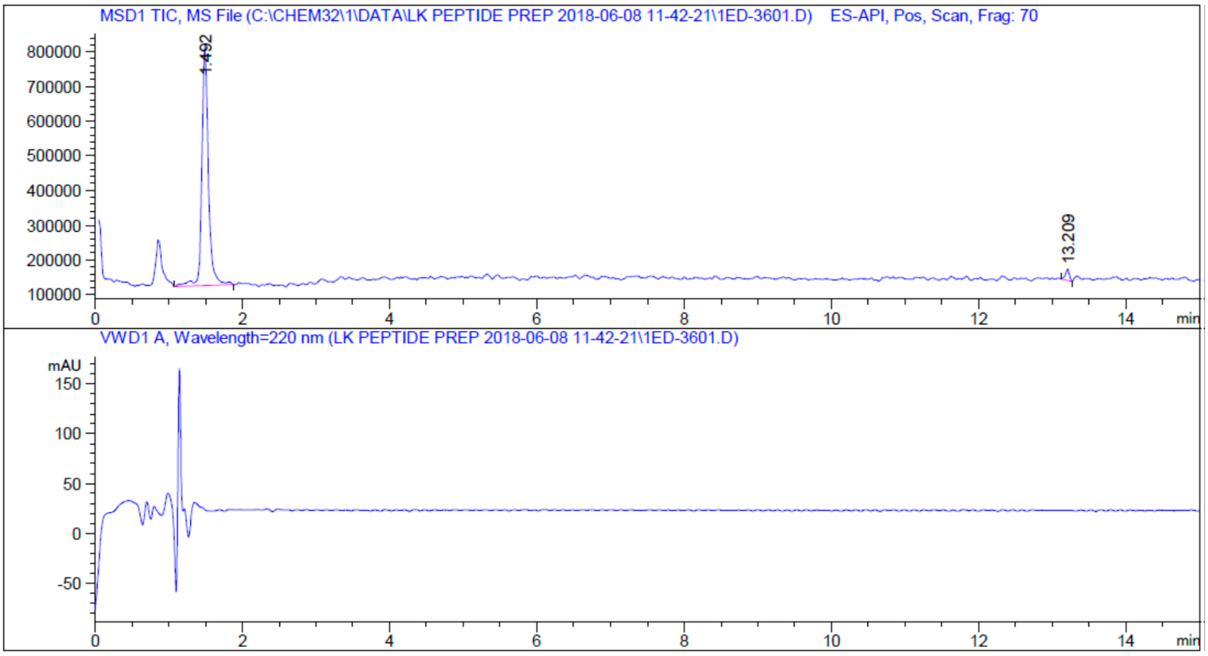


```
=====================================================================
                         Area Percent Report
=====================================================================

Sorted By             :      Signal
Multiplier:                  :      1.0000
Dilution:                    :      1.0000
Use Multiplier & Dilution Factor with ISTDs


Signal 1: MSD1 TIC, MS File

Peak RetTime Type  Width     Area      Height     Area
  #    [min]        [min]                          %
----|-------|----|-------|----------|----------|--------|
   1   1.492 BB    0.0888 4.28483e6  6.90281e5  97.1740
   2  13.209 BB    0.0588 1.24610e5  3.43131e4   2.8260

Totals :                  4.40944e6  7.24594e5
```

N-[(1*S*)-1-{[(1*S*)-1-{[(1*S*)-1-(1*H*-1,2,3,4-tetrazol-5-yl)ethyl]carbamoyl}ethyl]carbamoyl} ethyl]carbamate **13c-LLL**:

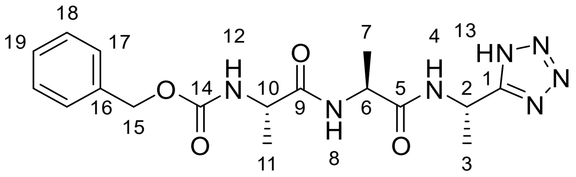

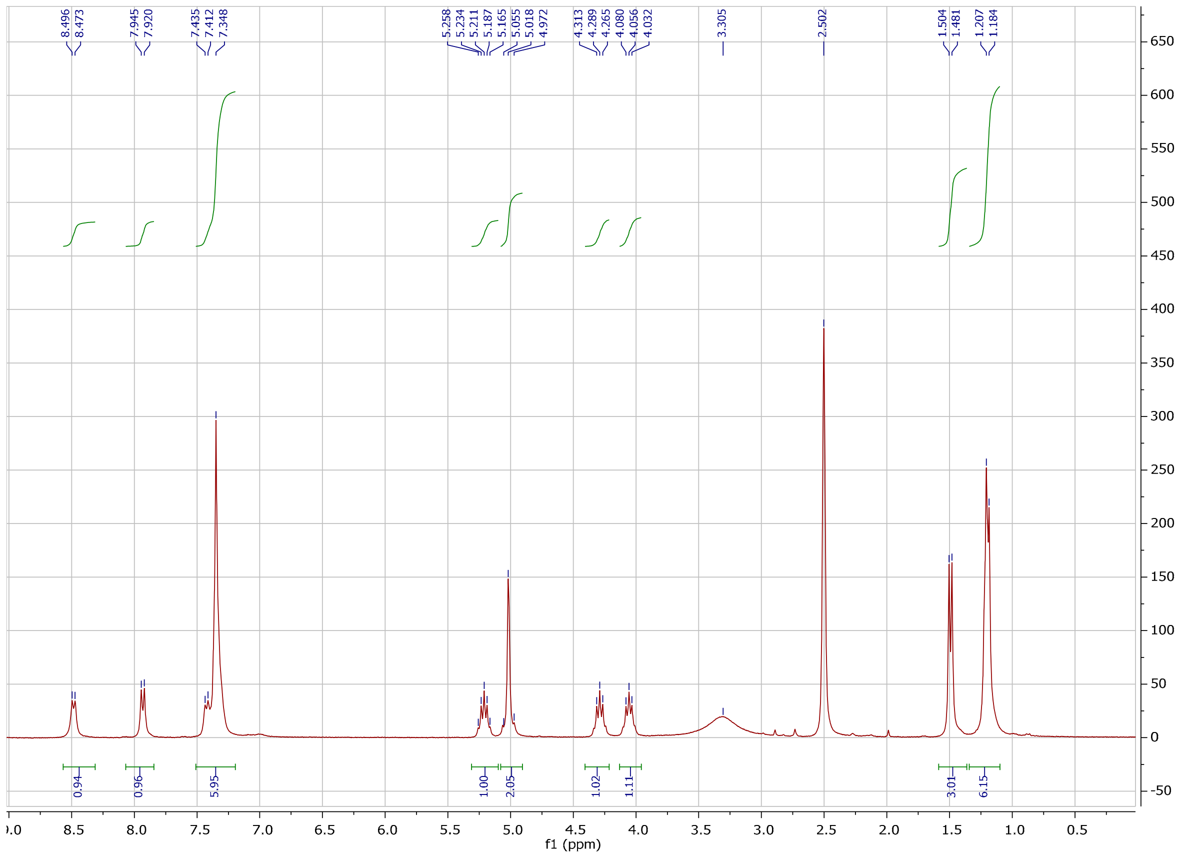

8.496
8.473
7.945
7.920
7.435
7.412
7.348
5.258
5.234
5.211
5.187
5.165
5.055
5.018
4.972
4.313
4.289
4.265
4.080
4.056
4.032
3.305
2.502
1.504
1.481
1.207
1.184
0.94
0.96
5.95
1.00
2.05
1.02
1.11
3.01
6.15
f1 (ppm)

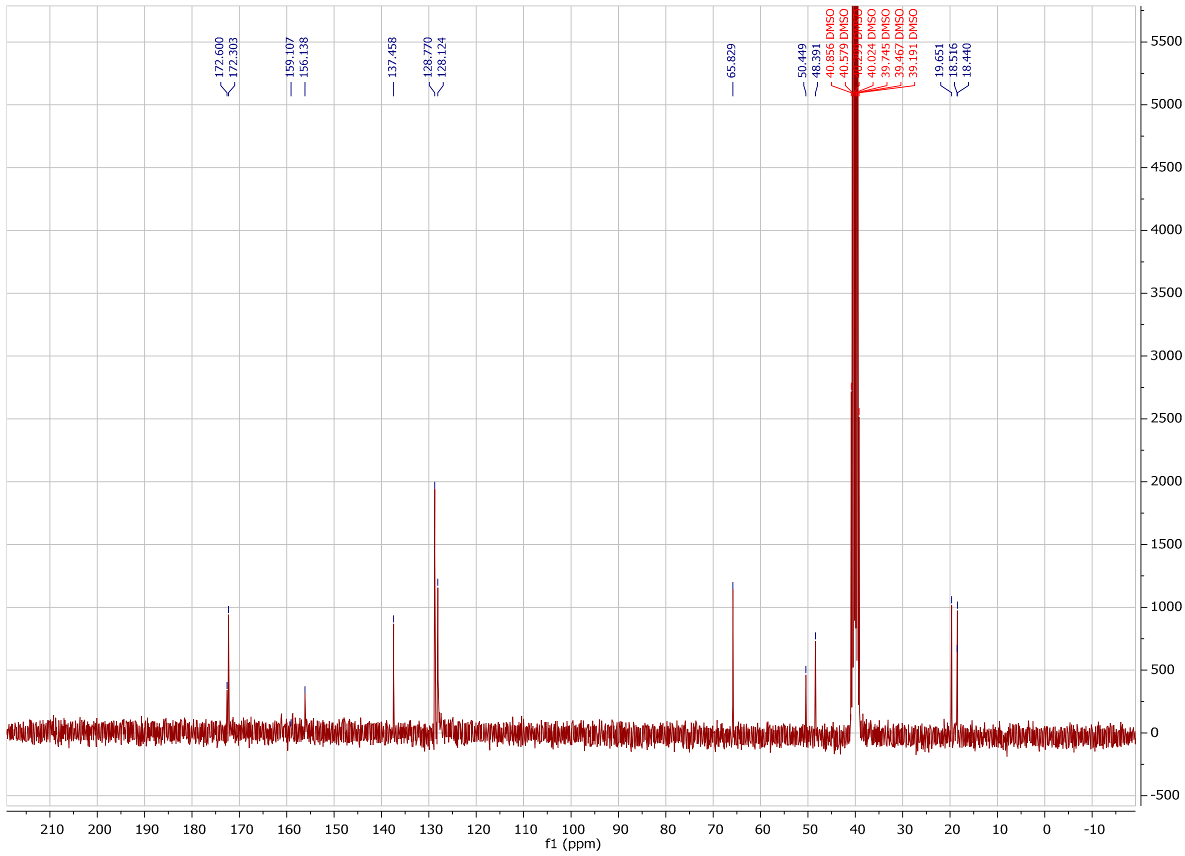

172.600
172.303
159.107
156.138
137.458
128.770
128.124
65.829
50.449
48.391
40.856 DMSO
40.579 DMSO
40.024 DMSO
39.745 DMSO
39.467 DMSO
39.191 DMSO
19.651
18.516
18.440
f1 (ppm)

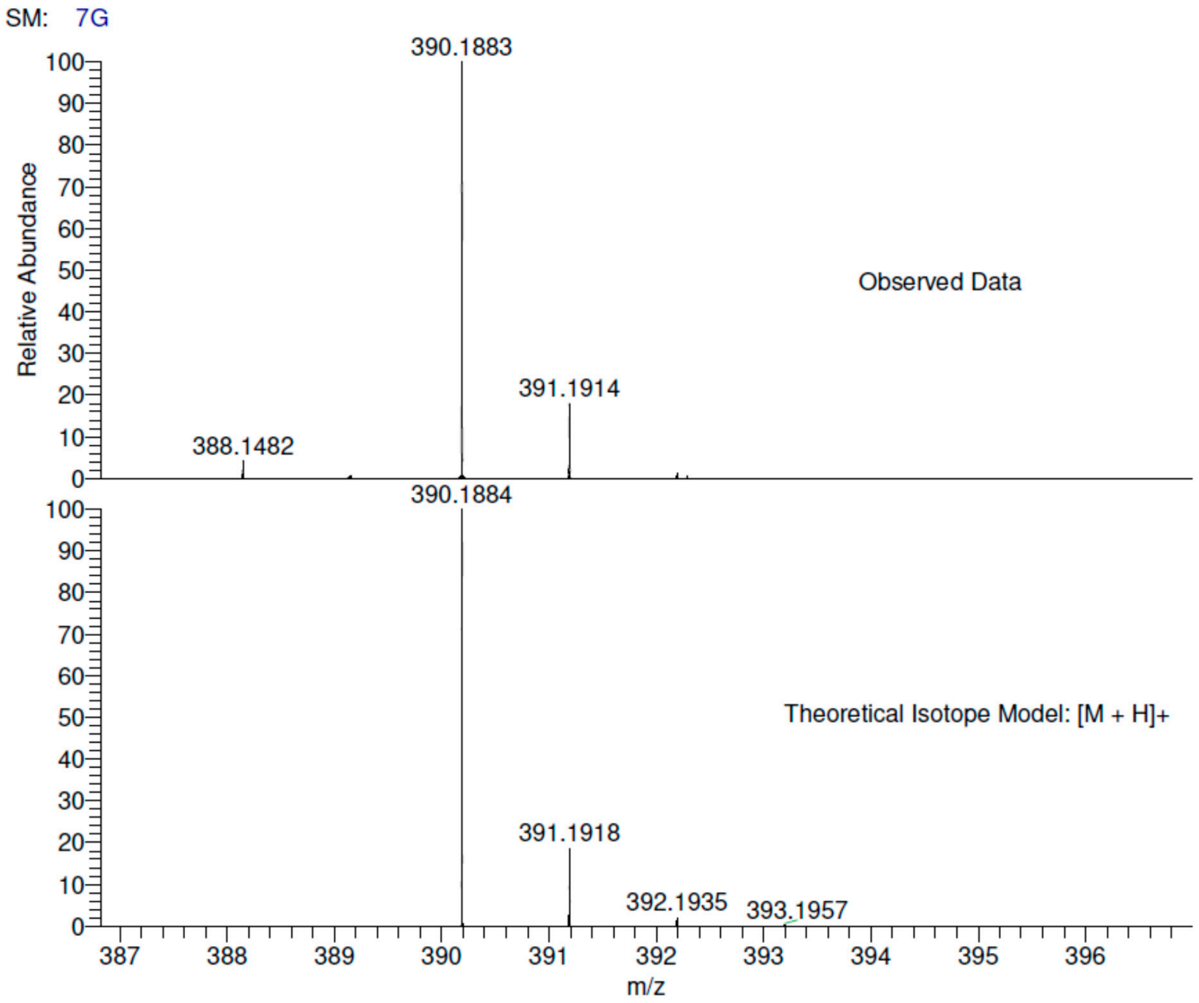

SM: 7G
390.1883
Relative Abundance
Observed Data
391.1914
388.1482
390.1884
Theoretical Isotope Model: [M + H]+
391.1918
392.1935
393.1957
387
388
389
390
391
392
393
394
395
396
m/z

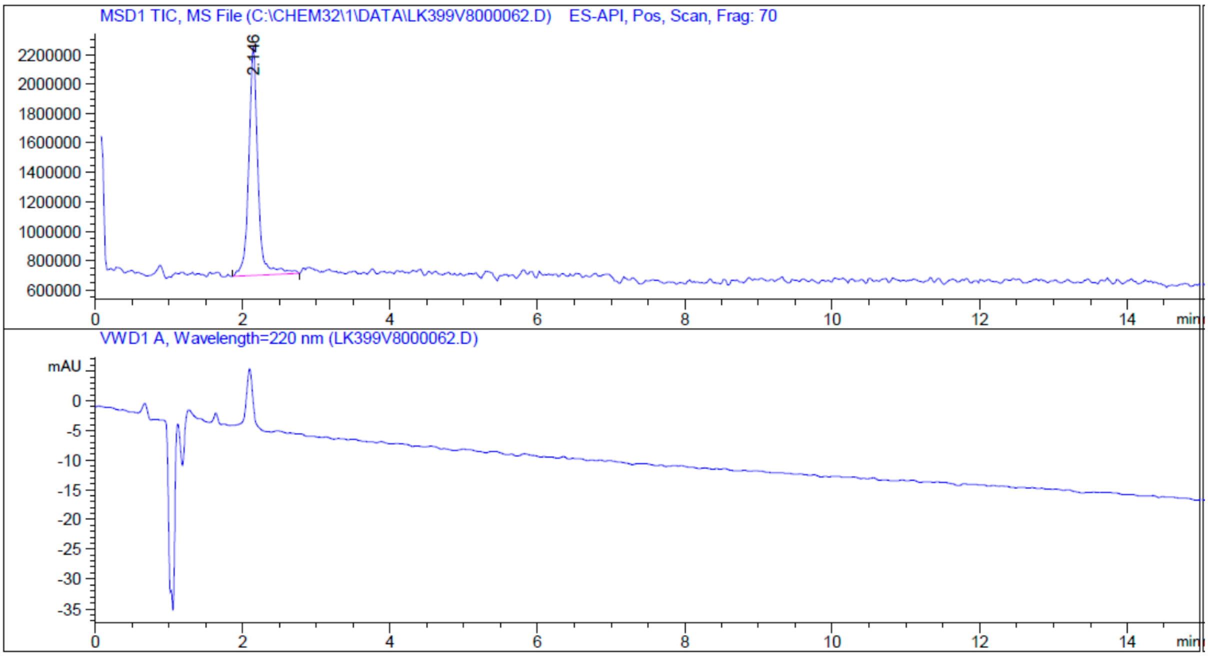


```
=====================================================================
                         Area Percent Report
=====================================================================

Sorted By             :      Signal
Multiplier:                    :      1.0000
Dilution:                      :      1.0000
Use Multiplier & Dilution Factor with ISTDs


Signal 1: MSD1 TIC, MS File

Peak RetTime Type  Width     Area      Height     Area
  #   [min]        [min]                           %
----|-------|----|-------|----------|----------|--------|
   1   2.146 BB    0.1280 1.34620e7  1.55971e6  100.0000

Totals :                  1.34620e7  1.55971e6
```

(*S*)-*N*-((*S*)-1-(1*H*-tetrazol-5-yl)ethyl)-2-((*S*)-2-aminopropanamido)propanamide **14c-LLL**:

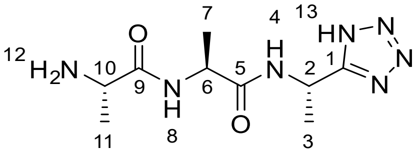

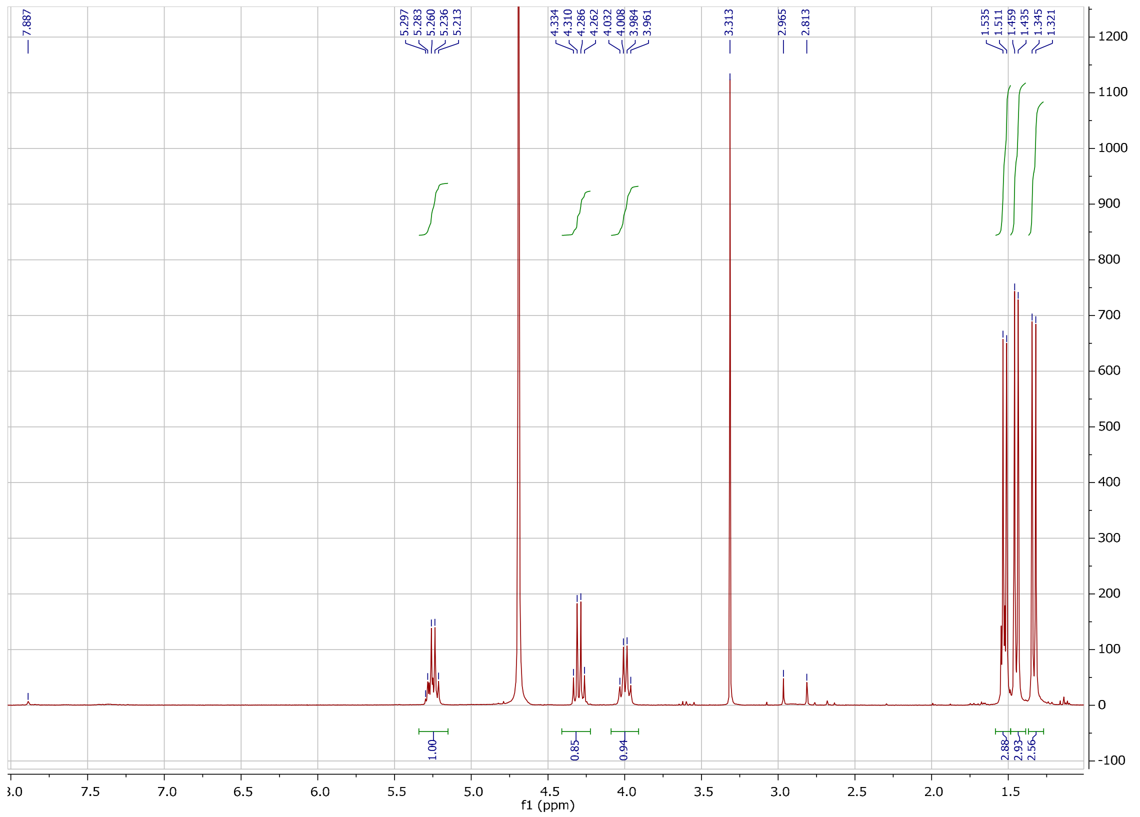
7.887
5.297
5.283
5.260
5.236
5.213
4.334
4.310
4.286
4.262
4.032
4.008
3.984
3.961
3.313
2.965
2.813
1.535
1.511
1.459
1.435
1.345
1.321
1.00
0.85
0.94
2.88
2.93
2.56
f1 (ppm)

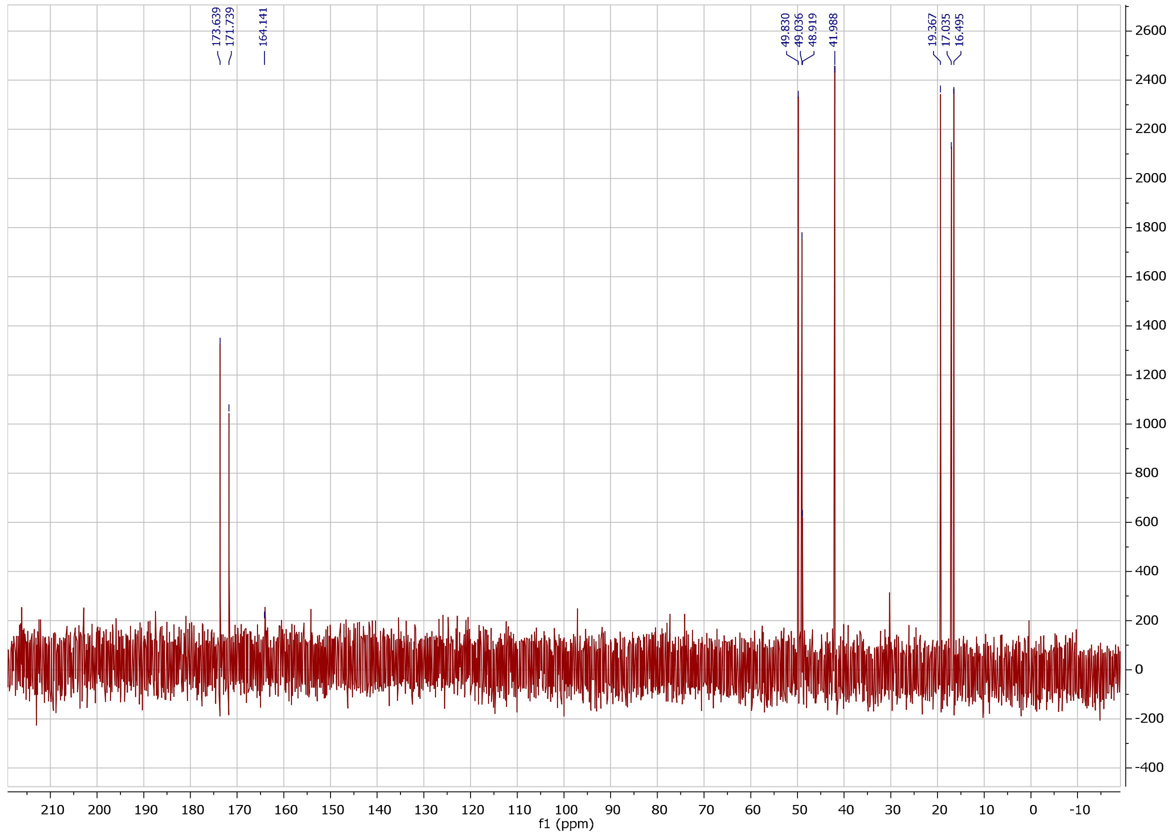
173.639
171.739
164.141
49.830
49.036
48.919
41.988
19.367
17.035
16.495
f1 (ppm)

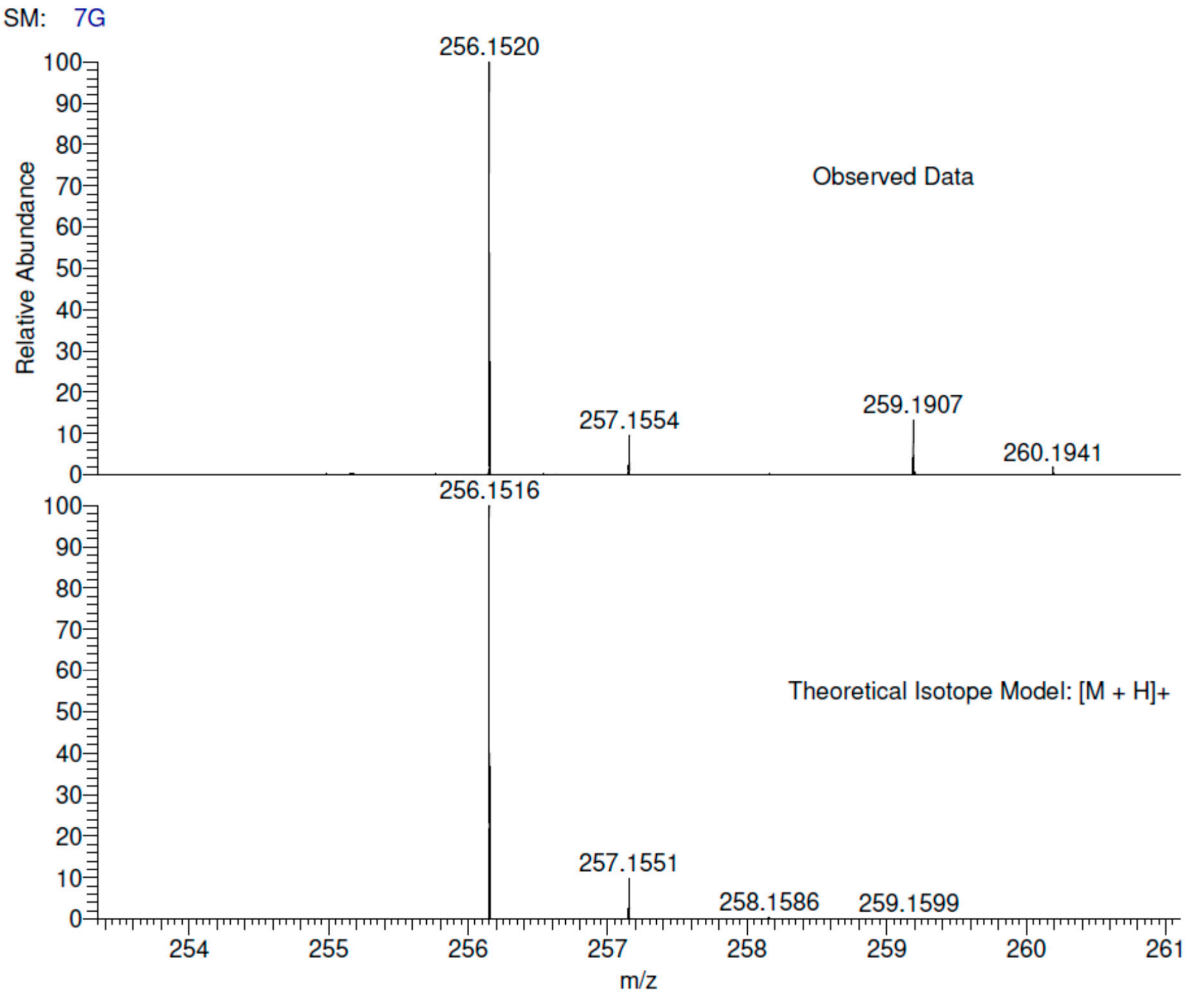

SM: 7G
256.1520
Observed Data
257.1554
259.1907
260.1941
256.1516
Theoretical Isotope Model: [M + H]+
257.1551
258.1586
259.1599
Relative Abundance
m/z

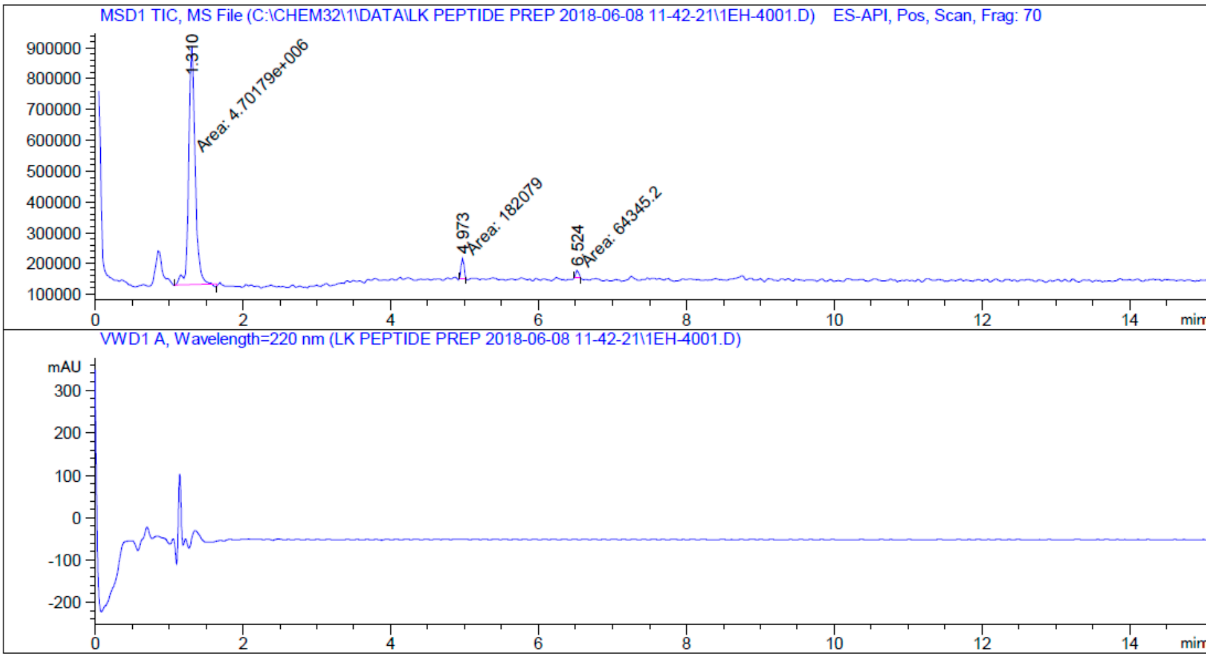


```
=====================================================================
                         Area Percent Report
=====================================================================

Sorted By             :      Signal
Multiplier:                  :      1.0000
Dilution:                    :      1.0000
Use Multiplier & Dilution Factor with ISTDs


Signal 1: MSD1 TIC, MS File

Peak RetTime Type  Width     Area      Height     Area
  #   [min]        [min]                            %
----|-------|----|-------|----------|----------|--------|
   1   1.310 MM    0.1002 4.70179e6  7.82248e5  95.0199
   2   4.973 MM    0.0457 1.82079e5  6.63530e4   3.6797
   3   6.524 MM    0.0427 6.43452e4  2.51431e4   1.3004

Totals :                  4.94821e6  8.73744e5
```

3-{[(1S)-1-{[(1S)-1-(1*H*-1,2,3,4-tetrazol-5-yl)ethyl]carbamoyl}ethyl]carbamoyl}propanoic acid **16-LL**:

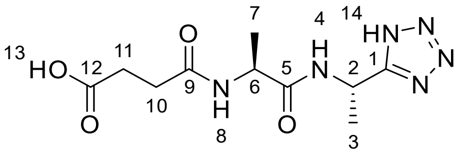

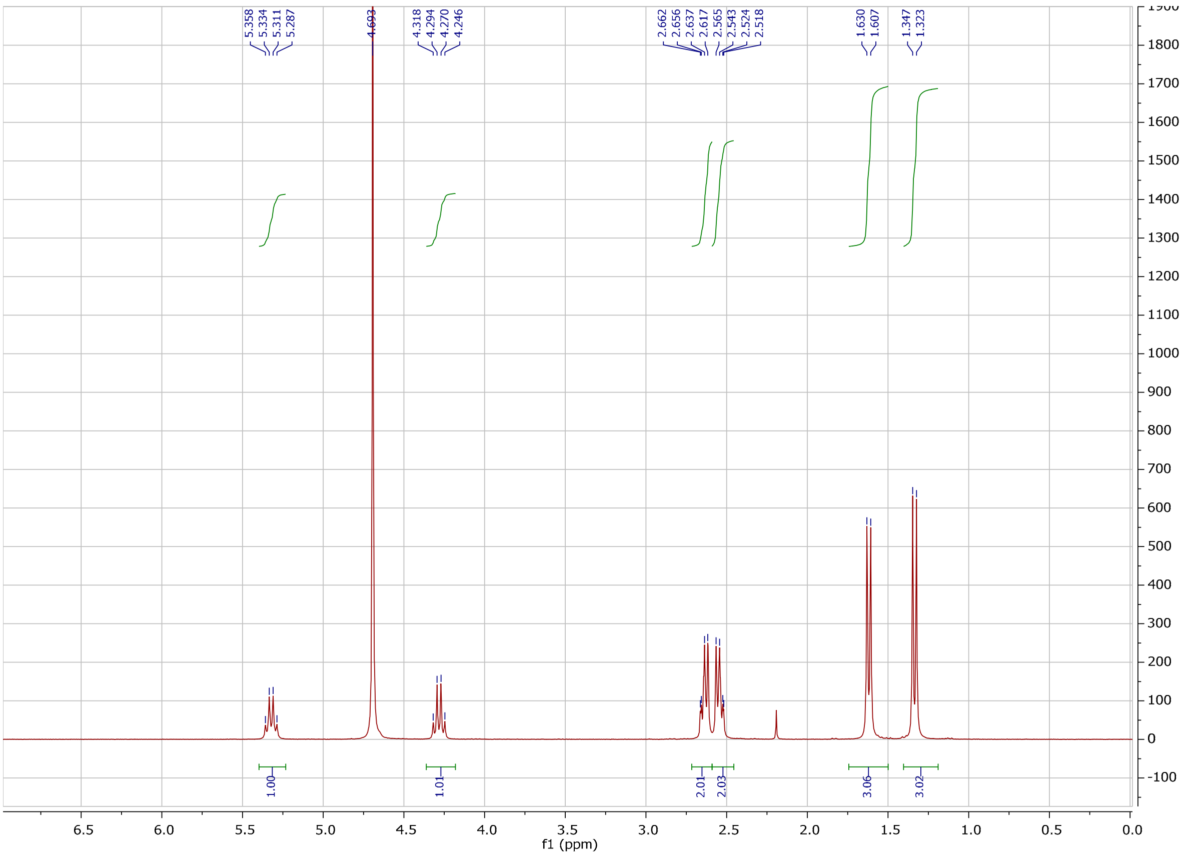
5.358
5.334
5.311
5.287
4.693
4.318
4.294
4.270
4.246
2.662
2.656
2.637
2.617
2.565
2.543
2.524
2.518
1.630
1.607
1.347
1.323
1.00
1.01
2.01
2.03
3.06
3.02
f1 (ppm)

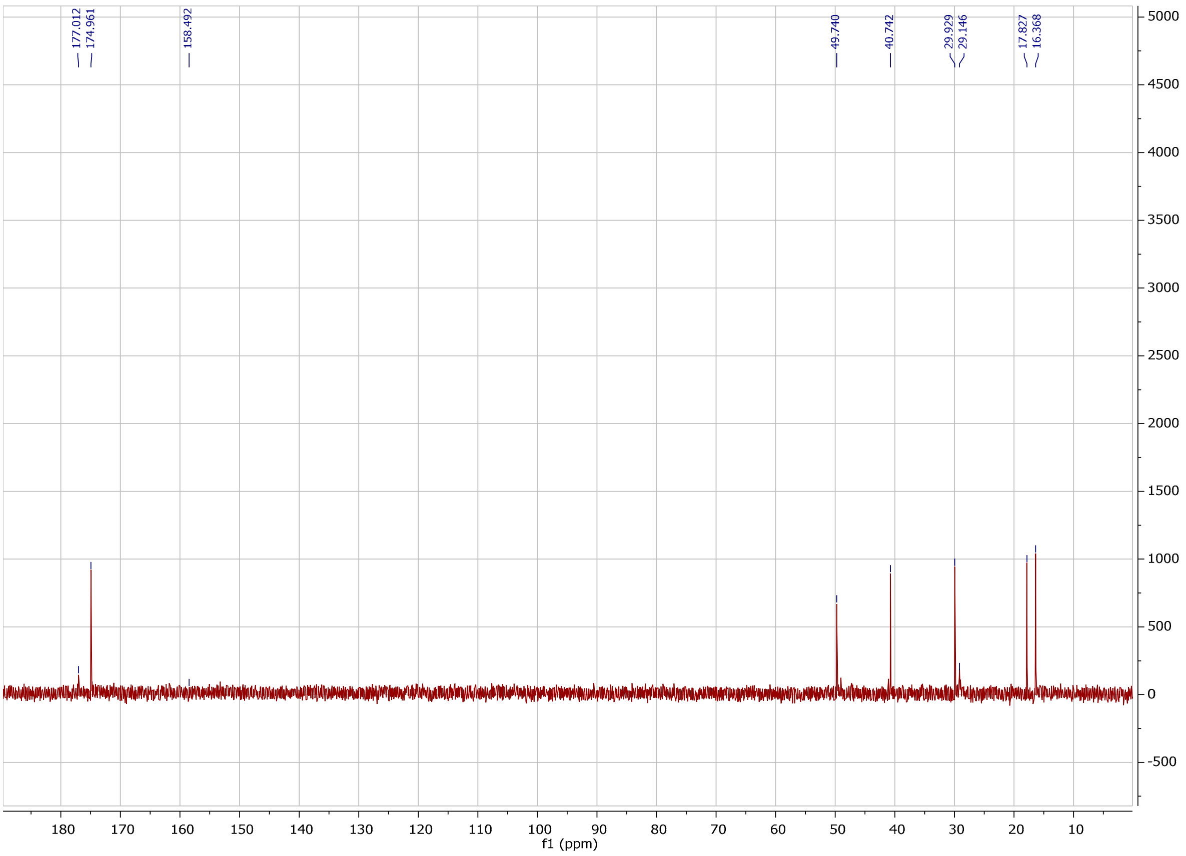
177.012
174.961
158.492
49.740
40.742
29.929
29.146
17.827
16.368
f1 (ppm)

SM: 7G

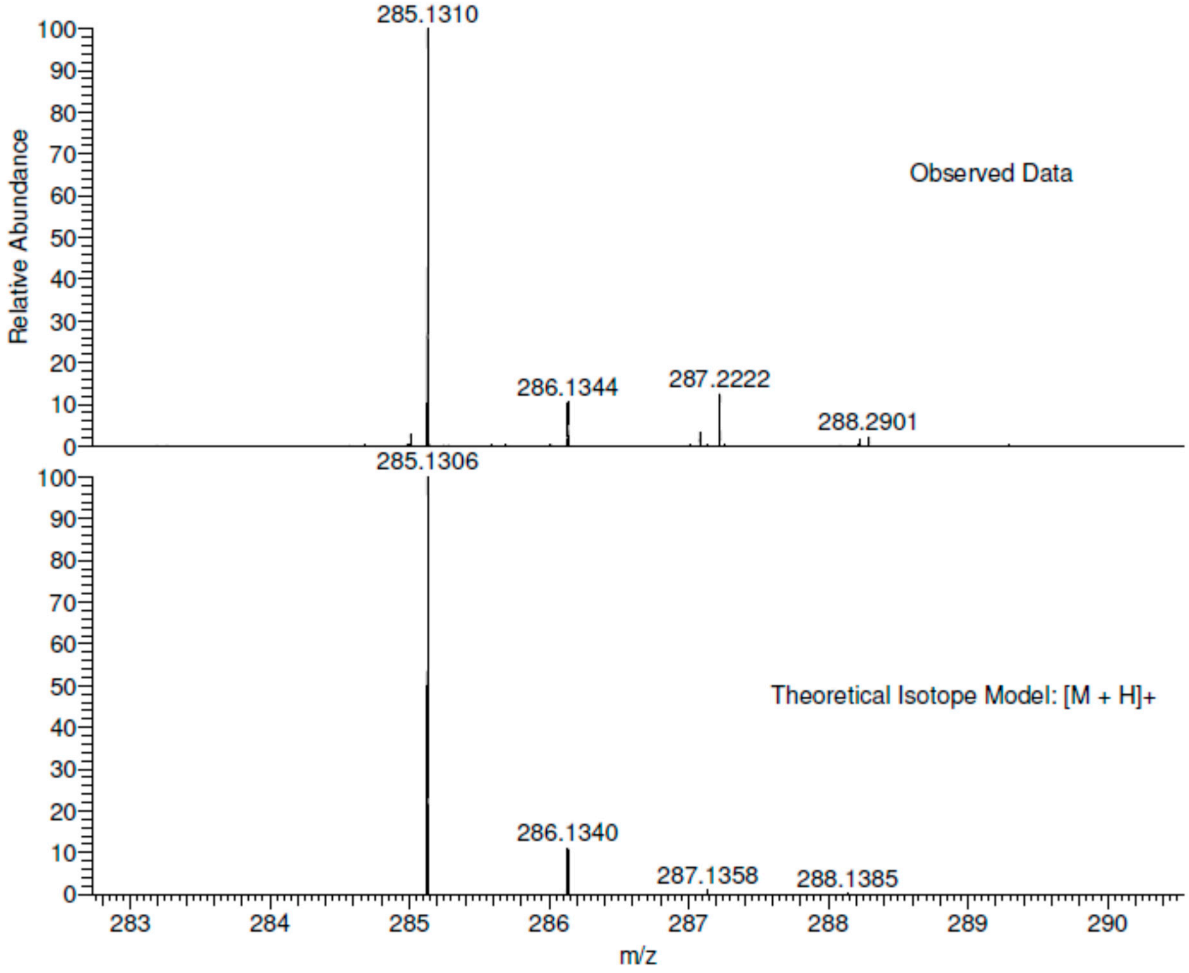

285.1310
286.1344
287.2222
288.2901
Observed Data
285.1306
286.1340
287.1358
288.1385
Theoretical Isotope Model: [M + H]+
Relative Abundance
m/z

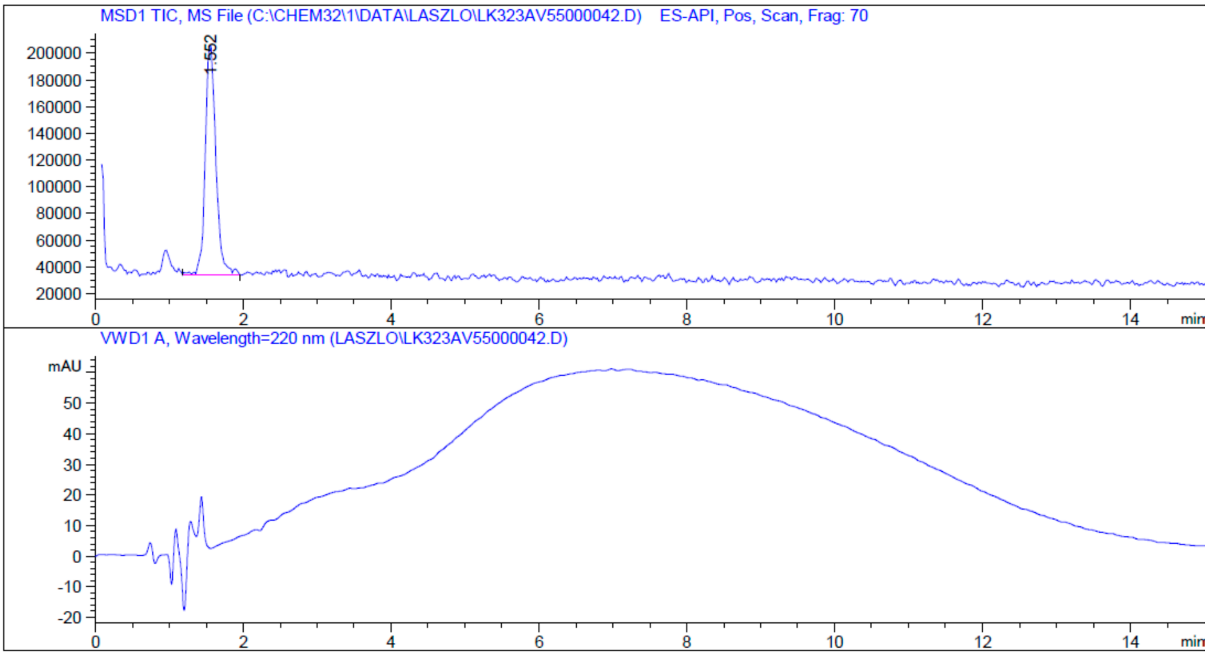

===

Area Percent Report

===

Sorted By : Signal
Multiplier: : 1.0000
Dilution: : 1.0000
Use Multiplier & Dilution Factor with ISTDs

Signal 1: MSD1 TIC, MS File

Peak #	RetTime [min]	Type	Width [min]	Area	Height	Area %
1	1.552	BB	0.1551	1.73583e6	1.73335e5	100.0000

Totals : 1.73583e6 1.73335e5

3-{[(1S)-1-{[(1S)-1-{[(1S)-1-(1H-1,2,3,4-tetrazol-5-yl)ethyl]carbamoyl}ethyl]carbamoyl}ethyl]carbamoyl}propanoic acid **17-LLL**:

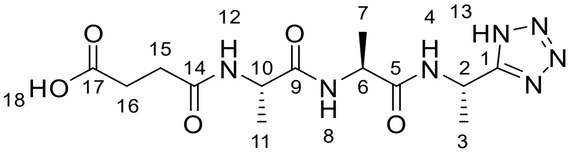

SSSSSSSSSSW

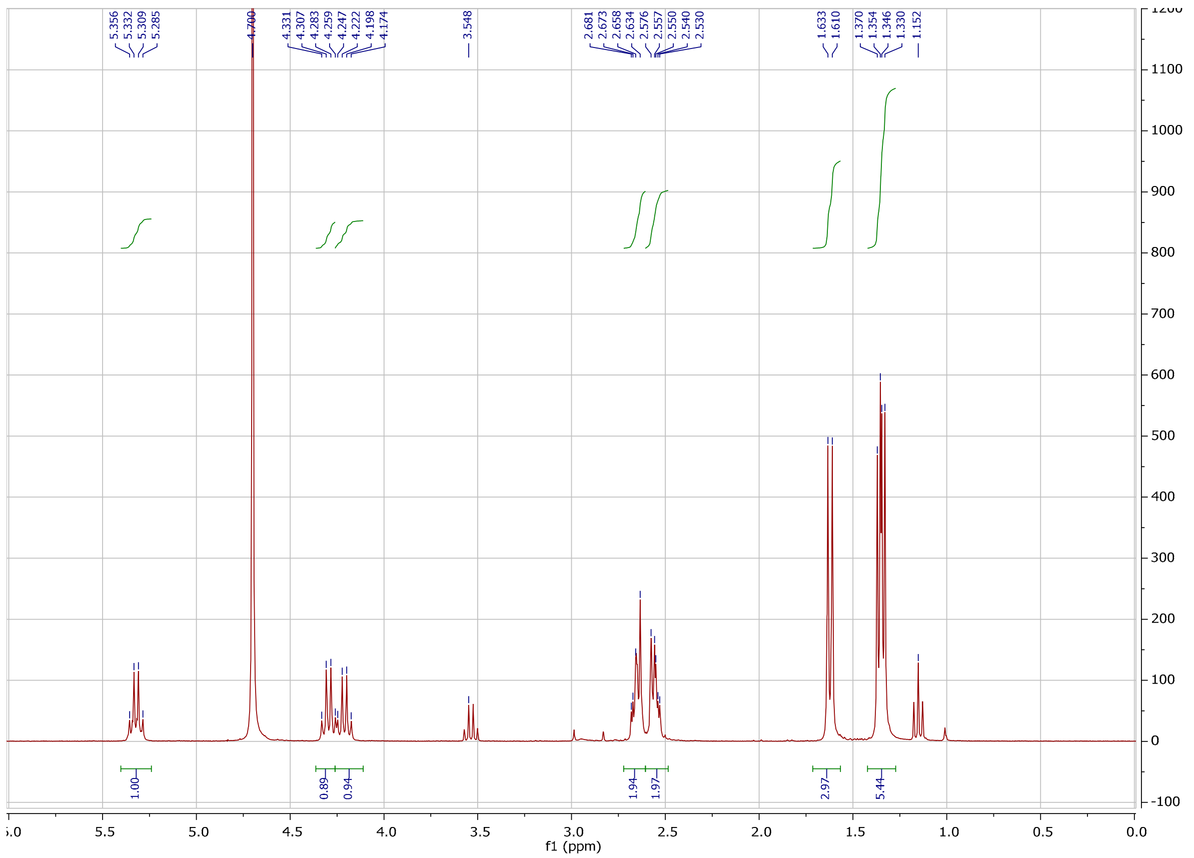

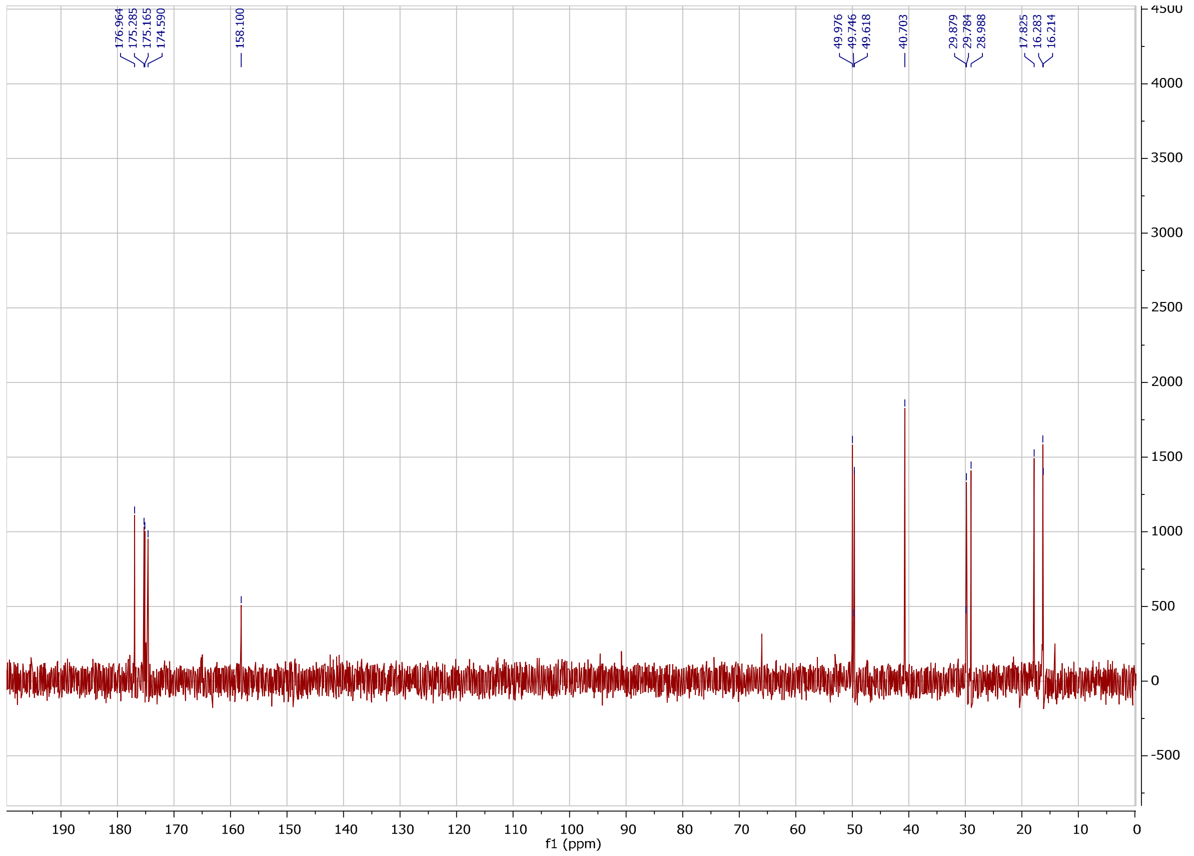

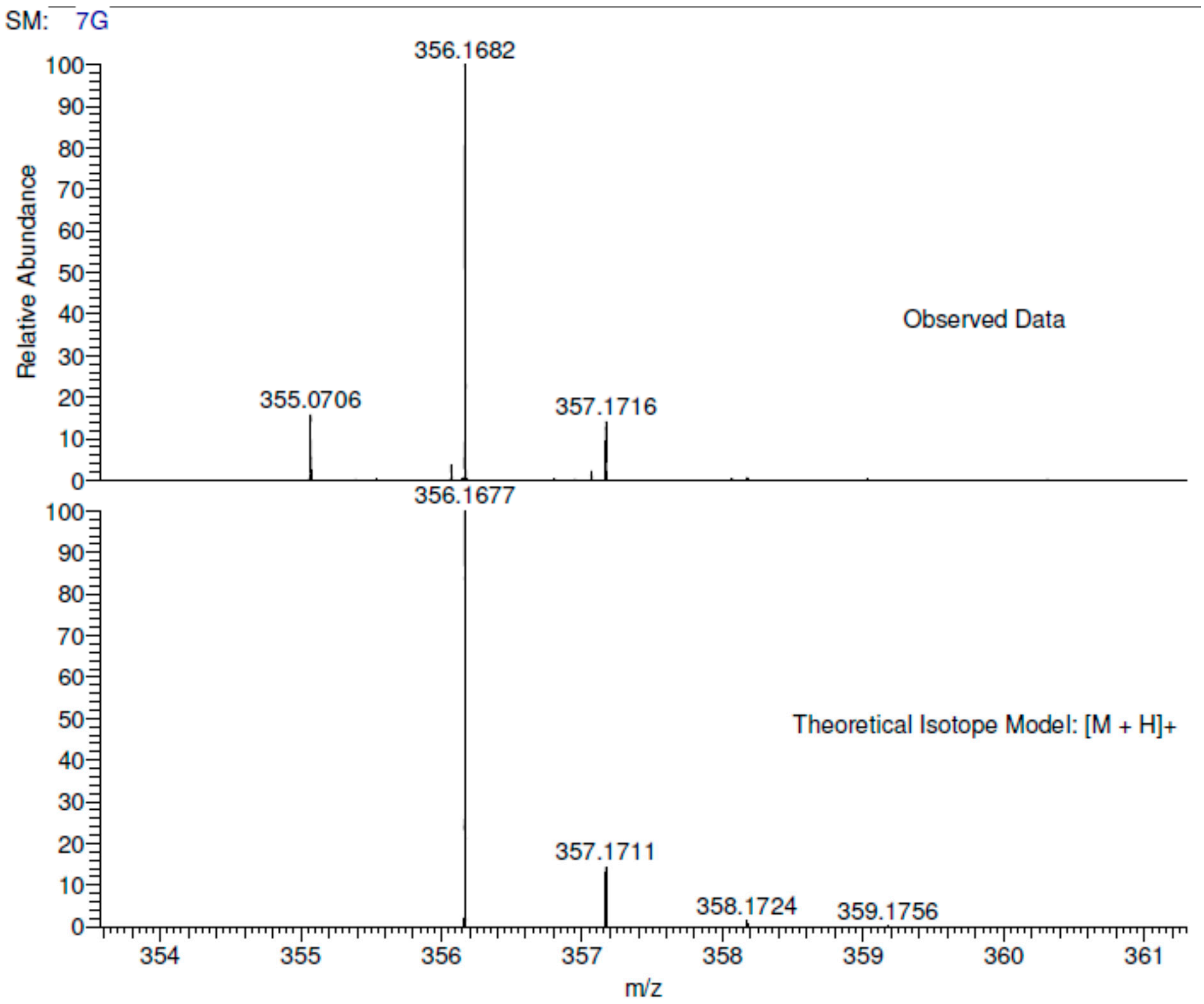
SM: 7G
356.1682
355.0706
357.1716
Observed Data
Relative Abundance
356.1677
357.1711
358.1724
359.1756
Theoretical Isotope Model: [M + H]+
m/z

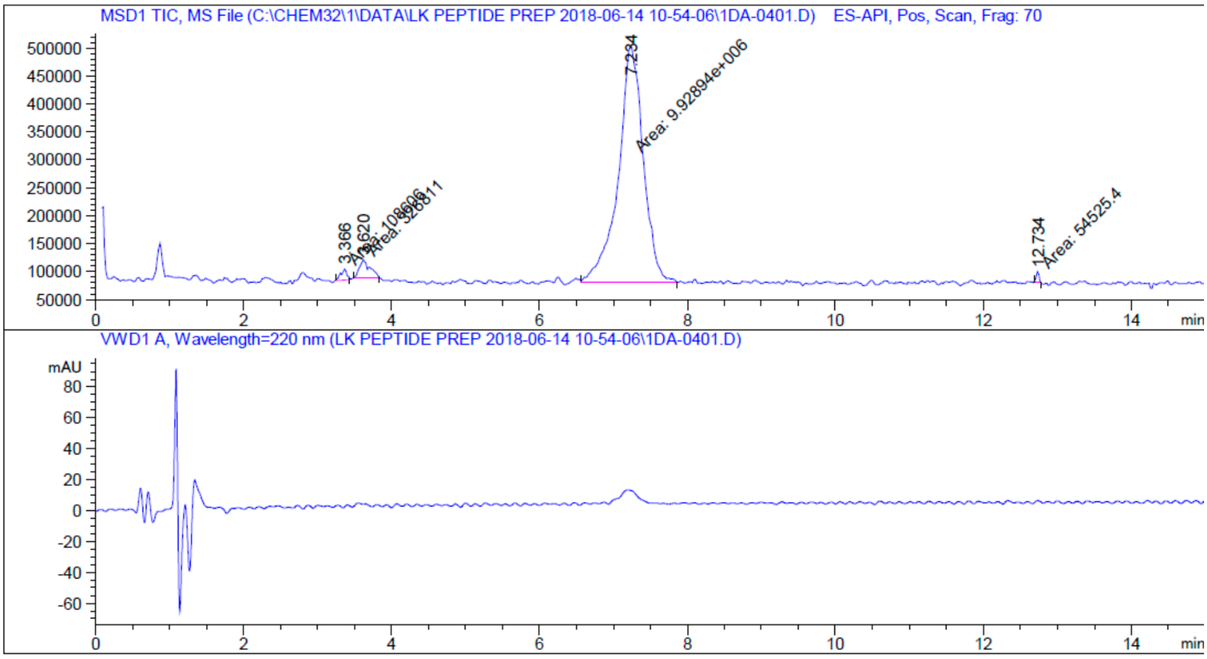

===

Area Percent Report

===

Sorted By : Signal
Multiplier: : 1.0000
Dilution: : 1.0000
Use Multiplier & Dilution Factor with ISTDs

Signal 1: MSD1 TIC, MS File

Peak #	RetTime [min]	Type	Width [min]	Area	Height	Area %
1	3.366	MM	0.0884	1.08606e5	2.04785e4	1.0424
2	3.620	MM	0.1675	3.26811e5	3.25102e4	3.1367
3	7.234	MM	0.3902	9.92894e6	4.24108e5	95.2976
4	12.734	MM	0.0456	5.45254e4	1.99175e4	0.5233

Totals : 1.04189e7 4.97014e5

3-{[(1*S*)-1-{[(1*S*)-1-{[(1*S*)-1-{[(1*S*)-1-(1*H*-1,2,3,4-tetrazol-5-yl)ethyl]carbamoyl}ethyl]carbamoyl}ethyl]carbamoyl}ethyl]carbamoyl}propanoic acid **18-LLLL**:

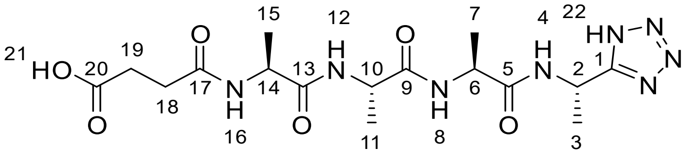

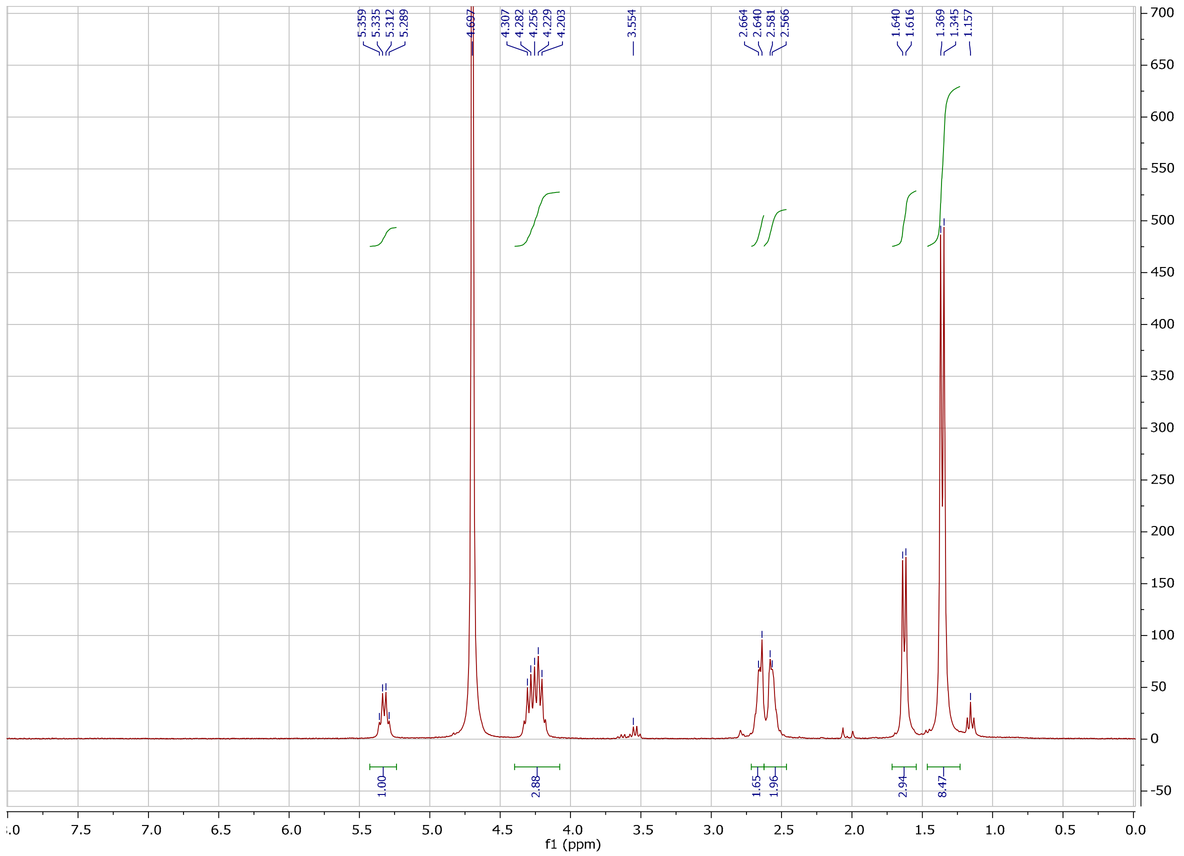
5.359
5.335
5.312
5.289
4.697
4.307
4.282
4.256
4.229
4.203
3.554
2.664
2.640
2.581
2.566
1.640
1.616
1.369
1.345
1.157
1.00
2.88
1.65
1.96
2.94
8.47
f1 (ppm)

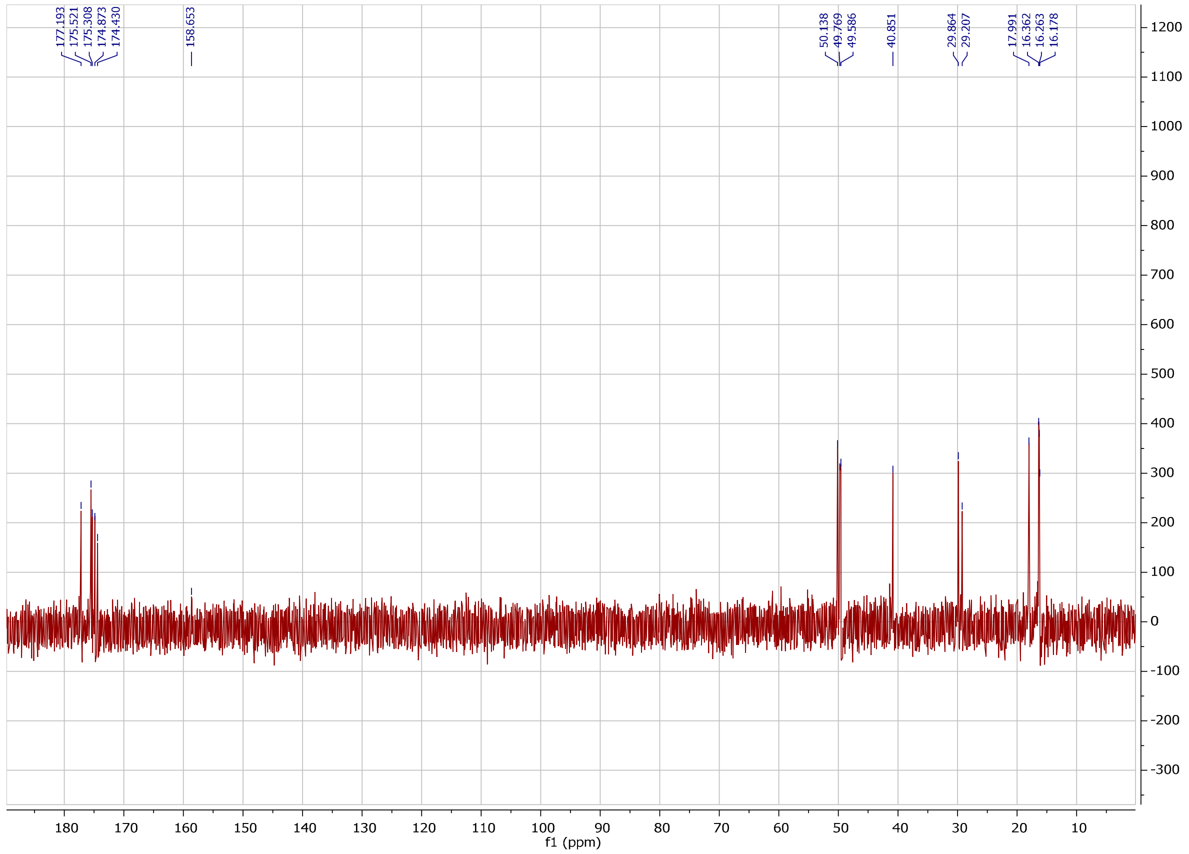
177.193
175.521
175.308
174.873
174.430
158.653
50.138
49.769
49.586
40.851
29.864
29.207
17.991
16.362
16.263
16.178
f1 (ppm)

SM: 7G

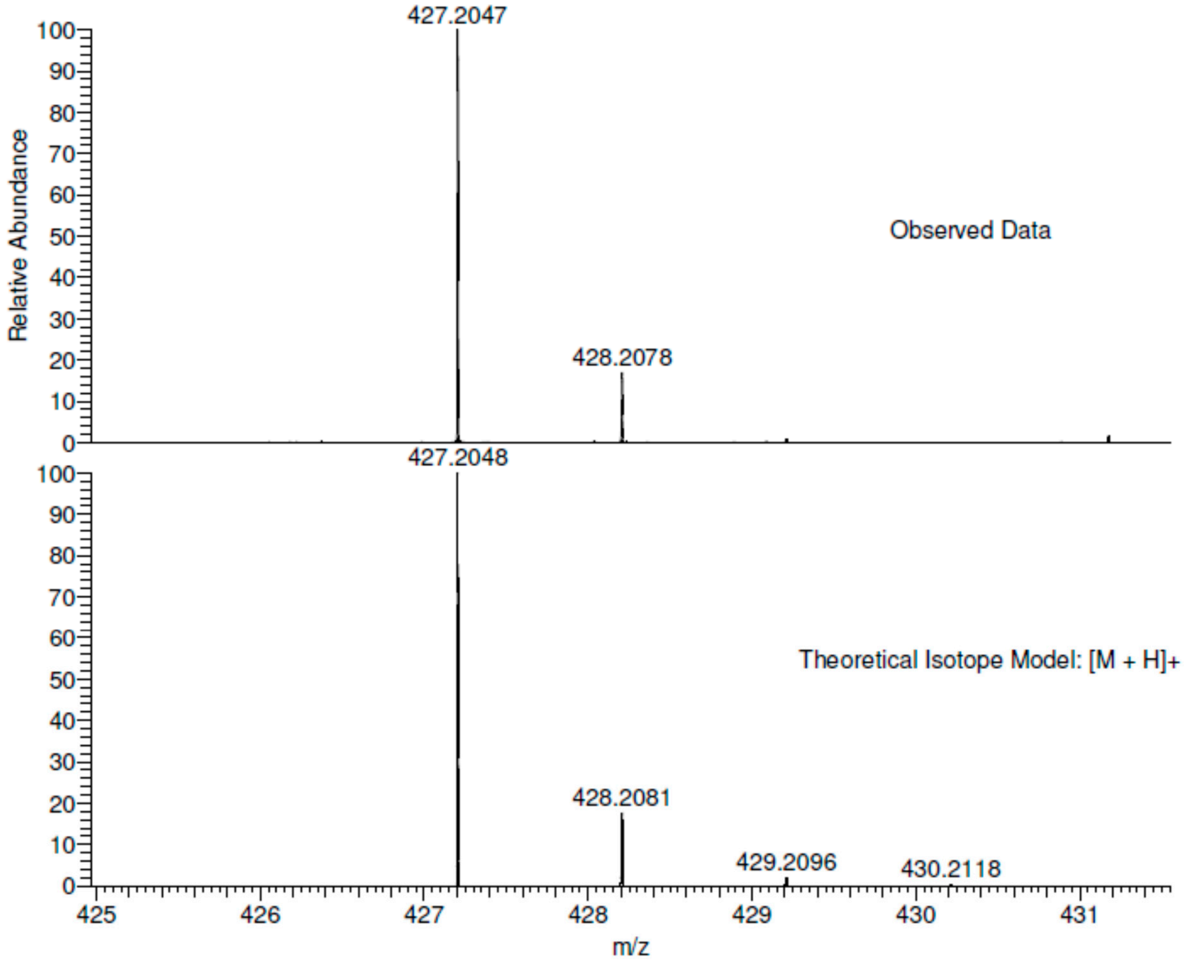

427.2047
Relative Abundance
Observed Data
428.2078
427.2048
Theoretical Isotope Model: [M + H]+
428.2081
429.2096
430.2118
m/z

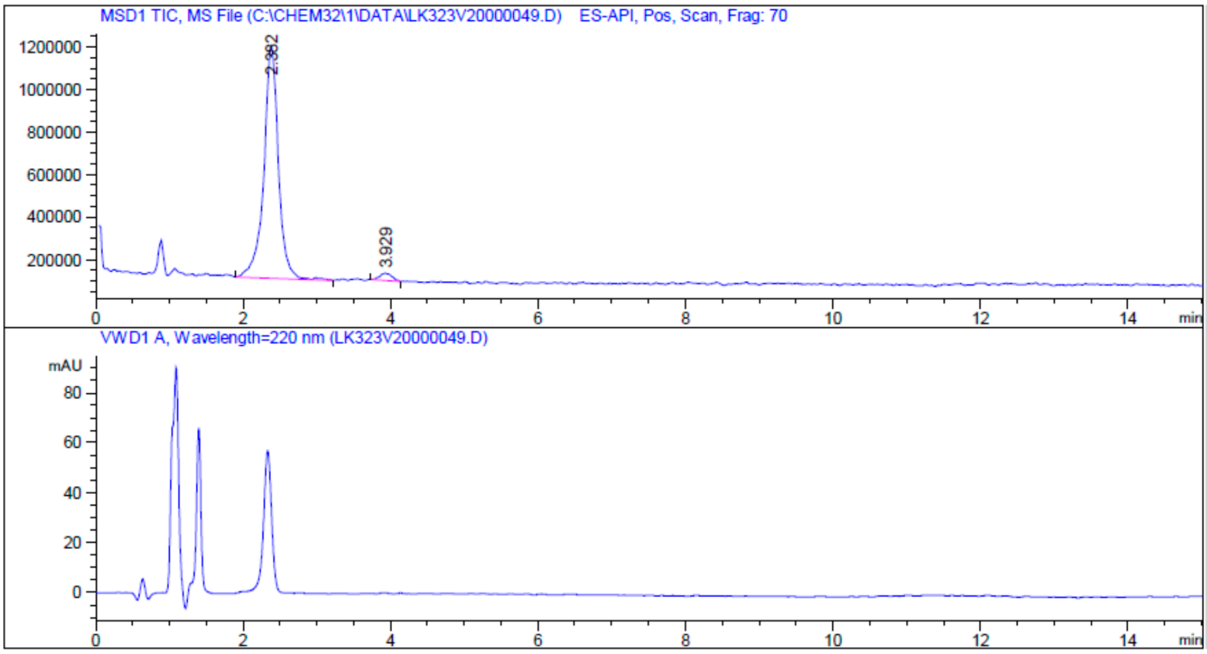


```
=====================================================================
                         Area Percent Report
=====================================================================

Sorted By             :      Signal
Multiplier:                   :      1.0000
Dilution:                     :      1.0000
Use Multiplier & Dilution Factor with ISTDs


Signal 1: MSD1 TIC, MS File

Peak RetTime Type  Width     Area      Height     Area
  #   [min]        [min]                            %
----|-------|----|-------|----------|----------|--------|
   1   2.382 BB    0.2094 1.50151e7  1.09338e6  97.3823
   2   3.929 BB    0.1539 4.03623e5  3.56642e4   2.6177

Totals :                  1.54188e7  1.12904e6
```

Microbiology

All MICs were determined using an agar dilution method.[16] This necessitated the use of a defined antagonist-free medium (peptone-free), prepared as previously described with the inclusion of 2% saponin-lysed horse blood, 25 µg/ml NAD and 25 µg/ml haemin.[46] Test compounds were dissolved in sterile deionised water and incorporated into the antagonist-free medium at a final concentration range of 1 – 128 mg/l. All bacteria were prepared from fresh (18 h) subcultures on blood agar. Each isolate was suspended in sterile deionised water to a density equivalent to 0.5 McFarland units using a densitometer (approx. 1.5 x 10^8 CFU/ml) and then diluted 1 in 15. A 1 µl aliquot of each diluted suspension was then delivered onto plates with a multipoint inoculator to give a final inoculum of approximately 10 000 or 150 000 CFU/spot as recommended.[16] All plates (including antimicrobial-free

controls) were incubated for 22 h at 37°C. The minimum inhibitory concentration (MIC) was recorded as the lowest concentration of test compound that resulted in complete inhibition of bacterial growth.

HPLC blanks

HPLC method A

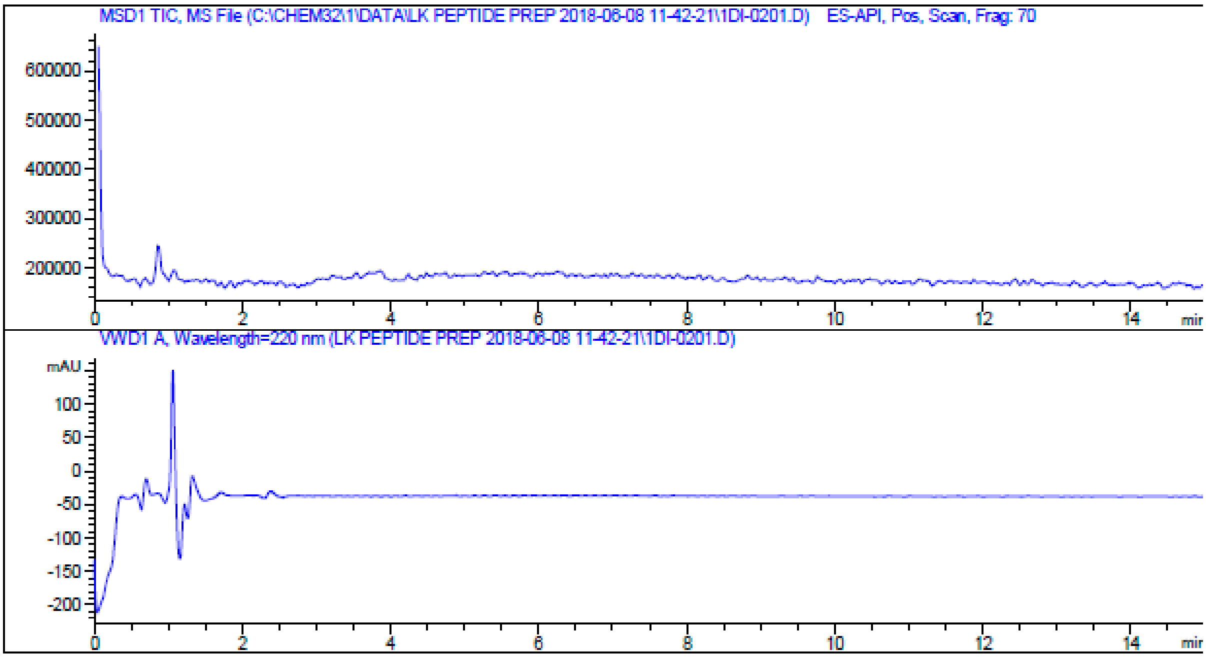

HPLC method B

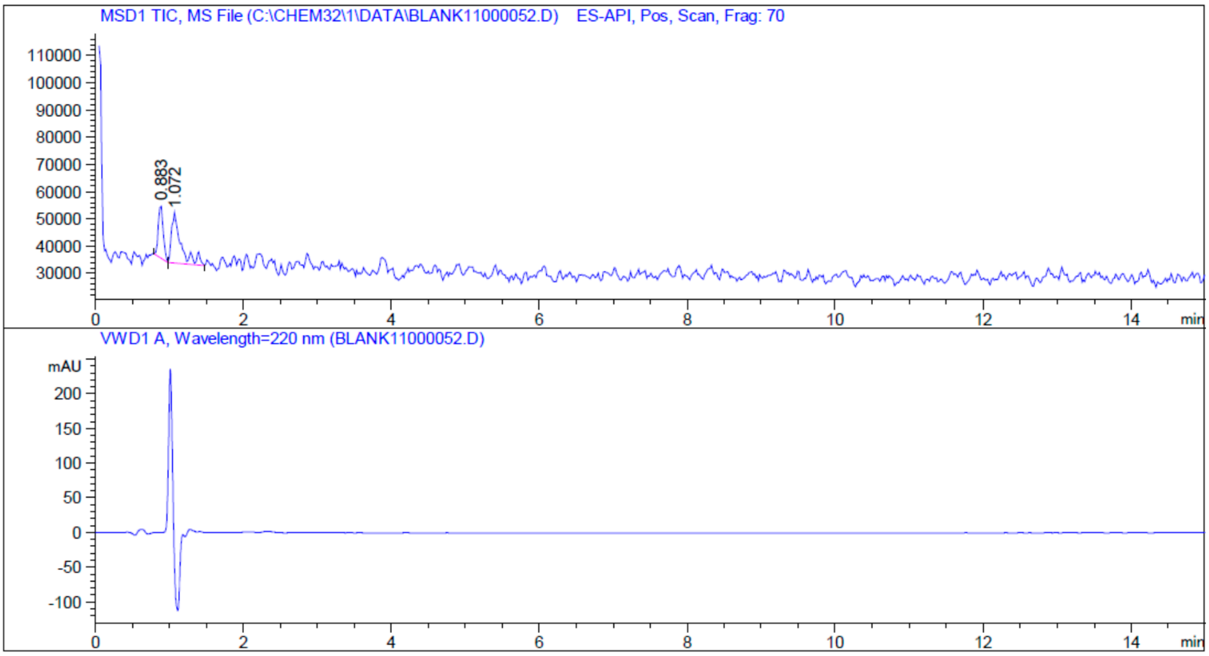

HPLC method C

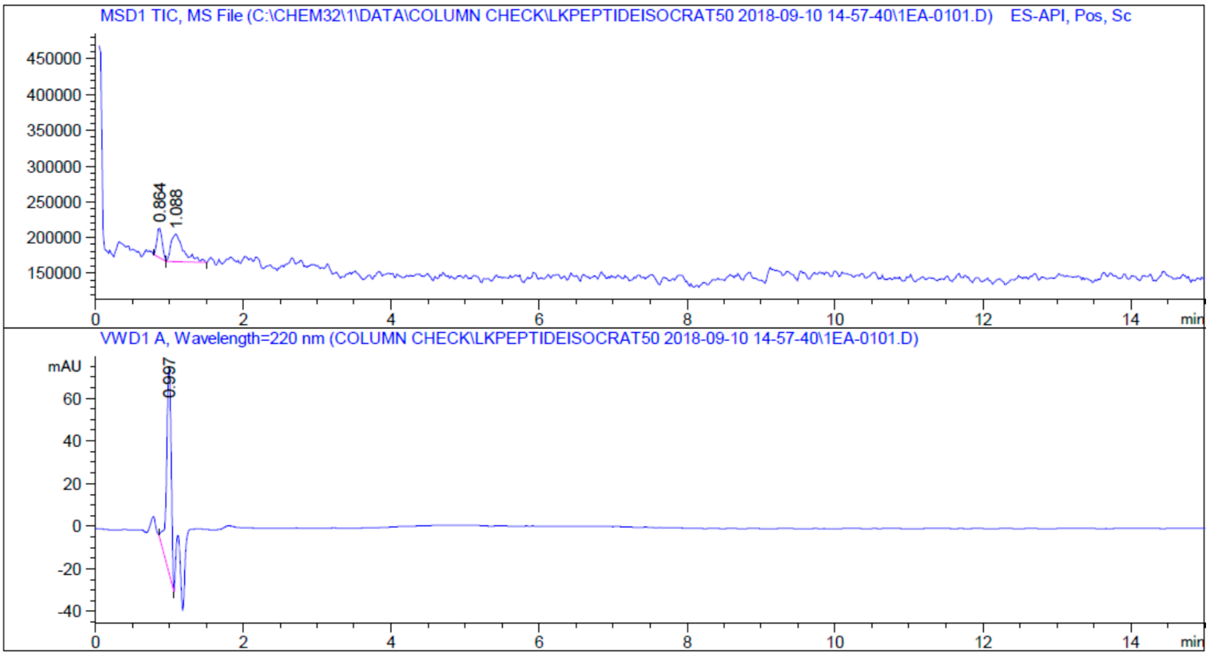

HPLC method D

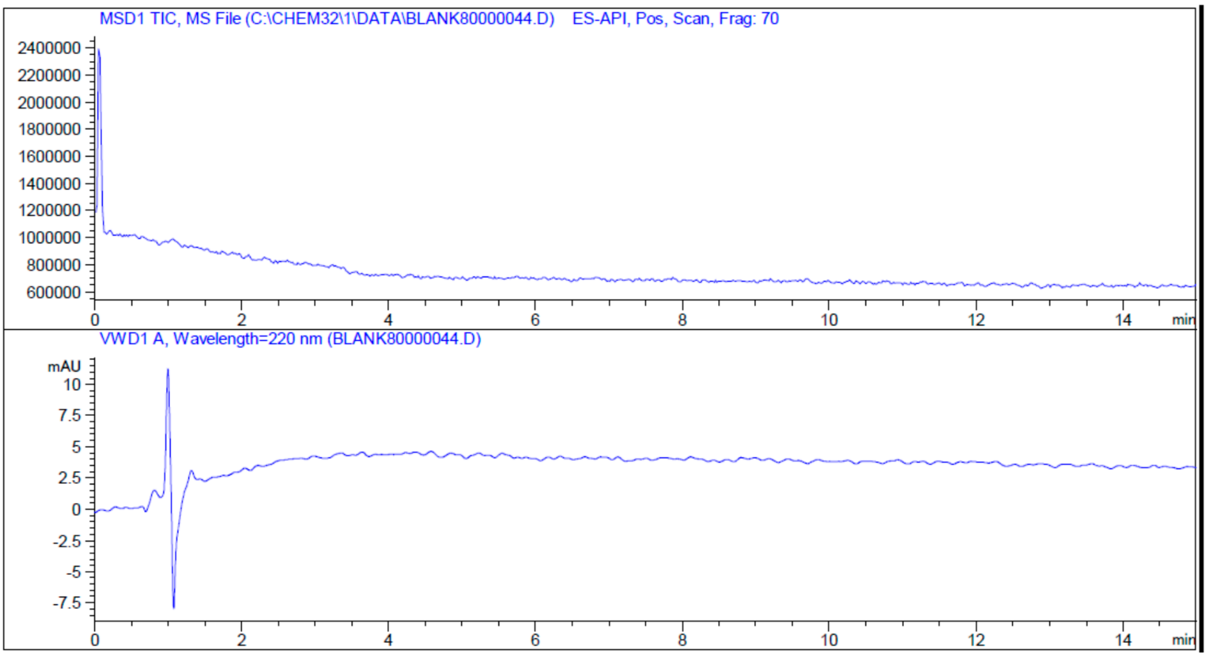

Supplement: Supplementary file 1 [file molecules-25-01315-s001.pdf]
